# Pyrodiversity interacts with rainfall to increase bird and mammal richness in African savannas

**DOI:** 10.1111/ele.12921

**Published:** 2018-02-14

**Authors:** Colin M. Beale, Colin J. Courtney Mustaphi, Thomas A. Morrison, Sally Archibald, T. Michael Anderson, Andrew P. Dobson, Jason E. Donaldson, Gareth P. Hempson, James Probert, Catherine L. Parr

**Affiliations:** ^1^ Department of Biology University of York Heslington York YO10 5DD UK; ^2^ York Institute for Tropical Ecosystems Environment Department University of York Heslington York YO10 5NG UK; ^3^ Institute of Biodiversity, Animal Health and Comparative Medicine University of Glasgow Glasgow G12 8QQ UK; ^4^ Centre for African Ecology School of Animal, Plant and Environmental Sciences University of the Witwatersrand Private Bag Johannesburg South Africa; ^5^ Natural Resources and the Environment CSIR PO Box 395 Pretoria 0001 South Africa; ^6^ Department of Biology Wake Forest University 049 Winston Hall Winston‐Salem North Carolina 27106 USA; ^7^ Ecology and Evolutionary Biology Princeton University Eno Hall Princeton NJ 08540 USA; ^8^ South African Environmental Observation Network (SAEON) Ndlovu Node Private Bag x1021 Phalaborwa Kruger National Park 1390 South Africa; ^9^ Department of Earth, Ocean & Ecological Sciences University of Liverpool Liverpool L69 3GP UK; ^10^ Department of Zoology & Entomology University of Pretoria Private Bag X20 Pretoria 0028 South Africa

**Keywords:** Bats, birds, conservation, fire, fire management, INLA, mammals, protected areas

## Abstract

Fire is a fundamental process in savannas and is widely used for management. Pyrodiversity, variation in local fire characteristics, has been proposed as a driver of biodiversity although empirical evidence is equivocal. Using a new measure of pyrodiversity (Hempson *et al*.), we undertook the first continent‐wide assessment of how pyrodiversity affects biodiversity in protected areas across African savannas. The influence of pyrodiversity on bird and mammal species richness varied with rainfall: strongest support for a positive effect occurred in wet savannas (> 650 mm/year), where species richness increased by 27% for mammals and 40% for birds in the most pyrodiverse regions. Range‐restricted birds were most increased by pyrodiversity, suggesting the diversity of fire regimes increases the availability of rare niches. Our findings are significant because they explain the conflicting results found in previous studies of savannas. We argue that managing savanna landscapes to increase pyrodiversity is especially important in wet savannas.

## Introduction

Fire is a key disturbance that plays a major role in determining the distribution of ecosystems (Bond *et al*. 2005; Bowman *et al*. 2009). Fire influences many ecological processes, including carbon storage (Williams *et al*. 2004), climate feedbacks (Beerling & Osbourne 2006) and tree recruitment (Bond 2008). It is a particularly important process underpinning the functioning of the tropical grassy biome (Parr *et al*. 2014). Savannas are a major component of this biome, and burning is widely used to manipulate habitats, yet biodiversity in savannas can be both positively and negatively impacted by fire. Using fire to maximise biodiversity requires detailed understanding of when, where and how often to burn (Andersen *et al*. 2012; Kelly & Brotons 2017).

Fires can be characterised by size, intensity, season and frequency of burning (Martin & Sapsis 1992). These attributes vary along gradients of primary productivity, human activity and vegetation, but geographical patterns exist that allow fire regimes to be classified globally (Archibald *et al*. 2013). No two fires are alike, and the variability among fires within a region generates ‘pyrodiversity’ (Martin & Sapsis 1992), an often overlooked emergent property of a fire regime. Martin & Sapsis (1992) argued that ‘pyrodiversity begets biodiversity’ in recognition that spatial and temporal variation in the attributes of fires may enhance the diversity of ecological niches thereby enhancing diversity.

**Figure 1:**
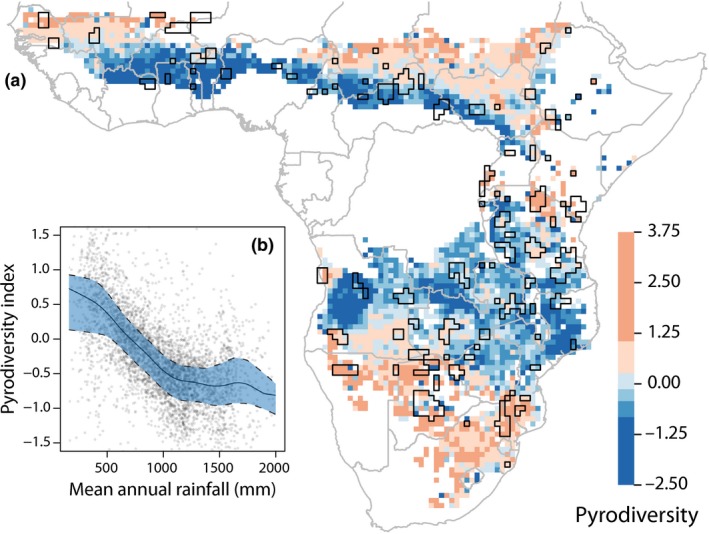
Pyrodiversity in the savannas of Africa is largely determined by rainfall. (a) Map of pyrodiversity across Africa at quarter degree resolution, with polygons encircling cells containing > 20% protected area overlaid in black. (b) The pyrodiversity rainfall relationship across Africa (from Hempson *et al*. 2017).

Pyrodiversity has been hard to quantify: it is multifaceted (Bowman *et al*. 2016) and seldom has variability in more than two fire attributes been considered simultaneously (although see Ponisio *et al*. 2016). We recently developed an index of pyrodiversity based on variability in four fire characteristics identified by Martin & Sapsis: size, season, return interval and intensity (Hempson *et al*. 2017). This index reveals that pyrodiversity varies significantly but predictably across African savannas (Fig. 1). Rainfall is the main driver, with highest pyrodiversity in low rainfall areas (<650 mm year^−1^), and lower pyrodiversity in wetter regions.

A common view is that pyrodiversity promotes biodiversity in flammable systems by generating spatial and temporal habitat heterogeneity (Martin & Sapsis 1992; Parr & Andersen 2006). This understanding underpins the widely applied practice of patch mosaic burning (Brockett *et al*. 2001). The pyrodiversity‐biodiversity hypothesis assumes that species differ in their response to fire and consequently patchy burning provides a range of habitats through space and time, promoting biodiversity (Burrows & Wardell‐Johnson 2003; Panzer 2003).

Although the pyrodiversity‐biodiversity hypothesis is appealing and has received support in forested ecosystems (Ponisio *et al*. 2016; Tingley *et al*. 2016), it has received limited support in savanna biomes studying several taxa at scales from a hectares to hundreds of square kilometres (Parr *et al*. 2004; Taylor *et al*. 2012, Davies *et al*. 2012; Farnsworth *et al*. 2014; but see Maravalhas & Vasconcelos 2014 and Ponisio *et al*. 2016). Indeed, in savannas, most studies emphasise the high resilience of biodiversity to burning. For example in semiarid mopane in South Africa, ant composition did not differ among six experimentally maintained fire regimes that ranged between annual burning vs. 50 years of fire exclusion (Parr *et al*. 2004). Similarly, in Australia, diversity of time since fire did not influence bird (Taylor *et al*. 2012) or reptile (Farnsworth *et al*. 2014) diversity. From a management perspective, these findings suggest that pyrodiversity is unimportant in many systems, with global drivers such as energy and water likely far more important (Hawkins *et al*. 2003).

Most previous studies occurred at one location or across a narrow environmental range, but the effect of pyrodiversity on biodiversity may be rainfall contingent. Although highest pyrodiversity occurs in drier savannas (<650 mm year^−1^), Hempson *et al*. (2017) predict that pyrodiversity promotes biodiversity (here, species richness) mainly in regions with intermediate rainfall of 650–1300 mm year^−1^ (here termed ‘wet savannas’). This prediction (and the 650 mm division) is based on the stronger effect of fire on vegetation structure in regions with intermediate rainfall (Sankaran *et al*. 2005; Higgins *et al*. 2007; Smit *et al*. 2010). In wet savannas there is enough rainfall for a substantial woody component, but short return intervals and high fire intensities can prevent sapling recruitment into adult size classes, thus reducing tree size‐class distribution and biomass (Bond 2008). It is thus possible to have large variation in woody vegetation structure in wet savannas if fire history is variable. In dry savannas both tree heights and densities are limited by rainfall, so fire has less influence on woody structure. Many taxonomic groups show strong responses to woody structure – particularly birds (MacArthur & MacArthur 1961), mammals (Olff *et al*. 2002) and reptiles (Donihue *et al*. 2013). Thus, the greater effect of fire on woody structure in wet savannas should hold greater implications for biodiversity. Nonetheless, it is possible that biodiversity responds directly to pyrodiversity if increased variation stretches resilience (Bird *et al*. 2012). In this case, we expect the response to be similar across rainfall gradients, or even larger for dry environments with greater pyrodiversity (Hempson *et al*. 2017).

Here we present the first continental test of the pyrodiversity‐biodiversity hypothesis in savannas using the pyrodiversity index developed by Hempson *et al*. (2017) and bird and mammal richness from protected areas across Africa. We restrict ourselves to analysis of richness within the savanna biome (not between savanna and forest biomes which may exist as alternative states modulated by fire: Bond *et al*. 2005). Specifically, we test how species richness (an important component of biodiversity) varies with an index of pyrodiversity based on fire size, intensity, seasonality and fire return interval. We test the hypothesis that pyrodiversity promotes richness to a greater extent in wet (> 650 mm year^−1^) than in drier savannas, due to the potential for larger effects on woody vegetation structure. We also predict that smaller, less mobile mammals would respond positively to pyrodiversity, though fire size may be important (Lawes *et al*. 2015). Bird and bat richness are predicted to respond similar to pyrodiversity. Our analyses are limited to protected areas because vertebrate richness is best surveyed and least disturbed in these areas. Due to the substantial positive impact of protected area status on biodiversity (Gray *et al*. 2016), we also examine how pyrodiversity could mediate this effect by comparing fire attributes inside and outside of protected areas.

## Methods

To test hypotheses concerning the effect of pyrodiversity on species richness we collated data on (1) fires, (2) the distributions of birds and mammals, (3) rainfall and (4) topographic heterogeneity within protected areas on Africa's savannas, and fitted a set of spatially explicit regression models.

### Fire data

We extracted data on individual fires from the MODIS burned‐area product MCD45A1 and the MODIS active‐fire product MCD14ML from April 2000 to June 2015. Full details of the pyrodiversity index are given in Hempson *et al*. (2017), but in brief we used a flood‐fill algorithm to identify individual fires for Sub‐Saharan Africa, south of 10° North following Archibald *et al*. (2010). For each fire we calculated log area (km^2^; area: variable *logArea*); probable date of ignition relative to local peak of the wet season (number of days between the earliest burn date within the fire and the 15th of the month with the highest monthly rainfall locally; Season: *Fireday*); time since last burn (the log mean number of days since an earlier fire for all pixels in the fire; fire return interval or *FRI*) and log fire radiative power (FRP), an index of fire intensity (see Archibald *et al*. 2010). Due to false positives and negatives in both MODIS data sets, not all fires were associated with an FRP value, and not all FRP values were associated with a fire: we used only fires where both were recorded. This produced a data set of 2 million individual fires from which we calculated pyrodiversity. As any fire can be located as a point within the four‐dimensional space described by the four fire attributes (after normalising by each attribute's standard deviation), pyrodiversity is computed as the volume of the minimum convex hull of the four‐dimensional space described by all the fires within a 30 arcminute cell, using a nonparametric bootstrap to correct volume for sample size (Hempson *et al*. 2017). Pyrodiversity is therefore an emergent property of the fire regime, describing spatial and temporal variation in fire type.

Animal distributions can reflect conditions over decades, whereas fire data are available recently: we make a strong assumption that pyrodiversity since 2000 reflects levels over recent decades. For two protected areas in South Africa fire records based on field maps are available since 1970 and allow us to test this assumption. To do so, we compared spatial variation in fire attributes since 2000 with historic data (Appendix S1). This revealed consistent correlations and gives us confidence that recent variation can reflect historical patterns.

### Rainfall data

We compiled mean annual precipitation data for each grid cell using the Climatic Research Unit (CRU) TS3.10 monthly 0.5° data set (Harris *et al*. 2014). This global‐scale product is an interpolated monthly data set based on meteorological station observations from 1961 to 2009. We identified wet (> 650 mm year^−1^) and dry (< 650 mm year^−1^) savannas. Note that wet savannas largely correspond with the intermediate rainfall areas in Hempson *et al*. (2017; i.e. 650–1300 mm year^−1^), and that climatological data are computed over a longer time series than the satellite‐derived data sets since (1) more data are available and (2) climate is best described by long‐term averages.

### Species richness

We focused on mammal and bird richness as the ranges of these species are relatively well‐known compared to other taxonomic groups. We defined savanna habitat using White (1983; see Supporting Information, Appendix S1) and a minimum rainfall threshold of 300 mm year^−1^. Our goal was to include only bird and mammal species known to occur in savannas, based on descriptions from literature and guidebooks. Non‐savanna dependent species could have ranges encompassing the savanna biome if they either occupy those savanna areas transiently or if they occupy patches of non‐savanna habitat which are too small (e.g. riverine forests) to be mapped at the spatial grain of our study. We validated our species lists using checklist data (Appendix S1).

### Mammal diversity

We compiled mammal species richness within quarter degree (30 min × 30 min) scales using range maps from the IUCN Red List of Threatened Species mammal diversity database <http://www.iucnredlist.org/technical-documents/spatial-data>. To explore detailed hypotheses about different taxonomic or trait‐based subsets of the data we calculated the richness of five data partitions: (1) bat species; (2) mammals excluding bats; (3) small mammals, defined by the lower 50th percentile of mean body mass; (4) large mammals, defined by the upper 50th percentile of mean body mass; and (5) common species, defined by the 50% most widely distributed species. Body sizes were assembled from primary literature and guidebooks, using average body mass (kg) of adult males and females, a full list is provided in Appendix S2.

### Bird diversity

Distributions of all African breeding bird species were obtained from the Copenhagen Museum (Hansen *et al*. 2007). We identified species associated with savanna habitats from habitat descriptions in Sinclair & Ryan (2003) and online resources. A full list of 819 species included is provided in Appendix S3. In addition to overall richness, we calculated richness of common species, defined as the 50% most widely distributed species.

### Net primary productivity

Net primary productivity (NPP) is widely used as the basis for understanding macro‐scale productivity‐diversity relationships (e.g. Mittelbach *et al*. 2001). We included NPP (kg C m^−2^) estimates from Terra MODIS‐1 km GPP/NPP MOD17 from 2001 to 2010. We stacked annual images and averaged pixels across years.

### Protected areas

We confined our analysis to protected areas (PAs) to focus on the dynamics of fire in landscapes relatively less modified by humans. In these areas, fire is a complex function of grazing pressure, rainfall, fuels and human fire management activities (Govender *et al*. 2006). We used the World Database on Protected Areas to define boundaries of protected areas (IUCN & UNEP‐WCMC 2015). We used only those PAs classified as primarily managed for biological conservation within the savanna biome (Fig. 1). Within each PA we extracted the richness of all bird and mammal groupings within grid cells covered by at least 50% of the protected area polygon.

### Data analysis

To test hypotheses concerning the impact of pyrodiversity on richness we fitted a series of conditional autoregressive models using Integrated Nested Laplace Approximation (INLA). INLA provides a Bayesian framework for approximating posterior parameter estimates in a computationally efficient manner (Rue *et al*. 2009; Lindgren *et al*. 2011) and conditional autoregressive models perform well in comparisons of spatial regression models (Beale *et al*. 2010). All analyses were undertaken in R using the R‐INLA package (Martins *et al*. 2013; R core team 2016) using vague priors (e.g. we used log‐gamma priors for the spatial effect, with shape parameters α = 0.1, β = 0.5 that have negligible structure across meaningful parameter space). Due to strong correlations between the mean and coefficients of variation in the individual fire attributes we could not include all variables in a single global model (covariate plots: Supplementary Figs. 1–6). Consequently, we built two classes of model for each richness data set: one modelling richness from pyrodiversity and the means of the four individual fire attributes in each cell, the second modelling richness from pyrodiversity and the coefficient of variation in the four individual fire attributes. Because our index of pyrodiversity is a volume and not a simple linear combination of the four individual components it is reasonable to model the contribution of both index and individual components simultaneously. In both sets of models we included a generalised additive model (GAM) with two knots to describe the relationship between richness and NPP and a linear effect of topographic heterogeneity. The first model allows us to test whether pyrodiversity and individual fire attributes are correlated with richness, the second model allows us to test whether pyrodiversity *per se* is correlated with richness, or whether any such relationship is a response to variation in individual fire attributes. This allows us to address whether pyrodiversity is more than the sum of the variation in each of its individual components. To test whether relationships with fire covariates are similar in wet and dry savanna within a single model, we fitted an interaction between covariates and rainfall. We repeated the analysis using quadratic terms for all covariates, to assess whether the main results were affected by nonlinearity in relationships and compared models using wAIC (Gelman *et al*. 2013). The hierarchical model described is equivalent to that described in detail by Beale *et al*. (2010) which deals well with spatial autocorrelation. In keeping with the Bayesian framework provided by INLA, we assessed the support for each parameter in the model by examining the 95% credible intervals. All models and parameter estimates are provided in Supporting Information (Appendix S4, Supplementary Figures 7‐86).

### Inside vs outside protected areas

Although our pyrodiversity‐richness analysis focused on protected areas, we were also interested in how pyrodiversity and its individual attributes might differ outside PAs where human can strongly influence the characteristics of fire. We buffered PAs by 100 km and calculated pyrodiversity attributes in all buffer cells, with an inclusion criterion that at least 20% of cells fell within the buffer area. We verified that smaller buffer sizes (50 km) produced qualitatively similar results (Supporting Information), but because 100 km was of similar scale to many PAs, we report results from the larger buffer. We used Generalised Linear Mixed Models (GLMM) to estimate the effect of PAs on the mean and variation (CV) of pyrodiversity and its four individual attributes, using PA identity as a random effect and PA status (inside vs. outside) as the fixed effect of interest (package *lmer* in R).

## Results

### Pyrodiversity‐richness relationships

**Figure 2:**
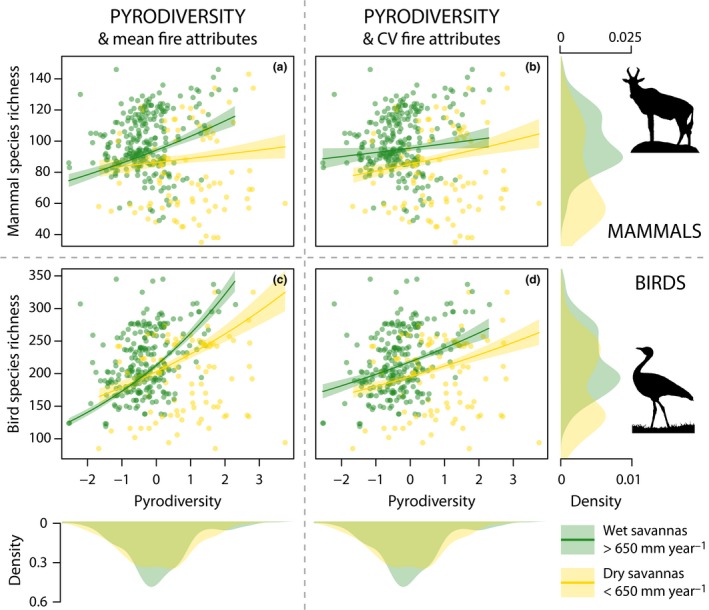
The relationships between pyrodiversity and mammal (top row) and bird (lower row) richness predicted from models without pyrodiversity estimated from models fitted with mean (a) and (c) and variation of fire attributes (coefficient of variation, cv (b) and (d)). Within each plot, the results for wet savannas (> 650 mm year^−1^, green points) and drier savannas (< 650 mm year^−1^, yellow points) are shown with median estimate and 95% confidence intervals (shaded). Confidence intervals are computed from models that include all fixed and spatially explicit random effects: the presence of strong spatial effects generates wider scatter in the points than may be expected from plotted confidence intervals. Density plots show density of wet and dry savannas along their respective axes: note lower pyrodiversity but higher richness and steeper relationships in wet savannas.

**Figure 3:**
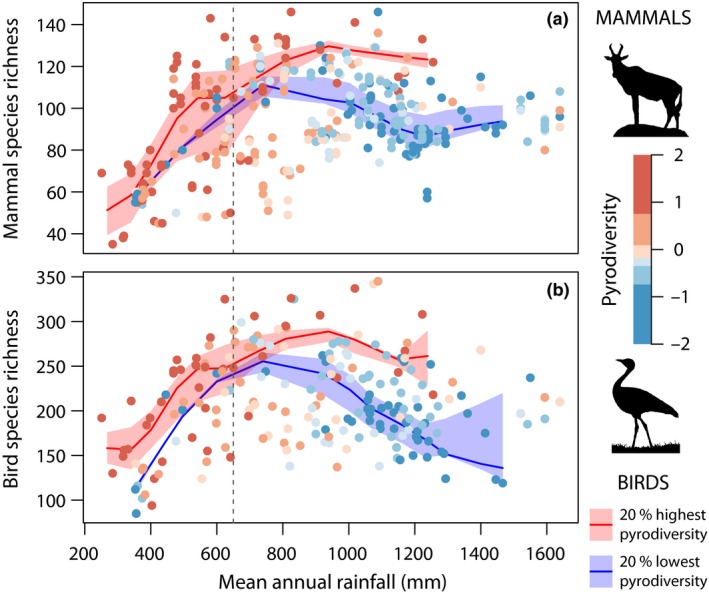
Relationship between mean annual rainfall and biodiversity illustrating the interaction between rainfall and pyrodiversity for (a) mammals and (b) birds. Colours identify 20% quantiles of pyrodiversity, with the exception of palest colours which represent 10% quantiles around the median, red line and shading indicates a loess regression of the top 20% most pyrodiverse sites with 80% confidence intervals, blue lines the 20% least pyrodiverse sites. The data presented are the same as in Figure 2 where the statistical modelling is accurately presented, but redrawn here to illustrate the pattern more clearly using raw data.

Although all models showed significant unexplained spatial variation modelled by the spatial effect, the expected positive relationships were found between each richness measure and both NPP and topographic variation (Supporting Information). Overall, there was strong support for a positive correlation between pyrodiversity and richness, particularly in wet savannas, where high pyrodiversity sites had reliably high biodiversity (Fig. 2; Tables 1 and 2 and Figs. S7‐80). Models with quadratic relationships were generally better supported than those with linear terms alone, but results were qualitatively similar (Supplementary Appendix 4, where full results exist). Due to the potential of overfitting highly parameterised models we discuss linear results here. Pyrodiversity was strongly correlated with richness of all taxon groups in wet savanna (particularly birds) in the mean effect models, but had much smaller and often only weakly supported effects in dry savanna (Table 1). In very high rainfall savanna regions (>1000 mm year^−1^), the 20% most pyrodiverse sites had 27% more mammal species and 40% more bird species than the 20% lowest pyrodiversity sites (Fig. 3). In models where pyrodiversity was modelled alongside variation in individual fire attributes (rather than mean values) pyrodiversity retained a positive correlation with species richness for most taxon groups, including all birds and all mammals, (Table 2), particularly in wet savannas.

**Table 1:** Raw mean effects of the INLA analysis for pyrodiversity overall and individual fire attributes (controlling for the effect of NPP), for wet (> 650 mm year^−1^) and wet (< 650 mm year^−1^) savannas

	Pyrodiversity		FRI		FRP		Seasonality		Area	
	Wet	Dry	Wet	Dry	Wet	Dry	Wet	Dry	Wet	Dry
Mammals	**0.091**	0.029	−**0.054**	−**0.107**	−**0.026**	−0.034	0.001	**0.088**	−**0.076**	0.005
	**(0.070, 0.113)**	(−0.025, 0.083)	**(**−**0.076,** −**0.033)**	**(**−**0.164,** −**0.051)**	**(**−**0.051,** −**0.002)**	(−0.094, 0.025)	(−0.018, 0.020)	**(0.038, 0.138)**	**(**−**0.096,** −**0.056)**	(−0.044, 0.054)
Mammals: no bats	**0.099**	0.020	−0.011	−**0.081**	0.002	−0.011	−0.010	**0.073**	−**0.044**	0.007
	**(0.075, 0.123)**	(−0.039, 0.079)	(−0.035, 0.014)	**(**−**0.144,** −**0.019)**	(−0.026, 0.028)	(−0.076, 0.053)	(−0.032, 0.011)	**(0.017, 0.128)**	**(**−**0.067,** −**0.022)**	(−0.047, 0.061)
Mammals: bats	**0.062**	0.093	−**0.155**	−**0.195**	−**0.087**	−**0.167**	0.018	**0.193**	−**0.145**	−0.011
	**(0.023, 0.101)**	(−0.015, 0.200)	**(**−**0.196,** −**0.115)**	**(**−**0.313,** −**0.079)**	**(**−**0.134,** −**0.040)**	**(**−**0.287,** −**0.047)**	(−0.017, 0.053)	**(0.087, 0.300)**	**(**−**0.183,** −**0.108)**	(−0.110, 0.088)
Common mammals	**0.066**	0.021	−**0.065**	−**0.101**	−**0.031**	−**0.081**	−0.019	0.034	−**0.044**	0.029
	**(0.042, 0.090)**	(−0.041, 0.083)	**(**−**0.090,** −**0.041)**	**(**−**0.166,** −**0.035)**	**(**−**0.059,** −**0.003)**	**(**−**0.149,** −**0.013)**	(−0.041, 0.003)	(−0.024, 0.093)	**(**−**0.067,** −**0.022)**	(−0.027, 0.085)
Large mammals	**0.086**	0.044	−**0.053**	−**0.114**	−**0.037**	−0.058	−0.001	**0.118**	−**0.074**	0.009
	**(0.062, 0.111)**	(−0.019, 0.106)	**(**−**0.077,** −**0.028)**	**(**−**0.181,** −**0.049)**	**(**−**0.066,** −**0.009)**	(−0.127, 0.011)	(−0.022, 0.021)	**(0.058, 0.177)**	**(**−**0.097,** −**0.051)**	(−0.048, 0.067)
Small mammals	**0.092**	0.004	−**0.048**	−0.076	0.014	0.011	0.000	0.035	−**0.074**	−0.008
	**(0.054, 0.130)**	(−0.089, 0.098)	**(**−**0.088,** −**0.008)**	(−0.177, 0.025)	(−0.030, 0.057)	(−0.092, 0.113)	(−0.035, 0.034)	(−0.053, 0.124)	**(**−**0.111,** −**0.038)**	(−0.094, 0.078)
Birds	**0.207**	**0.126**	−0.017	−**0.178**	−**0.085**	−0.039	**0.016**	**0.055**	−**0.042**	**0.079**
	**(0.189, 0.225)**	**(0.073, 0.179)**	(−0.034, 0.000)	**(**−**0.230,** −**0.128)**	**(**−**0.107,** −**0.063)**	(−0.094, 0.017)	**(0.000, 0.032)**	**(0.015, 0.095)**	**(**−**0.059,** −**0.026)**	**(0.033, 0.125)**
Common birds	**0.090**	**0.061**	0.010	−**0.069**	−**0.067**	−**0.094**	−**0.033**	0.003	**0.027**	**0.071**
	**(0.071, 0.109)**	**(0.012, 0.110)**	(−0.009, 0.029)	**(**−**0.119,** −**0.019)**	**(**−**0.090,** −**0.044)**	**(**−**0.149,** −**0.040)**	**(**−**0.050,** −**0.016)**	(−0.040, 0.047)	**(0.009, 0.045)**	**(0.025, 0.117)**

Medians with lower (0.025) and upper (0.975) quantiles shown in brackets. Well‐supported effects are in bold font.

**Table 2:** Raw variation (coefficient of variation) effects of the INLA analysis for pyrodiversity and fire attributes, separated between wet (> 650 mm year^−1^) and wet (< 650 mm year^−1^) savannas

	Pyrodiversity		FRI		FRP		Seasonality		Area	
	Wet	Dry	Wet	Dry	Wet	Dry	Wet	Dry	Wet	Dry
Mammals	**0.029**	0.055	−0.001	−0.033	0.002	0.012	**0.064**	−0.054	−0.005	−0.045
	**(0.002, 0.057)**	(−0.011, 0.120)	(−0.029, 0.027)	(−0.097, 0.031)	(−0.023, 0.027)	(−0.097, 0.031)	**(0.043, 0.086)**	(−0.109, 0.001)	(−0.034, 0.024)	(−0.112, 0.021)
Mammals: no bats	**0.044**	0.031	0.026	−0.022	−0.004	0.004	**0.062**	−0.036	0.024	−0.014
	**(0.013, 0.075)**	(−0.042, 0.103)	(−0.006, 0.058)	(−0.095, 0.05)	(−0.032, 0.023)	(−0.095, 0.050)	**(0.037, 0.086)**	(−0.097, 0.024)	(−0.008, 0.057)	(−0.088, 0.061)
Mammals: bats	−0.006	**0.198**	−**0.069**	−0.101	0.019	0.063	**0.068**	−**0.210**	−**0.077**	−**0.204**
	(−0.058, 0.045)	**(0.067, 0.330)**	**(**−**0.122,** −**0.017)**	(−0.228, 0.026)	(−0.027, 0.067)	(−0.228, 0.026)	**(0.027, 0.108)**	**(**−**0.333,** −**0.089)**	**(**−**0.130,** −**0.023)**	**(**−**0.333,** −**0.075)**
Common mammals	0.015	0.046	−0.019	−0.016	0.009	0.000	**0.047**	−0.043	0.028	−**0.084**
	(−0.017, 0.046)	(−0.029, 0.122)	(−0.051, 0.013)	(−0.090, 0.058)	(−0.019, 0.038)	(−0.090, 0.058)	**(0.022, 0.072)**	(−0.107, 0.021)	(−0.005, 0.062)	**(**−**0.161,** −**0.007)**
Large mammals	0.030	**0.077**	0.001	−0.040	0.007	0.013	**0.055**	−**0.083**	0.002	−0.059
	(−0.002, 0.061)	**(0.002, 0.153)**	(−0.032, 0.033)	(−0.115, 0.034)	(−0.021, 0.036)	(−0.115, 0.034)	**(0.030, 0.080)**	**(**−**0.149,** −**0.018)**	(−0.031, 0.035)	(−0.136, 0.018)
Small mammals	0.026	0.014	−0.001	−0.020	−0.018	0.013	**0.084**	−0.006	−0.018	−0.018
	(−0.023, 0.076)	(−0.102, 0.130)	(−0.053, 0.051)	(−0.137, 0.096)	(−0.061, 0.026)	(−0.137, 0.096)	**(0.045, 0.122)**	(−0.101, 0.088)	(−0.070, 0.034)	(−0.136, 0.099)
Birds	**0.092**	**0.079**	**0.086**	−0.027	**0.041**	0.005	**0.062**	0.000	−0.018	−0.056
	**(0.069, 0.116)**	**(0.023, 0.135)**	**(0.062, 0.111)**	(−0.082, 0.028)	**(0.019, 0.063)**	(−0.082, 0.028)	**(0.045, 0.080)**	(−0.043, 0.043)	(−0.043, 0.006)	(−0.113, 0.000)
Common birds	**0.036**	**0.063**	**0.060**	0.003	0.015	0.005	**0.023**	−0.004	**0.046**	−**0.082**
	**(0.012, 0.060)**	**(0.007, 0.119)**	**(0.035, 0.085)**	(−0.052, 0.059)	(−0.006, 0.037)	(−0.052, 0.059)	**(0.004, 0.041)**	(−0.050, 0.041)	**(0.020, 0.072)**	**(**−**0.141,** −**0.024)**

Medians with lower (0.025) and upper (0.975) quantiles shown in brackets. Well‐supported effects shown in bold font.

Several additional correlations between richness and individual attributes of fire were also identified (Figs. S7‐54). Mean fire return interval generally showed a negative correlation with species richness in both wet and dry savannas and was often the strongest correlation identified (Table 1, FRI). In contrast, variability in fire return interval rarely correlated with species richness, with mixed directionality of effect (Table 2, FRI). Thus, areas with relatively frequent fires generally have higher species richness in all savanna types – an effect that was independent of the correlation between NPP and FRI.

Mean fire size had contrasting effects in wet and dry savannas (Tables 1 and 2, Area). In wet savannas fire size had a strong and consistent negative correlation with species richness, whereas variation in fire size was rarely supported. In contrast, in dry savannas only avian diversity showed a positive relationship with mean fire size; variation in fire size was marginally more important than mean size and suggested reduced variation is associated with higher species richness. Consequently, in wet savannas a consistently small fire size is associated with higher species richness, whereas in dry savannas fire size *per se* is less important overall, but richness is higher where fires are of consistent size; only bird diversity increasing in areas with larger fires.

Although mean fire radiative power (FRP: fire intensity) had a smaller average effect than fire return interval or fire size, this was not consistent for all taxonomic groups; when significant, it always showed a negative correlation with species richness (Table 1, FRP). There was negligible support for an additional effect of variation in fire intensity, over and above its effect on pyrodiversity. Finally, one of the most consistent effects in wet savannas was a well‐supported positive correlation between variability in fire seasonality and species richness (Table 2, Seasonality). There were few well‐supported correlations in dry savanna: generally, these were smaller, negative associations with variation in fire seasonality (Table 2, Seasonality). Seasonality showed few correlations with richness in wet savannas and was more important in dry savannas, where higher richness was sometimes associated with later average burning. Thus, in dry savannas higher species richness is associated with consistent late season burning. In contrast, in wet savanna higher species richness is found in areas with high variation in fire season. The consistent association between variability in seasonality and species richness in wet savannas is, in fact, the only variability measure that shows the same association with richness as overall pyrodiversity and consequently probably explains why the pyrodiversity variable was less important in models including variation than in the mean effect models.

### Pyrodiversity effects on biodiversity groups

**Figure 4:**
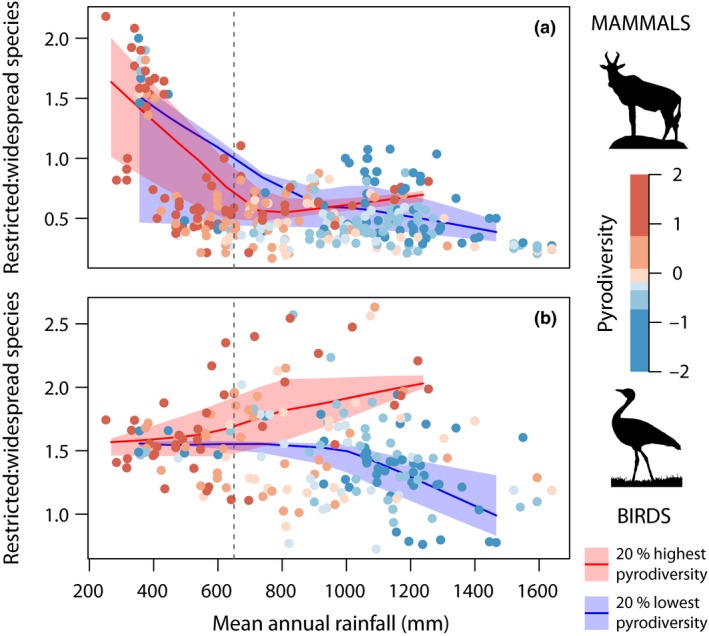
Relationship between mean annual rainfall and the ratio of restricted to widespread mammal (a) and bird (b) species, with loess regression by pyrodiversity quantile as Fig. 2: Colours identify 20% quantiles of pyrodiversity, with the exception of palest colours which represent 10% quantiles around the median, red line and shading indicates a loess regression of the top 20% most pyrodiverse sites with 80% confidence intervals, blue lines the 20% least pyrodiverse sites.

Overall, in wet savannas the existence of a positive correlation between pyrodiversity and species richness in all taxon groups was well supported, but there were differences between taxon groups with respect to the relative importance of various components of pyrodiversity. Birds were the most responsive group, and the only group to respond to pyrodiversity in dry environments (Table 1, Fig. 3). Although common birds and all birds showed broadly similar patterns, the contribution of restricted range species to overall diversity declines slightly in wet savannas with low pyrodiversity, whereas remaining comparably diverse in dry savannas (i.e. range‐restricted birds are underrepresented where pyrodiversity is low: Fig. 4b).

Contrary to expectation, small mammals showed fewer correlations with pyrodiversity than large mammals or other taxonomic groups (compare row 6 with others in Tables 1 and 2). We found no strong relationship in the relative diversity of restricted range mammals that could be attributed to pyrodiversity (Fig. 4a). Other taxonomic effects were marginal, although birds showed slightly more associations with pyrodiversity than other taxa. Correlations shown by bats often had opposite sign to those of birds providing limited support for our expectation that bats may be more like birds than other mammals (Table 2). The overall pattern was for a few fire attributes to show consistent patterns across all groups with variation between taxonomic groups limited to those fire attributes with limited overall support. Thus, the strong patterns were well supported across taxonomic groups, with only minor differences between groups.

### Inside and outside protected areas

We found no difference in overall pyrodiversity inside and outside savanna protected areas within 100 km of the boundaries (β_*pyrodiversity*_ = 0.098 ± 0.170; mean ± standard error). However, there were differences in individual pyrodiversity attributes. Relative to fires outside protected areas, fires inside were earlier in the fire season (β_*fireday*_ = − 30.514 ± 12.112), more frequent (β_*log(FRI)*_ **= − 0.338 ± 0.097) and of higher intensity (β_*log(FRP)*_ **= 0.239 ± 0.098). Variability in fire size (β_*cv‐log(area)*_ **= 0.021 ± 0.004) and seasonality (β_*cv‐fireday*_ = 0.087 ± 0.040) both increased inside of protected areas (see Supporting Information S1 for full details).

## Discussion

We found strong support for our primary hypothesis: once well‐known effects of primary productivity and topography are accounted for, there is a consistent positive effect of pyrodiversity on richness of both mammals and birds in protected areas across African savannas. This effect was notably stronger in wet (>650 mm year^−1^) than dry savannas, and results in increases in richness of 27–40% in the wettest savannas. While low pyrodiversity sites in wet savannas could have either high or low richness, high pyrodiversity was consistently associated with high richness, suggesting high pyrodiversity may be sufficient but is not necessary for richness.

The importance of fire regimes within the savanna biome has long been recognised, both as a determinant of savanna distribution through maintenance of savanna‐forest boundaries (Hoffmann *et al*. 2012) and for influencing vegetation structure and biodiversity (Higgins *et al*. 2007; Smit *et al*. 2010). However, the effects of fire regime variability (pyrodiversity) have been far more ambiguous: theoretical work suggests there should be a positive impact (e.g., Martin & Sapsis 1992), yet empirical work has previously yielded inconsistent results even within the savanna biome (e.g. Parr *et al*. 2004; Parr & Andersen 2006; Taylor *et al*. 2012; Maravalhas & Vasconcelos 2014). To our knowledge, this is the first study to show consistent positive effects of pyrodiversity in savannas at a continental scale across multiple taxonomic groups and, importantly, to demonstrate that the strength of this relationship depends on rainfall. This finding is remarkable given the continental scale and relatively crude distribution data that are available.

Our results provide a possible explanation for why previous pyrodiversity‐biodiversity studies in savannas have found variable support for expected effects. First, richness responses vary nonlinearly with rainfall and are subtly different between taxa. Previous studies in savannas have been conducted at smaller scales, rarely spanning a wide range of environmental conditions, and thus gaining only a limited understanding of how biodiversity responds to fire. Our results suggest that studies limited to drier savannas (<650 mm year^−1^) are unlikely to find strong pyrodiversity effects, whereas those in wet savannas could show diverse effects across different taxonomic groups. We focused on the response of birds and mammals to pyrodiversity, but predict that other taxa, particularly those with limited dispersal ability, may have weaker responses. Second, we found that the relationship between pyrodiversity and richness is not the sum of the variability in individual fire attributes, but is a complicated, multi‐dimensional process. Partly for logistical reasons, empirical studies have often focused on variation in just one or two aspects of the fire regime (e.g. fire return interval, Davies *et al*. 2012; seasonality, Parr *et al*. 2004; fire severity, Tingley *et al*. 2016), our study suggests that single variables do not capture the complexity of the role of fire in enhancing biodiversity.

Our finding that pyrodiversity is a stronger predictor of richness in wet savannas is consistent with field studies that have documented biodiversity‐pyrodiversity responses in wetter savannas (e.g., Parr *et al*. 2004; Davies *et al*. 2012; Maravalhas & Vasconcelos 2014). It supports the idea that the effects of pyrodiversity operate largely indirectly through the impact of fire on vegetation structure. Fauna often respond strongly to vegetation structure (e.g. MacArthur & MacArthur 1961), fire has a greater potential effect on vegetation structure (e.g. height and complexity) and tree cover in wetter savannas (e.g. Higgins *et al*. 2007; Bond 2008). In wet savannas, diversity in the most pyrodiverse sites may actually be higher than estimated here, as burning that generates a savanna forest mosaic will permit forest species to colonise the landscape, changes not considered here. Our finding that high pyrodiversity in wet savannas encouraged restricted range bird species, whereas low pyrodiversity sites are dominated by widespread species, suggests that pyrodiversity generates a diversity of rare habitats within wet savannas. That we did not find such a mechanism for mammals suggests the ecological mechanisms driving niche diversity may differ between birds and mammals.

Although our primary hypothesis was well supported, we found equivocal support for differences between taxa. Given the reported sensitivity of small mammals to fire (Andersen *et al*. 2012), we expected that smaller, less mobile animals would be most sensitive to pyrodiversity. Instead, we found consistent patterns across taxa in wet savannas, but weaker and variable effects in dry ones. We observed clearer effects in the full assemblages of birds and mammals than subset groupings. These effects could result from sampling: when binary presence‐absence data are used, the assemblages containing more species also contain greater information (vary more from highest to lowest richness), leading to greater statistical power. With low statistical power, fire attributes of marginal importance may be supported by chance alone in models for some groups but not in others, such stochastic variation is unlikely for attributes with strong effects. Despite statistical effects, the general similarity between taxon groups is surprising and suggests that direct effects of fire on mortality or emigration are minimal. Thus, the main effects of fire on biodiversity must be mediated through altered vegetation structure (Hempson *et al*. 2017).

In addition to the novelty of our pyrodiversity results, our results for individual fire attributes also provide new information. Despite common assumptions, few studies have explored the effects of multiple aspects of fire regimes on biodiversity. Several studies have focused on time since fire and found little support for the pyrodiversity‐biodiversity hypothesis (e.g. Taylor *et al*. 2012). Small mammal declines in the savannas of northern Australia have been linked to large, high‐frequency fires (Andersen *et al*. 2012; Lawes *et al*. 2015) countering our results on frequency but supporting our result that large fires generally reduce richness. Studies on FRI are scarce, but findings for ants and termites from South Africa suggest no clear relationship (Parr *et al*. 2004; Davies *et al*. 2012), in part this may be due to experiments only being carried out in dry ecosystems. In Africa, there have been fewer studies of the effects of fire on biodiversity in wet than in dry savannas.

That high variation in fire season might have benefits on richness in wet savanna makes intuitive sense, despite limited empirical data confirming this. The complementary insight that fire seasonal variation within dry savannas may reduce richness has not previously been considered, but such differences between wet and dry savannas are reasonable. Changes in the seasonality of fires affect both forage availability and fire intensity. Early fires are cooler and patchier, whereas late fires create more complete burns and are often more intense (Williams *et al*. 1998). In wet savannas variability in fire season would increase variability in both woody and grass structure, thereby increasing habitat heterogeneity. Burned sites in wet areas flush new growth within days, maintaining some forage (grass and seeds) throughout the season. However, in dry savannas there is often insufficient soil moisture for a post‐fire flush, and the impact of early or late‐season fires on food availability might have a more negative effect than the positive impact of generating slight structural variation. Consequently, in wet savannas fires in both early and late seasons could alter vegetation structure and hence richness, but similar structural effects are unlikely in dry savanna where direct effects of reduced forage may be magnified; optimal management in such regions would involve a consistent pattern of late season fires.

If pyrodiversity can be manipulated, our study provides clear management recommendations. Comparisons of pyrodiversity inside and outside protected areas showed no overall effect of protection on pyrodiversity. Yet, individual pyrodiversity components differed across protected area boundaries, implying management can alter components of pyrodiversity in contrasting ways that together generate similar levels of pyrodiversity (see Hempson *et al*. 2017). Our results indicate that in wet savannas, managing directly for pyrodiversity could be beneficial through increasing variation in seasonality and area burnt. Current debates on when to burn in Africa focus on greenhouse gas emissions but the impacts on biodiversity should also be considered: restricting pyrodiversity could result in biodiversity loss. We show here that in dry savannas biodiversity may be higher where fires are later. We caution that our results are correlative, nonetheless we recommend trial management of varied season and frequency which should maintain high diversity. Such manipulations aimed at increasing pyrodiversity should be maintained within the range of naturally occurring variation to which local communities may be adapted. Finally, we emphasise these findings are based on savannas in Africa. More work is needed to determine whether similar results can be expected in savannas and other flammable biomes elsewhere. We are confident that our new pyrodiversity metric and approach will facilitate advances across other systems.

## Authorship

All authors designed the study, prepared data sets and wrote the manuscript; CB led the analysis.

## Data Accessibility Statement

All the richness patterns analysed in this paper are available from Figshare: DOI: 10.6084/m9.figshare.5809524. Pyrodivresity data are available from Dryad: http://dx.doi.org/10.5061/dryad.26sb5.
