# Supplementary material for: Pyrodiversity interacts with rainfall to increase bird and mammal richness in African savannas
Source: Ecol Lett. 2018 Feb 14;21(4):557–67. doi: 10.1111/ele.12921 (PMC5888149; doi:10.1111/ele.12921)
Supplement: Supplementary file 1 [file ELE-21-557-s001.pdf]

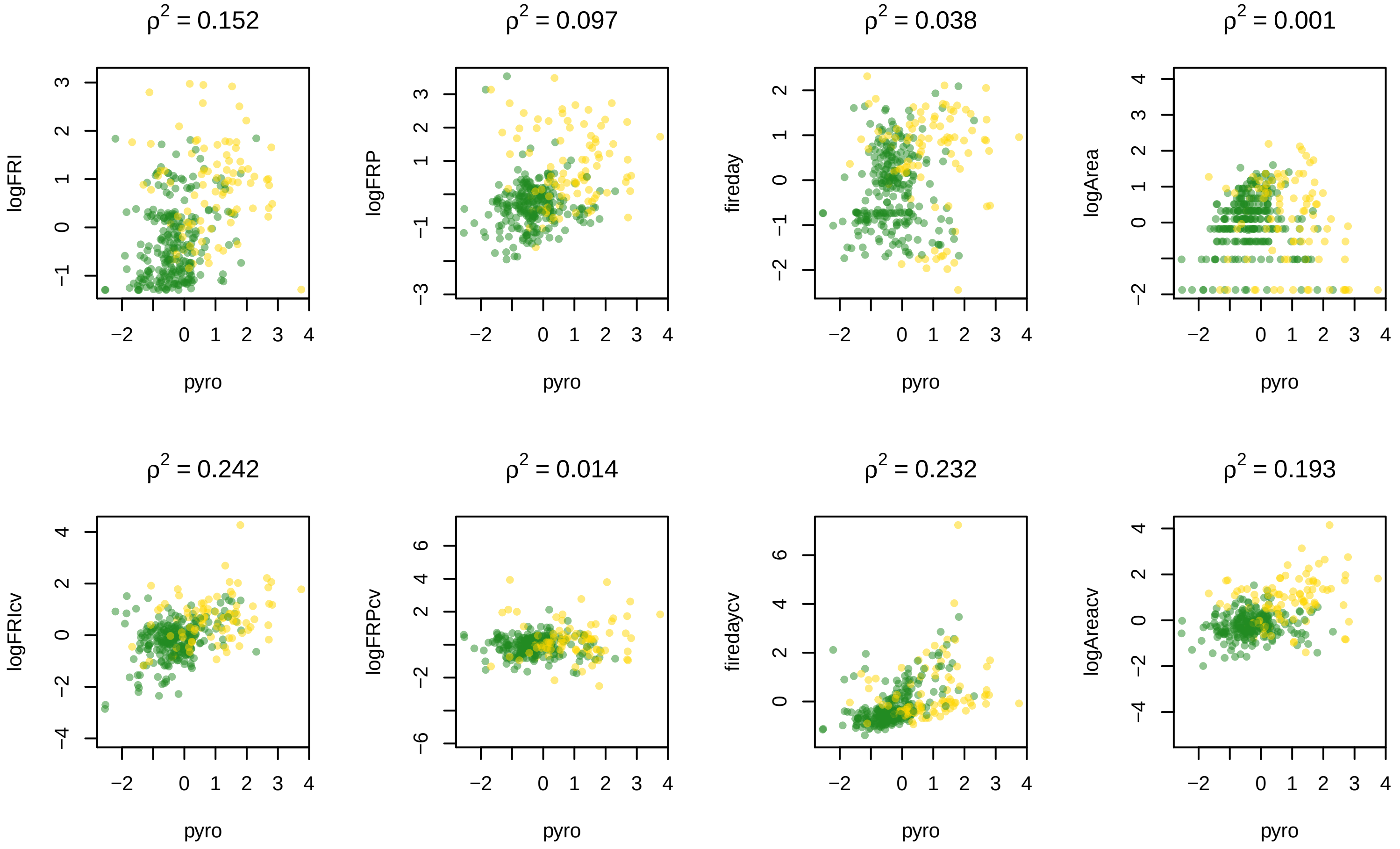

ρ² = 0.152
logFRI
pyro
ρ² = 0.097
logFRP
pyro
ρ² = 0.038
fireday
pyro
ρ² = 0.001
logArea
pyro
ρ² = 0.242
logFRIcv
pyro
ρ² = 0.014
logFRPcv
pyro
ρ² = 0.232
firedaycv
pyro
ρ² = 0.193
logAreacv
pyro

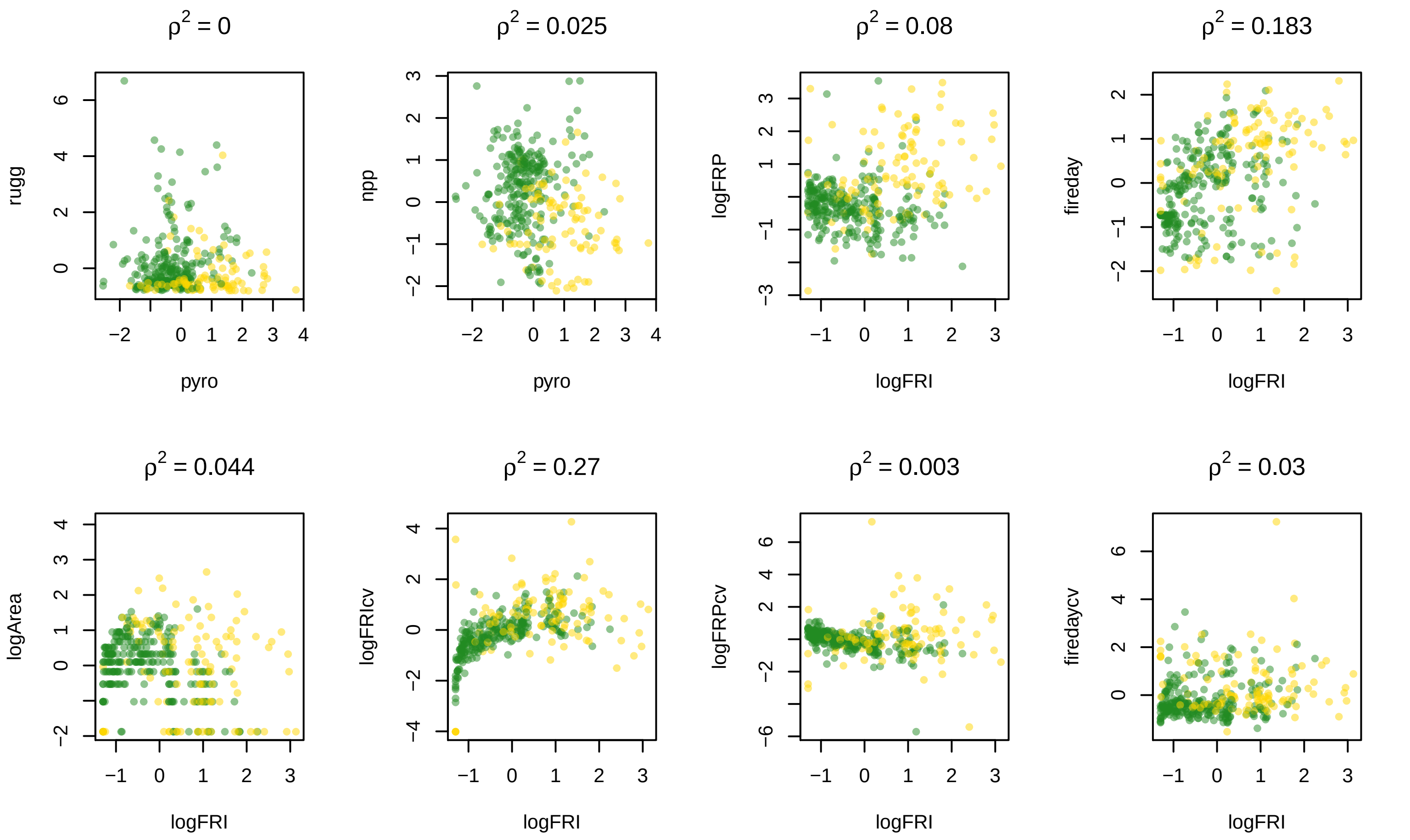

$\rho^2 = 0$
rugg
pyro
$\rho^2 = 0.025$
npp
pyro
$\rho^2 = 0.08$
logFRP
logFRI
$\rho^2 = 0.183$
fireday
logFRI
$\rho^2 = 0.044$
logArea
logFRI
$\rho^2 = 0.27$
logFRIcv
logFRI
$\rho^2 = 0.003$
logFRPcv
logFRI
$\rho^2 = 0.03$
firedaycv
logFRI

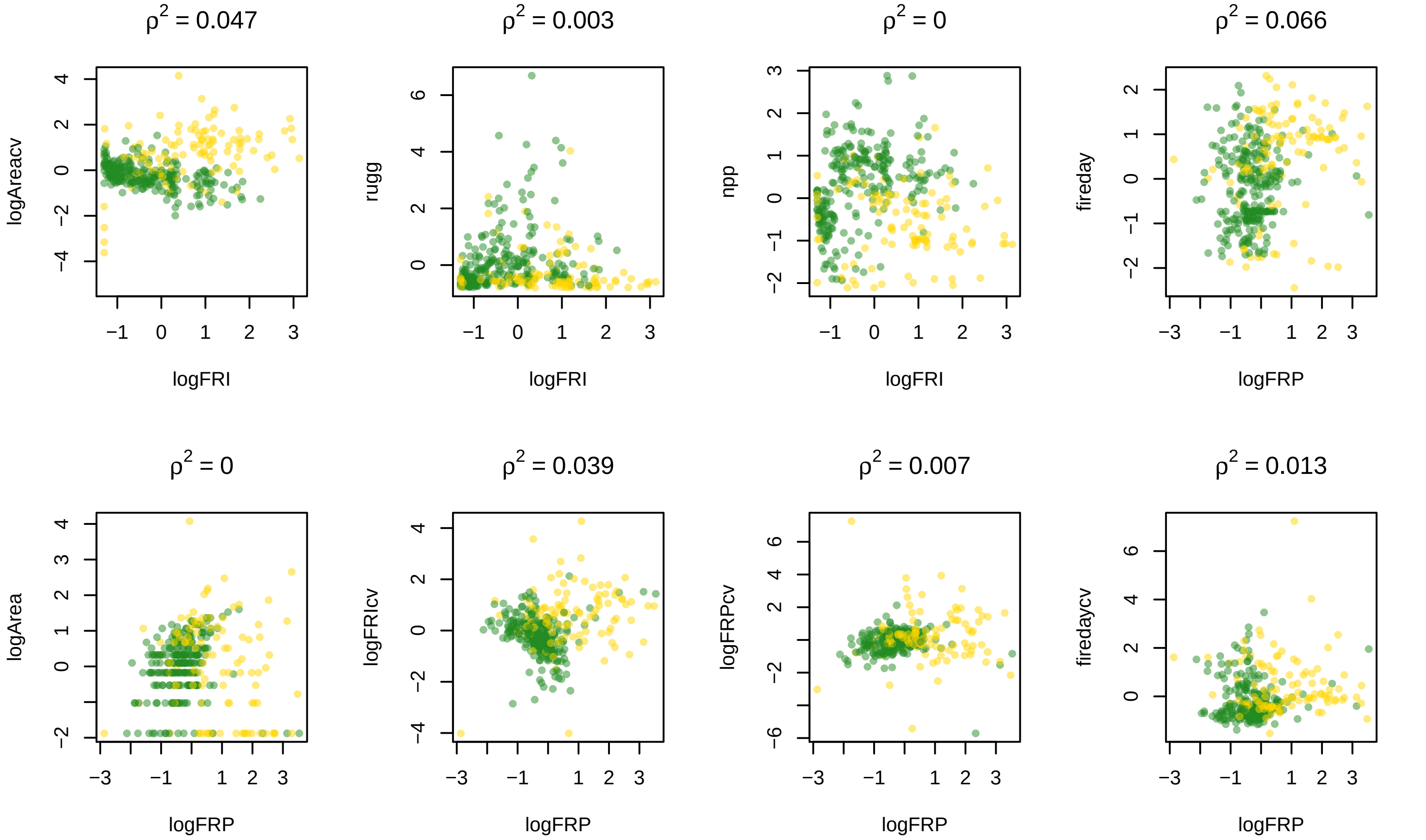

$\rho^2 = 0.047$
logAreacv
logFRI
$\rho^2 = 0.003$
rugg
logFRI
$\rho^2 = 0$
npp
logFRI
$\rho^2 = 0.066$
fireday
logFRP
$\rho^2 = 0$
logArea
logFRP
$\rho^2 = 0.039$
logFRIcv
logFRP
$\rho^2 = 0.007$
logFRPcv
logFRP
$\rho^2 = 0.013$
firedaycv
logFRP

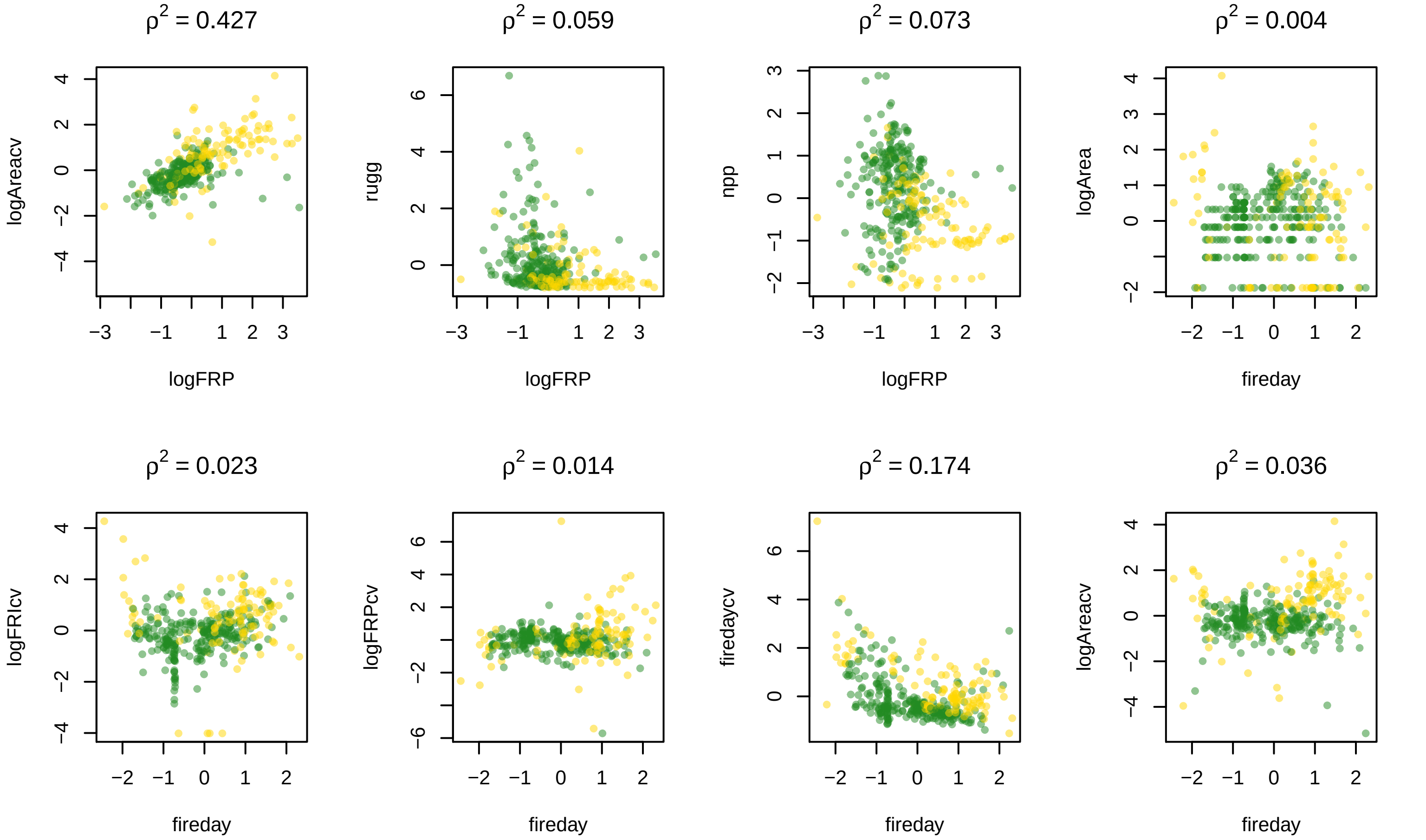

ρ2 = 0.427
logAreacv
logFRP
ρ2 = 0.059
rugg
logFRP
ρ2 = 0.073
npp
logFRP
ρ2 = 0.004
logArea
fireday
ρ2 = 0.023
logFRIcv
fireday
ρ2 = 0.014
logFRPcv
fireday
ρ2 = 0.174
firedaycv
fireday
ρ2 = 0.036
logAreacv
fireday

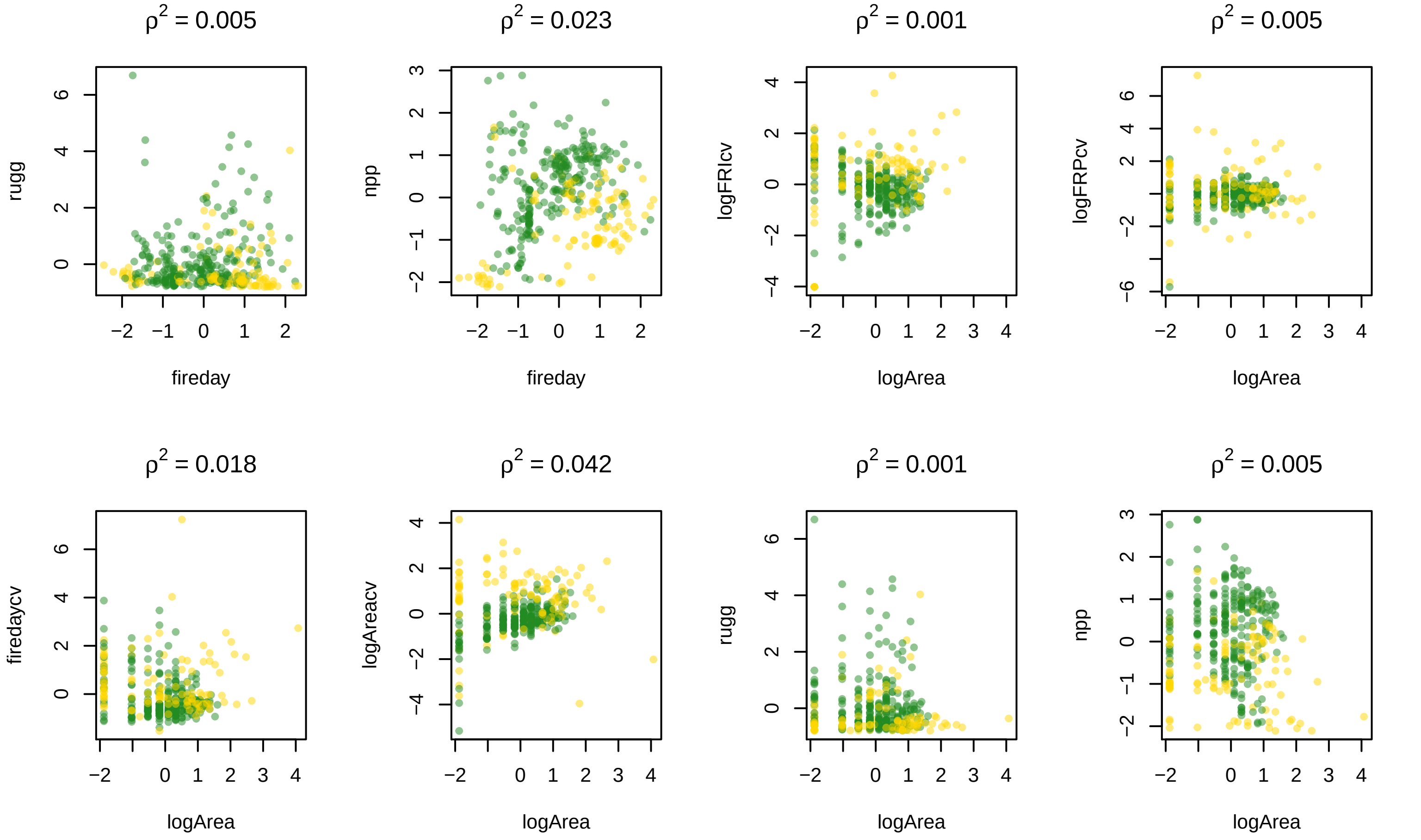

$\rho^2 = 0.005$
rugg
fireday
$\rho^2 = 0.023$
npp
fireday
$\rho^2 = 0.001$
logFRIcv
logArea
$\rho^2 = 0.005$
logFRPcv
logArea
$\rho^2 = 0.018$
firedaycv
logArea
$\rho^2 = 0.042$
logAreacv
logArea
$\rho^2 = 0.001$
rugg
logArea
$\rho^2 = 0.005$
npp
logArea

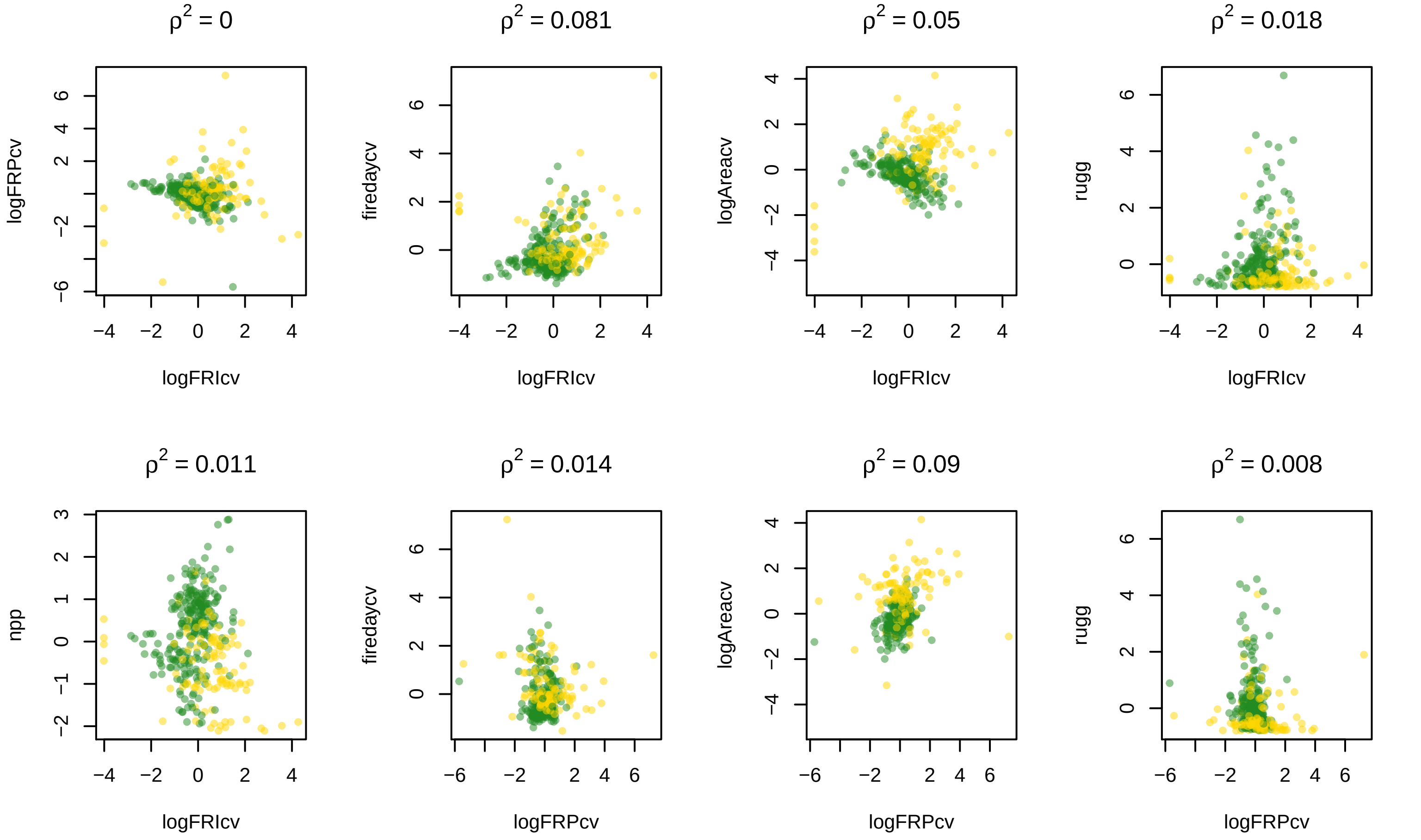

ρ² = 0
logFRPcv
logFRIcv
ρ² = 0.081
firedaycv
logFRIcv
ρ² = 0.05
logAreacv
logFRIcv
ρ² = 0.018
rugg
logFRIcv
ρ² = 0.011
npp
logFRIcv
ρ² = 0.014
firedaycv
logFRPcv
ρ² = 0.09
logAreacv
logFRPcv
ρ² = 0.008
rugg
logFRPcv

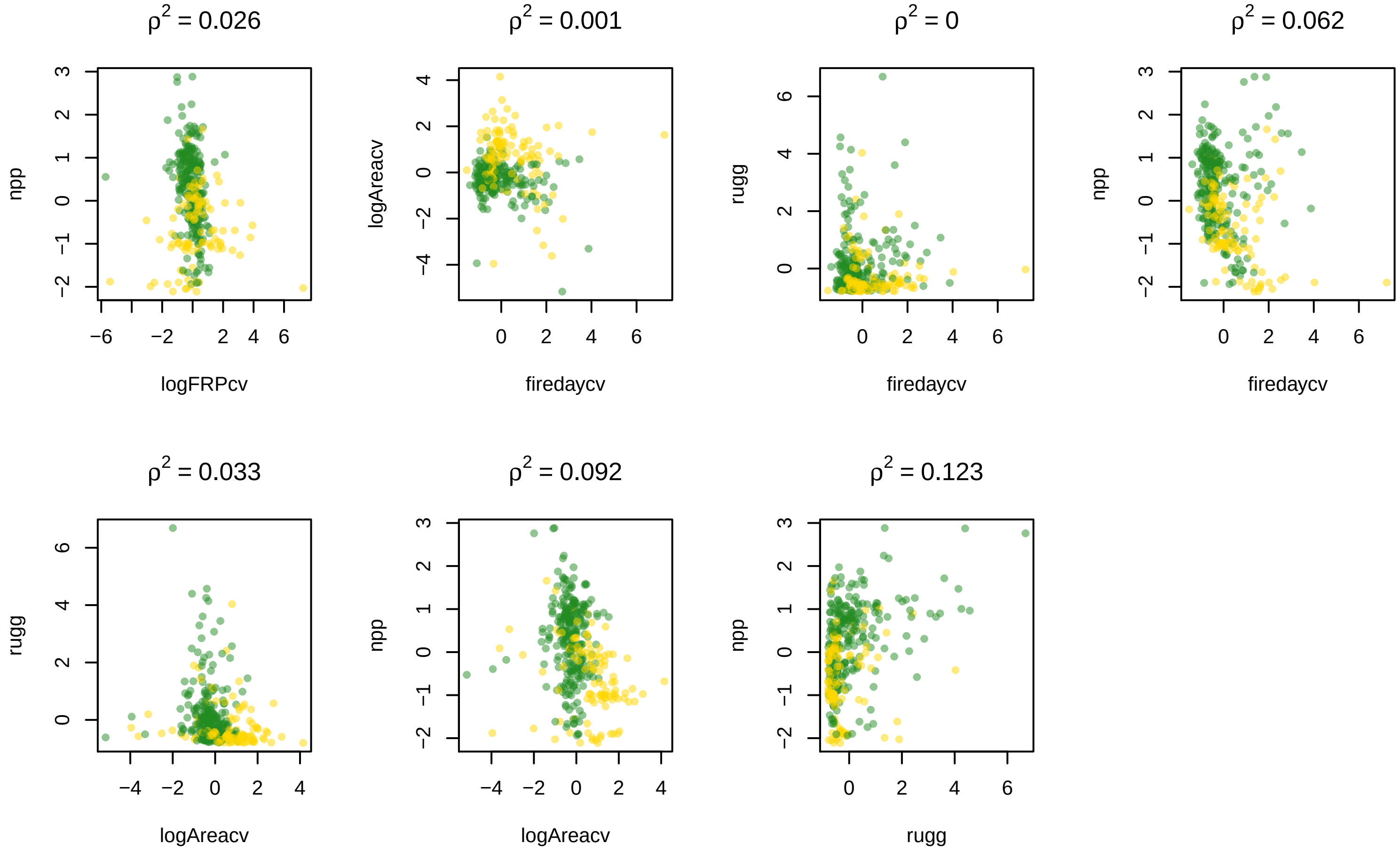
ρ² = 0.026
npp
logFRPcv
ρ² = 0.001
logAreacv
firedaycv
ρ² = 0
rugg
firedaycv
ρ² = 0.062
npp
firedaycv
ρ² = 0.033
rugg
logAreacv
ρ² = 0.092
npp
logAreacv
ρ² = 0.123
npp
rugg
