# Supplementary material for: Pyrodiversity interacts with rainfall to increase bird and mammal richness in African savannas
Source: Ecol Lett. 2018 Feb 14;21(4):557–67. doi: 10.1111/ele.12921 (PMC5888149; doi:10.1111/ele.12921)
Supplement: Supplementary file 2 [file ELE-21-557-s002.pdf]

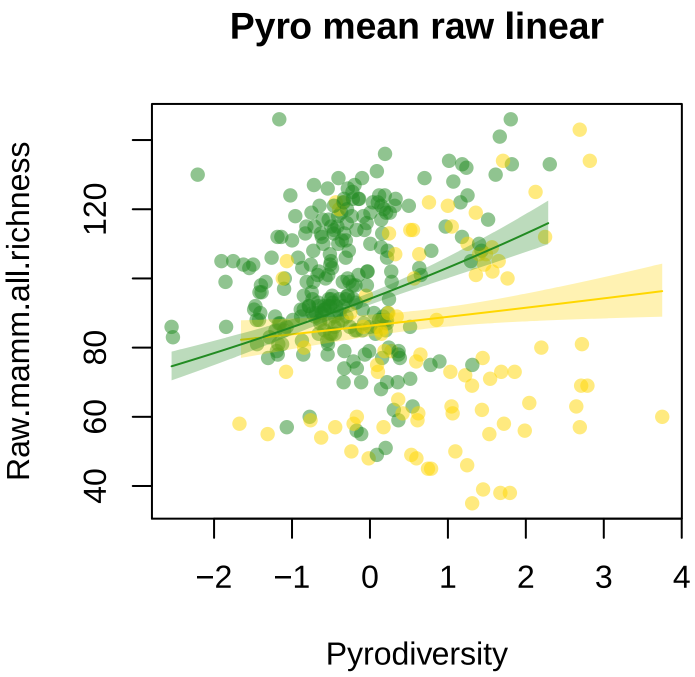

Pyro mean raw linear
Raw.mamm.all.richness
40
60
80
120
−2
−1
0
1
2
3
4
Pyrodiversity

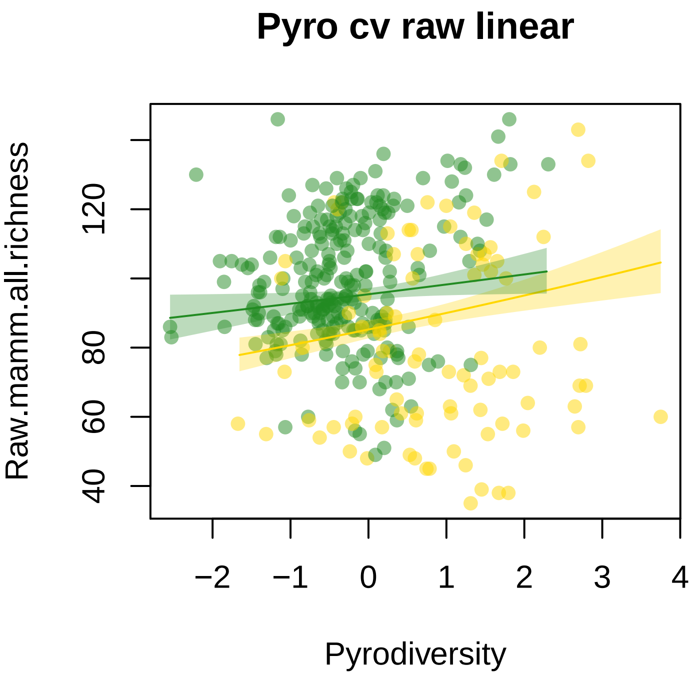

Pyro cv raw linear
Raw.mamm.all.richness
40
60
80
120
−2
−1
0
1
2
3
4
Pyrodiversity

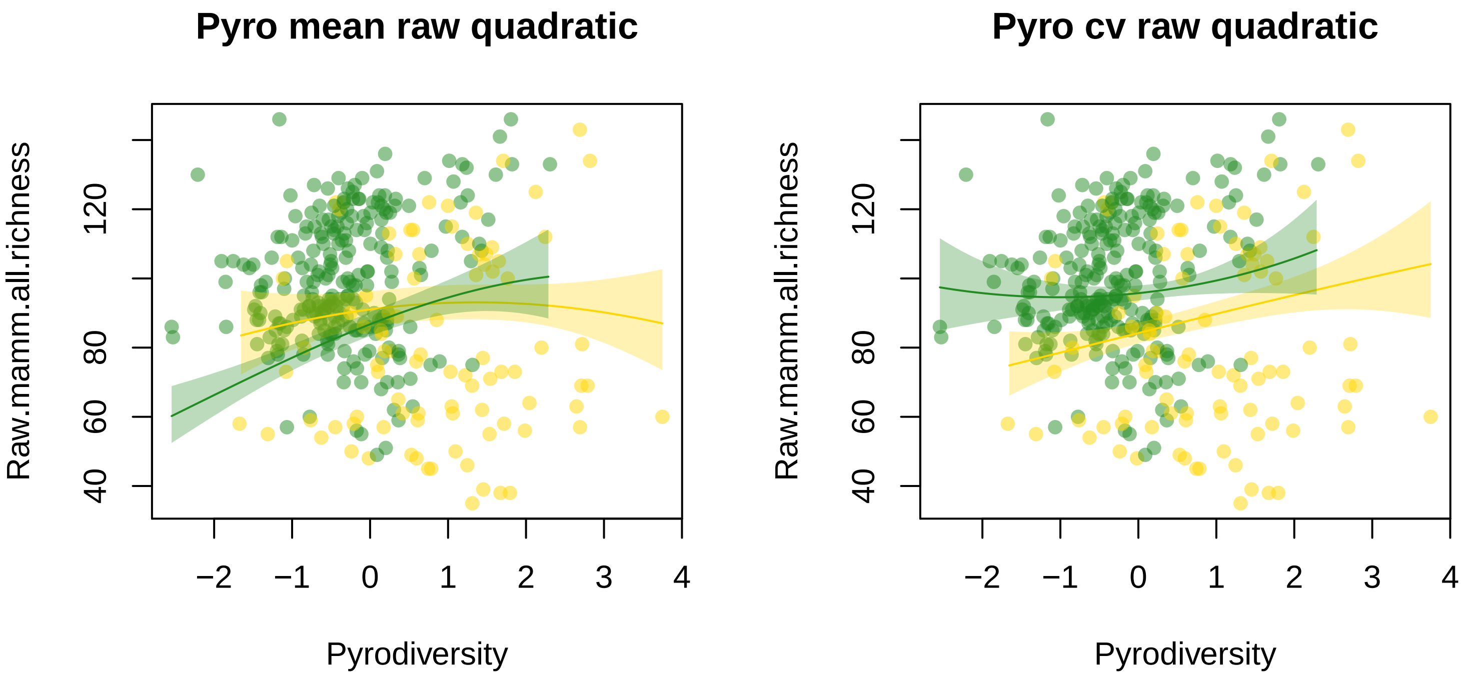

Pyro mean raw quadratic
Pyro cv raw quadratic
Raw.mamm.all.richness
40
60
80
120
−2
−1
0
1
2
3
4
Pyrodiversity

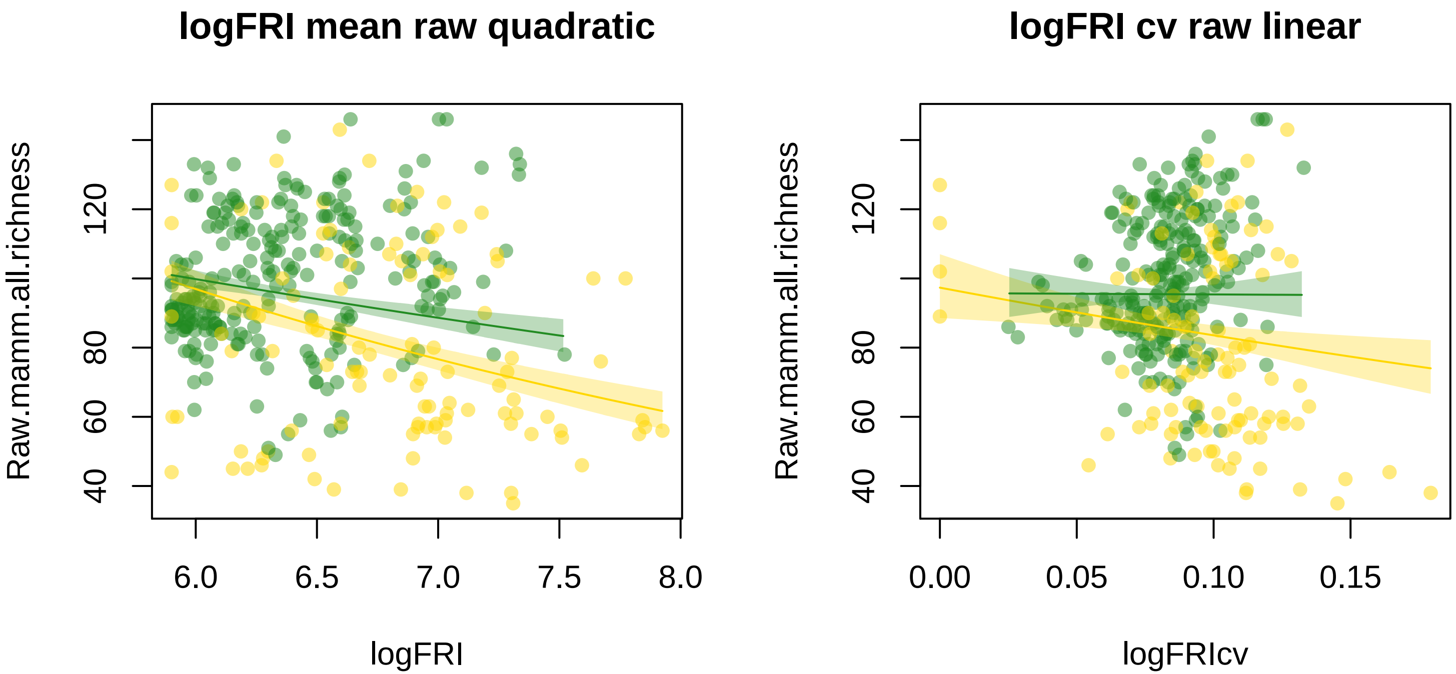
logFRI mean raw quadratic
Raw.mamm.all.richness
40
60
80
120
6.0
6.5
7.0
7.5
8.0
logFRI
logFRI cv raw linear
Raw.mamm.all.richness
40
60
80
120
0.00
0.05
0.10
0.15
logFRIcv

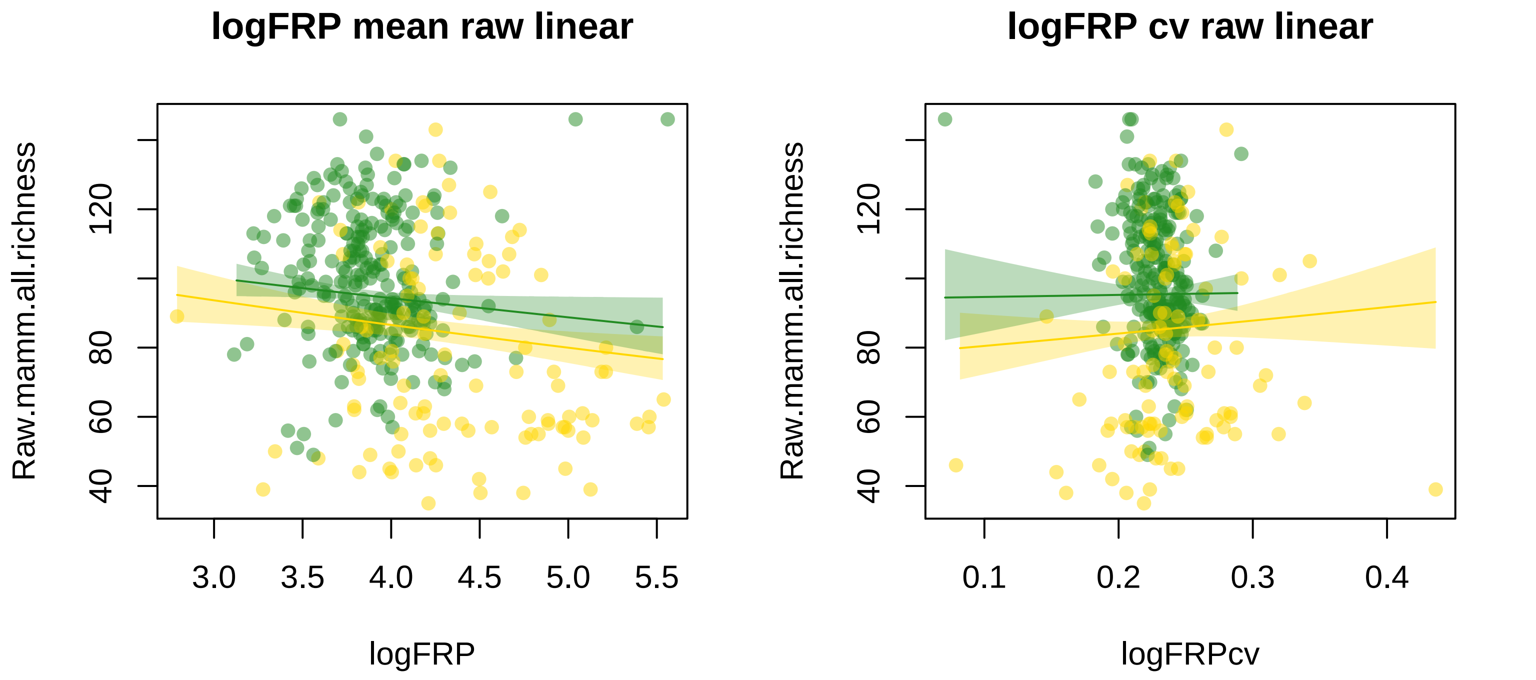
logFRP mean raw linear
logFRP cv raw linear
Raw.mamm.all.richness
40
60
80
120
3.0
3.5
4.0
4.5
5.0
5.5
logFRP
0.1
0.2
0.3
0.4
logFRPcv

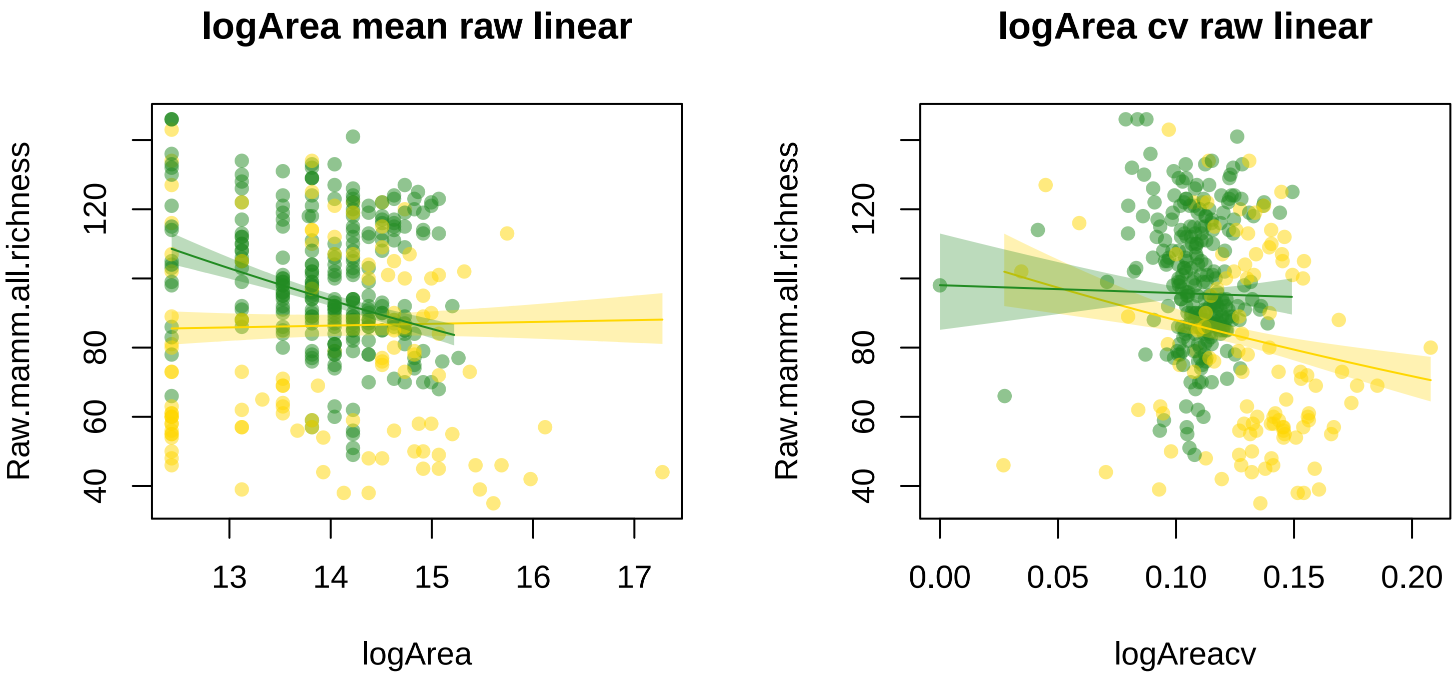
logArea mean raw linear
Raw.mamm.all.richness
40
60
80
120
13
14
15
16
17
logArea
logArea cv raw linear
Raw.mamm.all.richness
40
60
80
120
0.00
0.05
0.10
0.15
0.20
logAreacv

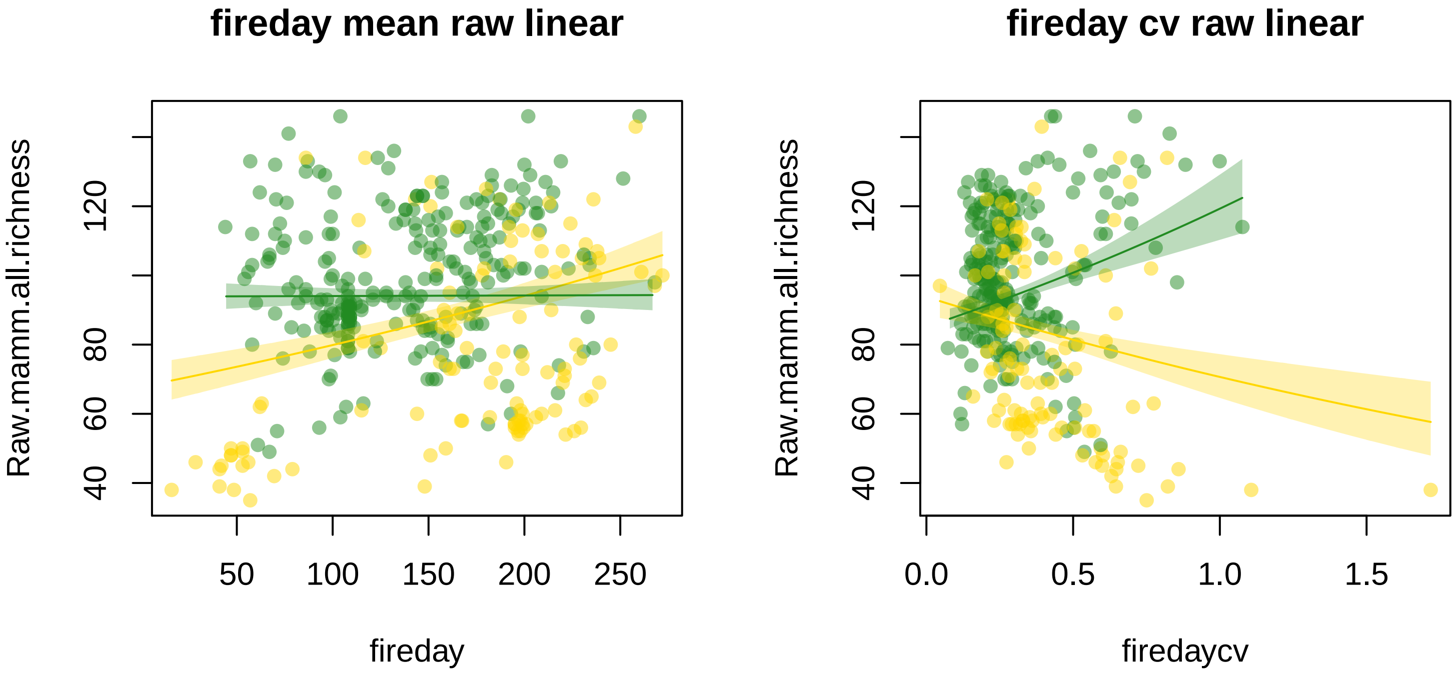
fireday mean raw linear
Raw.mamm.all.richness
40
60
80
120
50
100
150
200
250
fireday
fireday cv raw linear
Raw.mamm.all.richness
40
60
80
120
0.0
0.5
1.0
1.5
firedaycv

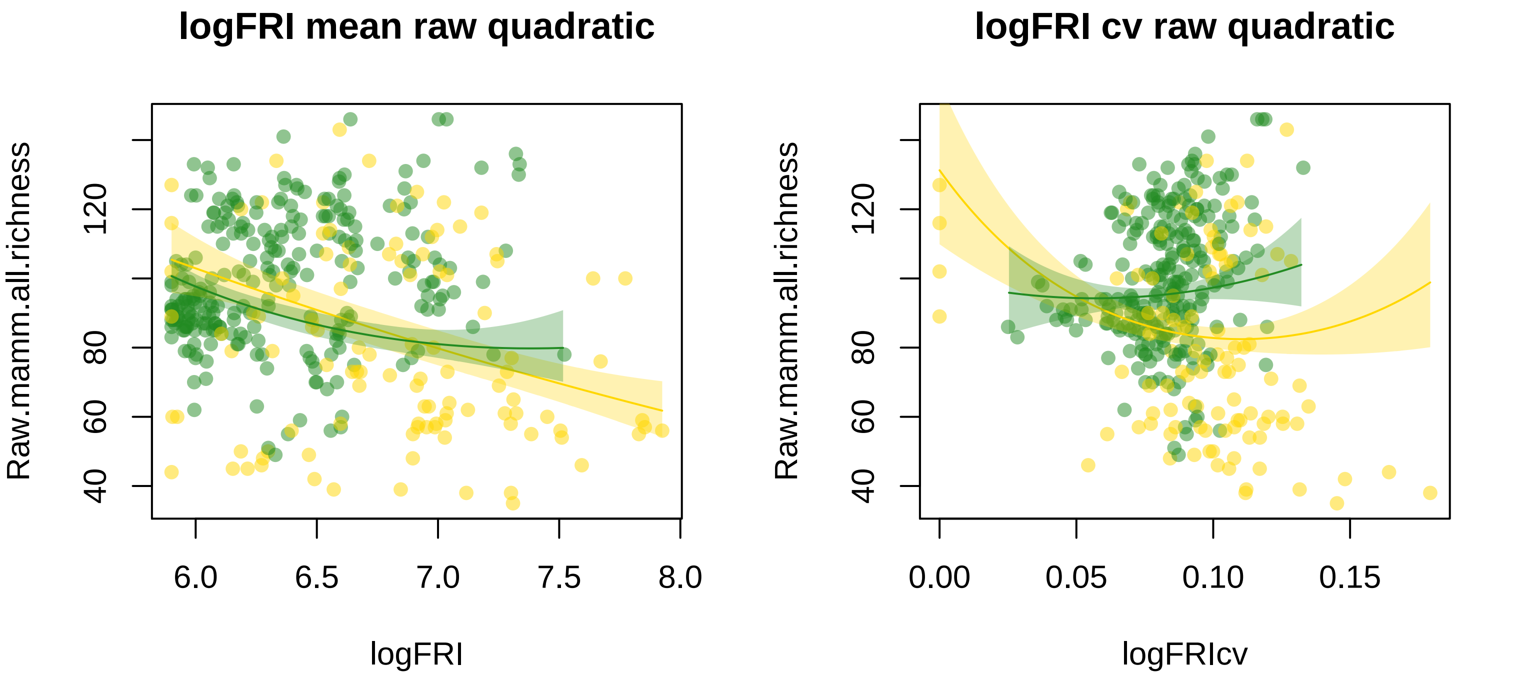

logFRI mean raw quadratic
Raw.mamm.all.richness
40
60
80
120
6.0
6.5
7.0
7.5
8.0
logFRI
logFRI cv raw quadratic
Raw.mamm.all.richness
40
60
80
120
0.00
0.05
0.10
0.15
logFRIcv

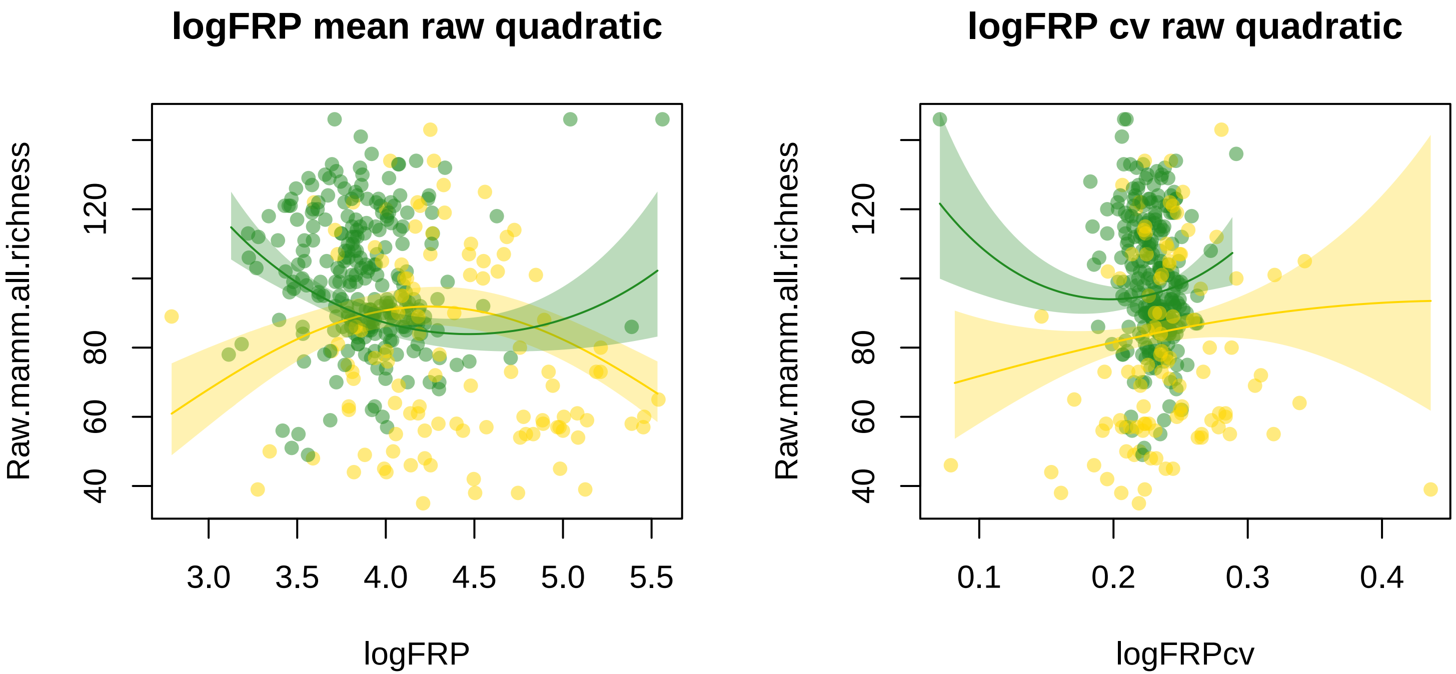
logFRP mean raw quadratic
logFRP cv raw quadratic
Raw.mamm.all.richness
40
60
80
120
3.0
3.5
4.0
4.5
5.0
5.5
logFRP
0.1
0.2
0.3
0.4
logFRPcv

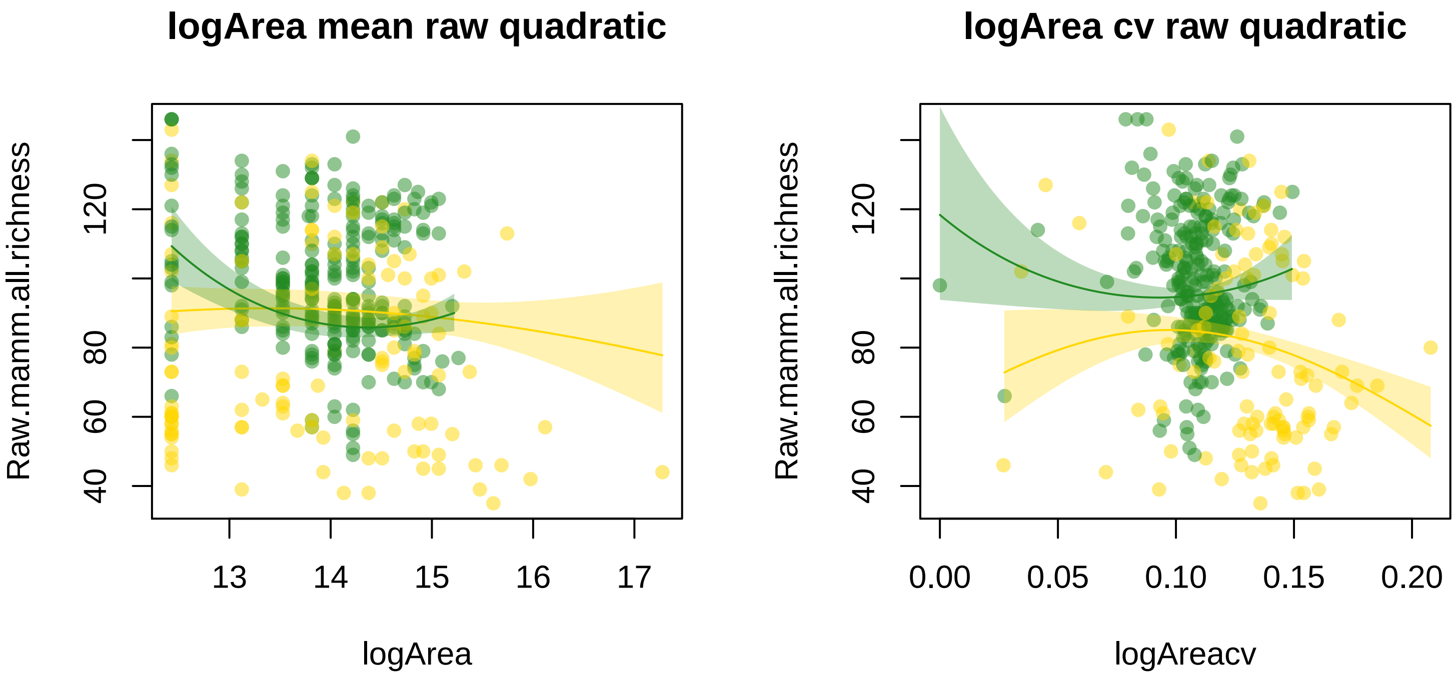

logArea mean raw quadratic
Raw.mamm.all.richness
40
60
80
120
13
14
15
16
17
logArea
logArea cv raw quadratic
Raw.mamm.all.richness
40
60
80
120
0.00
0.05
0.10
0.15
0.20
logAreacv

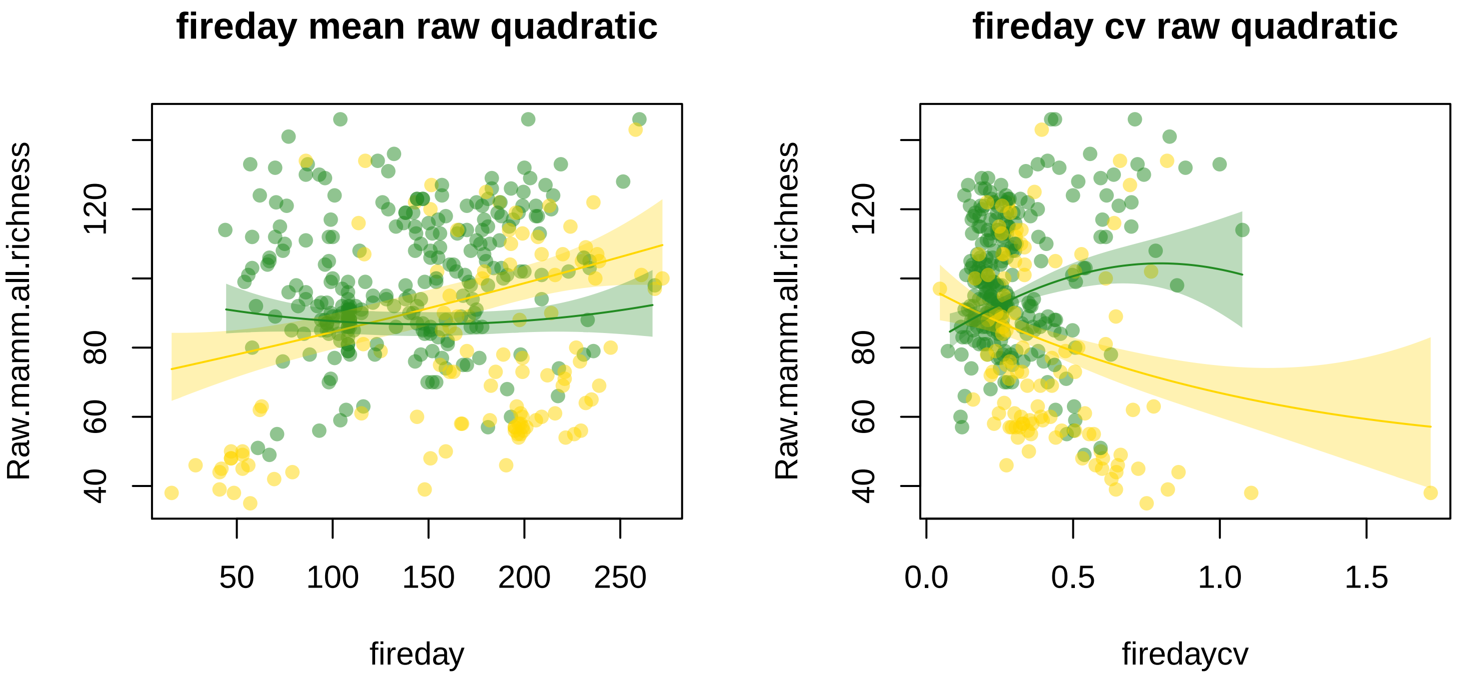
fireday mean raw quadratic
Raw.mamm.all.richness
40
60
80
120
50
100
150
200
250
fireday
fireday cv raw quadratic
Raw.mamm.all.richness
40
60
80
120
0.0
0.5
1.0
1.5
firedaycv

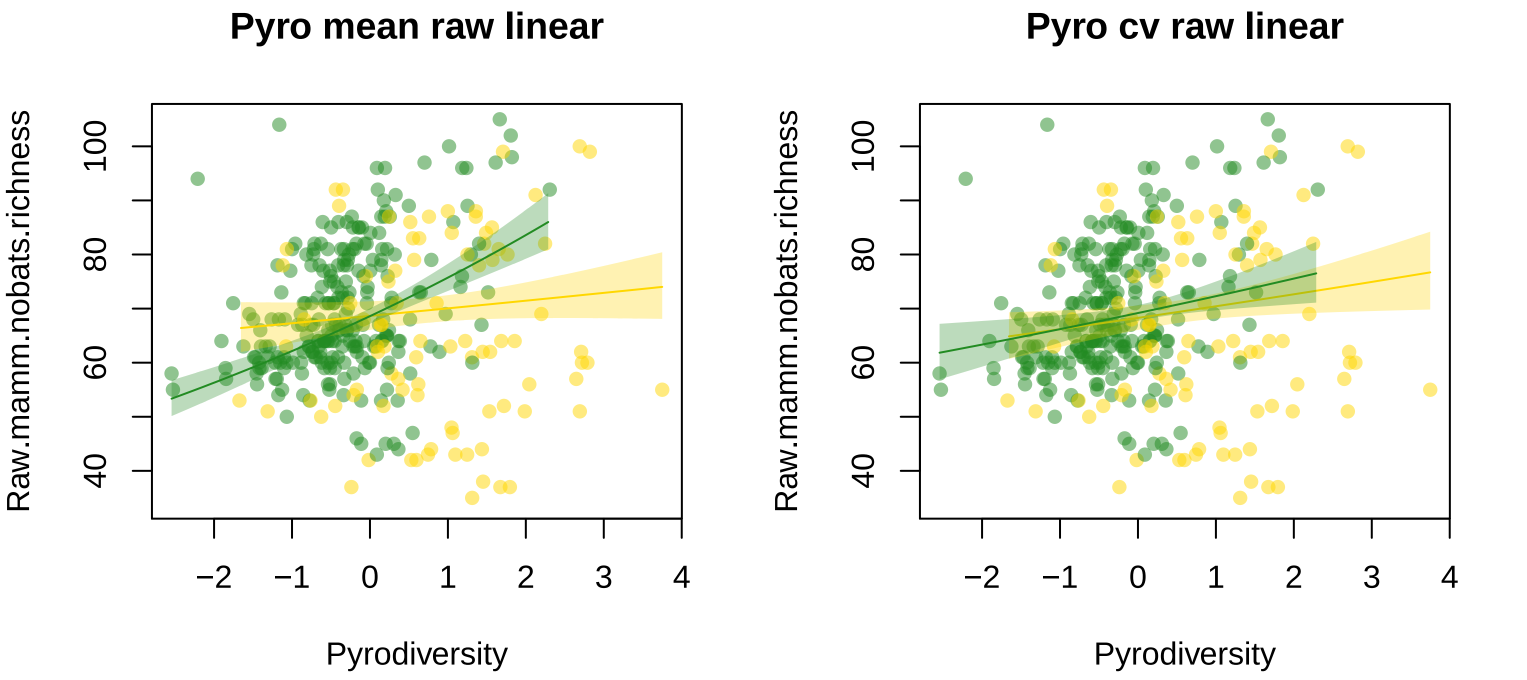

Pyro mean raw linear
Pyro cv raw linear
Raw.mamm.nobats.richness
40
60
80
100
−2
−1
0
1
2
3
4
Pyrodiversity

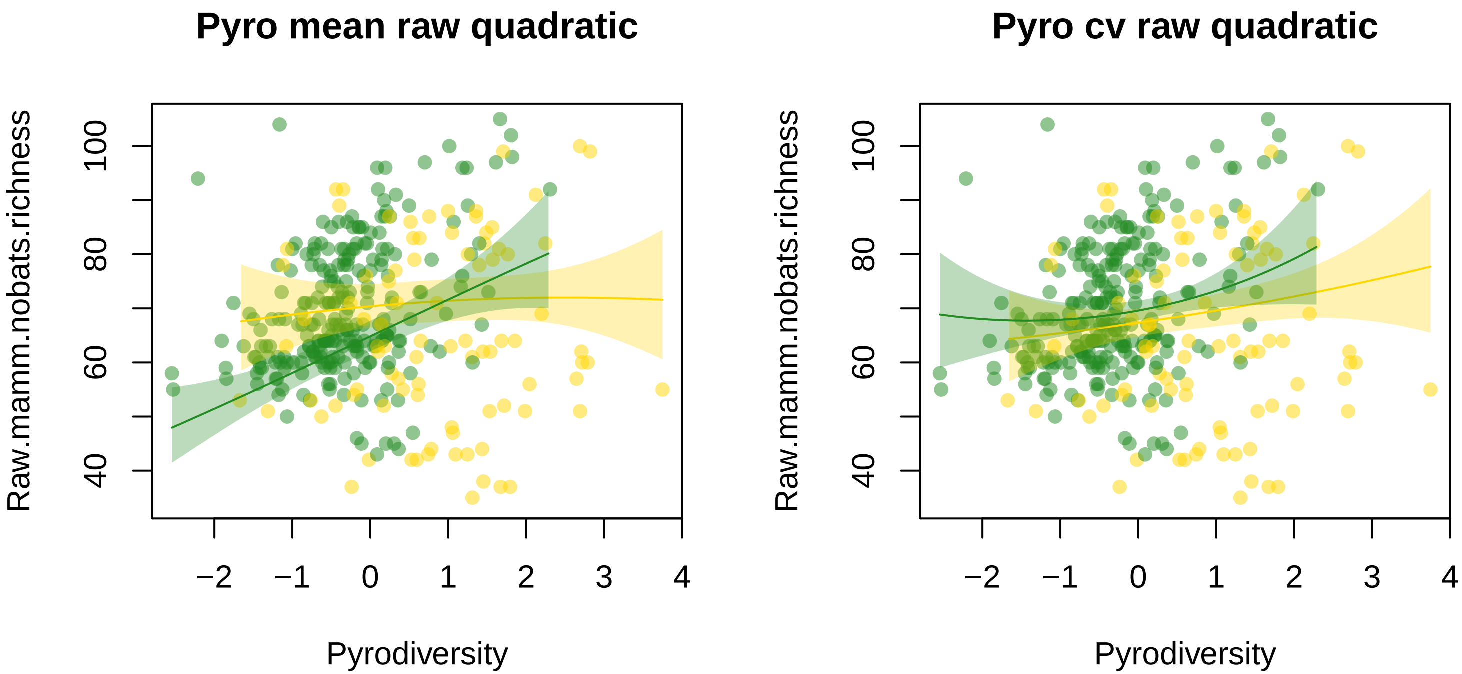
Pyro mean raw quadratic
Pyro cv raw quadratic
Raw.mamm.nobats.richness
Pyrodiversity
100
80
60
40
−2
−1
0
1
2
3
4

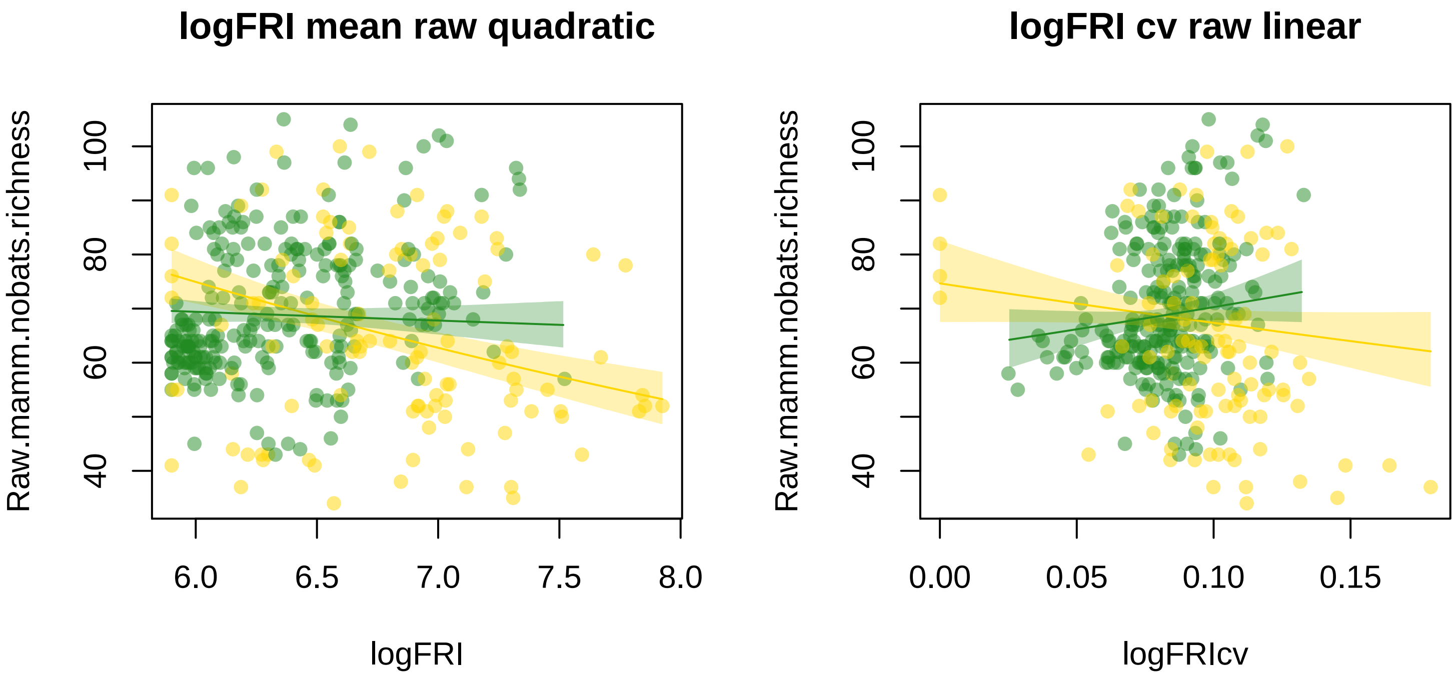

logFRI mean raw quadratic
logFRI cv raw linear
Raw.mamm.nobats.richness
40
60
80
100
6.0
6.5
7.0
7.5
8.0
logFRI
0.00
0.05
0.10
0.15
logFRIcv

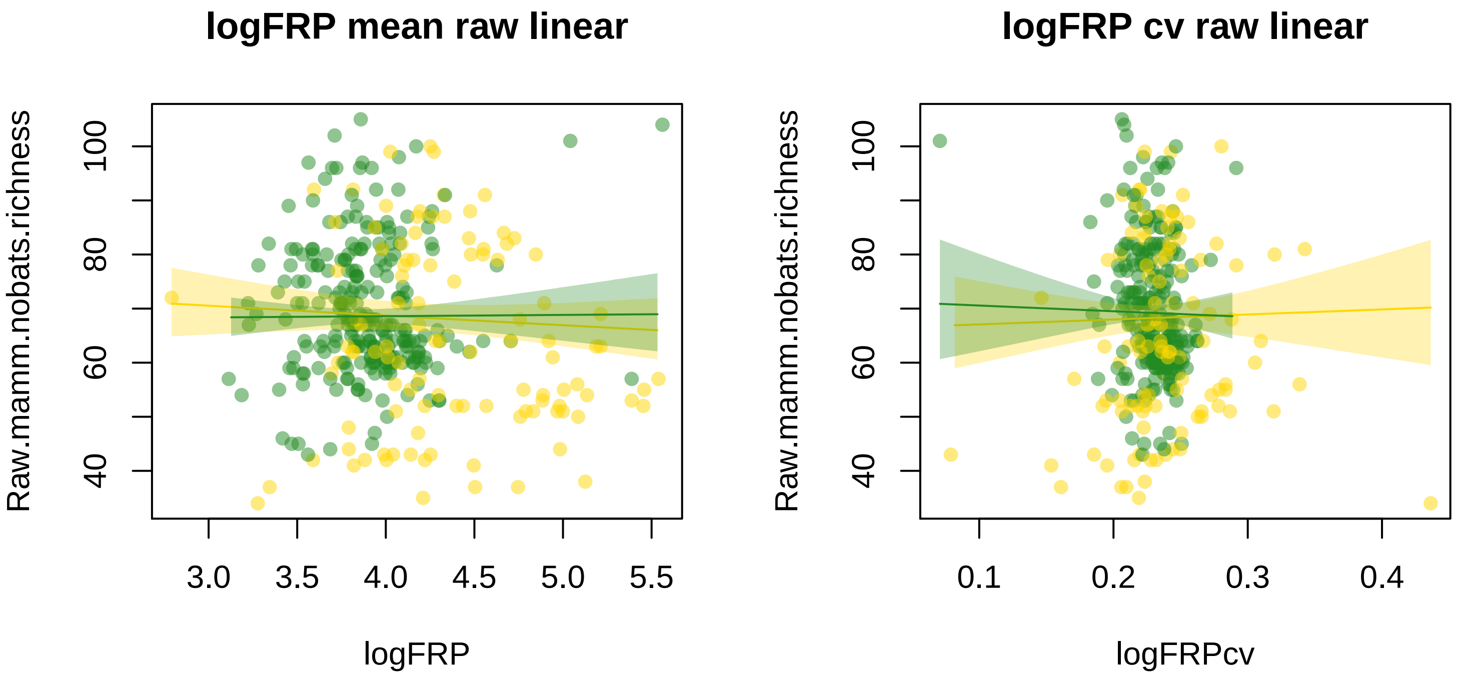
logFRP mean raw linear
logFRP cv raw linear
Raw.mamm.nobats.richness
40
60
80
100
3.0
3.5
4.0
4.5
5.0
5.5
logFRP
0.1
0.2
0.3
0.4
logFRPcv

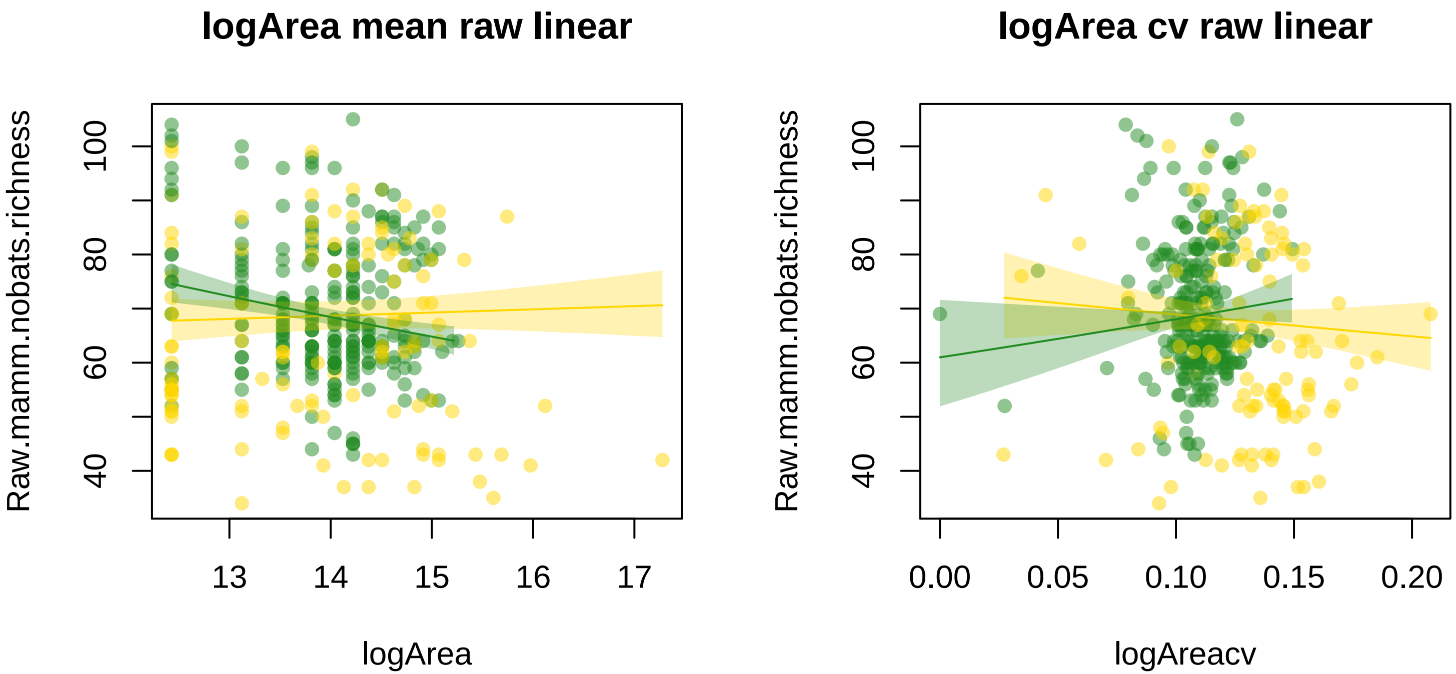

logArea mean raw linear
Raw.mamm.nobats.richness
40
60
80
100
13
14
15
16
17
logArea
logArea cv raw linear
Raw.mamm.nobats.richness
40
60
80
100
0.00
0.05
0.10
0.15
0.20
logAreacv

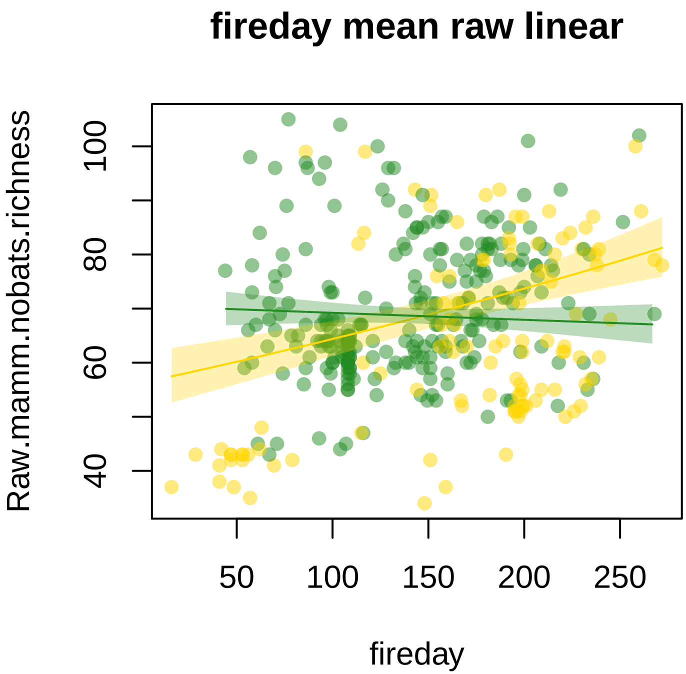
fireday mean raw linear
Raw.mamm.nobats.richness
40
60
80
100
50
100
150
200
250
fireday

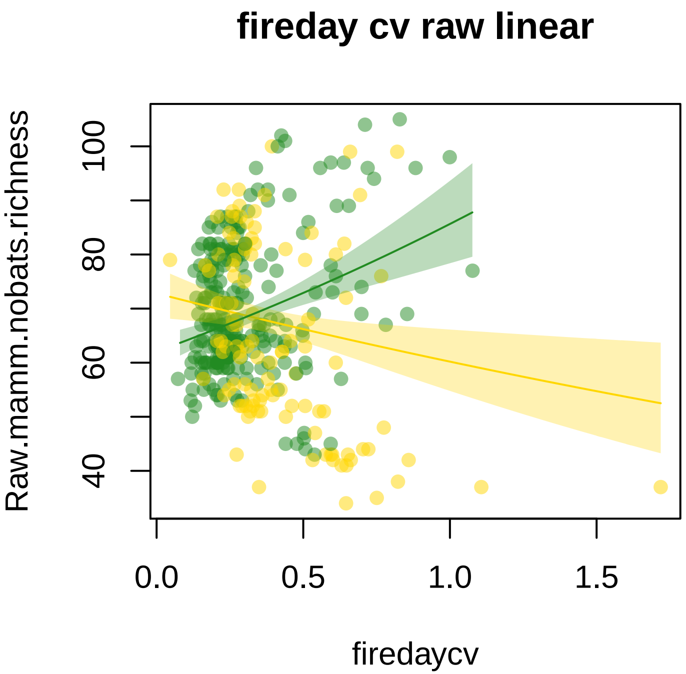
fireday cv raw linear
Raw.mamm.nobats.richness
40
60
80
100
0.0
0.5
1.0
1.5
firedaycv

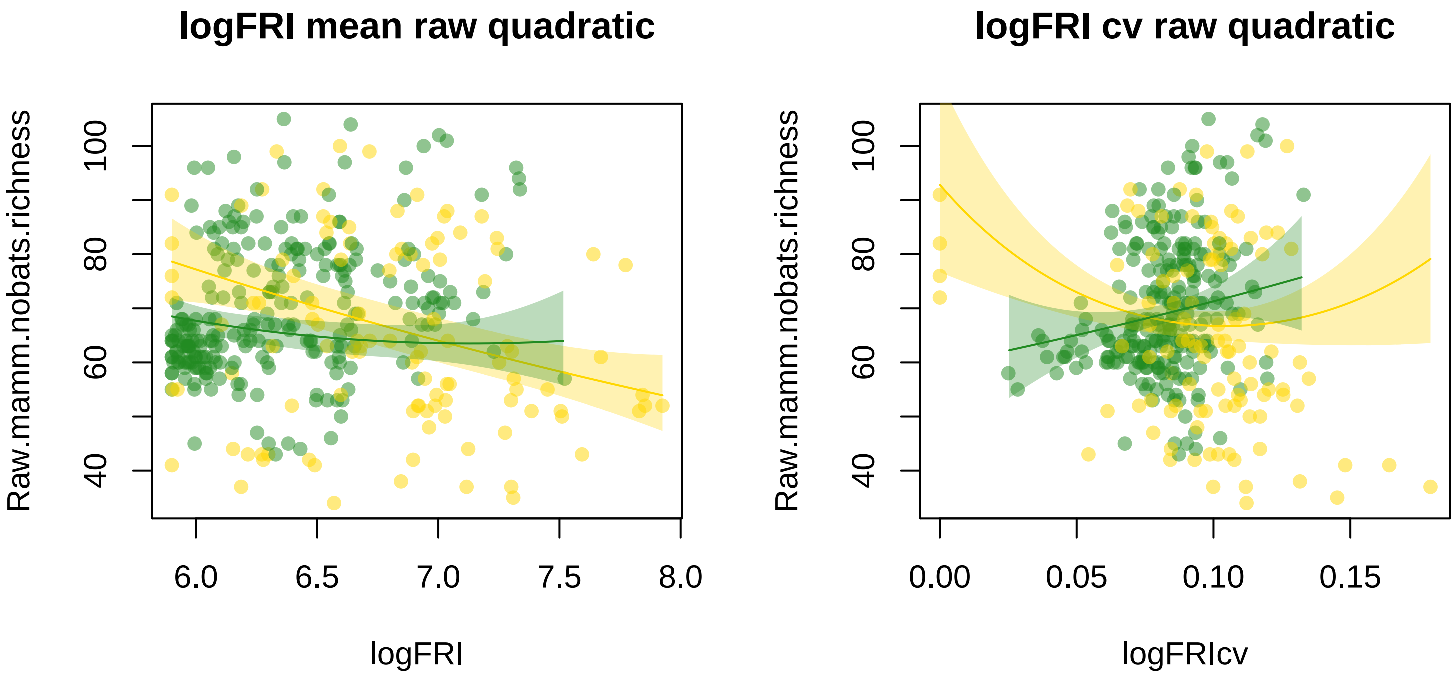

logFRI mean raw quadratic
Raw.mamm.nobats.richness
40
60
80
100
6.0
6.5
7.0
7.5
8.0
logFRI
logFRI cv raw quadratic
Raw.mamm.nobats.richness
40
60
80
100
0.00
0.05
0.10
0.15
logFRIcv

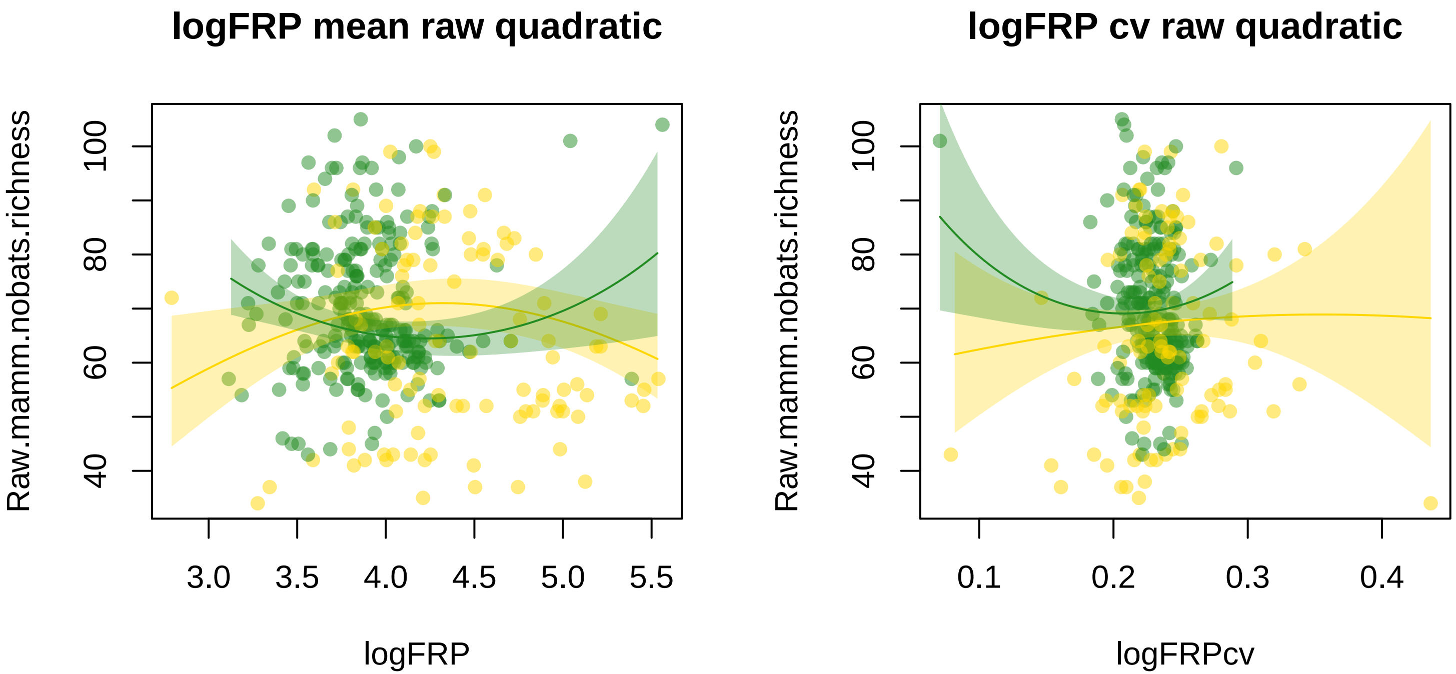
logFRP mean raw quadratic
logFRP cv raw quadratic
Raw.mamm.nobats.richness
40
60
80
100
3.0
3.5
4.0
4.5
5.0
5.5
logFRP
0.1
0.2
0.3
0.4
logFRPcv

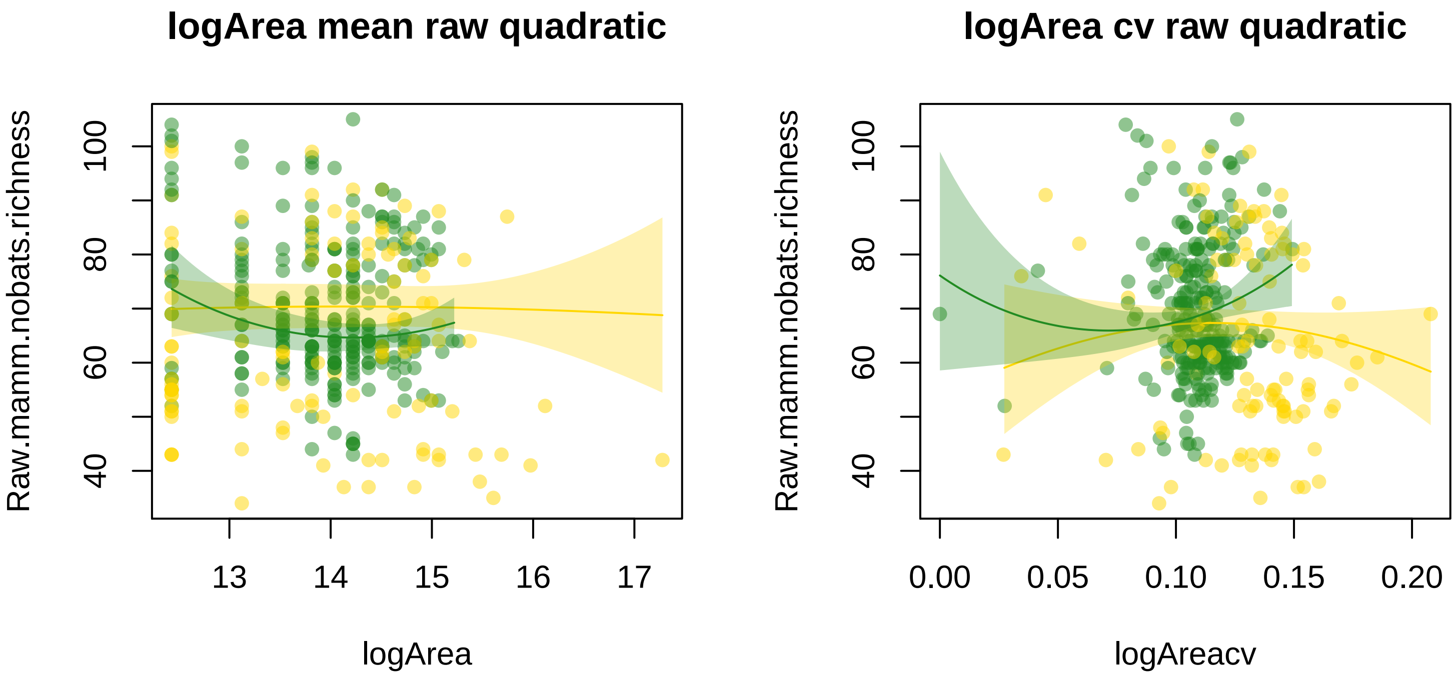
logArea mean raw quadratic
Raw.mamm.nobats.richness
40
60
80
100
13
14
15
16
17
logArea
logArea cv raw quadratic
Raw.mamm.nobats.richness
40
60
80
100
0.00
0.05
0.10
0.15
0.20
logAreacv

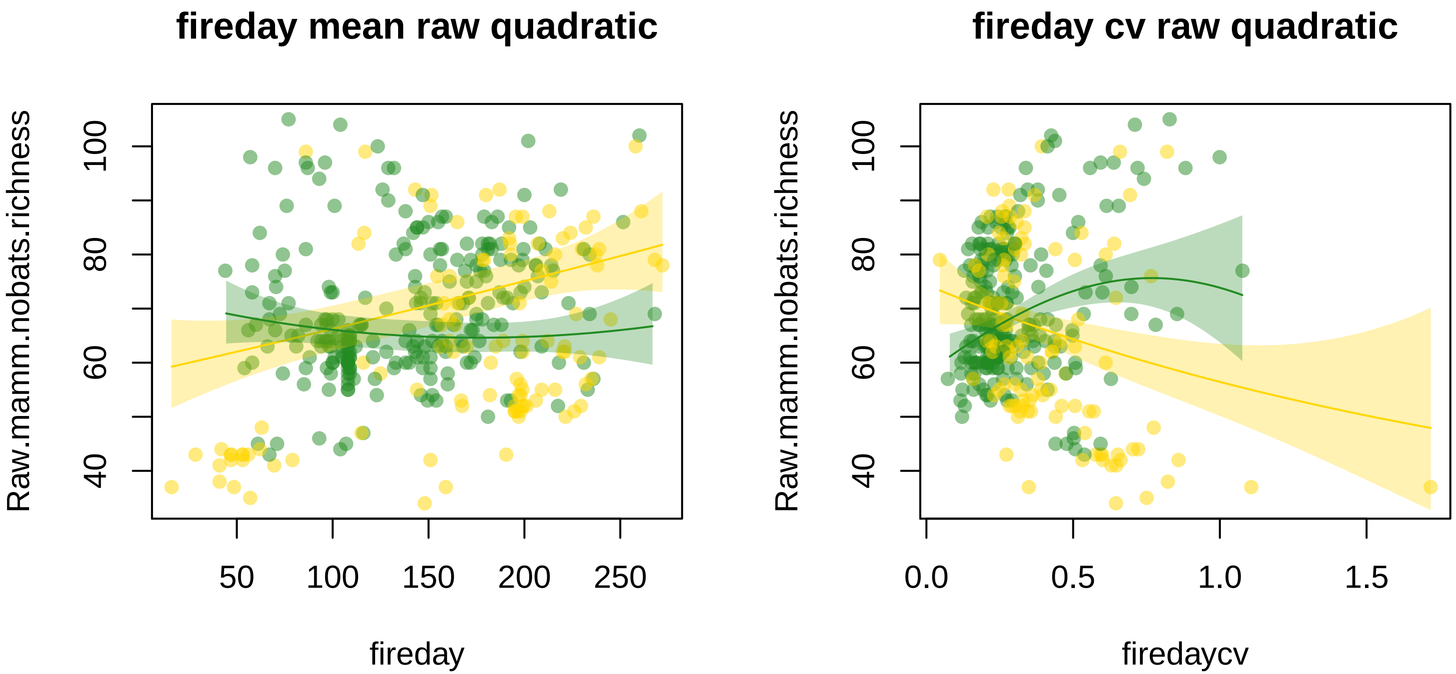
fireday mean raw quadratic
Raw.mamm.nobats.richness
40
60
80
100
50
100
150
200
250
fireday
fireday cv raw quadratic
Raw.mamm.nobats.richness
40
60
80
100
0.0
0.5
1.0
1.5
firedaycv

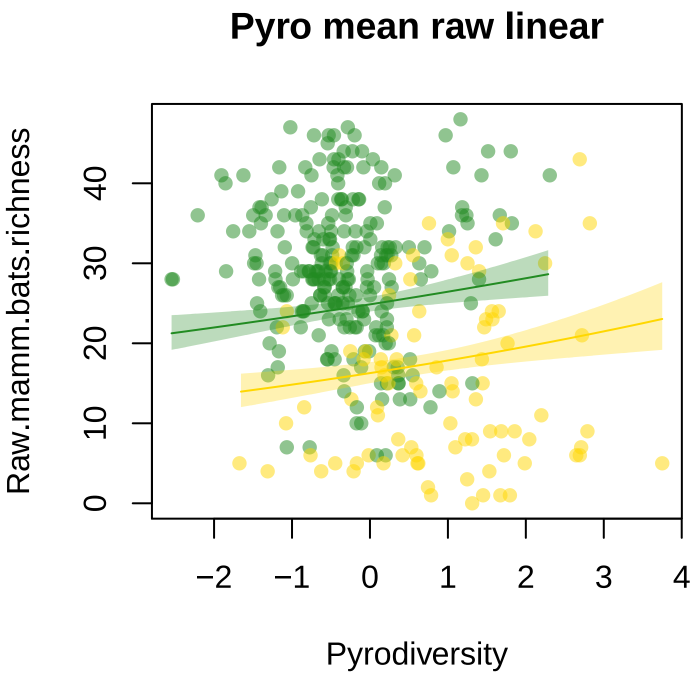
Pyro mean raw linear
Raw.mamm.bats.richness
0
10
20
30
40
−2
−1
0
1
2
3
4
Pyrodiversity

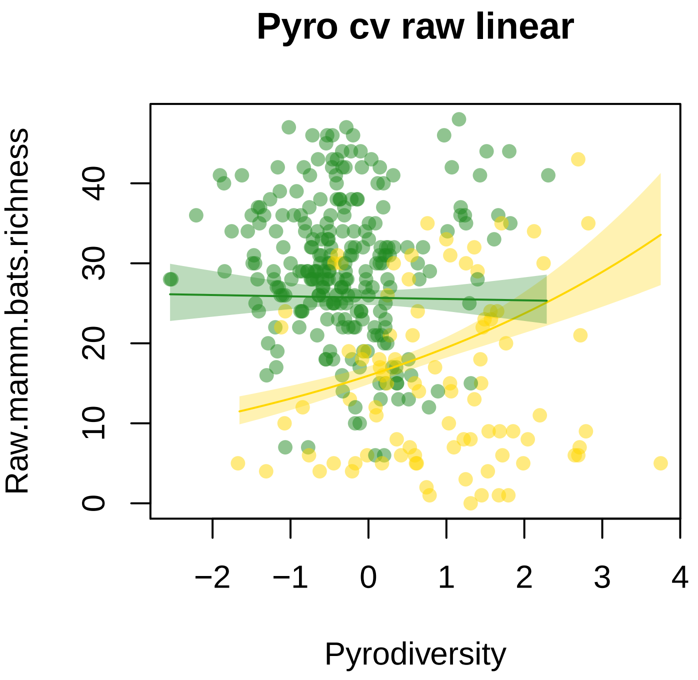
Pyro cv raw linear
Raw.mamm.bats.richness
0
10
20
30
40
−2
−1
0
1
2
3
4
Pyrodiversity

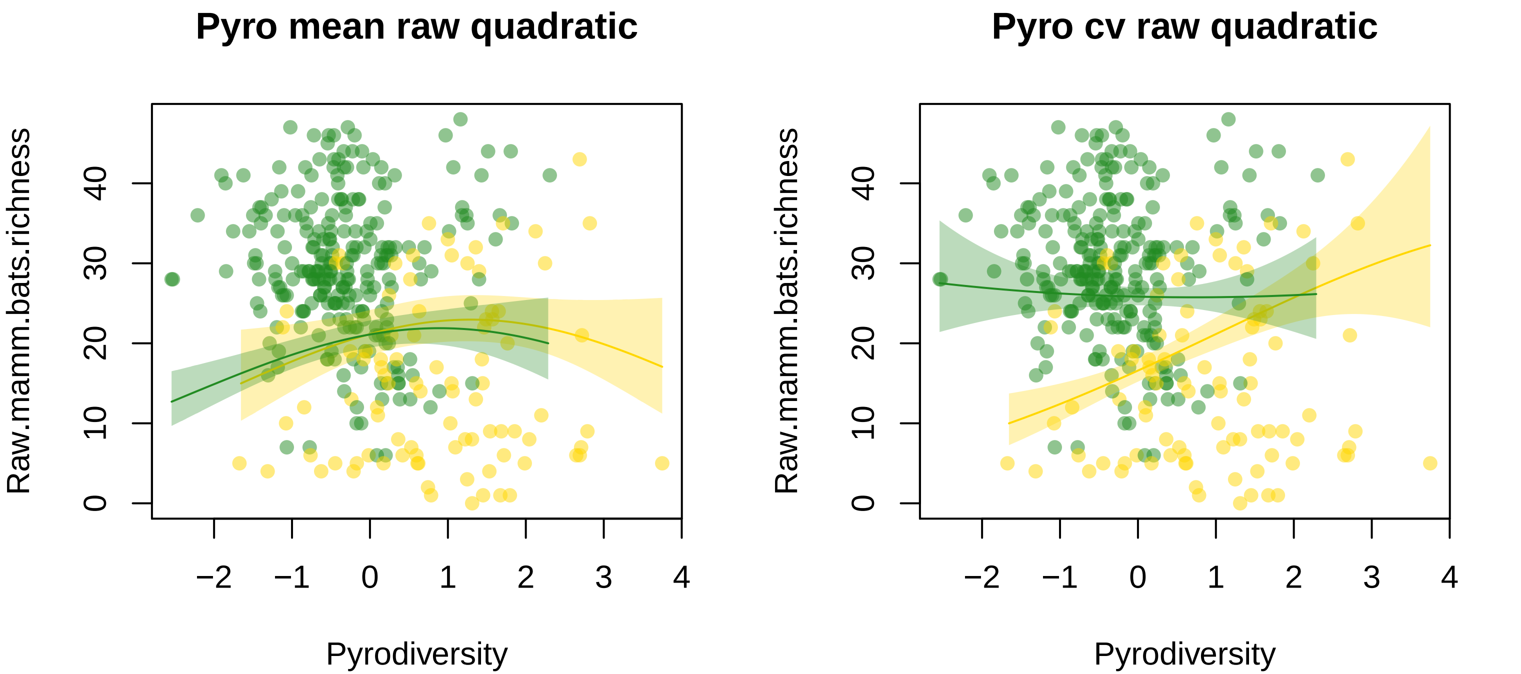
Pyro mean raw quadratic
Pyro cv raw quadratic
Raw.mamm.bats.richness
0
10
20
30
40
−2
−1
0
1
2
3
4
Pyrodiversity

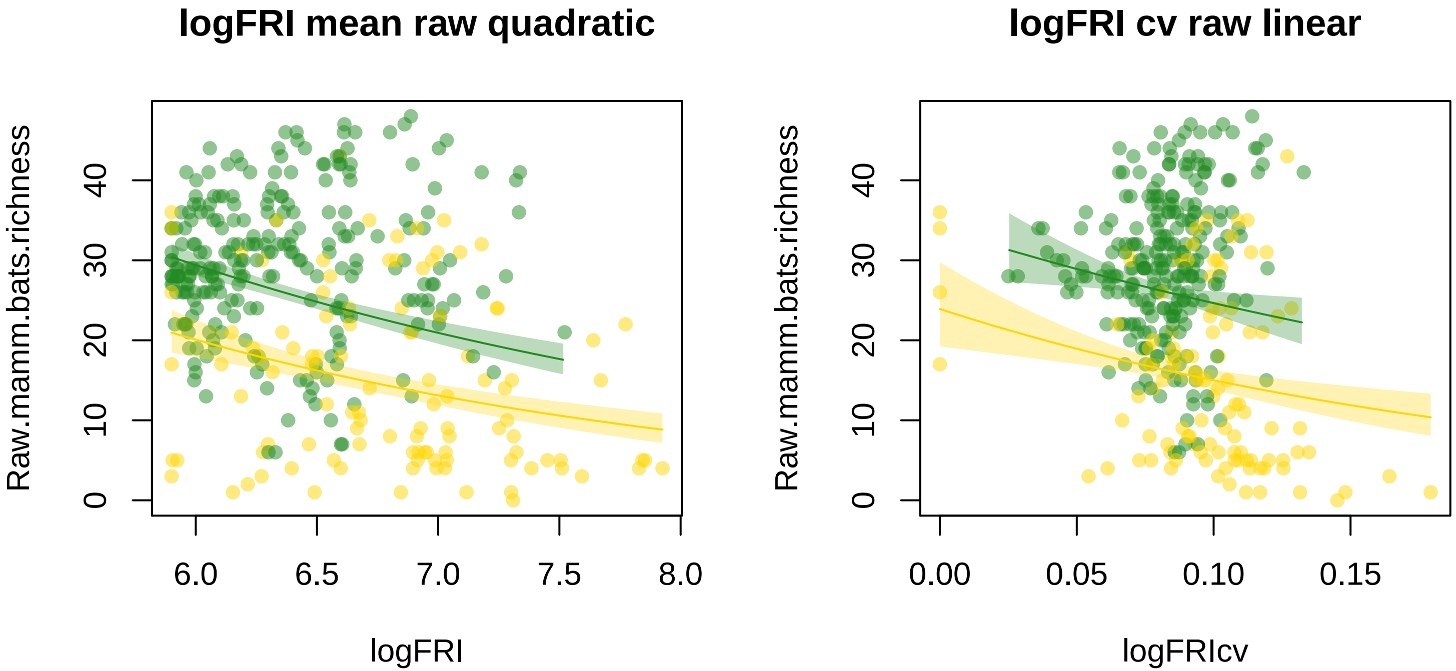

logFRI mean raw quadratic
Raw.mamm.bats.richness
0
10
20
30
40
6.0
6.5
7.0
7.5
8.0
logFRI
logFRI cv raw linear
Raw.mamm.bats.richness
0
10
20
30
40
0.00
0.05
0.10
0.15
logFRIcv

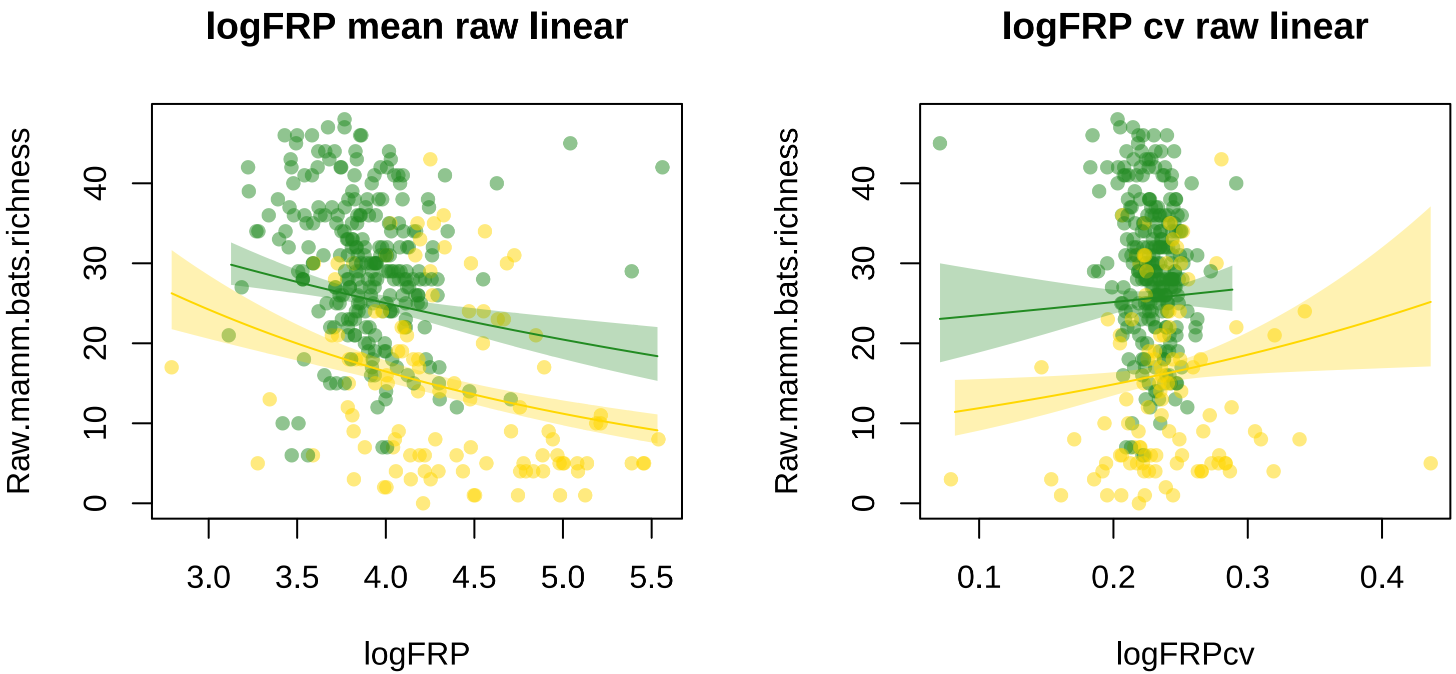
logFRP mean raw linear
Raw.mamm.bats.richness
0
10
20
30
40
3.0
3.5
4.0
4.5
5.0
5.5
logFRP
logFRP cv raw linear
Raw.mamm.bats.richness
0
10
20
30
40
0.1
0.2
0.3
0.4
logFRPcv

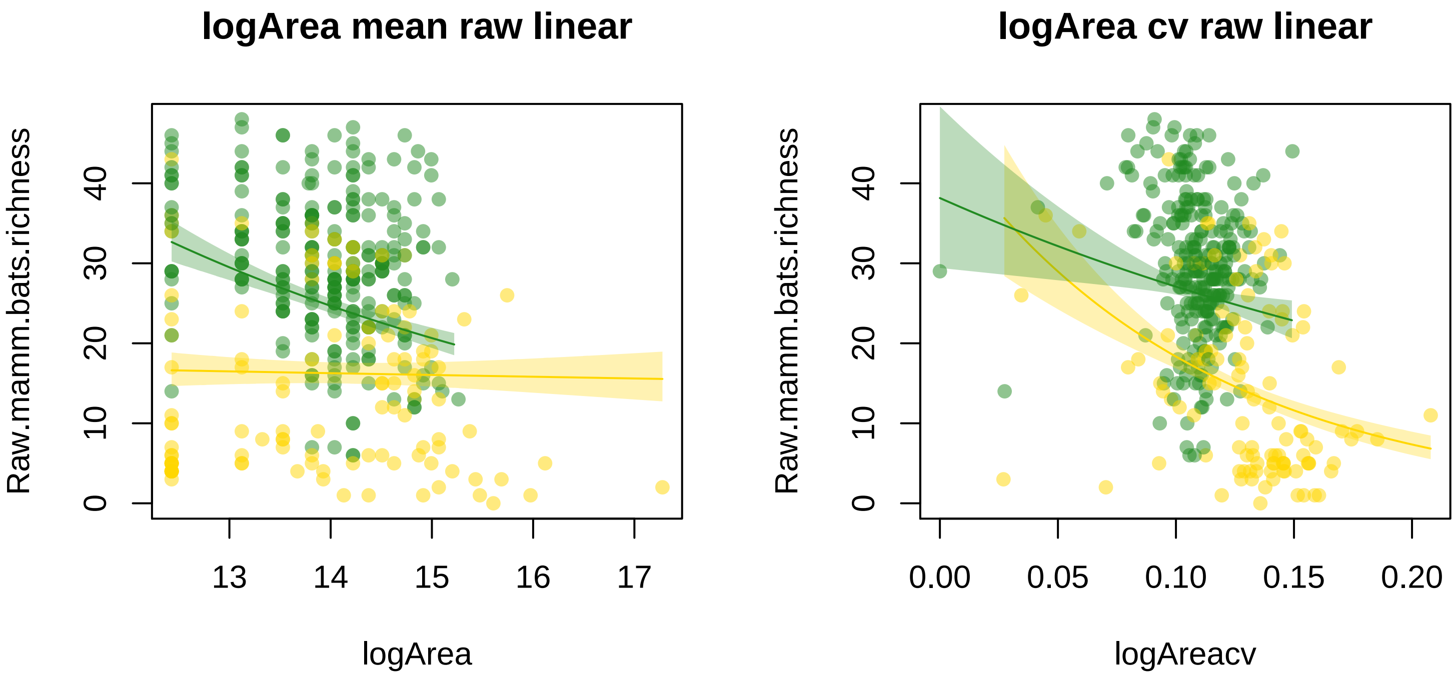
logArea mean raw linear
Raw.mamm.bats.richness
0
10
20
30
40
13
14
15
16
17
logArea
logArea cv raw linear
Raw.mamm.bats.richness
0
10
20
30
40
0.00
0.05
0.10
0.15
0.20
logAreacv

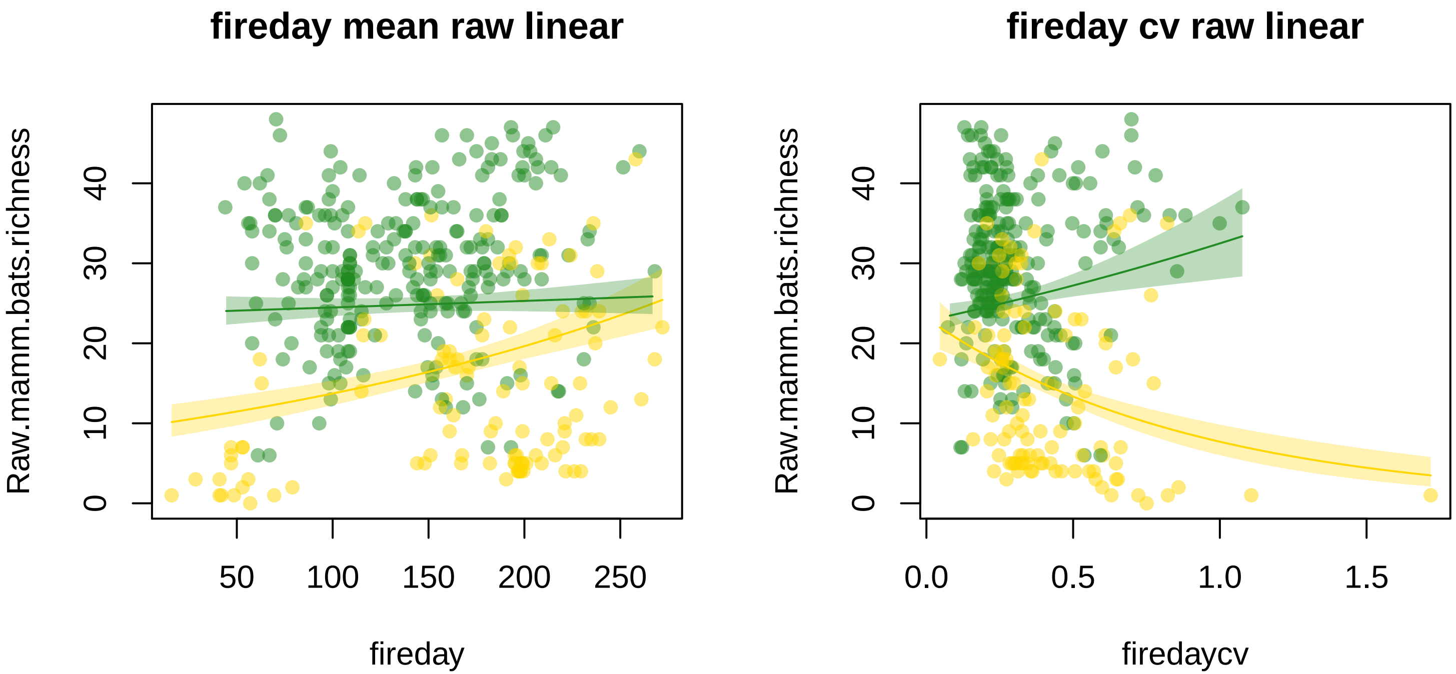
fireday mean raw linear
Raw.mamm.bats.richness
0
10
20
30
40
50
100
150
200
250
fireday
fireday cv raw linear
Raw.mamm.bats.richness
0
10
20
30
40
0.0
0.5
1.0
1.5
firedaycv

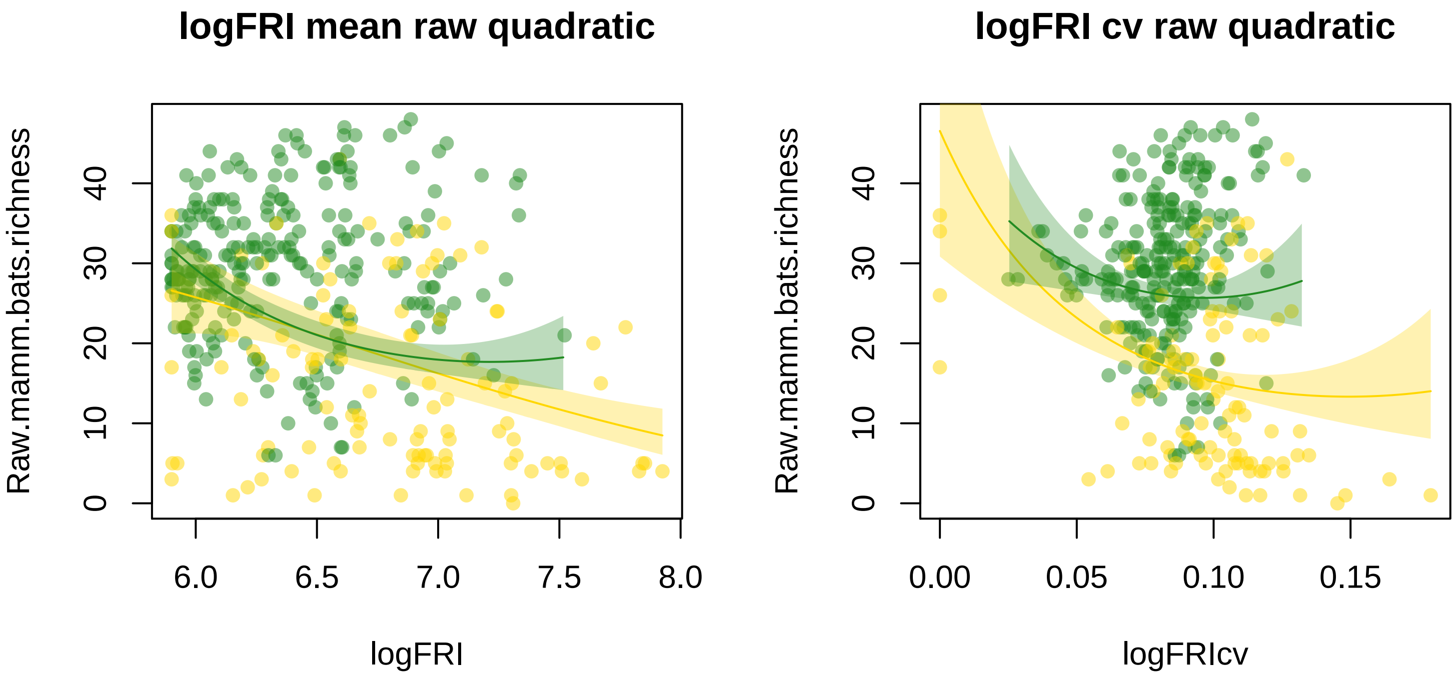
logFRI mean raw quadratic
logFRI cv raw quadratic
Raw.mamm.bats.richness
0
10
20
30
40
6.0
6.5
7.0
7.5
8.0
logFRI
0.00
0.05
0.10
0.15
logFRIcv

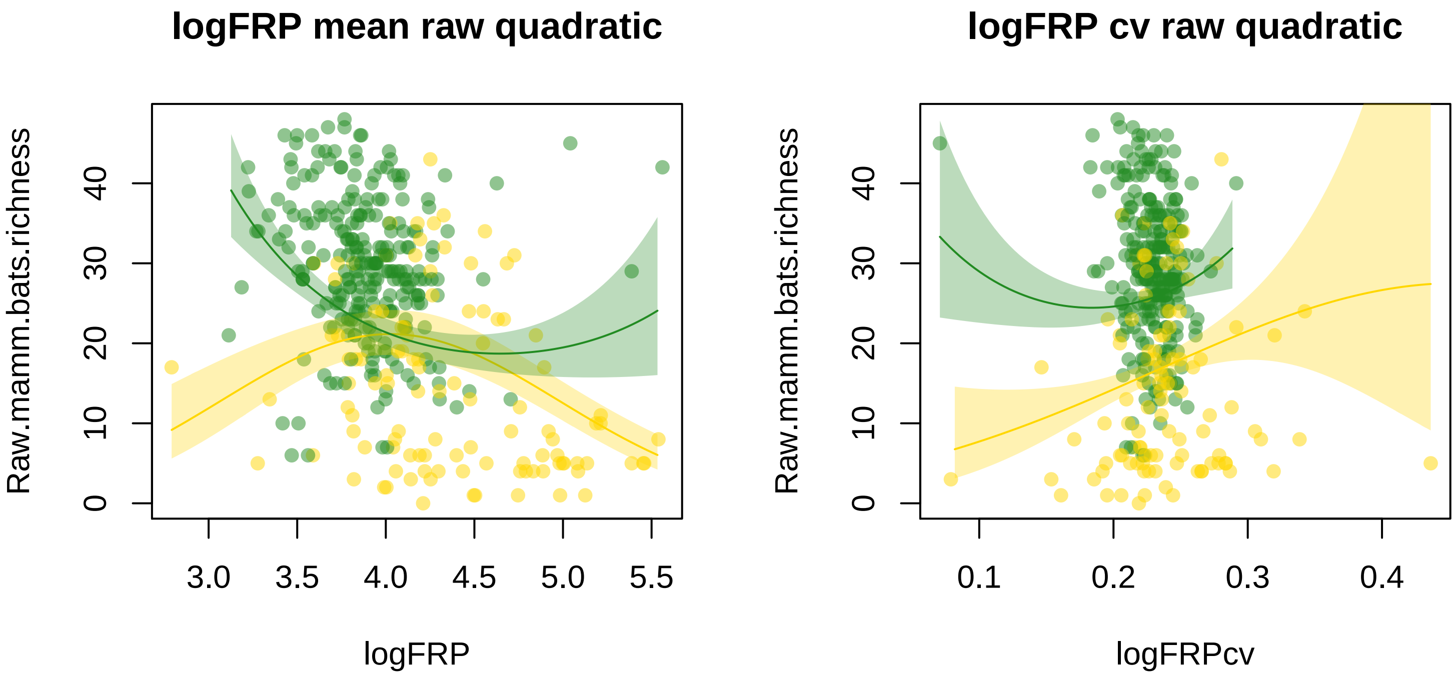
logFRP mean raw quadratic
Raw.mamm.bats.richness
0
10
20
30
40
3.0
3.5
4.0
4.5
5.0
5.5
logFRP
logFRP cv raw quadratic
Raw.mamm.bats.richness
0
10
20
30
40
0.1
0.2
0.3
0.4
logFRPcv

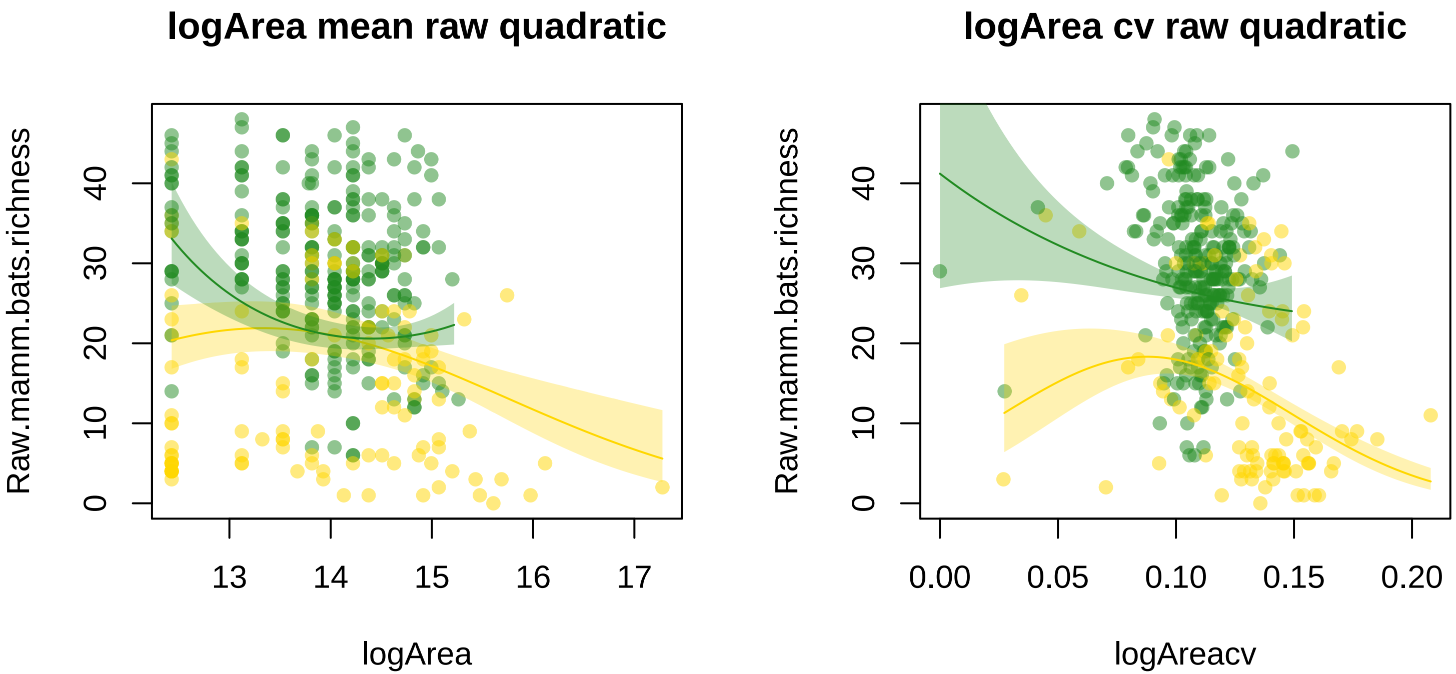

logArea mean raw quadratic
Raw.mamm.bats.richness
0
10
20
30
40
13
14
15
16
17
logArea
logArea cv raw quadratic
Raw.mamm.bats.richness
0
10
20
30
40
0.00
0.05
0.10
0.15
0.20
logAreacv

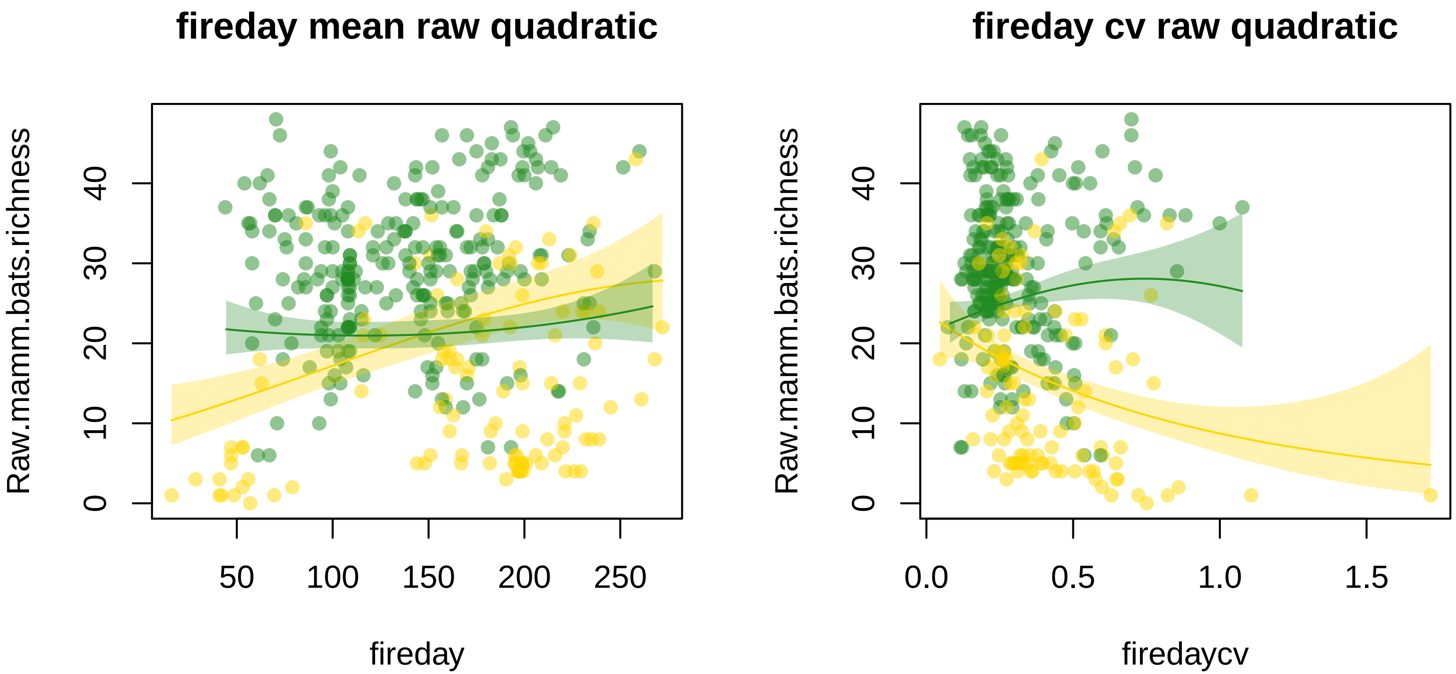
fireday mean raw quadratic
Raw.mamm.bats.richness
0
10
20
30
40
50
100
150
200
250
fireday
fireday cv raw quadratic
Raw.mamm.bats.richness
0
10
20
30
40
0.0
0.5
1.0
1.5
firedaycv

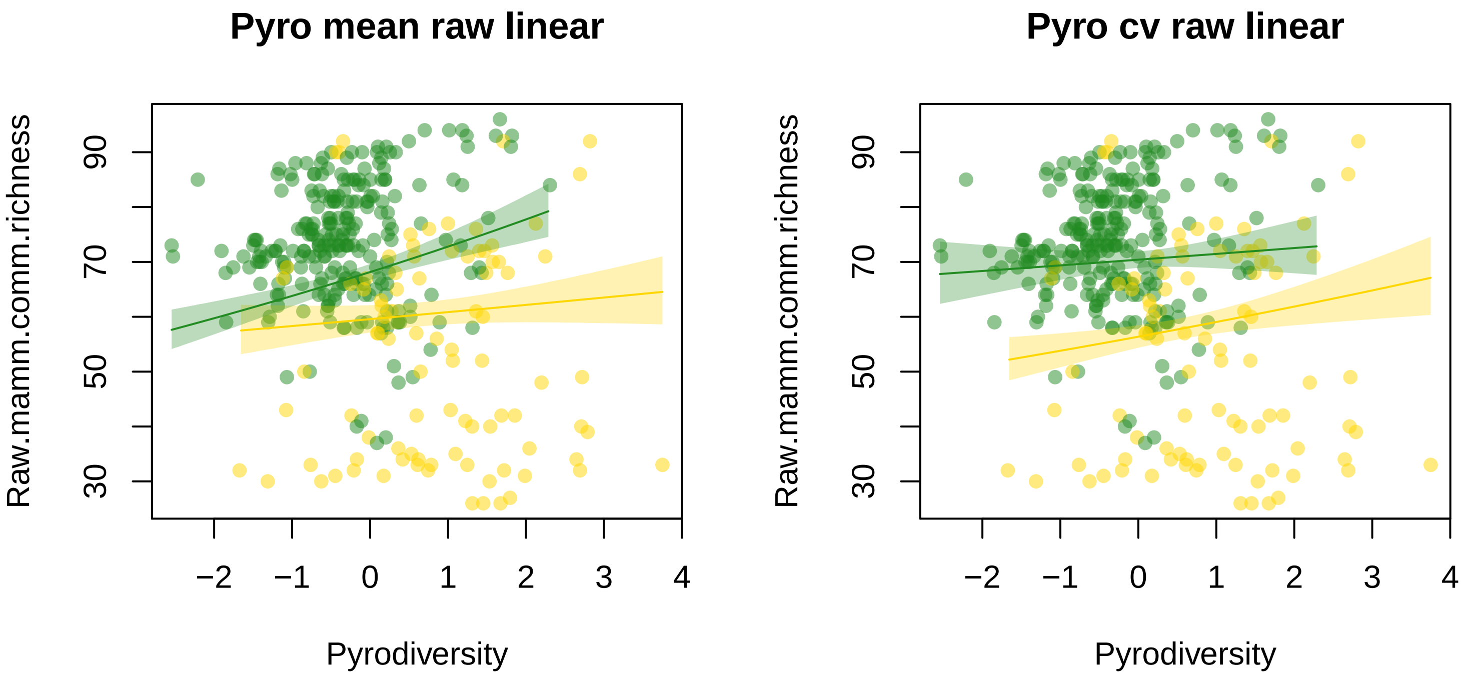

Pyro mean raw linear
Pyro cv raw linear
Raw.mamm.comm.richness
Pyrodiversity
30
50
70
90
−2
−1
0
1
2
3
4

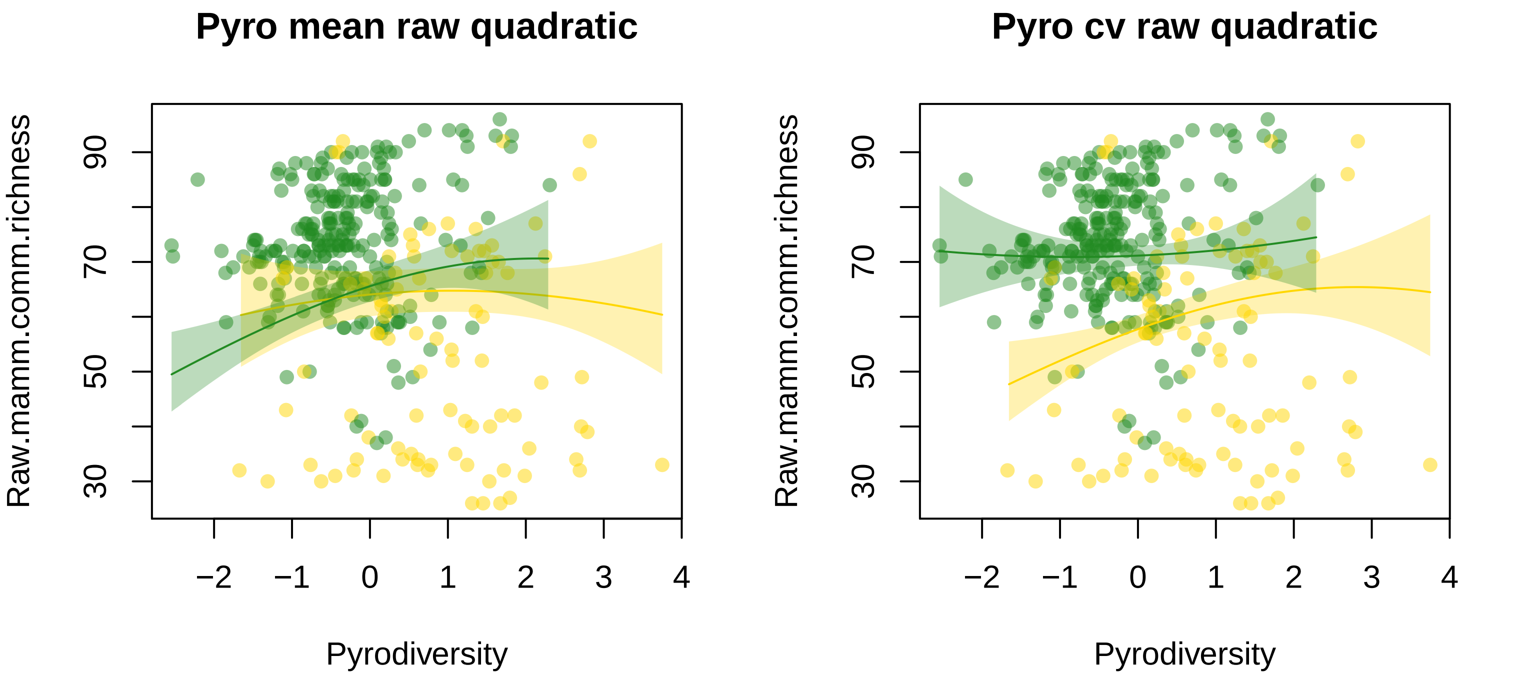

Pyro mean raw quadratic
Pyro cv raw quadratic
Raw.mamm.comm.richness
30
50
70
90
−2
−1
0
1
2
3
4
Pyrodiversity

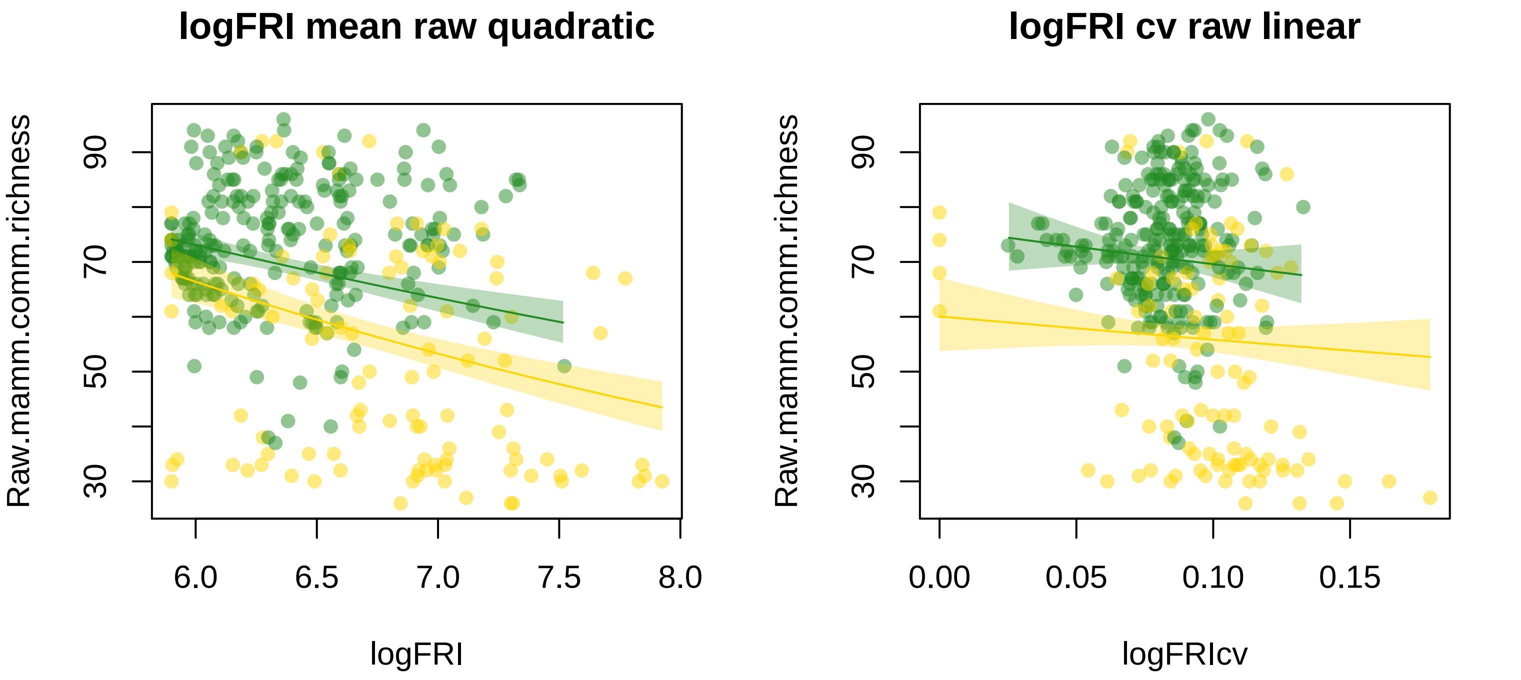

logFRI mean raw quadratic
Raw.mamm.comm.richness
90
70
50
30
6.0
6.5
7.0
7.5
8.0
logFRI
logFRI cv raw linear
Raw.mamm.comm.richness
90
70
50
30
0.00
0.05
0.10
0.15
logFRIcv

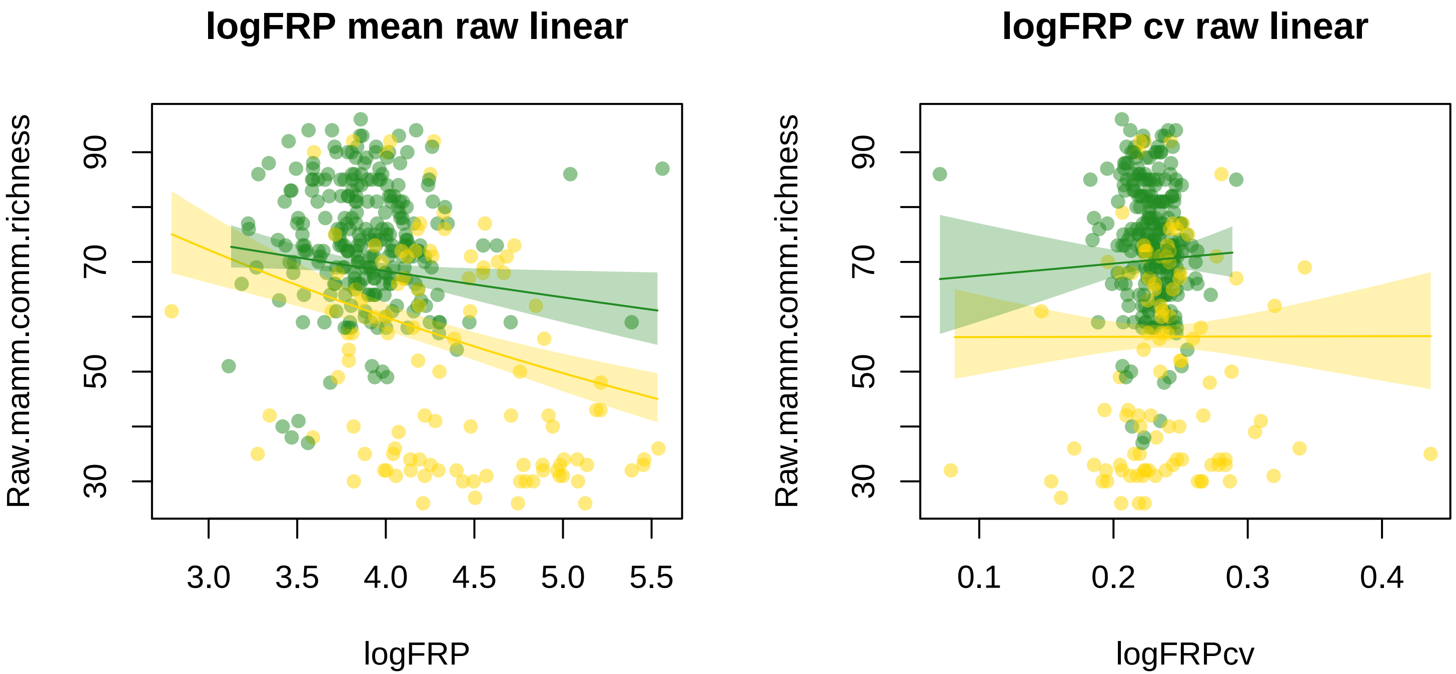

logFRP mean raw linear
logFRP cv raw linear
Raw.mamm.comm.richness
30
50
70
90
3.0
3.5
4.0
4.5
5.0
5.5
logFRP
0.1
0.2
0.3
0.4
logFRPcv

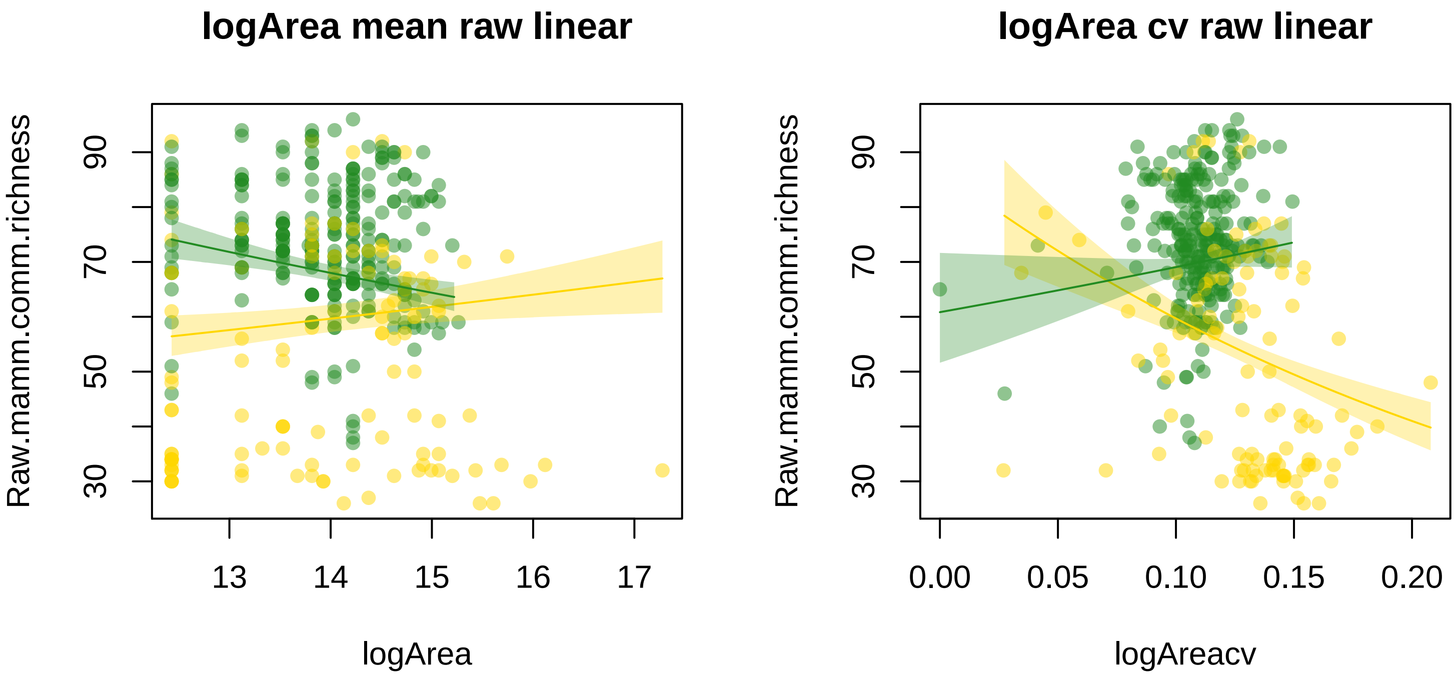
logArea mean raw linear
Raw.mamm.comm.richness
30
50
70
90
13
14
15
16
17
logArea
logArea cv raw linear
Raw.mamm.comm.richness
30
50
70
90
0.00
0.05
0.10
0.15
0.20
logAreacv

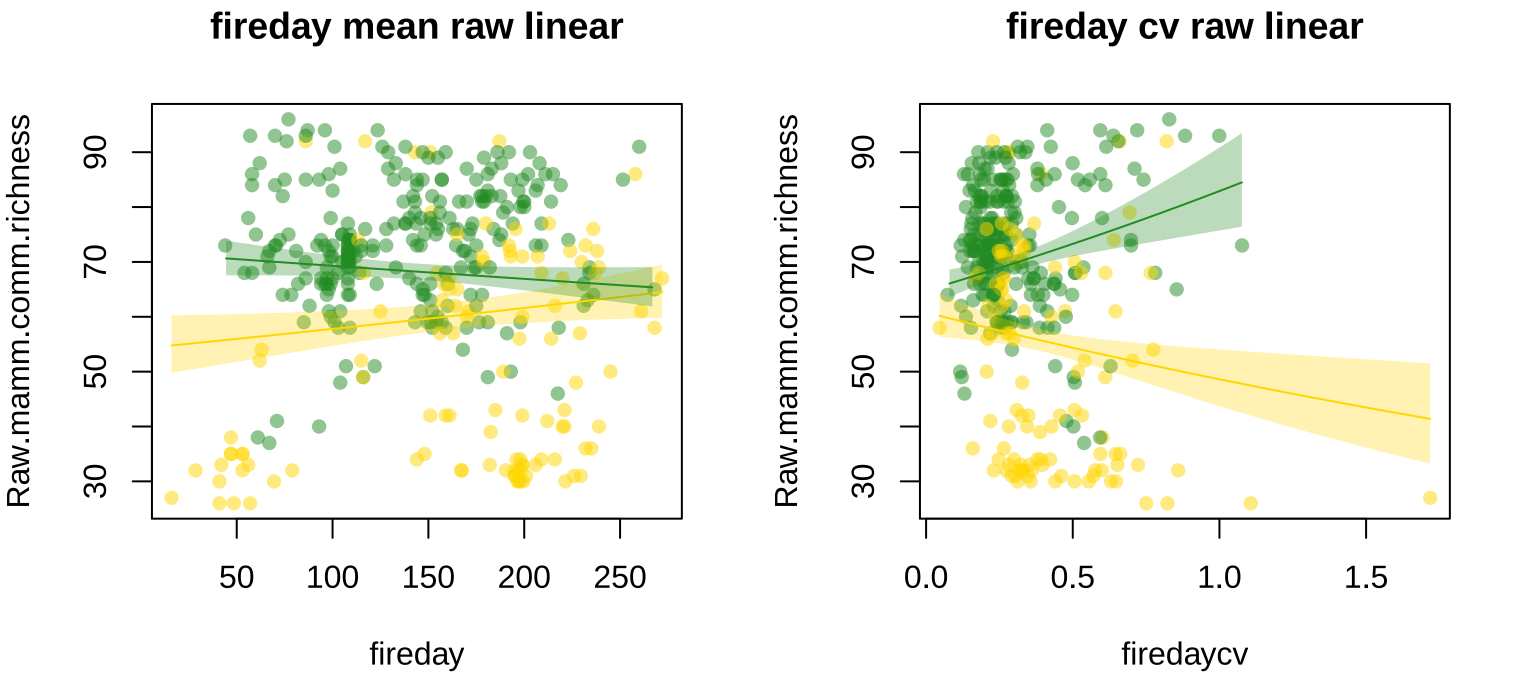

fireday mean raw linear
Raw.mamm.comm.richness
30
50
70
90
50
100
150
200
250
fireday
fireday cv raw linear
Raw.mamm.comm.richness
30
50
70
90
0.0
0.5
1.0
1.5
firedaycv

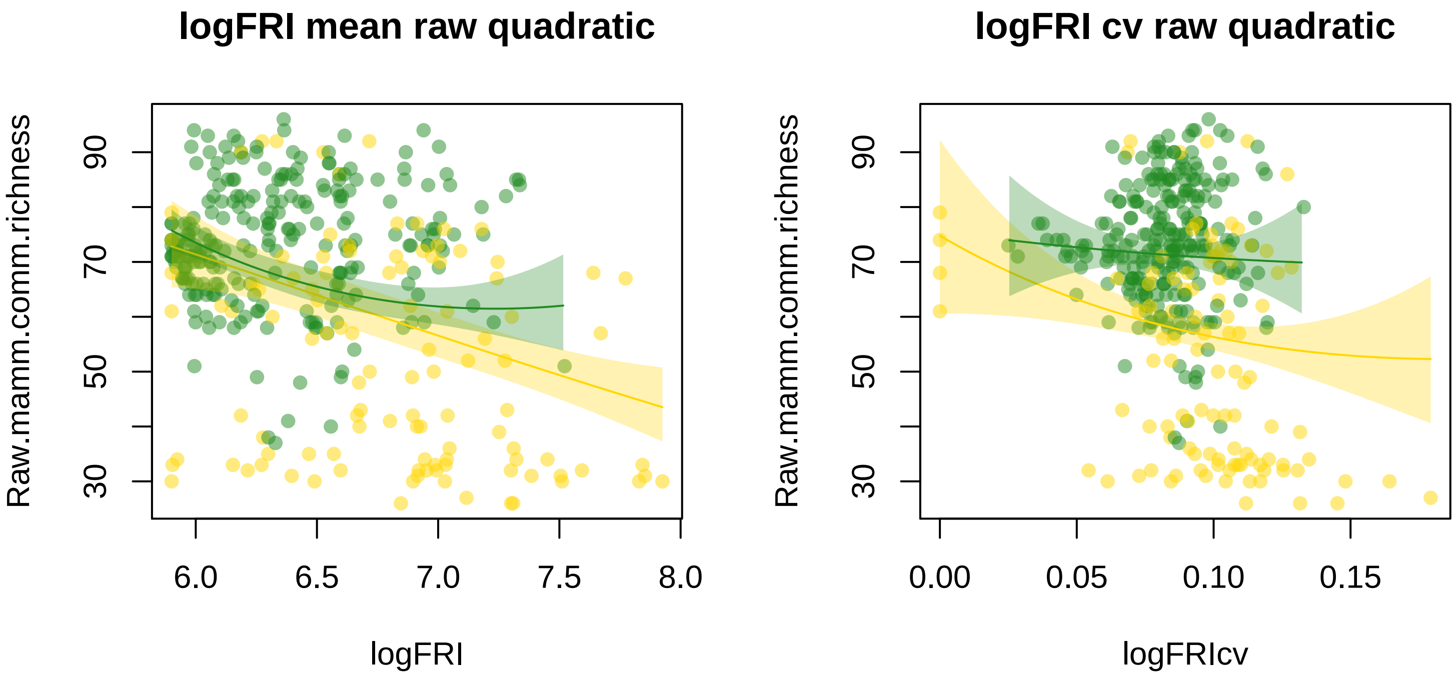

logFRI mean raw quadratic
Raw.mamm.comm.richness
30
50
70
90
6.0
6.5
7.0
7.5
8.0
logFRI
logFRI cv raw quadratic
Raw.mamm.comm.richness
30
50
70
90
0.00
0.05
0.10
0.15
logFRIcv

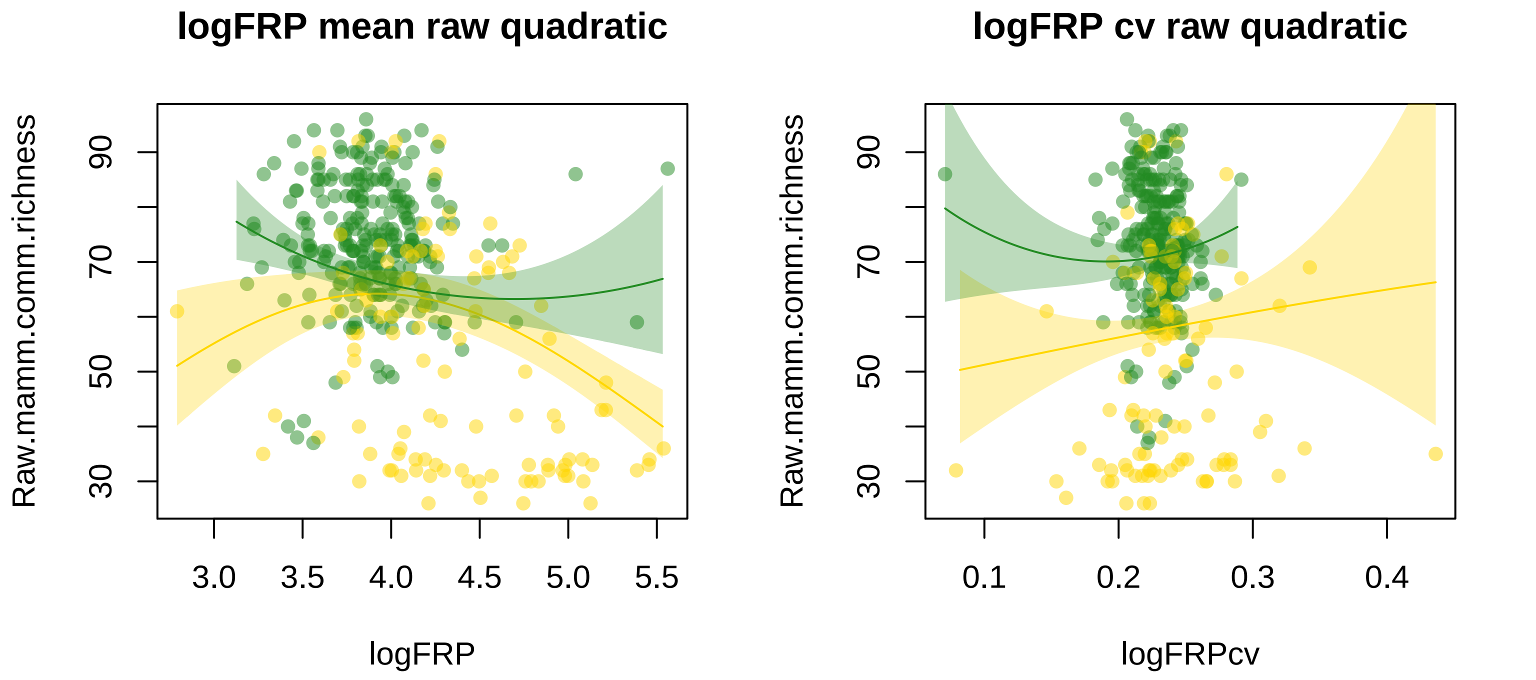

logFRP mean raw quadratic
logFRP cv raw quadratic
Raw.mamm.comm.richness
30
50
70
90
3.0
3.5
4.0
4.5
5.0
5.5
logFRP
0.1
0.2
0.3
0.4
logFRPcv

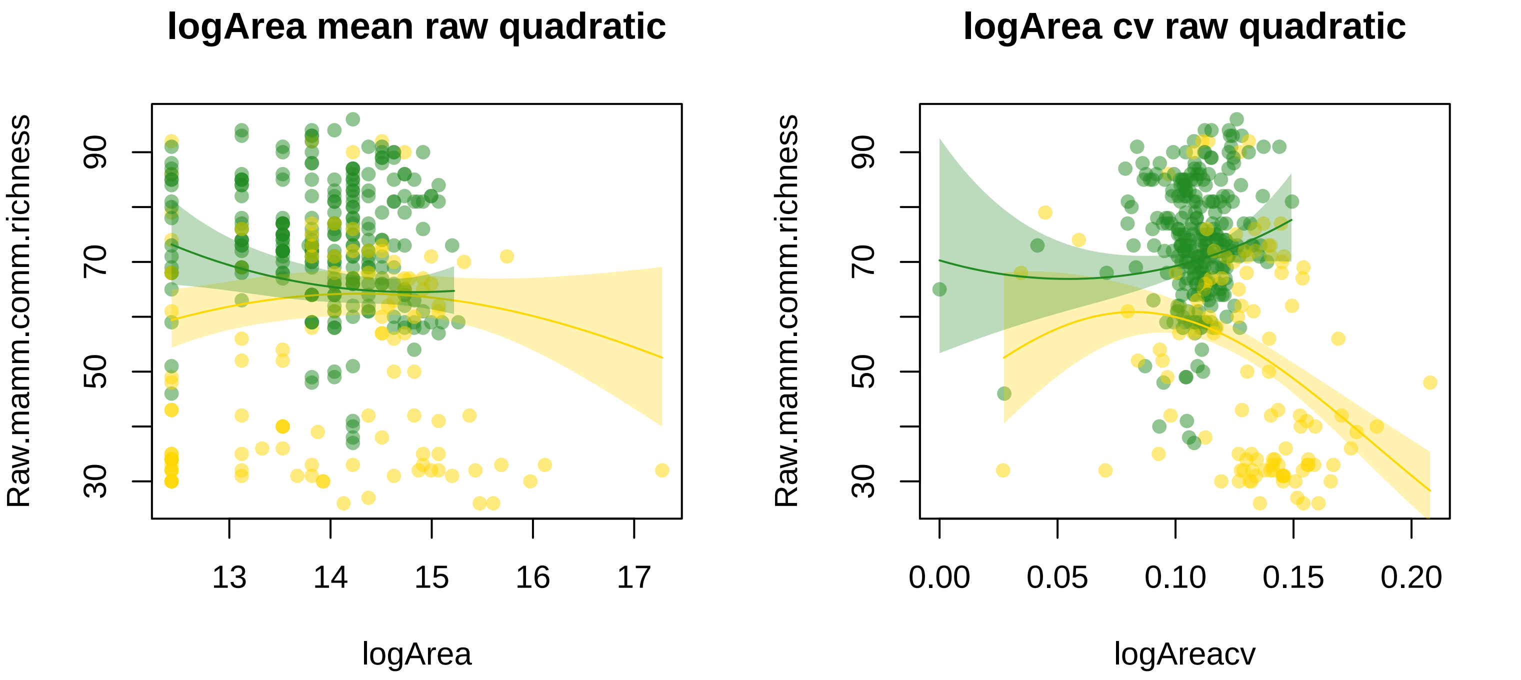
logArea mean raw quadratic
Raw.mamm.comm.richness
30
50
70
90
13
14
15
16
17
logArea
logArea cv raw quadratic
Raw.mamm.comm.richness
30
50
70
90
0.00
0.05
0.10
0.15
0.20
logAreacv

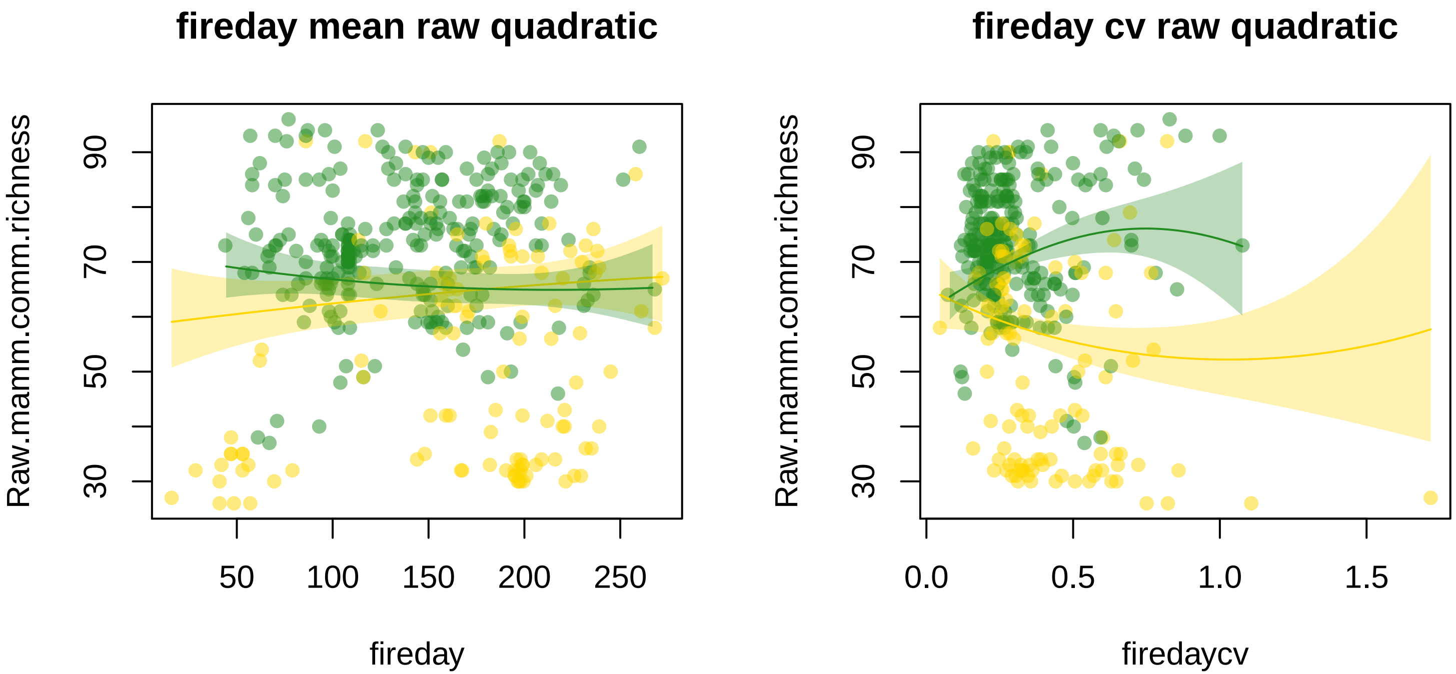
fireday mean raw quadratic
fireday cv raw quadratic
Raw.mamm.comm.richness
30
50
70
90
50
100
150
200
250
fireday
0.0
0.5
1.0
1.5
firedaycv

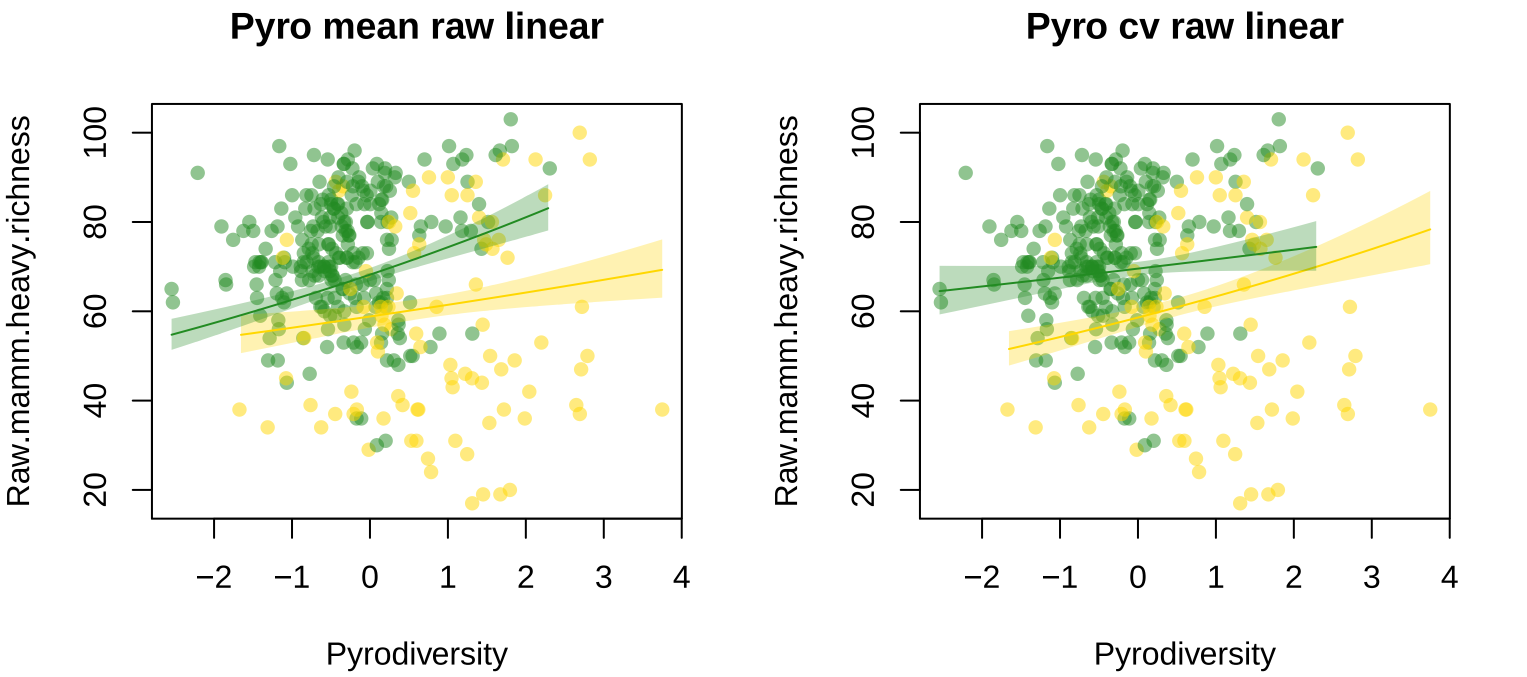

Pyro mean raw linear
Pyro cv raw linear
Raw.mamm.heavy.richness
20
40
60
80
100
−2
−1
0
1
2
3
4
Pyrodiversity

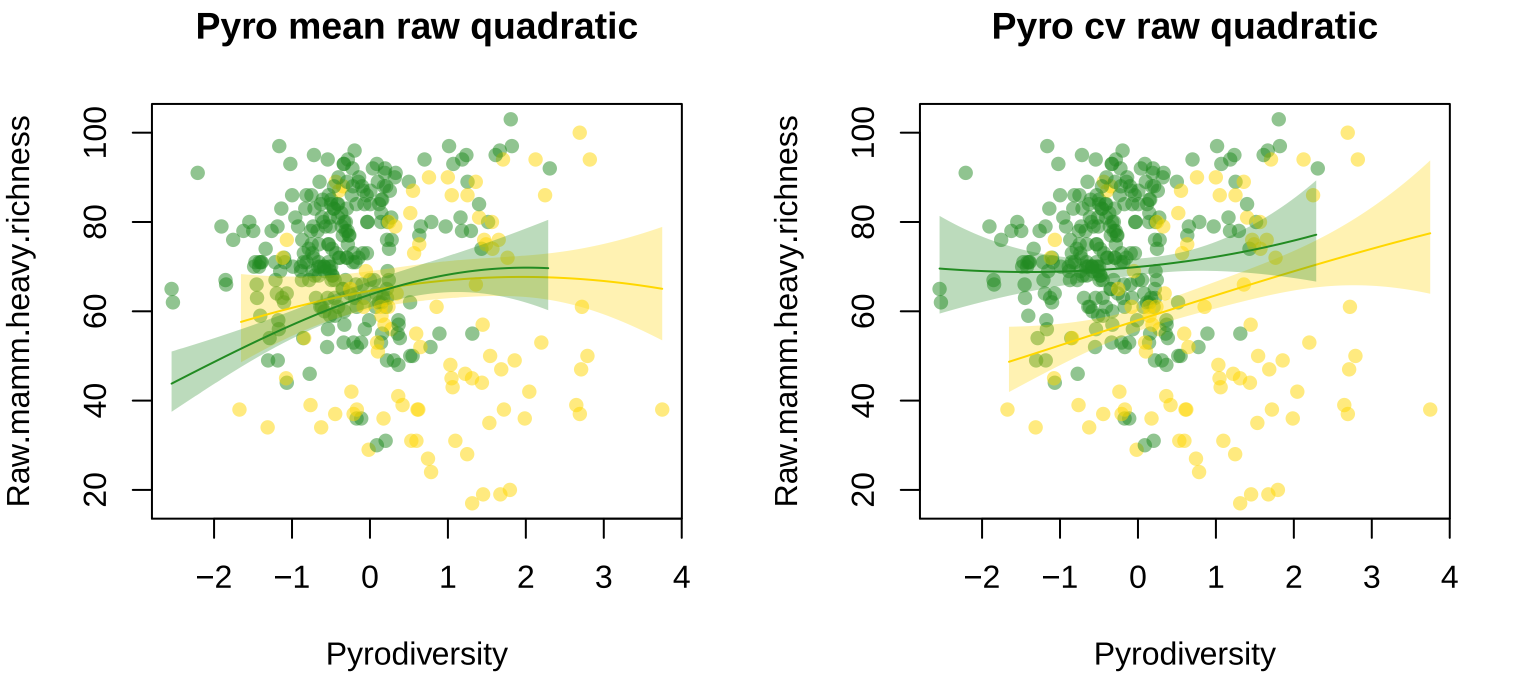
Pyro mean raw quadratic
Pyro cv raw quadratic
Raw.mamm.heavy.richness
20
40
60
80
100
−2
−1
0
1
2
3
4
Pyrodiversity

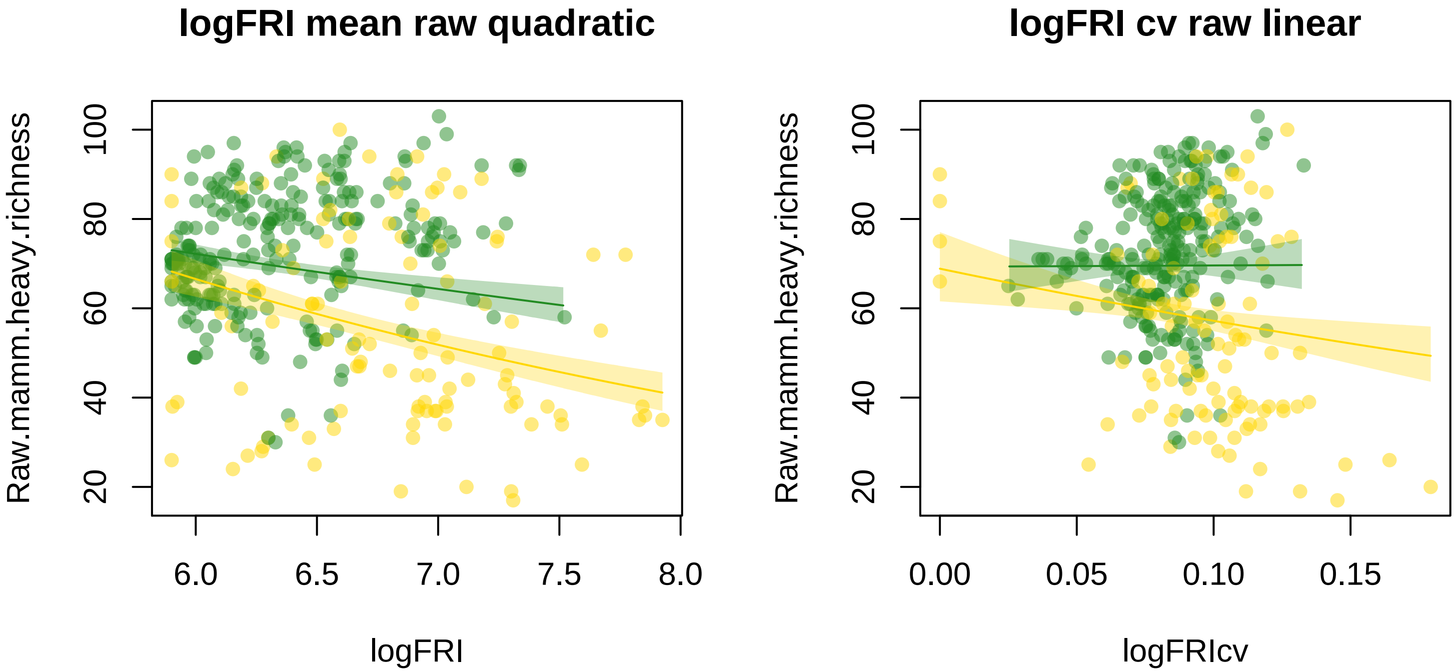
logFRI mean raw quadratic
Raw.mamm.heavy.richness
20
40
60
80
100
6.0
6.5
7.0
7.5
8.0
logFRI
logFRI cv raw linear
Raw.mamm.heavy.richness
20
40
60
80
100
0.00
0.05
0.10
0.15
logFRIcv

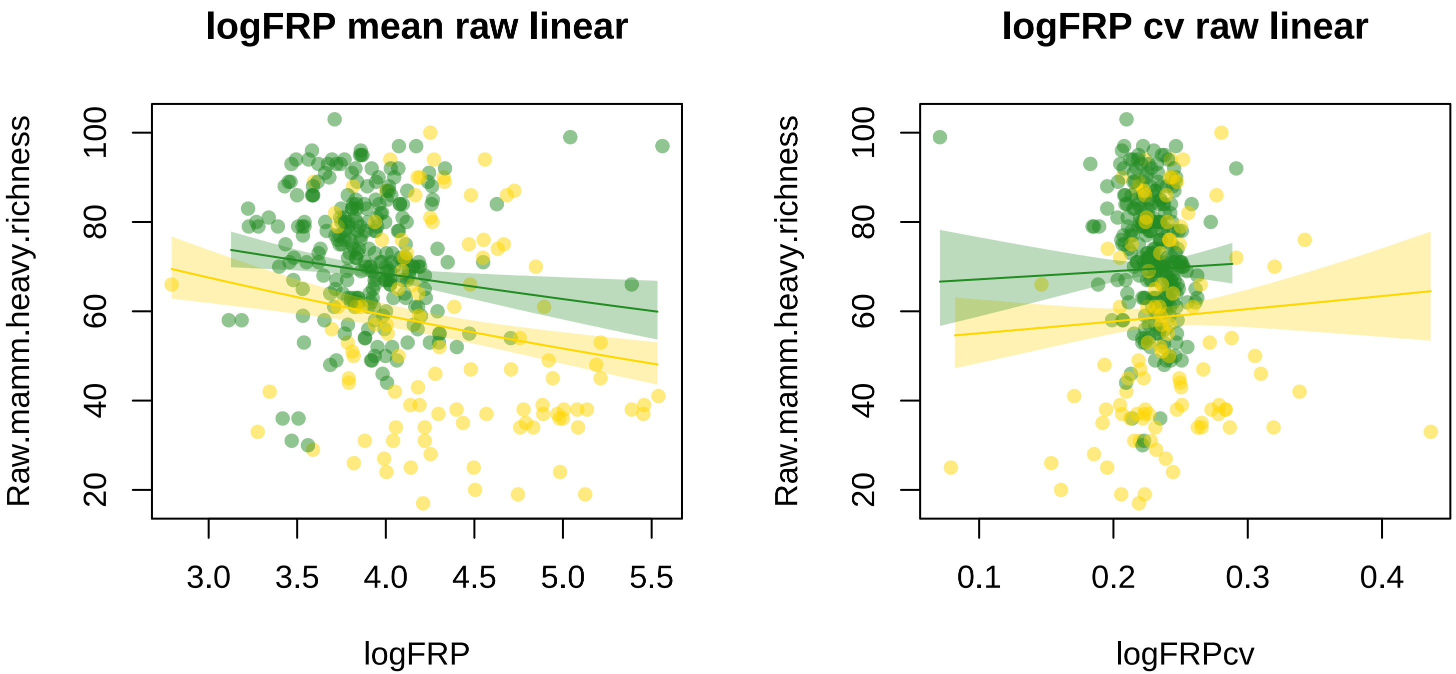
logFRP mean raw linear
Raw.mamm.heavy.richness
20
40
60
80
100
3.0
3.5
4.0
4.5
5.0
5.5
logFRP
logFRP cv raw linear
Raw.mamm.heavy.richness
20
40
60
80
100
0.1
0.2
0.3
0.4
logFRPcv

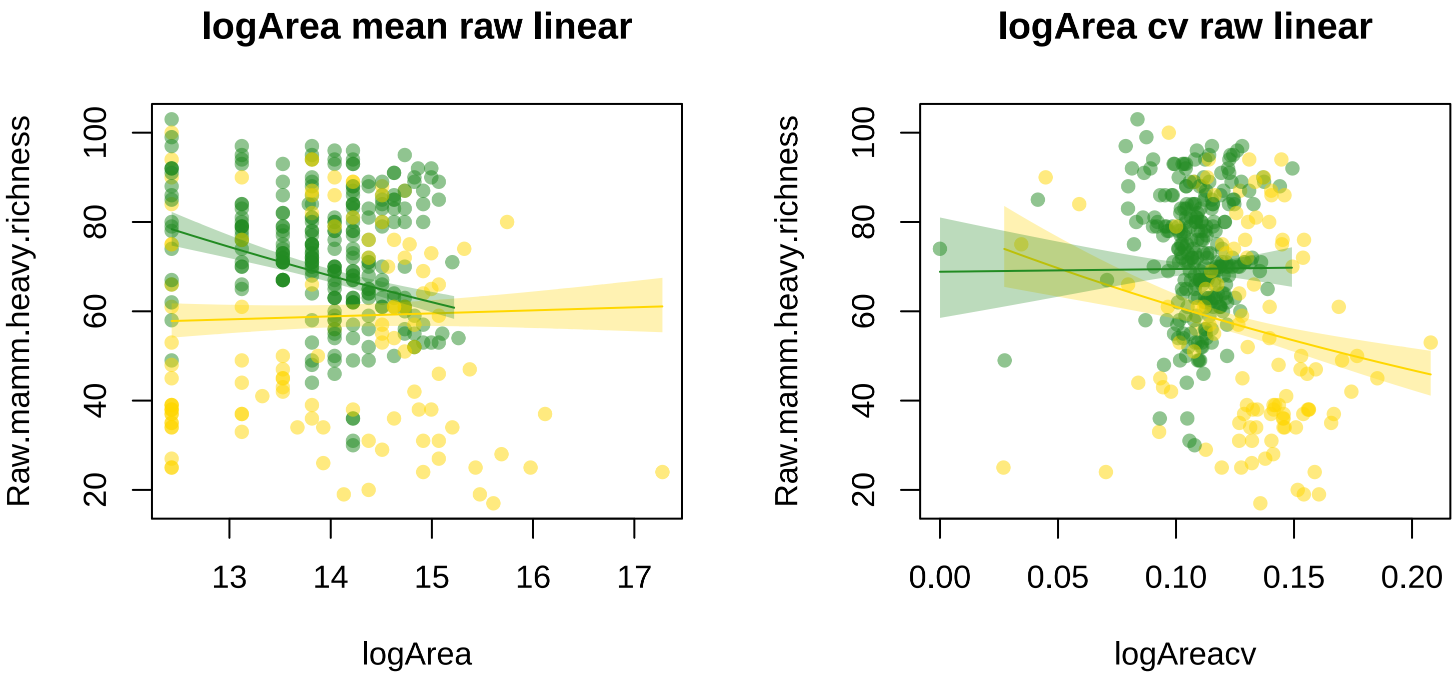
logArea mean raw linear
Raw.mamm.heavy.richness
20
40
60
80
100
13
14
15
16
17
logArea
logArea cv raw linear
Raw.mamm.heavy.richness
20
40
60
80
100
0.00
0.05
0.10
0.15
0.20
logAreacv

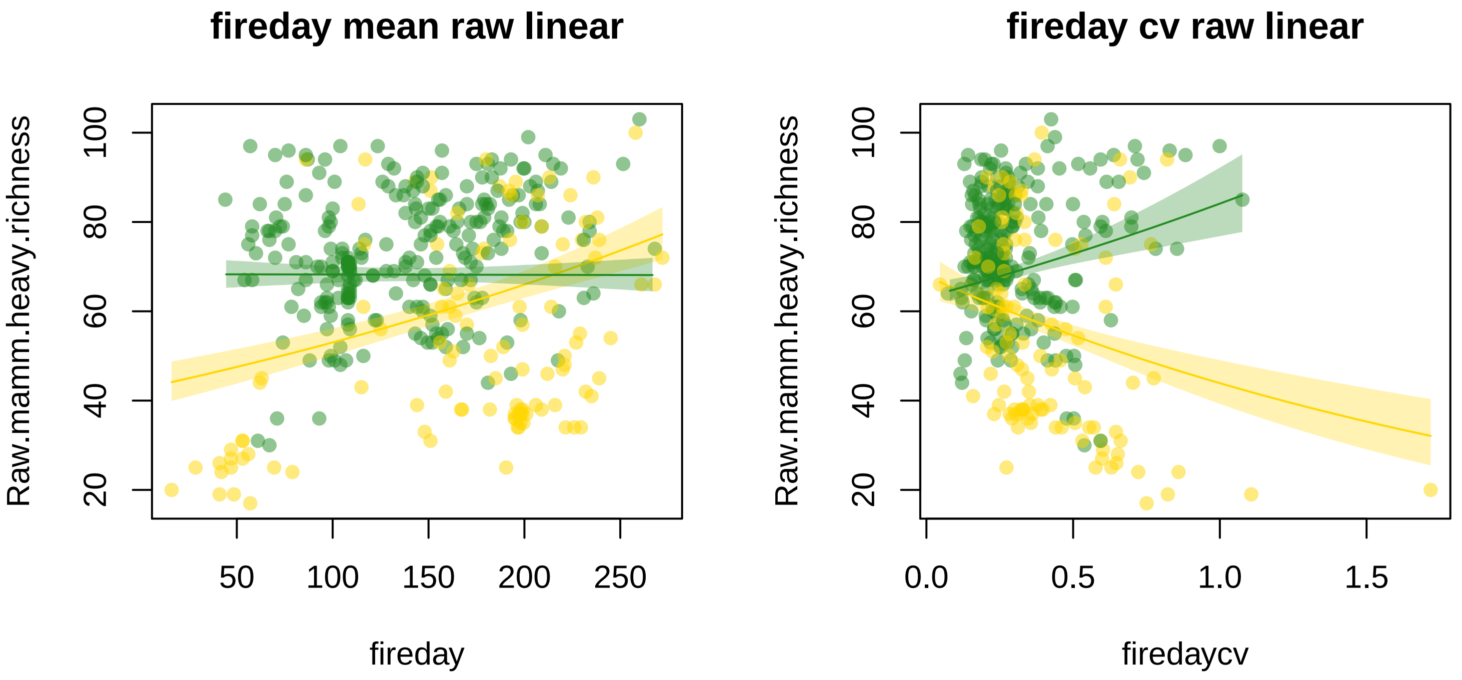
fireday mean raw linear
Raw.mamm.heavy.richness
fireday
fireday cv raw linear
Raw.mamm.heavy.richness
firedaycv

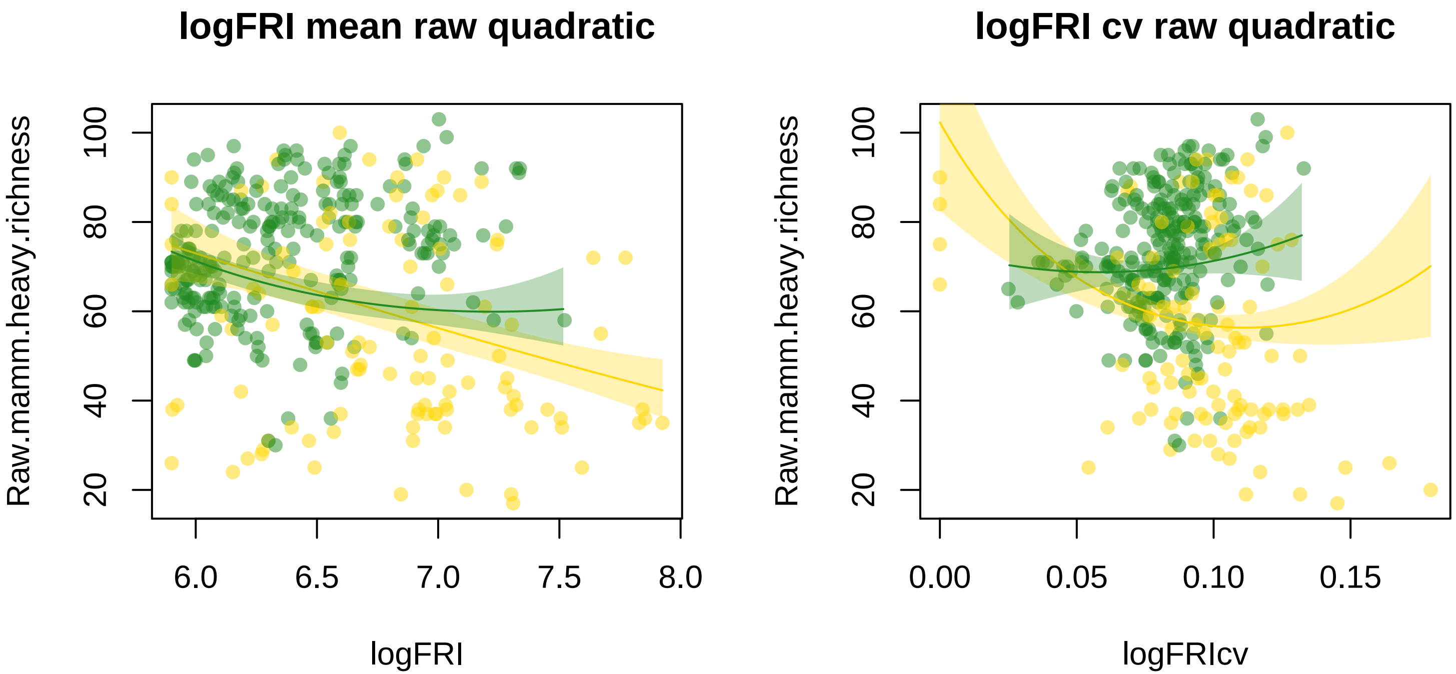
logFRI mean raw quadratic
Raw.mamm.heavy.richness
20
40
60
80
100
6.0
6.5
7.0
7.5
8.0
logFRI
logFRI cv raw quadratic
Raw.mamm.heavy.richness
20
40
60
80
100
0.00
0.05
0.10
0.15
logFRIcv

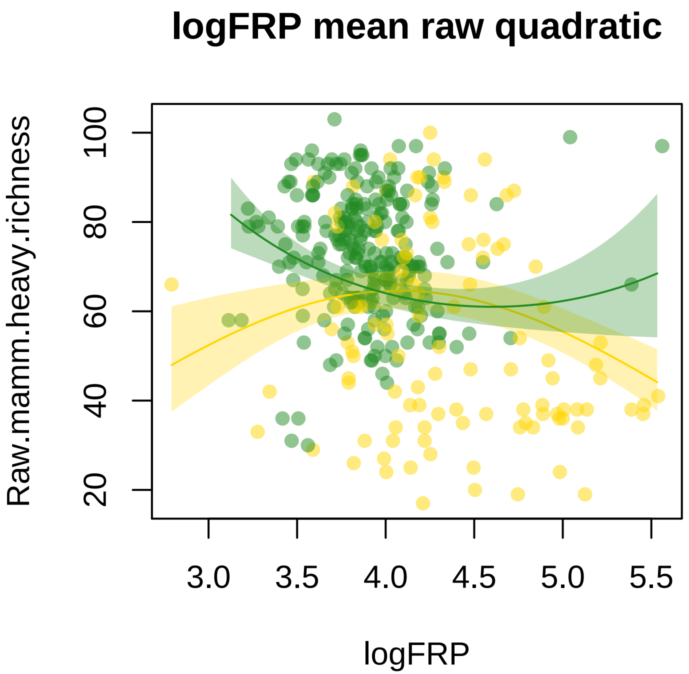
logFRP mean raw quadratic
Raw.mamm.heavy.richness
20
40
60
80
100
3.0
3.5
4.0
4.5
5.0
5.5
logFRP

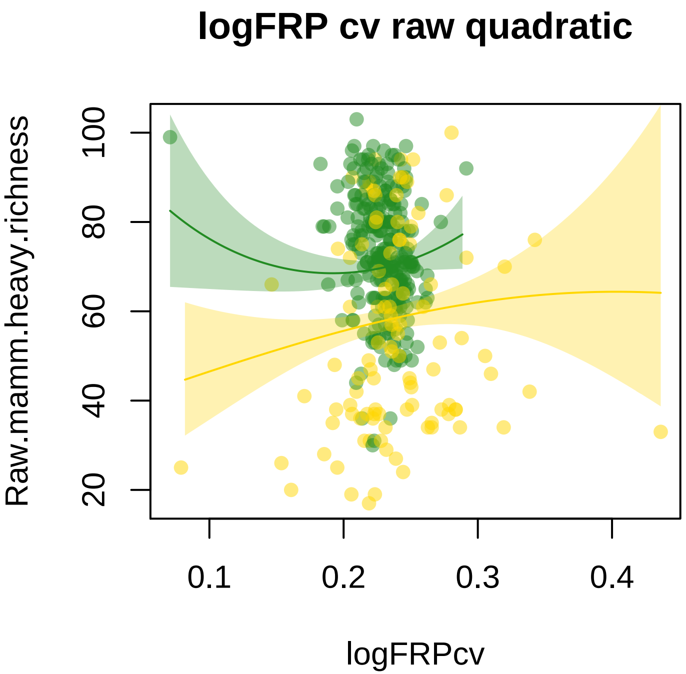
logFRP cv raw quadratic
Raw.mamm.heavy.richness
20
40
60
80
100
0.1
0.2
0.3
0.4
logFRPcv

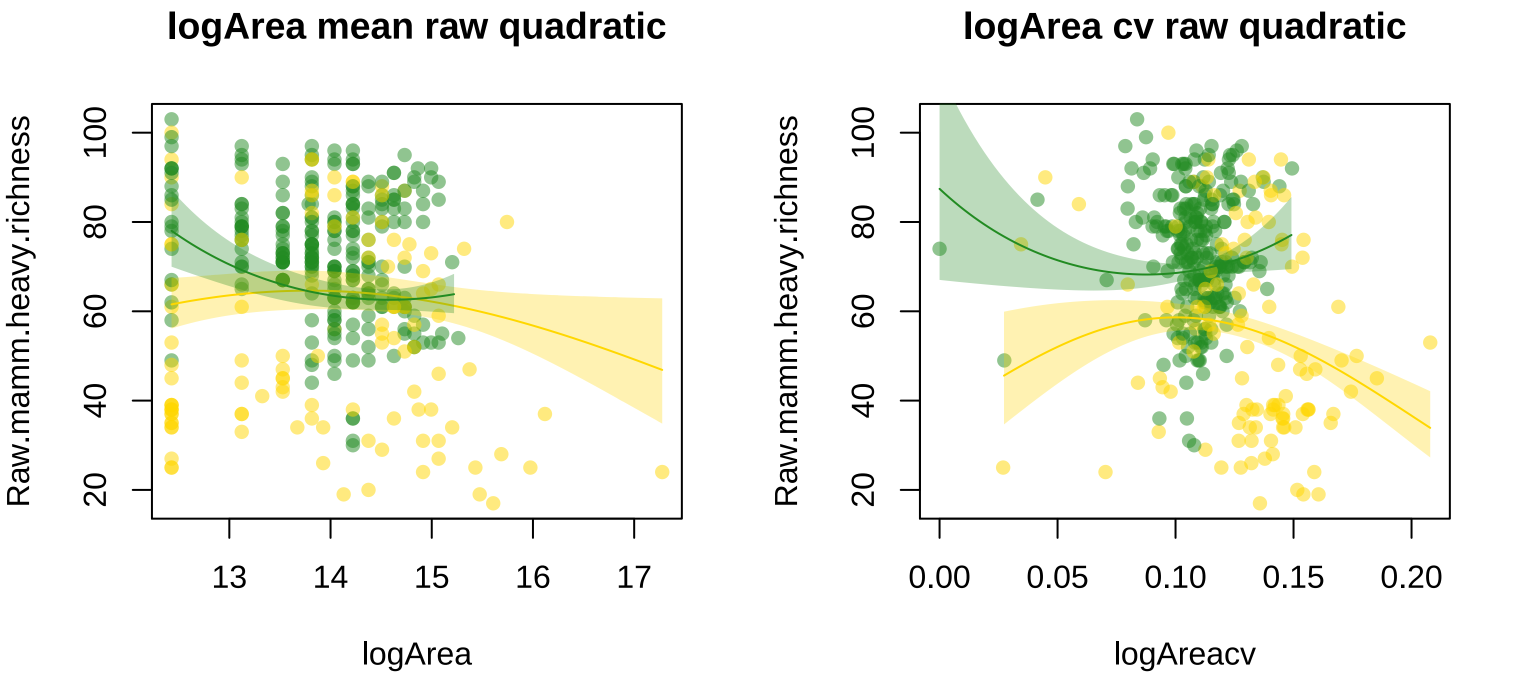

logArea mean raw quadratic
Raw.mamm.heavy.richness
20
40
60
80
100
13
14
15
16
17
logArea
logArea cv raw quadratic
Raw.mamm.heavy.richness
20
40
60
80
100
0.00
0.05
0.10
0.15
0.20
logAreacv

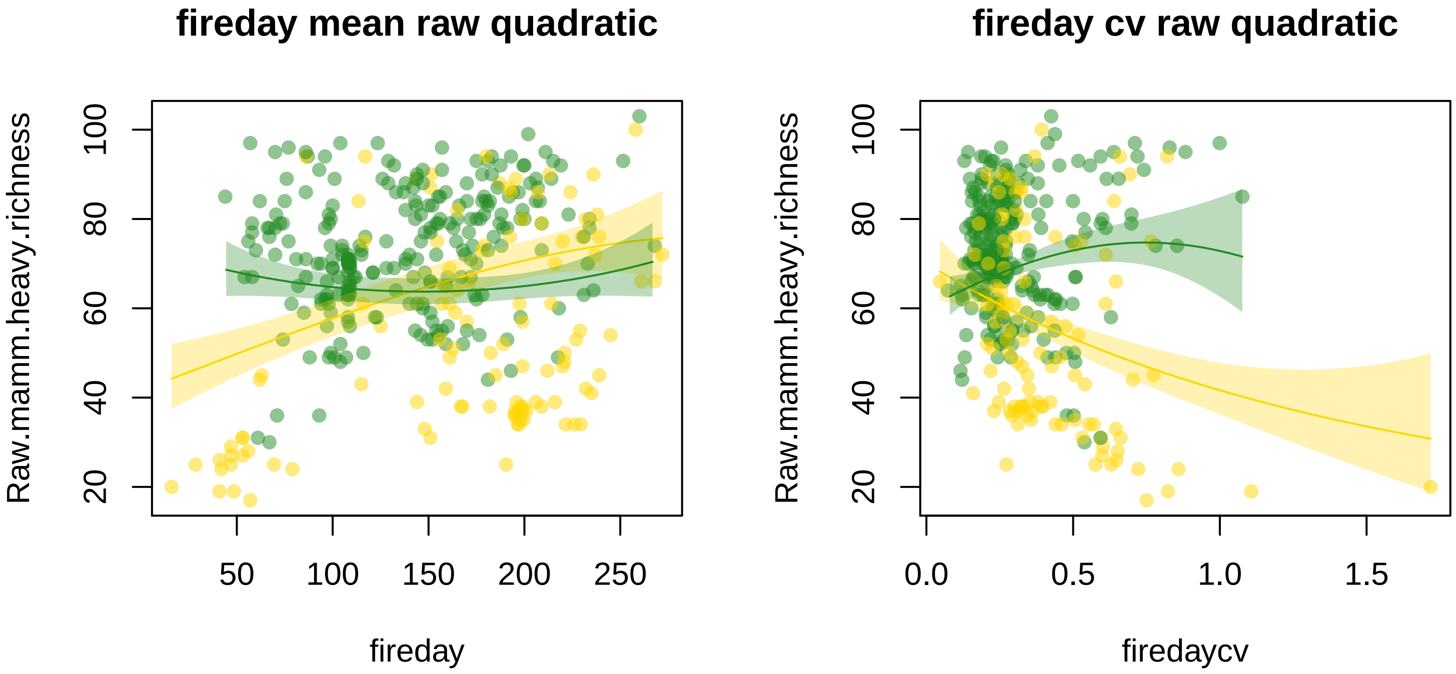
fireday mean raw quadratic
Raw.mamm.heavy.richness
20
40
60
80
100
50
100
150
200
250
fireday
fireday cv raw quadratic
Raw.mamm.heavy.richness
20
40
60
80
100
0.0
0.5
1.0
1.5
firedaycv

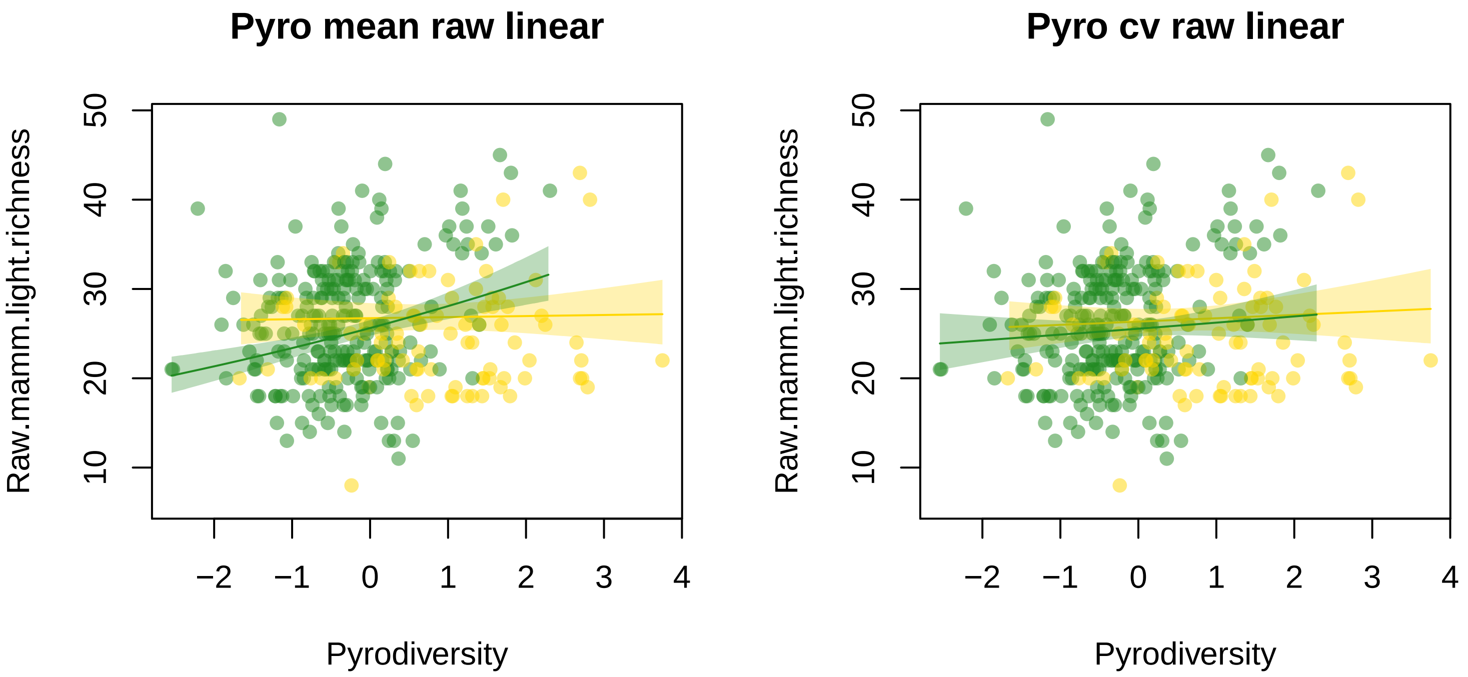

Pyro mean raw linear
Pyro cv raw linear
Raw.mamm.light.richness
Pyrodiversity
10
20
30
40
50
−2
−1
0
1
2
3
4

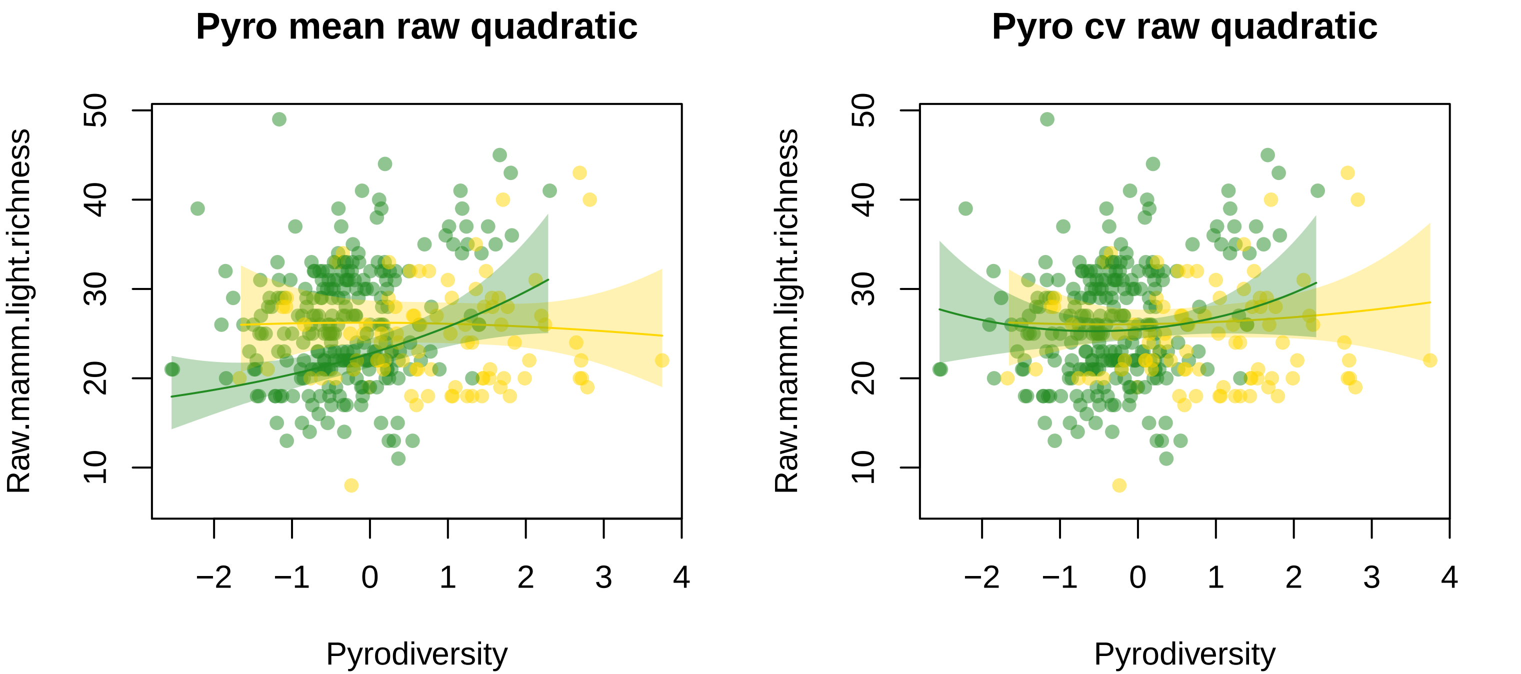

Pyro mean raw quadratic
Raw.mamm.light.richness
10
20
30
40
50
−2
−1
0
1
2
3
4
Pyrodiversity
Pyro cv raw quadratic
Raw.mamm.light.richness
10
20
30
40
50
−2
−1
0
1
2
3
4
Pyrodiversity

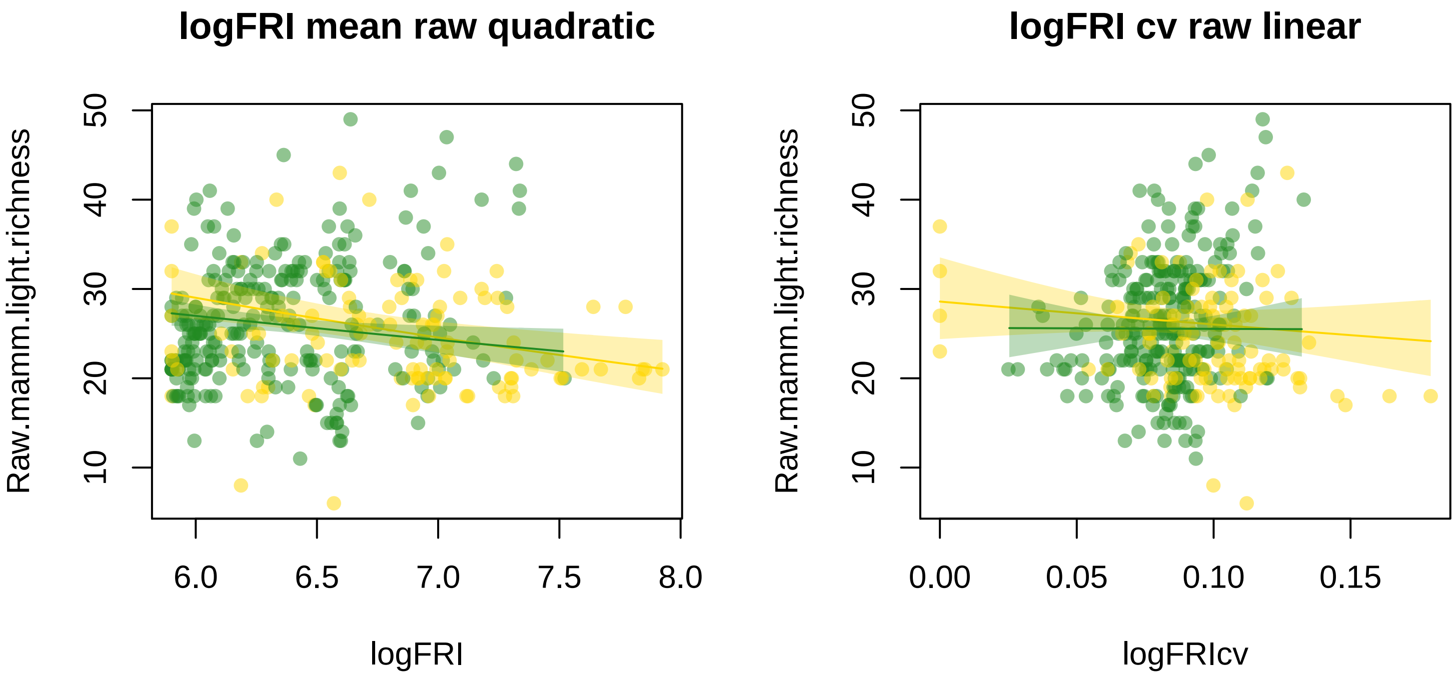
logFRI mean raw quadratic
logFRI cv raw linear
Raw.mamm.light.richness
Raw.mamm.light.richness
10
20
30
40
50
6.0
6.5
7.0
7.5
8.0
0.00
0.05
0.10
0.15
logFRI
logFRIcv

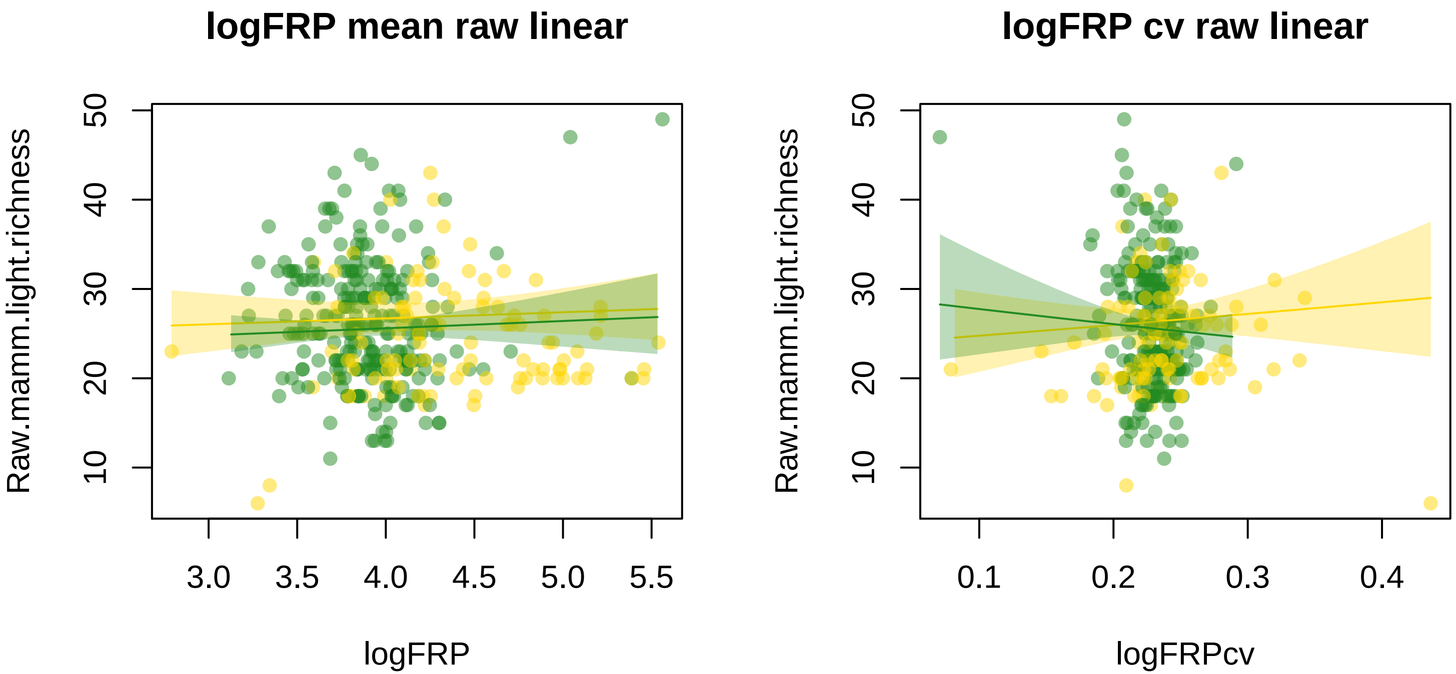
logFRP mean raw linear
logFRP cv raw linear
Raw.mamm.light.richness
Raw.mamm.light.richness
10
20
30
40
50
3.0
3.5
4.0
4.5
5.0
5.5
0.1
0.2
0.3
0.4
logFRP
logFRPcv

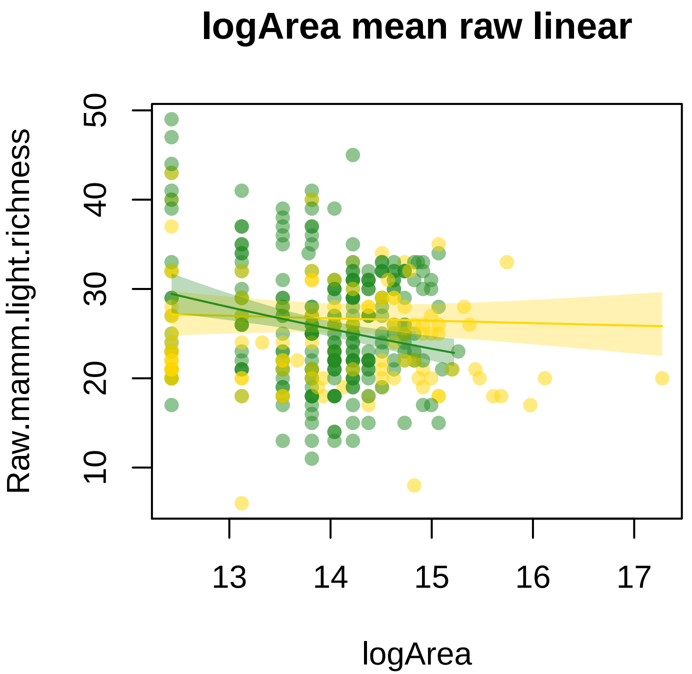
logArea mean raw linear
Raw.mamm.light.richness
10
20
30
40
50
13
14
15
16
17
logArea

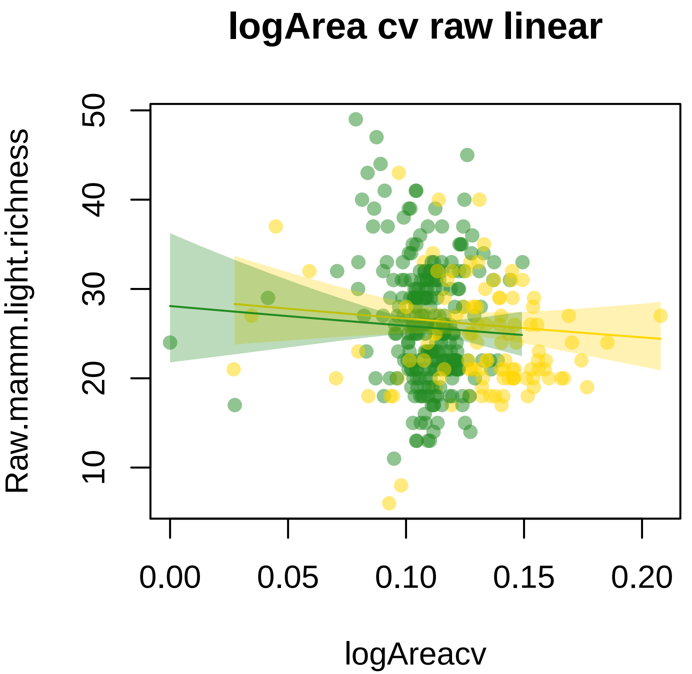
logArea cv raw linear
Raw.mamm.light.richness
10
20
30
40
50
0.00
0.05
0.10
0.15
0.20
logAreacv

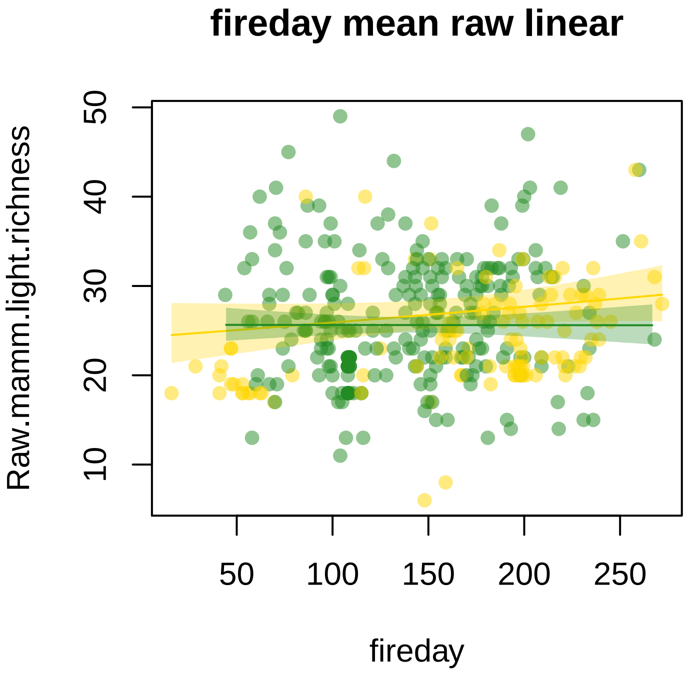

fireday mean raw linear
Raw.mamm.light.richness
10
20
30
40
50
50
100
150
200
250
fireday

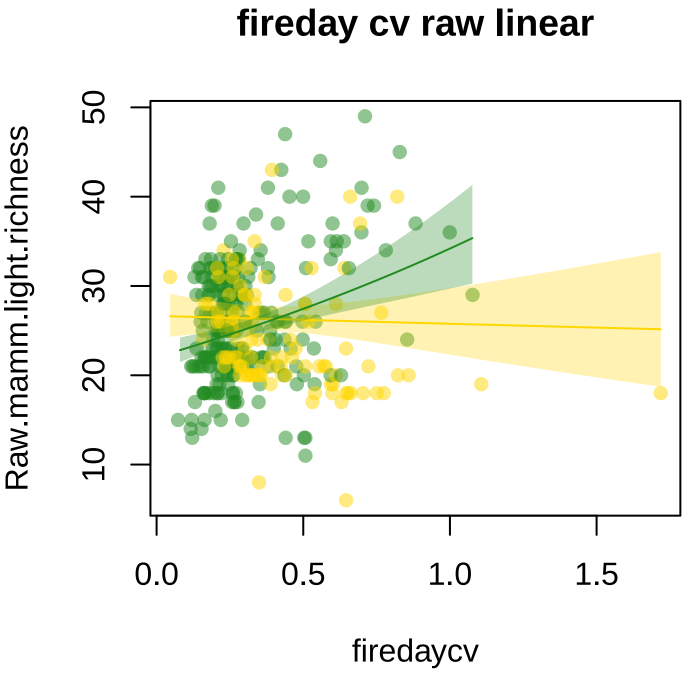

fireday cv raw linear
Raw.mamm.light.richness
10
20
30
40
50
0.0
0.5
1.0
1.5
firedaycv

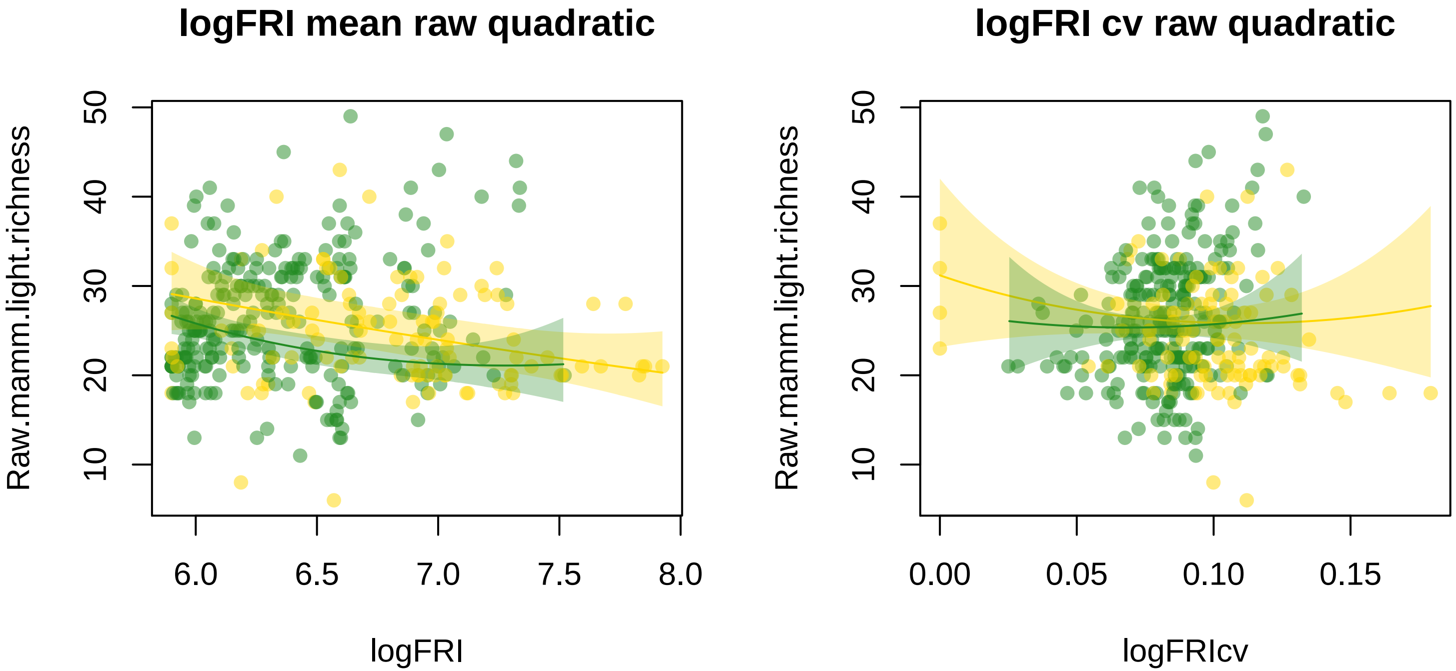

logFRI mean raw quadratic
Raw.mamm.light.richness
10
20
30
40
50
6.0
6.5
7.0
7.5
8.0
logFRI
logFRI cv raw quadratic
Raw.mamm.light.richness
10
20
30
40
50
0.00
0.05
0.10
0.15
logFRIcv

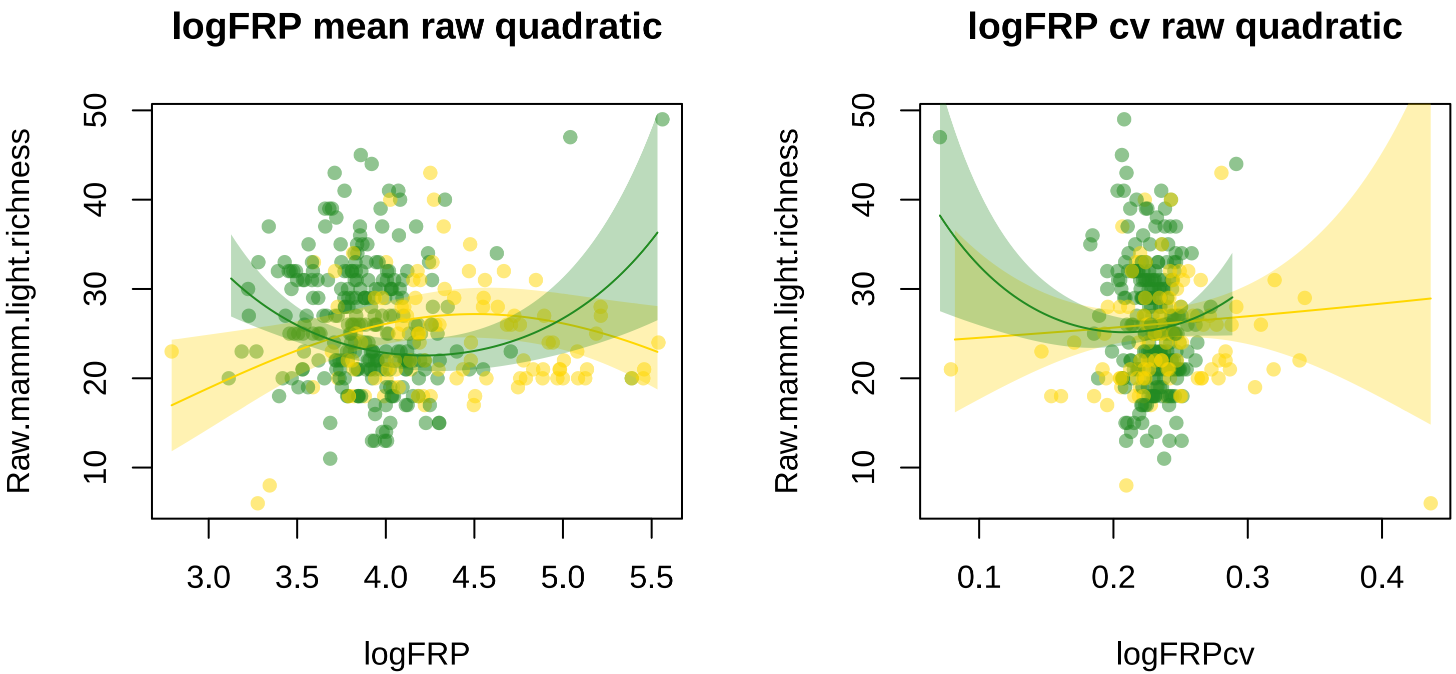
logFRP mean raw quadratic
Raw.mamm.light.richness
10
20
30
40
50
3.0
3.5
4.0
4.5
5.0
5.5
logFRP
logFRP cv raw quadratic
Raw.mamm.light.richness
10
20
30
40
50
0.1
0.2
0.3
0.4
logFRPcv

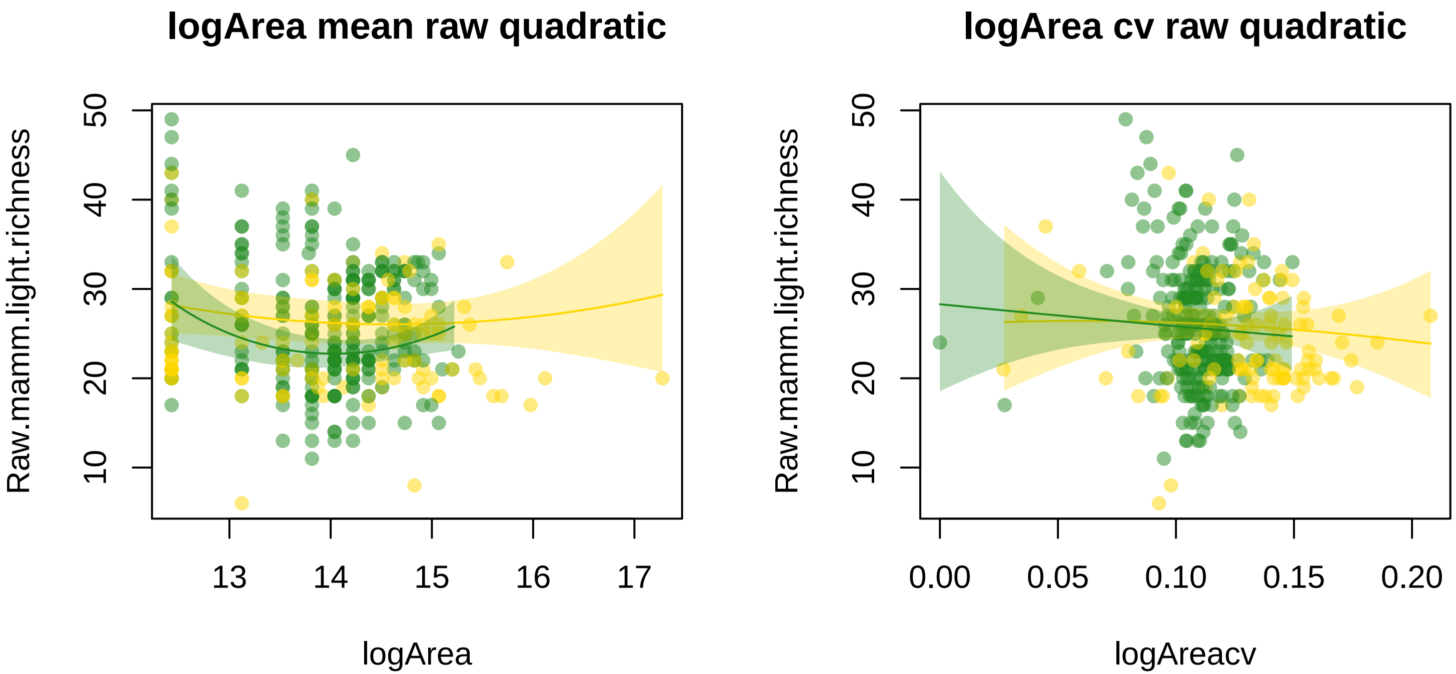

logArea mean raw quadratic
Raw.mamm.light.richness
10
20
30
40
50
13
14
15
16
17
logArea
logArea cv raw quadratic
Raw.mamm.light.richness
10
20
30
40
50
0.00
0.05
0.10
0.15
0.20
logAreacv

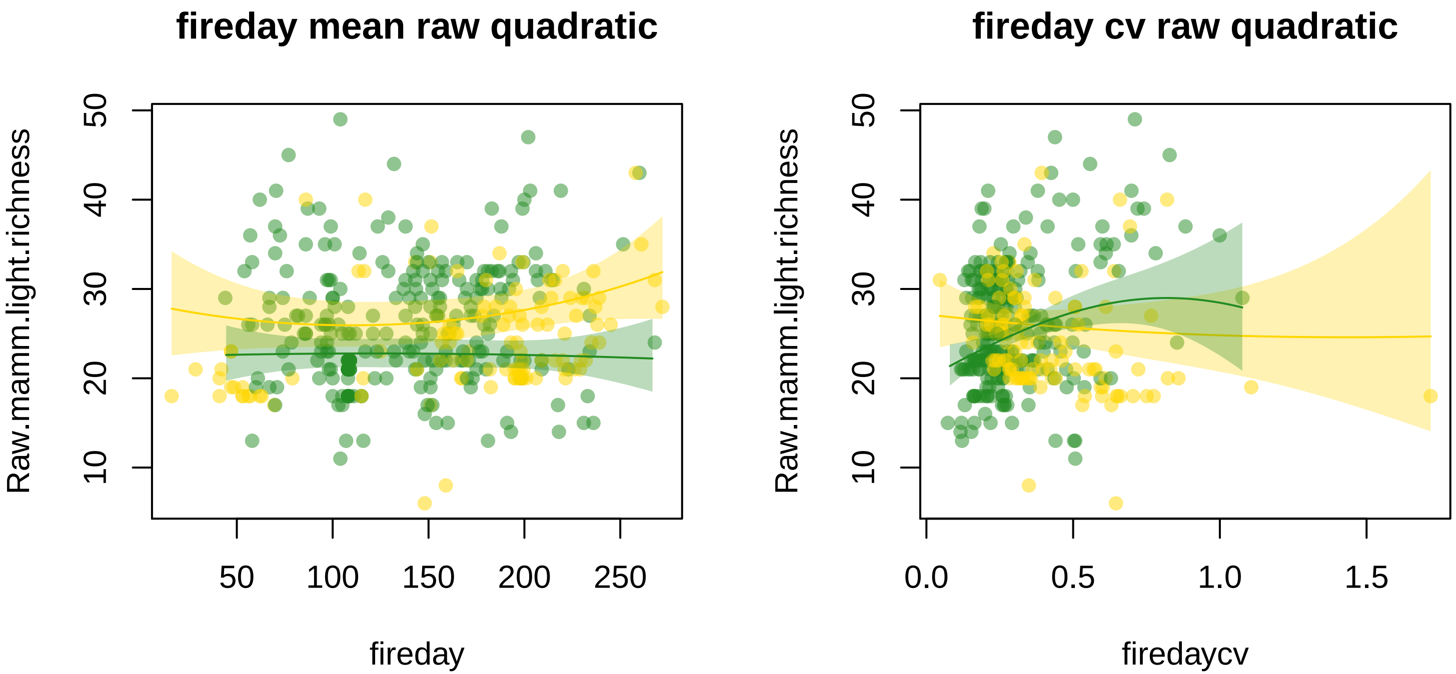
fireday mean raw quadratic
Raw.mamm.light.richness
10
20
30
40
50
50
100
150
200
250
fireday
fireday cv raw quadratic
Raw.mamm.light.richness
10
20
30
40
50
0.0
0.5
1.0
1.5
firedaycv

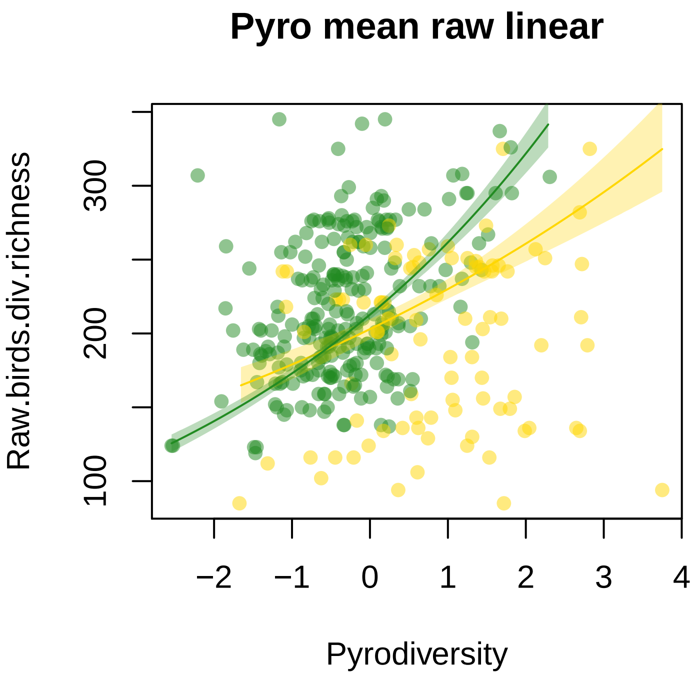
Pyro mean raw linear
Raw.birds.div.richness
100
200
300
−2
−1
0
1
2
3
4
Pyrodiversity

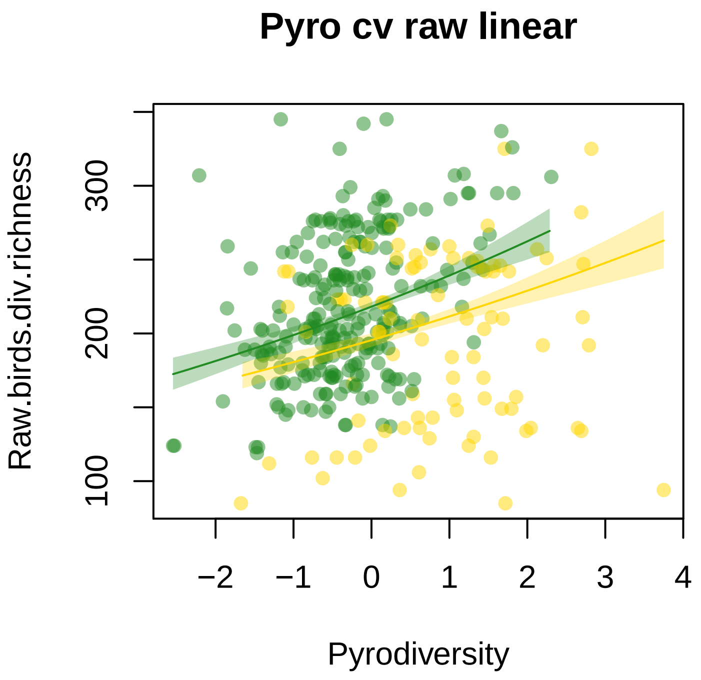
Pyro cv raw linear
Raw.birds.div.richness
100
200
300
−2
−1
0
1
2
3
4
Pyrodiversity

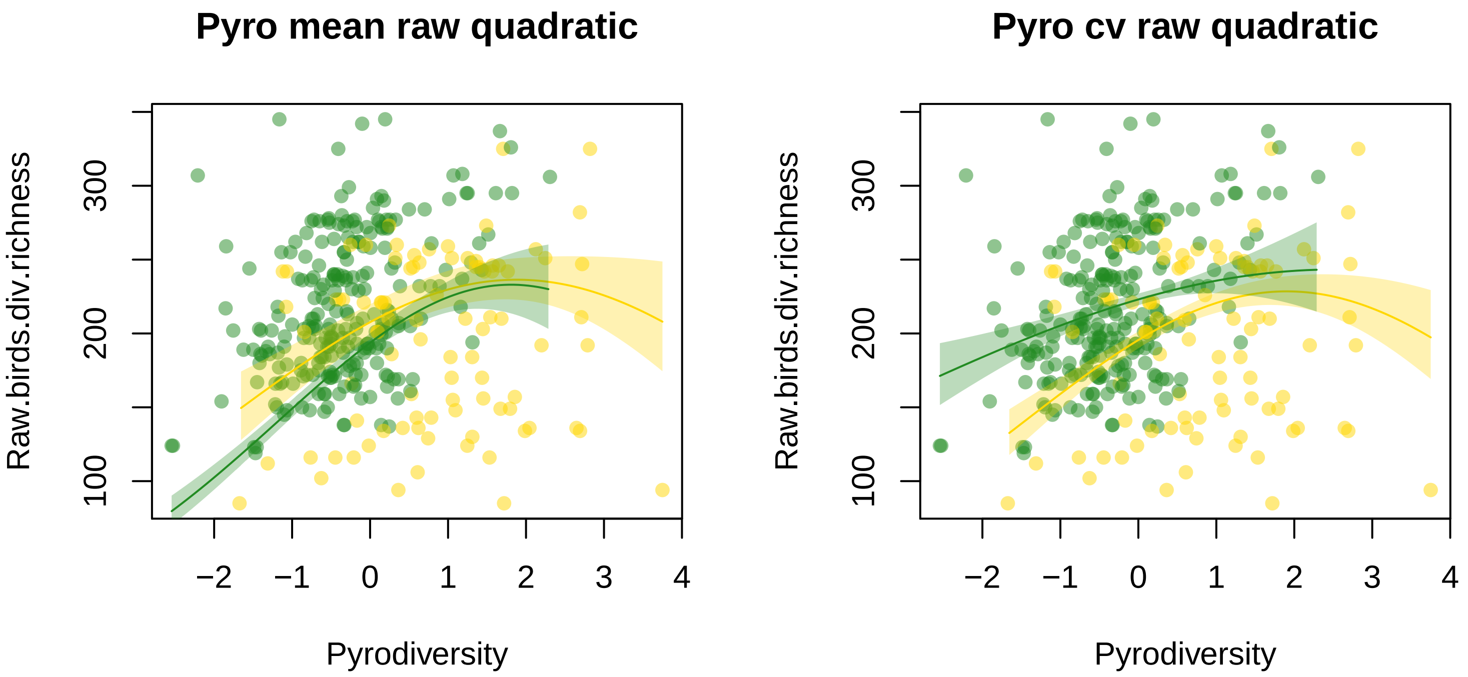

Pyro mean raw quadratic
Pyro cv raw quadratic
Raw.birds.div.richness
100
200
300
−2
−1
0
1
2
3
4
Pyrodiversity

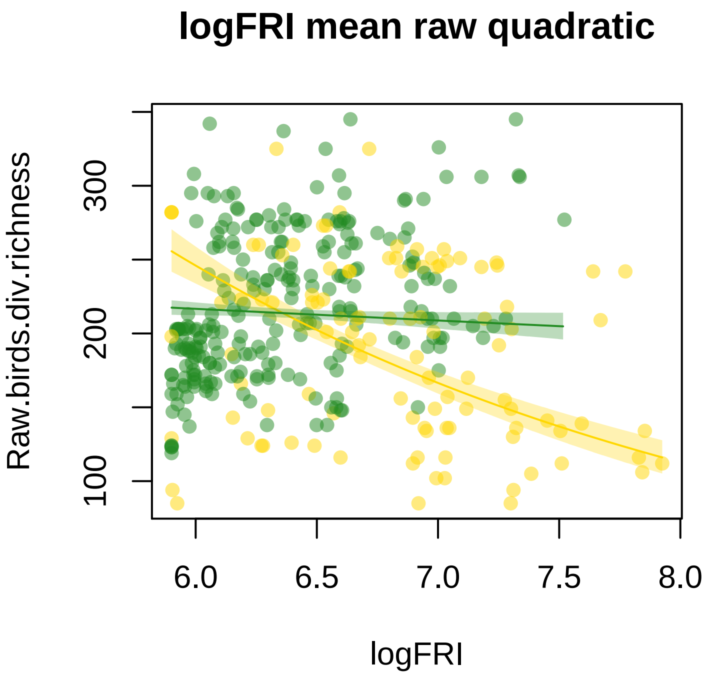
logFRI mean raw quadratic
Raw.birds.div.richness
100
200
300
6.0
6.5
7.0
7.5
8.0
logFRI

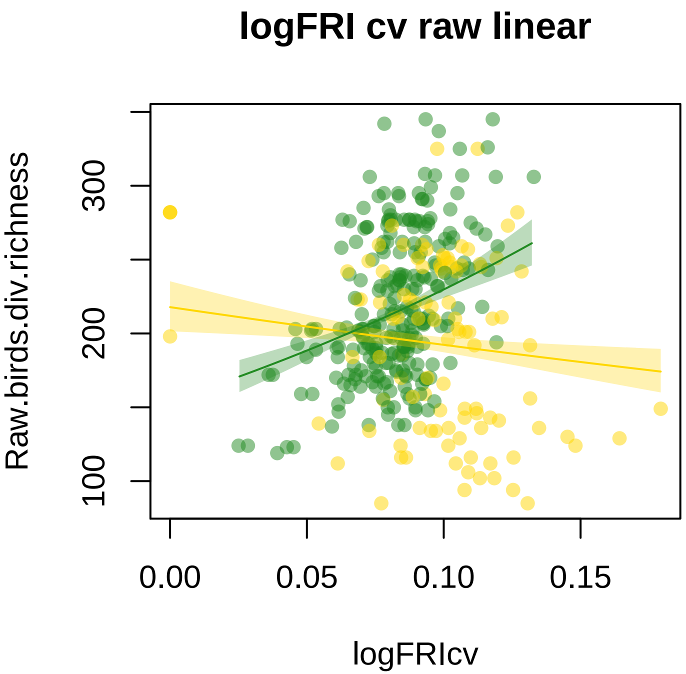
logFRI cv raw linear
Raw.birds.div.richness
100
200
300
0.00
0.05
0.10
0.15
logFRIcv

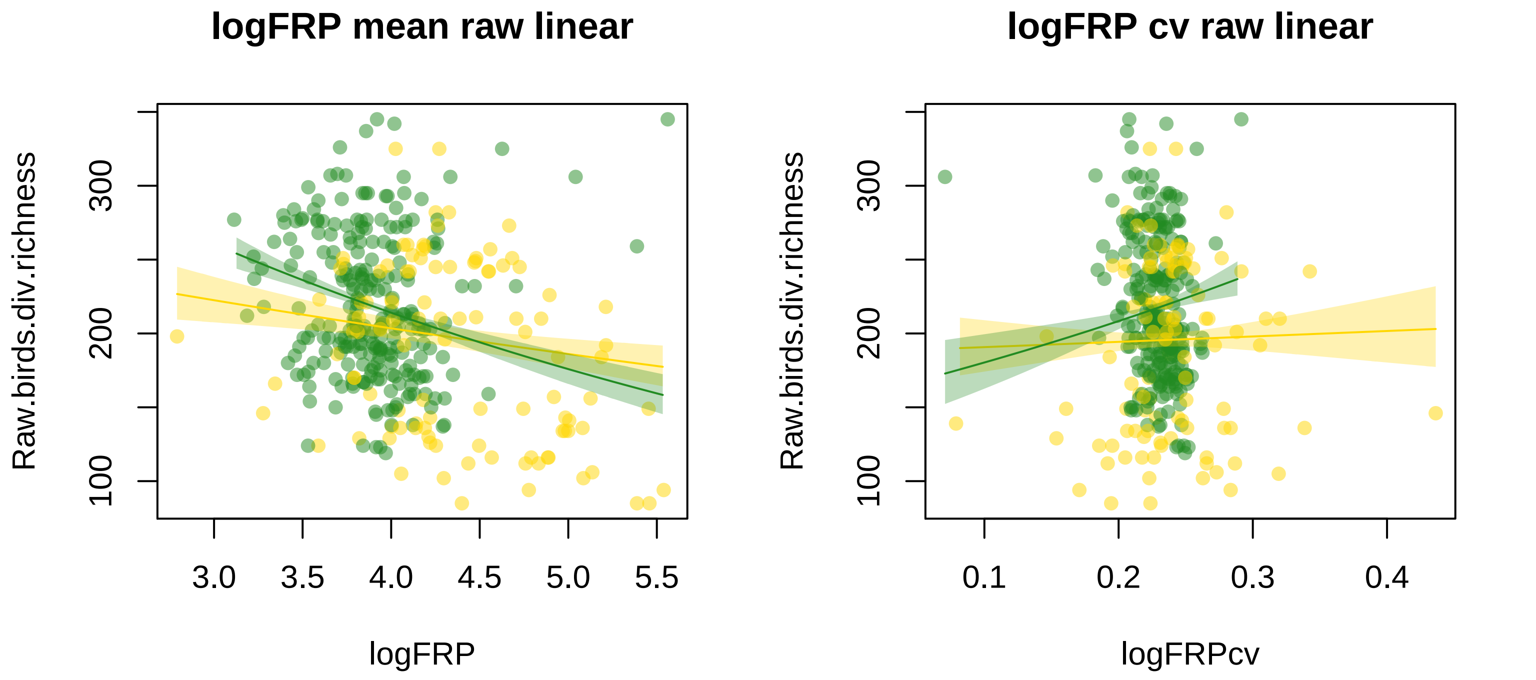
logFRP mean raw linear
logFRP cv raw linear
Raw.birds.div.richness
100
200
300
3.0
3.5
4.0
4.5
5.0
5.5
logFRP
0.1
0.2
0.3
0.4
logFRPcv

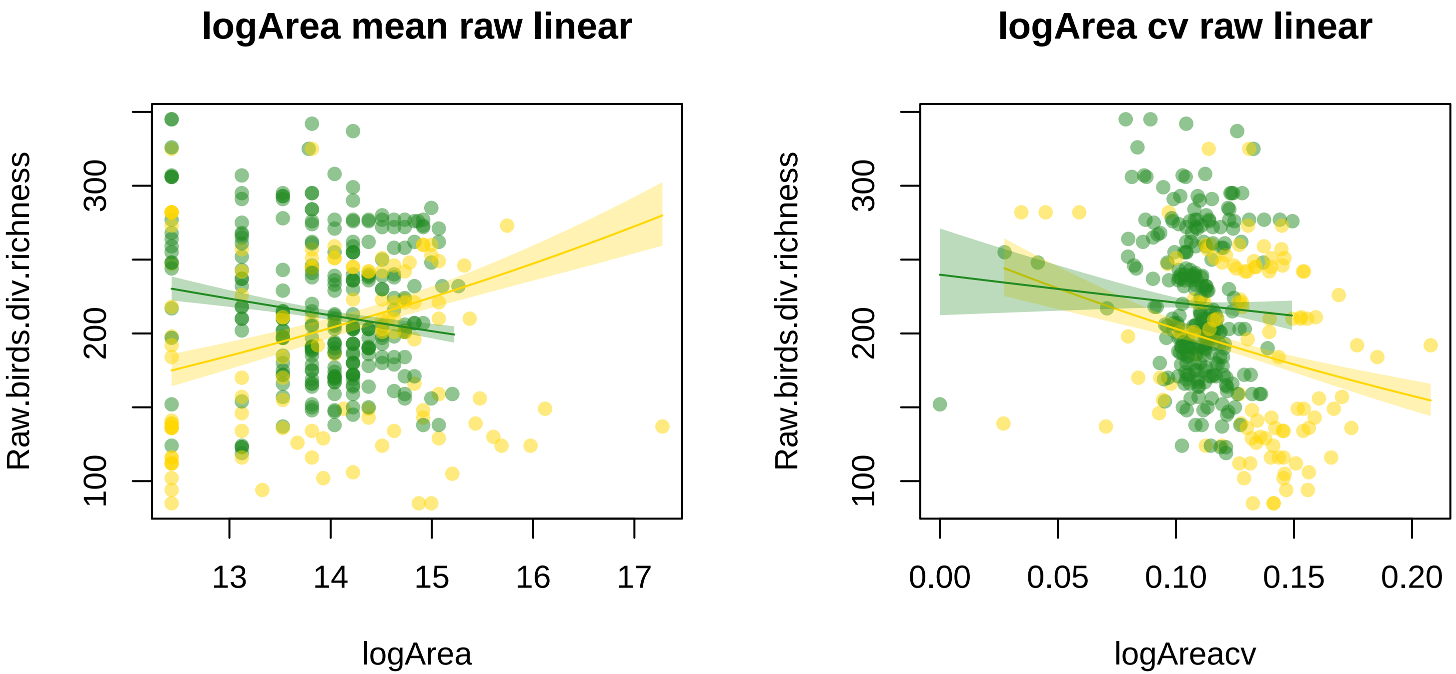
logArea mean raw linear
Raw.birds.div.richness
100
200
300
13
14
15
16
17
logArea
logArea cv raw linear
Raw.birds.div.richness
100
200
300
0.00
0.05
0.10
0.15
0.20
logAreacv

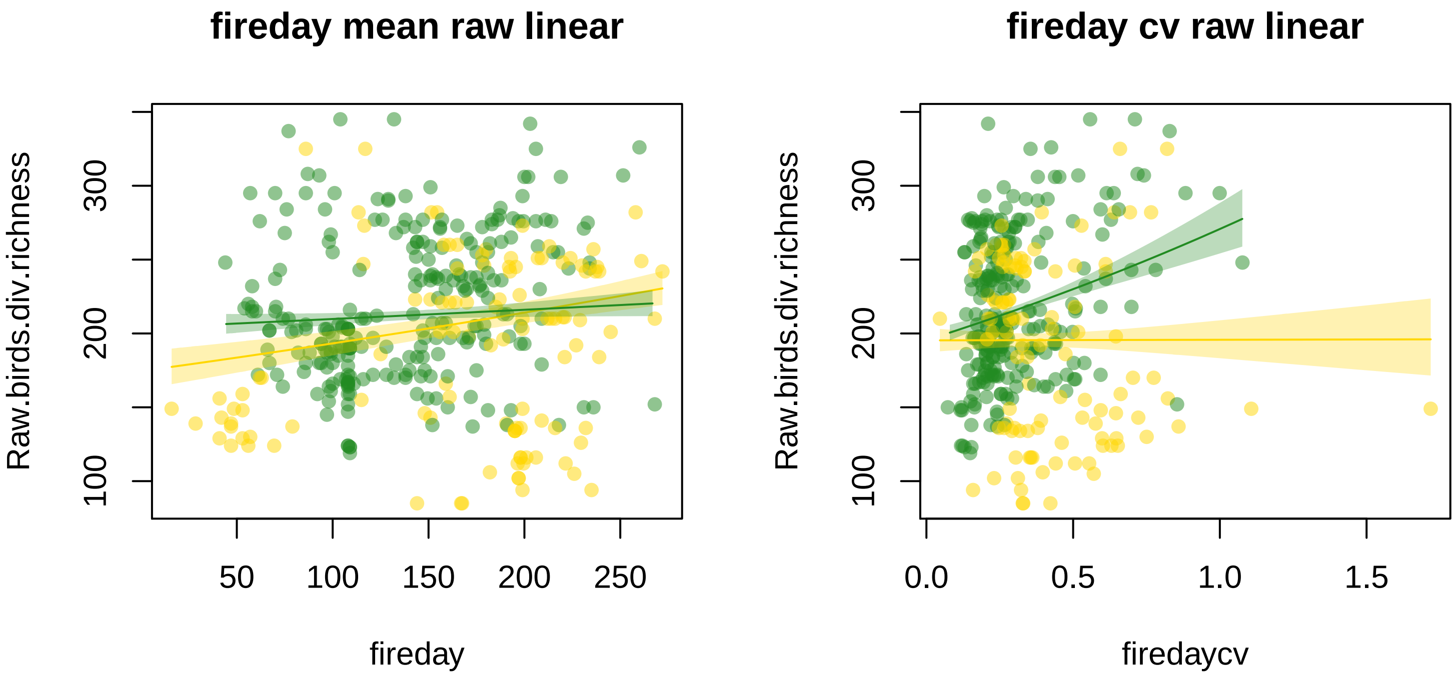
fireday mean raw linear
Raw.birds.div.richness
100
200
300
50
100
150
200
250
fireday
fireday cv raw linear
Raw.birds.div.richness
100
200
300
0.0
0.5
1.0
1.5
firedaycv

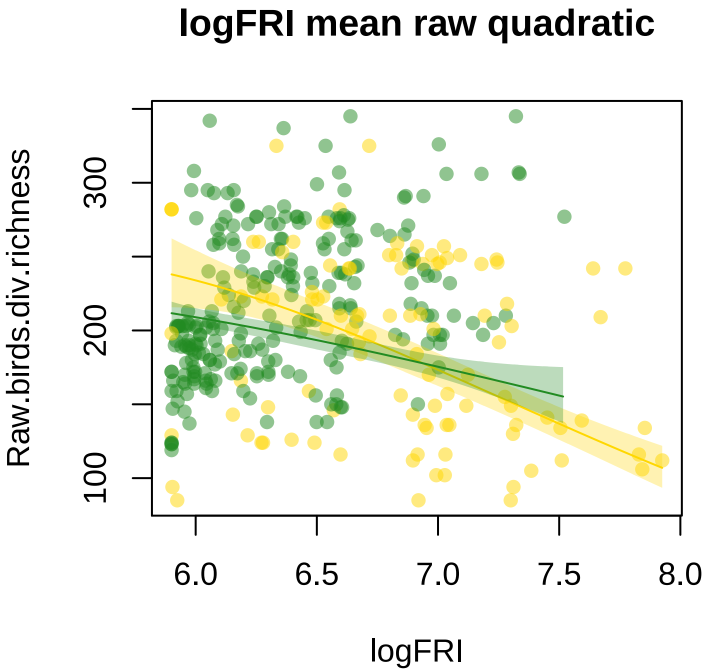

logFRI mean raw quadratic
Raw.birds.div.richness
300
200
100
6.0
6.5
7.0
7.5
8.0
logFRI

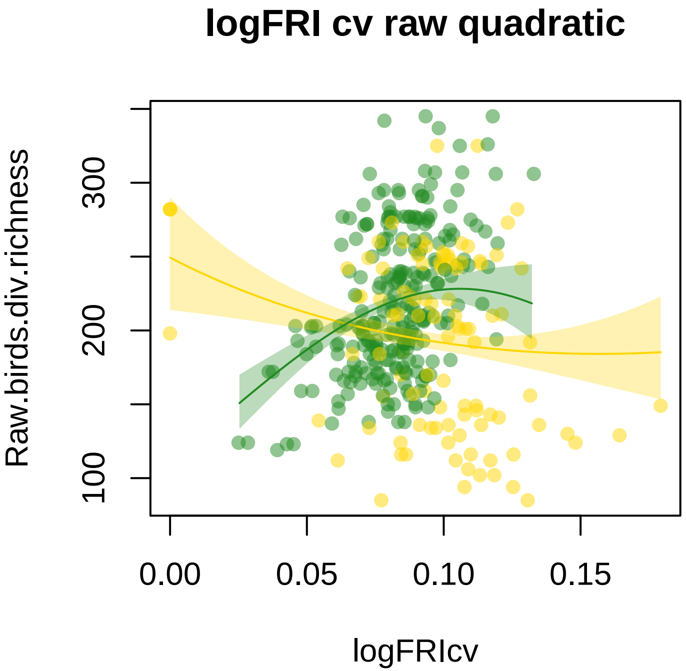

logFRI cv raw quadratic
Raw.birds.div.richness
300
200
100
0.00
0.05
0.10
0.15
logFRIcv

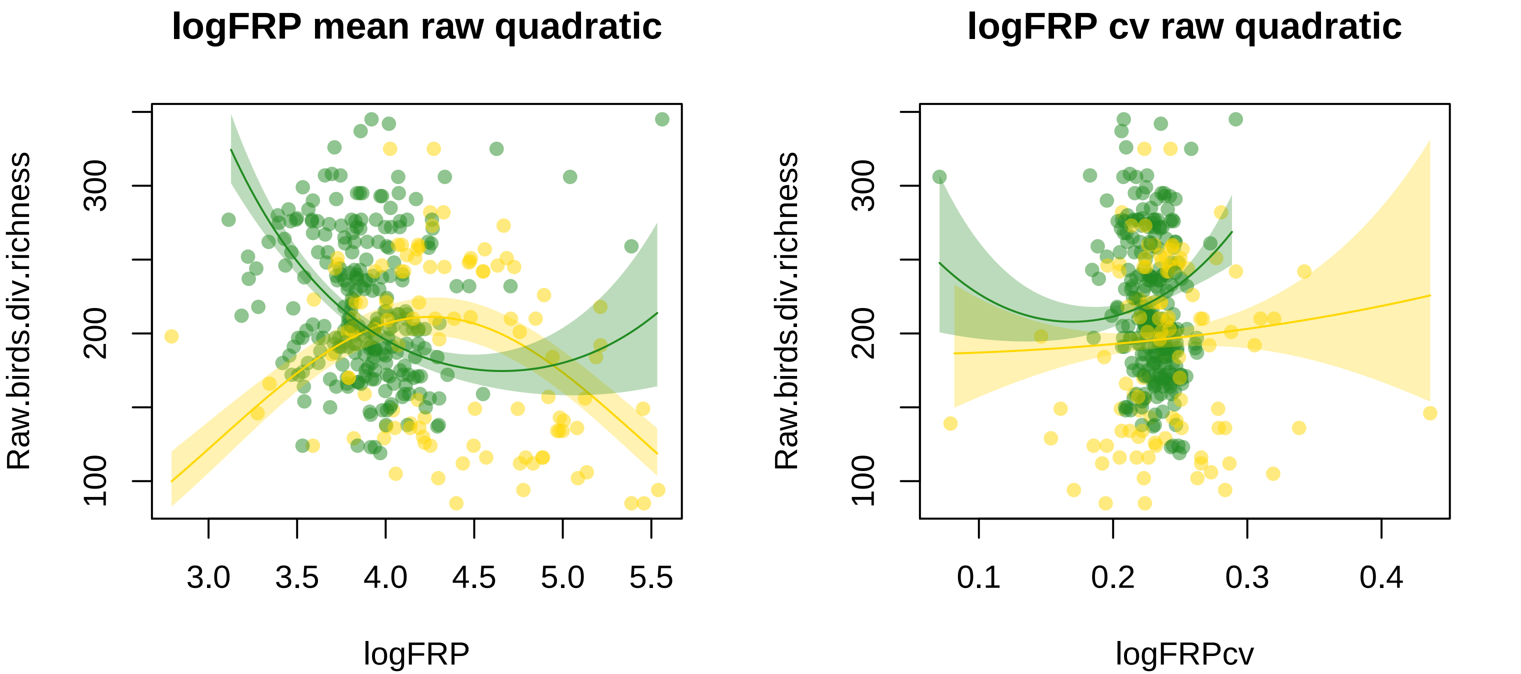

logFRP mean raw quadratic
Raw.birds.div.richness
100
200
300
3.0
3.5
4.0
4.5
5.0
5.5
logFRP
logFRP cv raw quadratic
Raw.birds.div.richness
100
200
300
0.1
0.2
0.3
0.4
logFRPcv

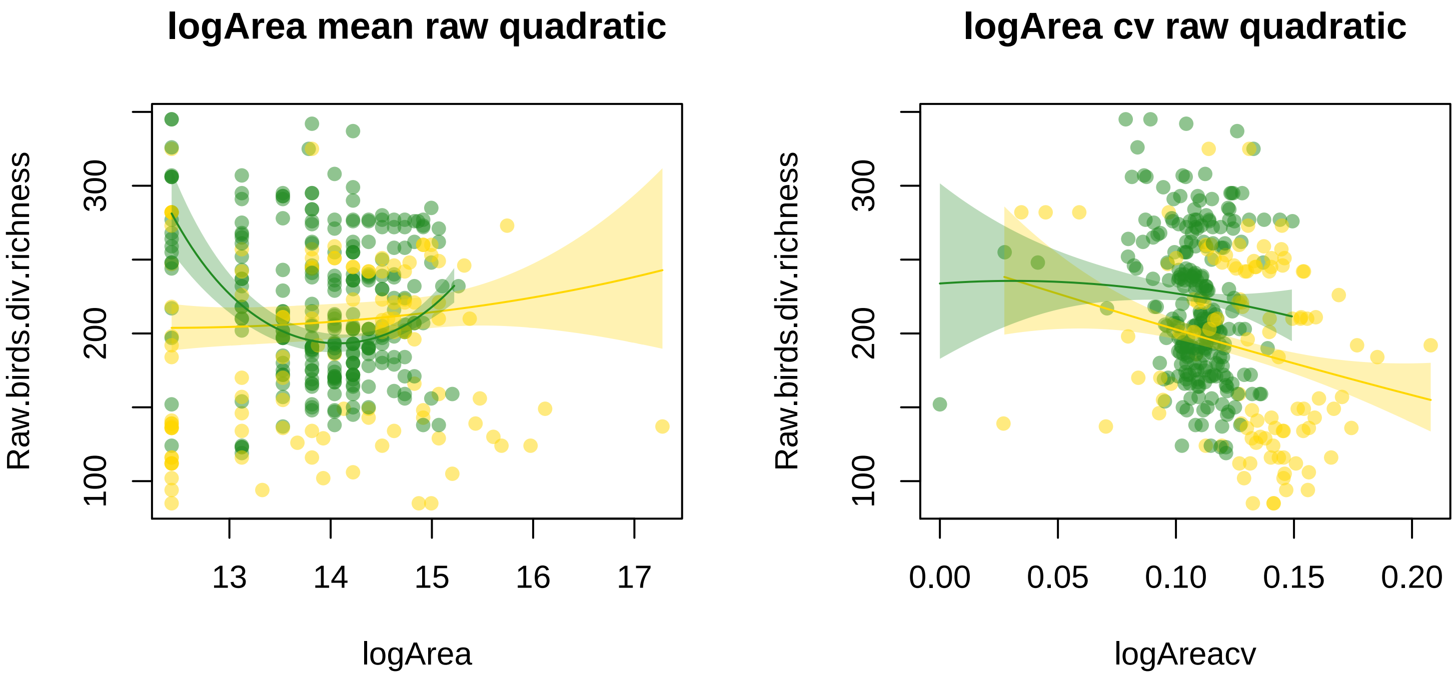
logArea mean raw quadratic
Raw.birds.div.richness
100
200
300
13
14
15
16
17
logArea
logArea cv raw quadratic
Raw.birds.div.richness
100
200
300
0.00
0.05
0.10
0.15
0.20
logAreacv

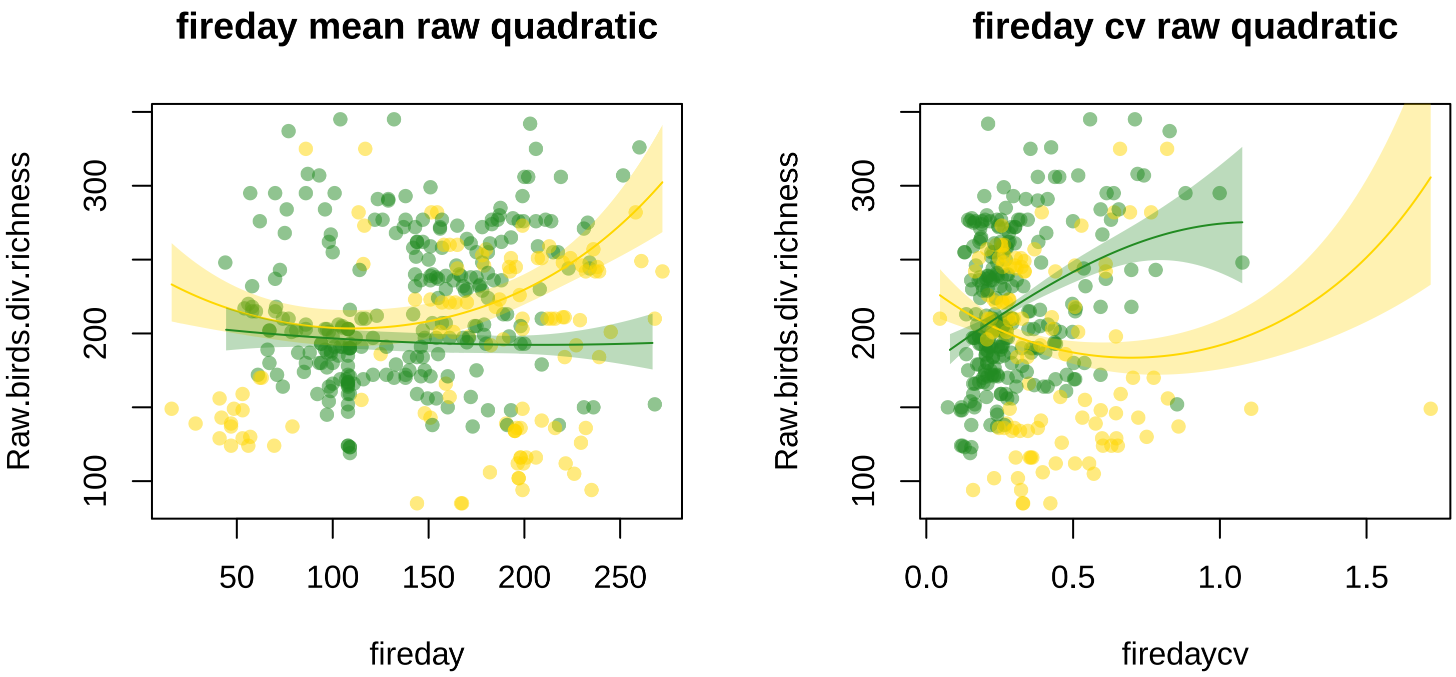

fireday mean raw quadratic
Raw.birds.div.richness
100
200
300
50
100
150
200
250
fireday
fireday cv raw quadratic
Raw.birds.div.richness
100
200
300
0.0
0.5
1.0
1.5
firedaycv

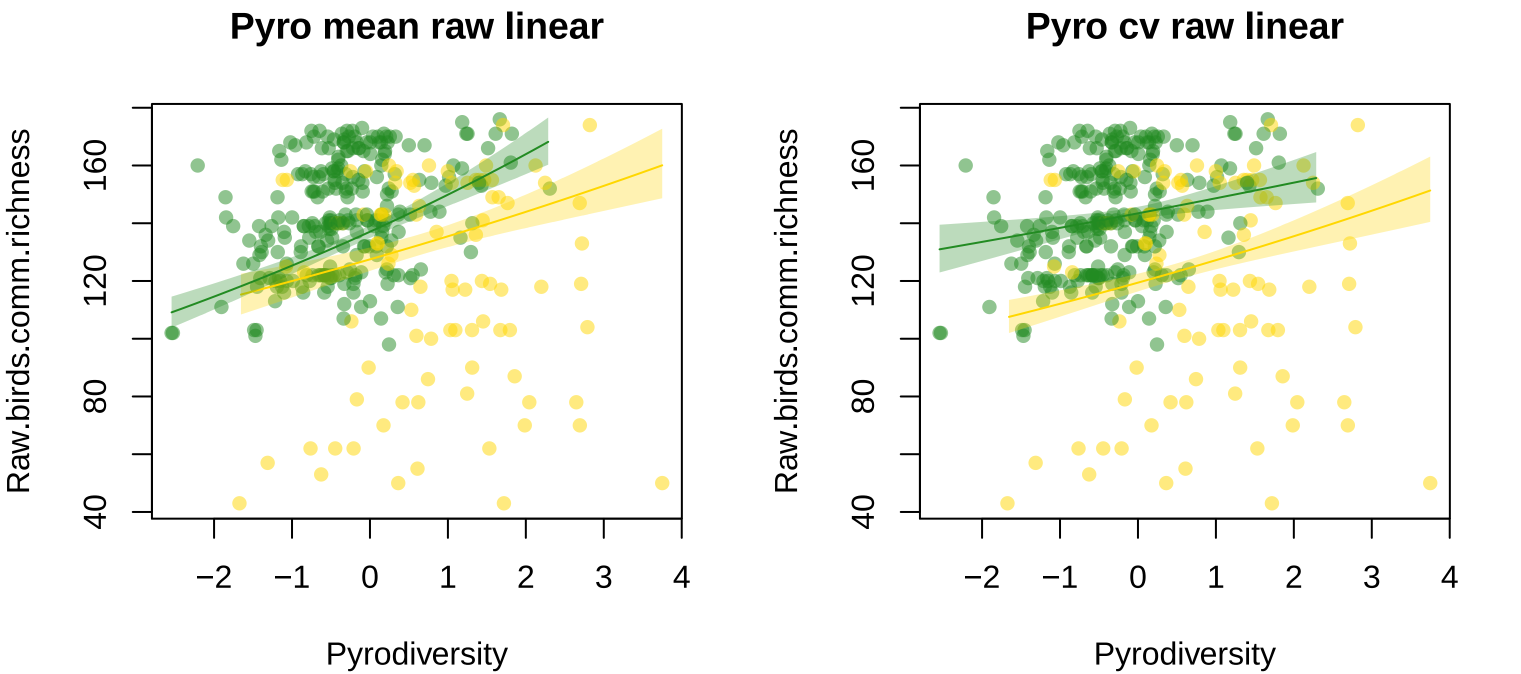
Pyro mean raw linear
Pyro cv raw linear
Raw.birds.comm.richness
40
80
120
160
−2
−1
0
1
2
3
4
Pyrodiversity

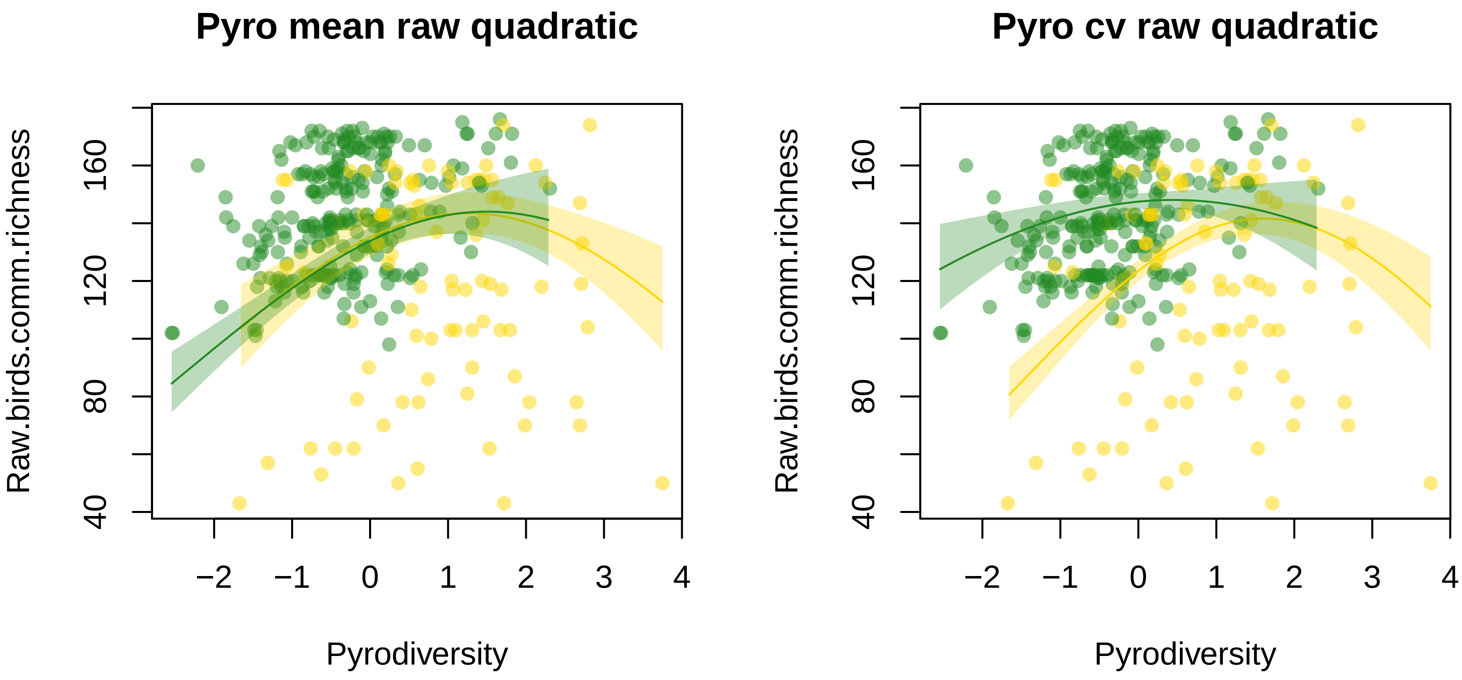

Pyro mean raw quadratic
Pyro cv raw quadratic
Raw.birds.comm.richness
40
80
120
160
−2
−1
0
1
2
3
4
Pyrodiversity

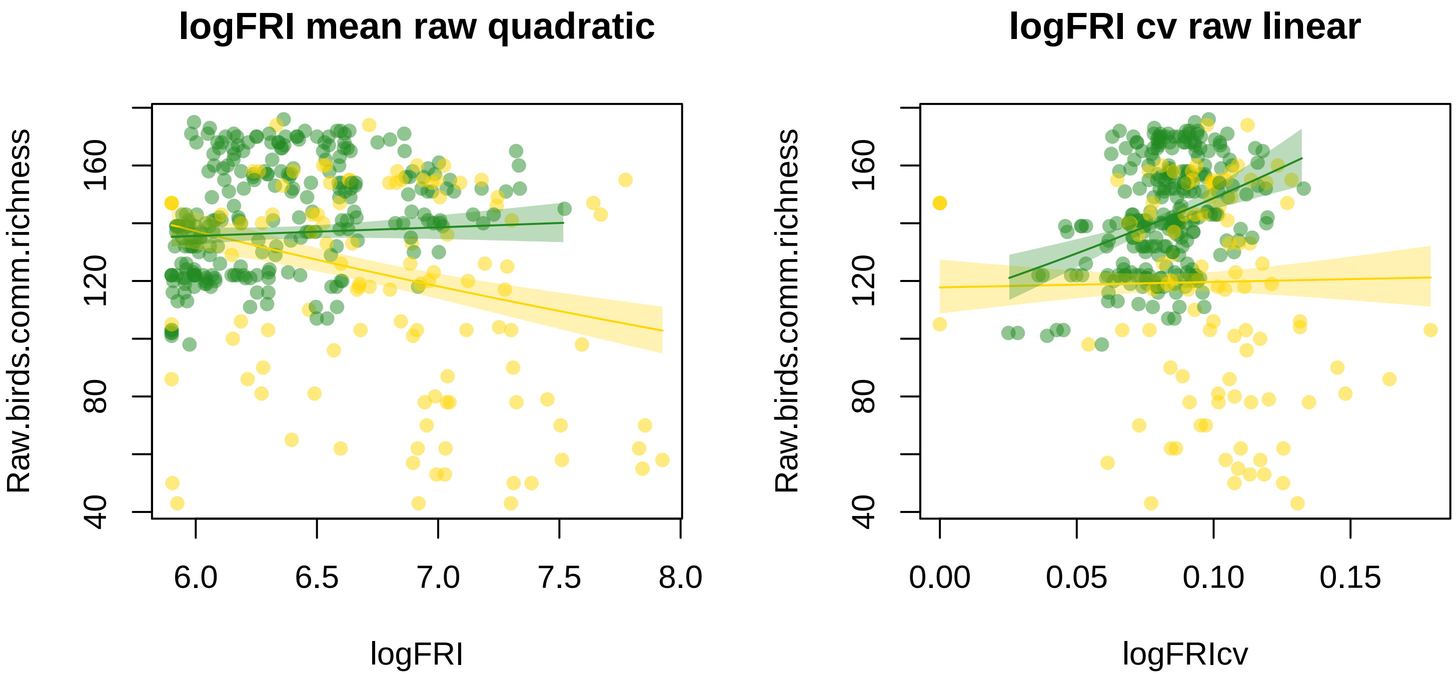
logFRI mean raw quadratic
Raw.birds.comm.richness
40
80
120
160
6.0
6.5
7.0
7.5
8.0
logFRI
logFRI cv raw linear
Raw.birds.comm.richness
40
80
120
160
0.00
0.05
0.10
0.15
logFRIcv

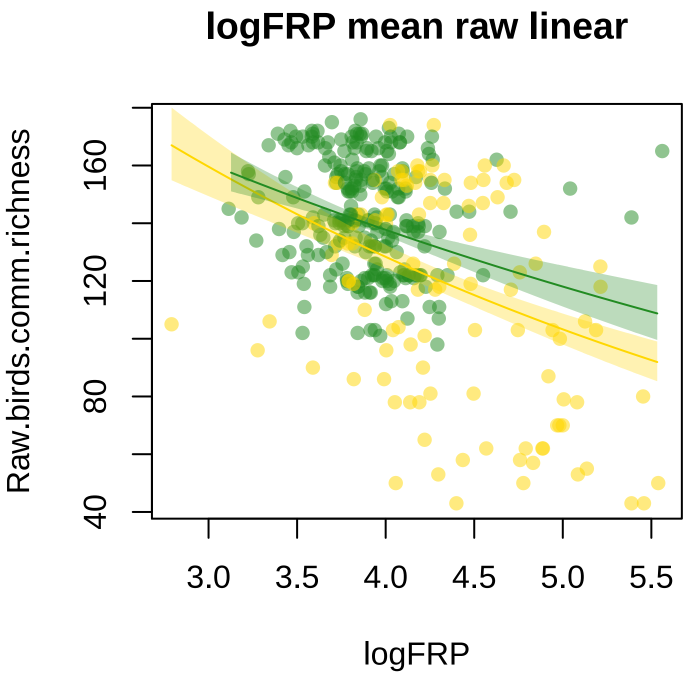

logFRP mean raw linear
Raw.birds.comm.richness
40
80
120
160
3.0
3.5
4.0
4.5
5.0
5.5
logFRP

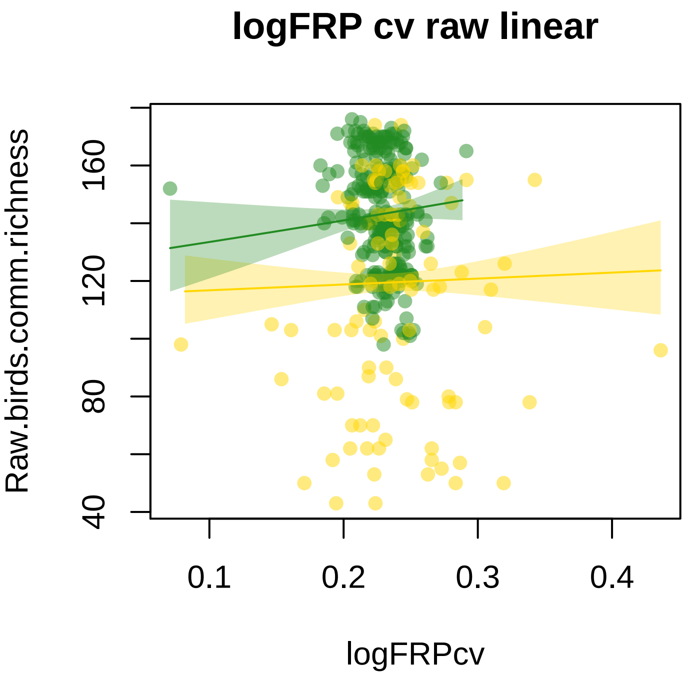

logFRP cv raw linear
Raw.birds.comm.richness
40
80
120
160
0.1
0.2
0.3
0.4
logFRPcv

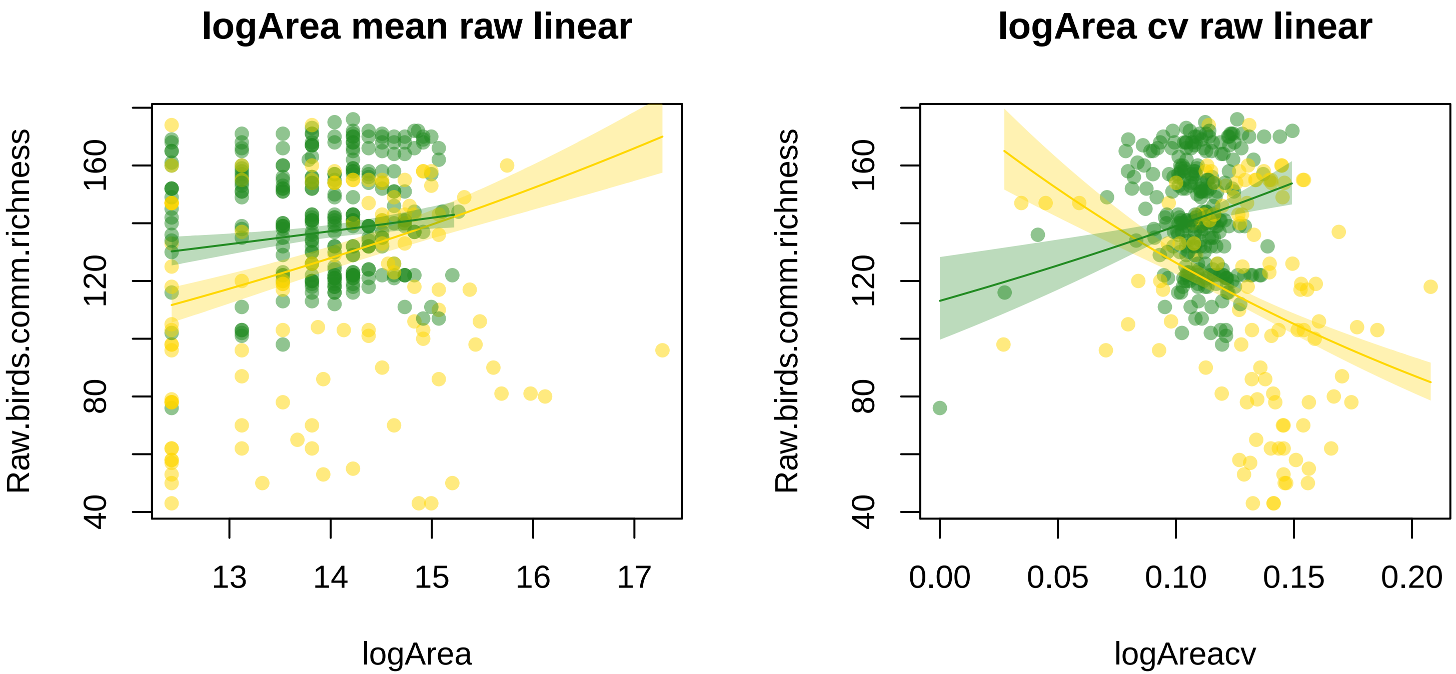
logArea mean raw linear
Raw.birds.comm.richness
40
80
120
160
13
14
15
16
17
logArea
logArea cv raw linear
Raw.birds.comm.richness
40
80
120
160
0.00
0.05
0.10
0.15
0.20
logAreacv

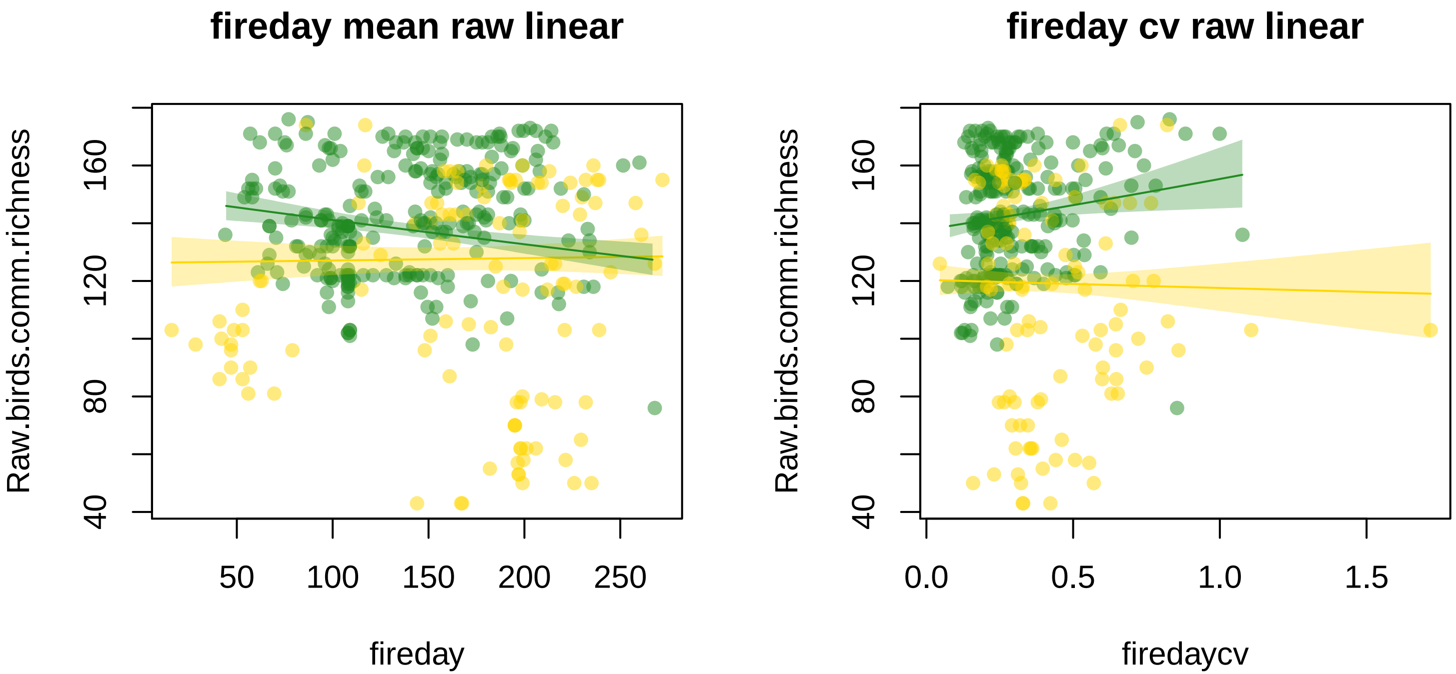
fireday mean raw linear
Raw.birds.comm.richness
40
80
120
160
50
100
150
200
250
fireday
fireday cv raw linear
Raw.birds.comm.richness
40
80
120
160
0.0
0.5
1.0
1.5
firedaycv

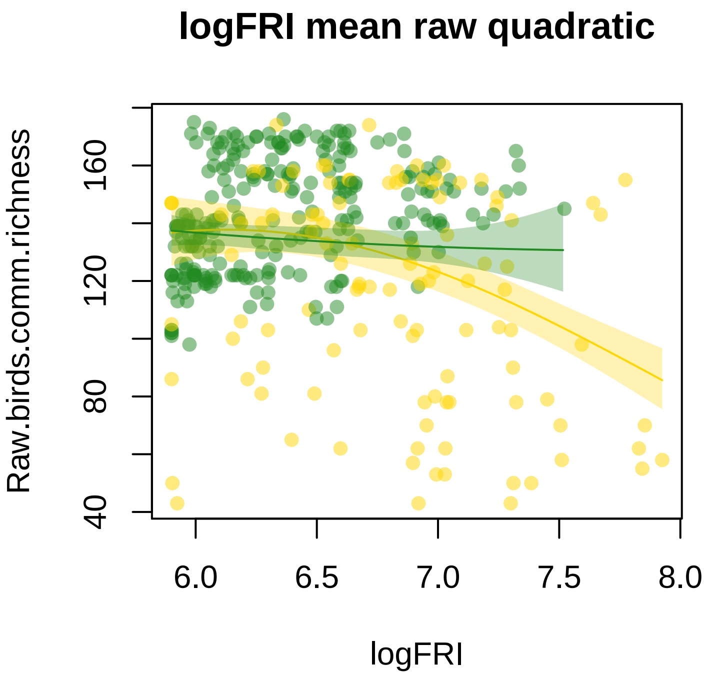

logFRI mean raw quadratic
Raw.birds.comm.richness
40
80
120
160
6.0
6.5
7.0
7.5
8.0
logFRI

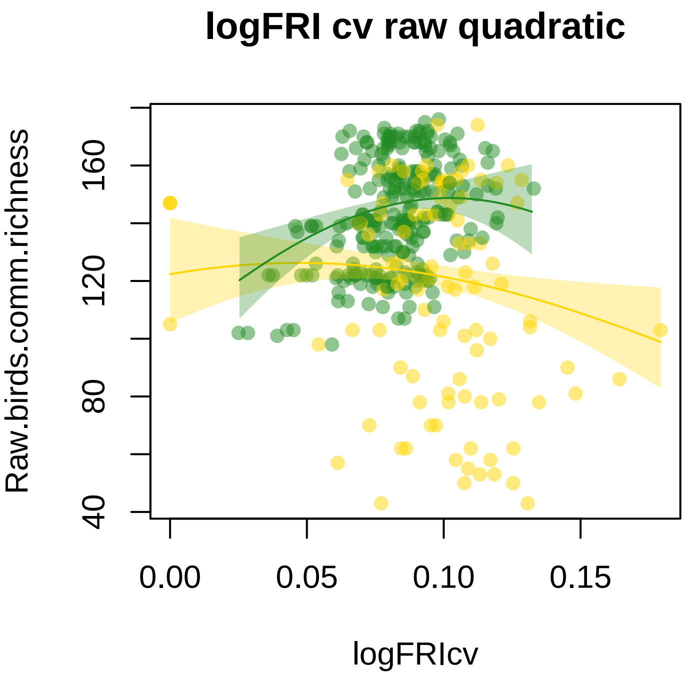

logFRI cv raw quadratic
Raw.birds.comm.richness
40
80
120
160
0.00
0.05
0.10
0.15
logFRIcv

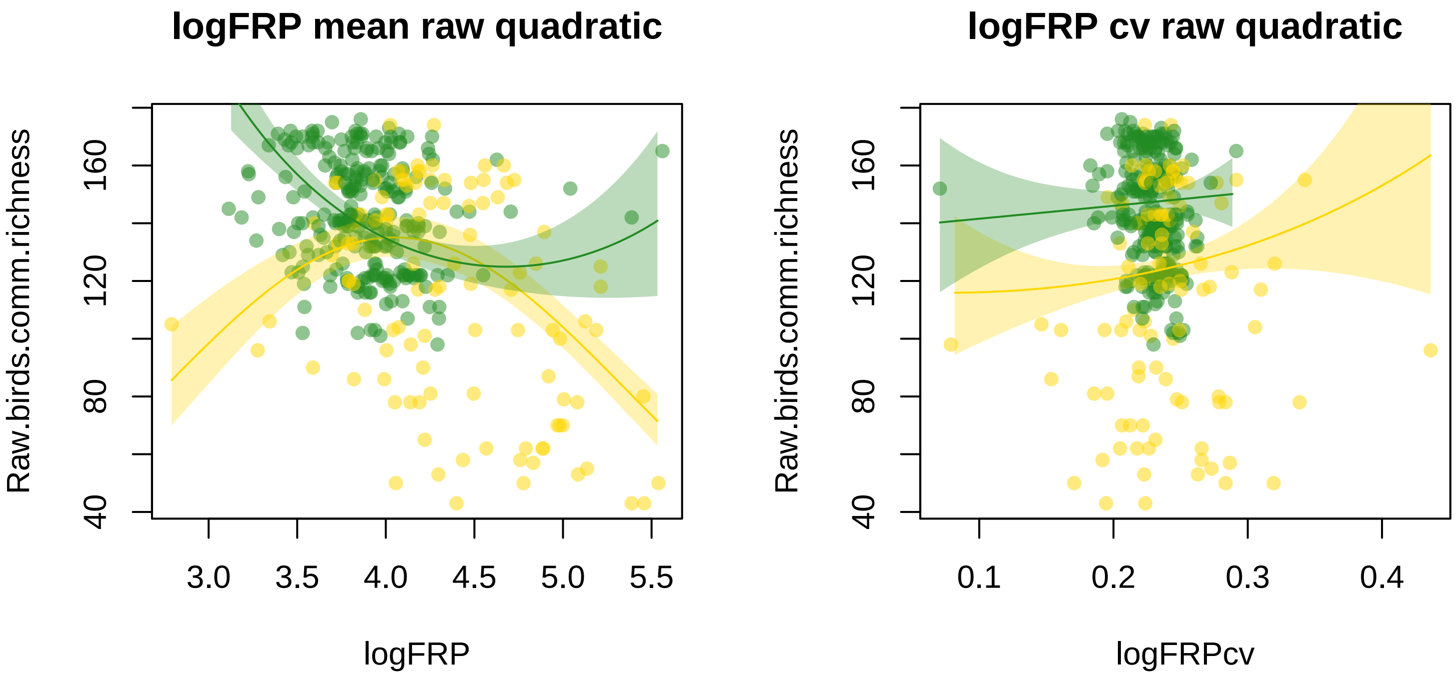
logFRP mean raw quadratic
Raw.birds.comm.richness
40
80
120
160
3.0
3.5
4.0
4.5
5.0
5.5
logFRP
logFRP cv raw quadratic
Raw.birds.comm.richness
40
80
120
160
0.1
0.2
0.3
0.4
logFRPcv

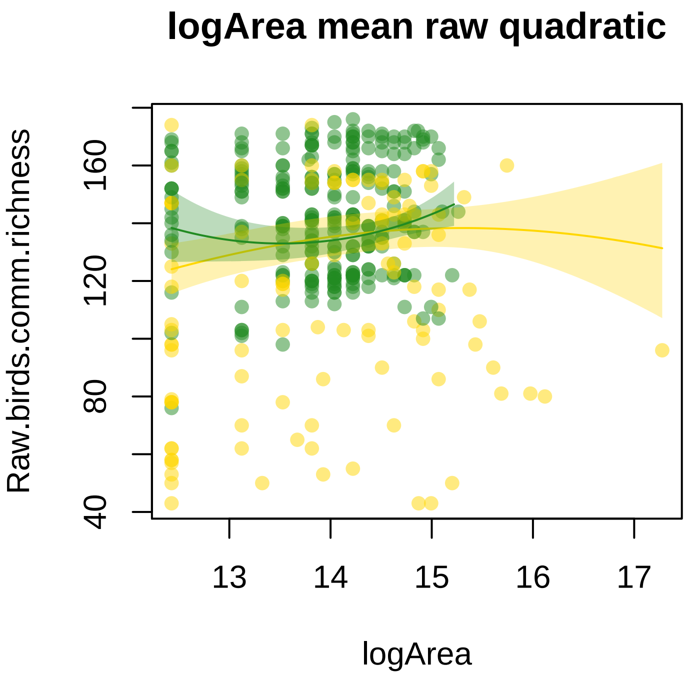
logArea mean raw quadratic
Raw.birds.comm.richness
40
80
120
160
13
14
15
16
17
logArea

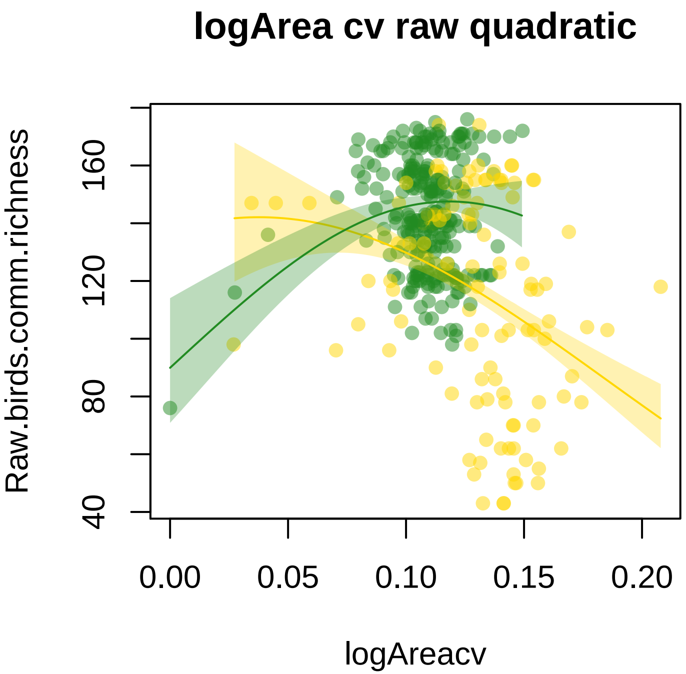
logArea cv raw quadratic
Raw.birds.comm.richness
40
80
120
160
0.00
0.05
0.10
0.15
0.20
logAreacv

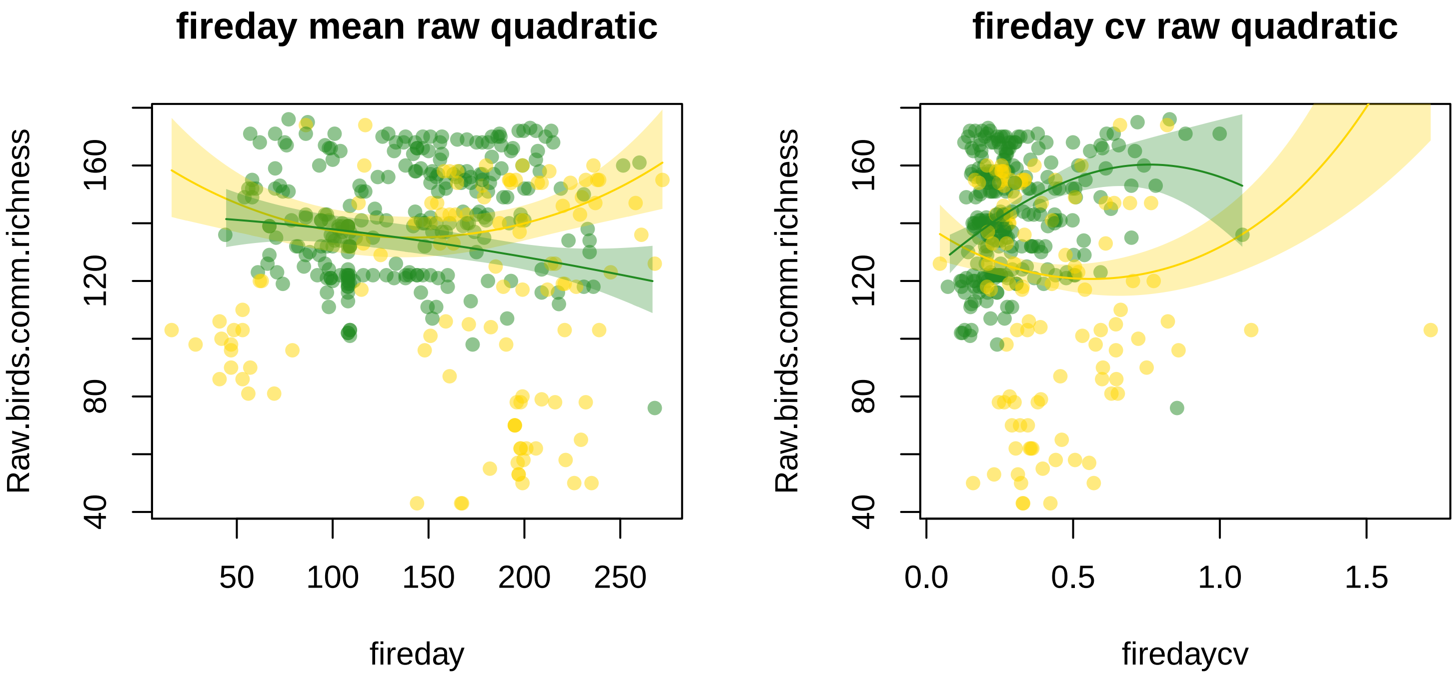
fireday mean raw quadratic
Raw.birds.comm.richness
40
80
120
160
50
100
150
200
250
fireday
fireday cv raw quadratic
Raw.birds.comm.richness
40
80
120
160
0.0
0.5
1.0
1.5
firedaycv
