# Supplementary material for: Pyrodiversity interacts with rainfall to increase bird and mammal richness in African savannas
Source: Ecol Lett. 2018 Feb 14;21(4):557–67. doi: 10.1111/ele.12921 (PMC5888149; doi:10.1111/ele.12921)
Supplement: Supplementary file 3 [file ELE-21-557-s003.docx]

**Supporting information Appendix S1**

***Methods for identifying savannas in Africa from White (1983)***

The following vegetation categories from White (1983) were used to constrain the extent of savannas in Africa: 16b, 19a, 20, 22a, 20, 24, 25, 26, 27, 28, 29a, 29b, 29c, 29d, 29e, 31, 35a, 35b, 35c, 36, 37, 38, 40, 43, 44, 45, 47, 48, 51, 56, 57a, 57b, 58, 59, 60, 61, 62, 63, 64 and 75. The region covered by these vegetation types was then further constrained by imposing a minimum rainfall threshold of 300 mm.yr^-1^.

***Methods for validation of species lists***

For our results to be valuable, it is important that the estimated species richness of birds and mammals in each protected area is accurate, or at least strongly correlated with actual richness on the ground. To ensure this, we used the best datasets available. IUCN redlist polygons for mammals are created to map the actual extent of occurrence of species at the time of listing (all since 1999), and are probably at their most accurate within protected areas. The bird data has been widely used to map richness and assess conservation status (e.g. Larsen et al 2007) and is built from a diverse set of sources, and checked by experts locally to reflect the actual distribution of species in 2001. Nonetheless, it is important to assess our checklists of bird and mammal species using independent data on the ground if possible. To do this, we used published checklists available for a subset of 10 sites. For mammals, checklists of larger mammals were available for Tanzanian protected areas from Foley et al 2015, for Kruger we found data from SANParks (<https://www.sanparks.org/docs/parks_kruger/conservation/scientific/ff/mammal_checklist.pdf>). For birds, full checklists are unwise as they frequently contain large numbers of rare and vagrant species that should not be included in these analyses. Consequently, we used IBA descriptions from Birdlife (Important Bird Areas factsheets. Downloaded from <http://datazone.birdlife.org/home)> which list typical species present in each IBA, with an emphasis on the ‘special’ species of the region. This allowed us to check for missing species in bird lists using the IBA descriptions.

For larger mammals, we built lists for Kruger (South Africa), Selous, Tarangire, Mkomazi, Serengeti, Ruaha, Katavi, and Saadane ecosystems (Tanzania). We found our lists missed between 0 and 5% of species per protected area (median richness of larger mammals as recorded by checklists in our areas was 52, range: 35- 65), and included a median of 3% (0 – 13%) species not present on checklists. Note that for some of these (e.g. *Otolemur* species in Tarangire NP) the source checklists note that they are unrecorded but are possibly present because suitable habitat exists in the protected area. Until widespread checklists of bats and smaller rodents are compiled, we have to assume the patterns remain similar across the groups.

For birds, we built lists for Mole (Ghana), Air Tenere (Algeria), Ferlo Nord (Senegal), Serengeti (Tanzania), Tsavo (Kenya), Tarangire (Tanzania), Kruger (South Africa) St Floris (CAR) and Khalahari (Botswana). We found our species lists missed a median of 9% (IQR 3% - 12.5%) of species identified by BirdLife for those protected areas. Although the BirdLife lists are not designed to be comprehensive, we found a strong positive correlation between total richness in our lists and number of species mentioned in the BirdLife accounts (r = 0.74, d.f = 7, p = 0.022), providing further evidence that our richness surface reflects one the ground reality.

***Covariate correlations:***

Correlations and plots of covariates are presented in Supplementary figures 1-6, with green dots indicating wet savanna and yellow indicating dry savanna. Titles give r^2^. Note that the correlations between variable are low and there is no important heteroscedasticity, so fitting models with an interaction between fire covariates and rainfall is a reasonable test for such a relationship.

***Comparison of modern (post 2000) with historic fire attributes***

To ensure that our index of pyrodiversity based on MODIS data (post 2000) is indicative of historic fire regimes, we compared spatial variation in those attributes of fires that were historically available for Kruger and Hluhluwe-iUmfolozi parks (fire return interval, area burnt and fire season) for the 15 year periods 1970-1984 and 1985-1999 with the spatial variation recorded from MODIS and used to generate our index of pyrodiversity. We assumed that if spatial variation is reasonably consistent across this time period, our index would be a reasonable index of long-term fire regimes capable of influencing the distribution of species. The long-term fire data were derived from vector maps of the location and the date of individual fires which were recorded by section rangers in the Hluhluwe iMfolozi Park and the Kruger National Park. Ezemvelo Wildlife and the South African National Parks respectively have been recording this information since 1955 (HiP) and 1957 (Kruger). Although these data are very different from the satellite derived maps (the section rangers would mark the extent of the fire on printed maps for example), they have been gathered consistently between years, and therefore represent an alternative record of the variability in these fire attributes over a longer period than is available from remotely sensed data. Fire size and date were available in the original datasets. The time since last fire was calculated from these maps in the same way as it was calculated from the satellite data (see Hempson 2017 Ecospheres). There are no long-term fire radiative power data available. These data are the property of the conservation agencies, but can be made available to researchers on request.

We aggregated data into 10 x 10km squares and correlated the spatial patterns from each variable in each time period for each park separately, computing the correlation for the means and standard deviation of each variable in each cell.

Correlations for fire return interval over time were the strongest and most significant correlated variables over time, significant in all six comparisons and averaged 0.81 (range = 0.50, 0.94). Spatial structure in variability was also strongly correlated over time (mean correlation 0.64, range 0.24, 0.86).

Correlations for area over time for mean area were significant for five of six comparisons and averaged 0.48 (range = -0.14, 0.87). The non-significant correlation was between the most recent data and the oldest data from Kruger – a period where active management aimed at altering fire size has been implemented. Variability was weakly correlated over time (mean correlation 0.12, range -0.5, 0.87)

Weakest relationships were found for seasonality. Indeed, in both parks, all correlations in mean season of fire were negative (mean = -0.17, range -0.06, -0.36) reflecting active management change in fire seasonality (Hempson et al 2017). Despite negative correlations in mean variables variation in season remained positively (albeit weakly) positively correlated over time (mean = 0.15, range = 0.09, 0.29), and in HiP.

Although the empirical evidence is rather limited, the existence of broad temporal correlations over 60 years in spatial variation of fire attributes despite active management aimed at altering fire regimes suggests our wider spatial analysis using modern fire variables is likely to reflect spatial patterns in the fire regime over the longer periods that generate species distributions. This is perhaps further supported by the strong influence of rainfall variation on most of the measured variables that we have previously reported (Hempson et al 2017): a pattern that is likely to be largely stable over time.

***Full models:***

Full model results are presented in Supplementary figures 7-86. For each figure the left-hand panel shows the results for the mean value of the covariate and the right hand shows the relationship with the coefficient of variation. Model descriptions (title) are as in Supplement 4. Note that quadratic models show qualitatively similar patterns (increases or decreases) that match those of equivalent linear plots, but in some examples appear overfitted.

***Comparison of fire traits inside and outside of protected areas***

**Figure S55.** The effect of burning location (inside versus outside of protected areas) on pyrodiversity, variability in individual fire attributes and fire attributes. Values correspond to beta coefficients and 95% confidence intervals from output of separate general linear mixed-effects models, with all values being the parameter estimate within protected areas, relative to outside of protected areas (i.e. a positive value indicates greater variation or mean effects inside than outside protected areas). We show results for two buffer sizes surrounding protected areas, representing “outside” locations: (1) 100km buffer (blue, values reported in the main text of the manuscript) and (2) 50 km buffer (red). Note “Fireday” is on a different scale from other coefficients, and “Fireday” was not significant (i.e. no difference between day of fire inside vs outside of PA’s) when using the 50 km buffer size around PA’s. All other data categories remained qualitatively similar when using 50 km versus 100 km buffers.


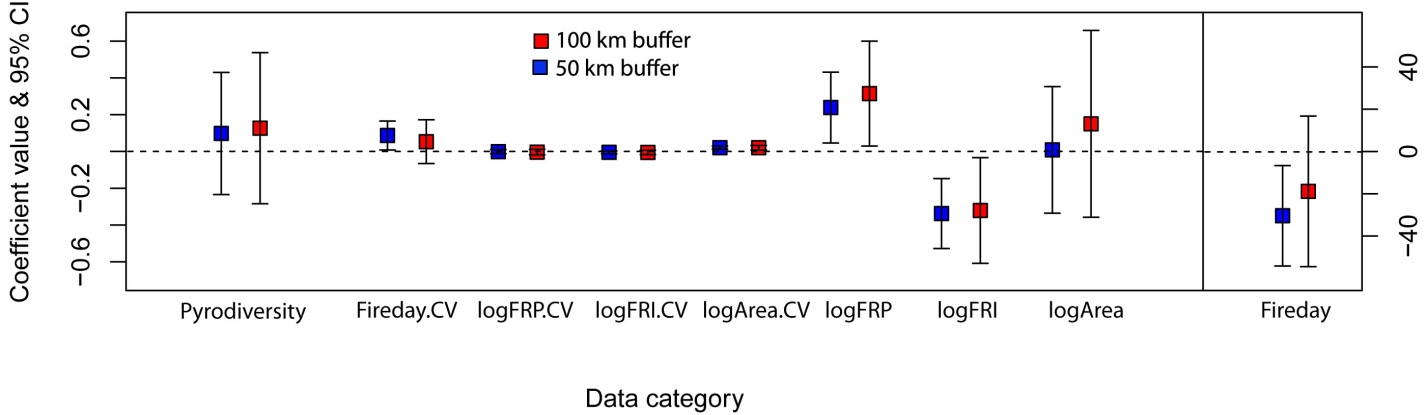


***Supplementary references:***

Foley, C., Foley, L., Lobora, A., De Luca, D., Msuha, M., Davenport, T. R., & Durant, S. M. (2014). *A Field Guide to the Larger Mammals of Tanzania*. Princeton University Press.

Larsen F.W., Bladt J., and Rahbek C. 2007. Improving the performance of indicator groups for the identification of important areas for conservation of species. Conservation Biology DOI: 10.1111/j.1523-1739.2007.00658.x.
