# Supplementary material for: Pyrodiversity interacts with rainfall to increase bird and mammal richness in African savannas
Source: Ecol Lett. 2018 Feb 14;21(4):557–67. doi: 10.1111/ele.12921 (PMC5888149; doi:10.1111/ele.12921)
Supplement: Supplementary file 4 [file ELE-21-557-s004.docx]

SUPPORTING INFORMATION APPENDIX 2: Savannah mammal species included in the analysis: to be included, each species must be regularly found within savannah, though it may well primarily occur in other biomes.

| Scientific Name | Body Mass (g) |
| --- | --- |
| *Pipistrellus musciculus* | 2 |
| *Crocidura lusitania* | 2.5 |
| *Pipistrellus nanulus* | 2.5 |
| *Pipistrellus somalicus* | 3.5 |
| *Pipistrellus helios* | 3.6 |
| *Crocidura fulvastra* | 3.7 |
| *Pipistrellus nanus* | 3.9 |
| *Crocidura cinderella* | 4 |
| *Pipistrellus zuluensis* | 4.1 |
| *Cloeotis percivali* | 4.2 |
| *Suncus infinitesimus* | 4.4 |
| *Scotoecus albofuscus* | 4.5 |
| *Pipistrellus rusticus* | 4.6 |
| *Pipistrellus inexspectatus* | 4.9 |
| *Crocidura yankariensis* | 5 |
| *Pipistrellus anchietae* | 5 |
| *Pipistrellus guineensis* | 5 |
| *Pipistrellus hesperidus* | 5 |
| *Steatomys parvus* | 5 |
| *Suncus varilla* | 5 |
| *Nycticeinops schlieffeni* | 5.1 |
| *Crocidura allex* | 5.2 |
| *Pipistrellus tenuipinnis* | 5.4 |
| *Suncus megalura* | 5.4 |
| *Crocidura baileyi* | 5.5 |
| *Hipposideros jonesi* | 5.5 |
| *Crocidura fuscomurina* | 5.6 |
| *Pipistrellus capensis* | 6 |
| *Laephotis wintoni* | 6.1 |
| *Mus indutus* | 6.2 |
| *Pipistrellus savii* | 6.3 |
| *Rhinolophus denti* | 6.3 |
| *Cistugo lesueuri* | 6.4 |
| *Mus minutoides* | 6.4 |
| *Pipistrellus rendalli* | 6.4 |
| *Crocidura nanilla* | 6.5 |
| *Crocidura pasha* | 6.5 |
| *Kerivoula lanosa* | 6.7 |
| *Laephotis angolensis* | 6.7 |
| *Laephotis namibensis* | 6.7 |
| *Mus setzeri* | 6.7 |
| *Crocidura silacea* | 7 |
| *Nycteris nana* | 7 |
| *Nycteris gambiensis* | 7.1 |
| *Pipistrellus rueppellii* | 7.1 |
| *Rhinolophus swinnyi* | 7.1 |
| *Eptesicus floweri* | 7.2 |
| *Pipistrellus melckorum* | 7.2 |
| *Laephotis botswanae* | 7.3 |
| *Miniopterus fraterculus* | 7.4 |
| *Tadarida chapini* | 7.5 |
| *Nycteris woodi* | 7.6 |
| *Myotis bocagii* | 7.9 |
| *Dendromus oreas* | 8 |
| *Nycteris hispida* | 8 |
| *Suncus lixus* | 8 |
| *Rhinolophus simulator* | 8.1 |
| *Dendromus mystacalis* | 8.2 |
| *Mus musculoides* | 8.2 |
| *Crocidura jacksoni* | 8.5 |
| *Scotoecus hirundo* | 8.5 |
| *Crocidura cyanea* | 8.6 |
| *Asellia patrizii* | 8.7 |
| *Mimetillus moloneyi* | 8.9 |
| *Rhinolophus darlingi* | 8.9 |
| *Surdisorex polulus* | 9 |
| *Crocidura douceti* | 9.1 |
| *Dendromus melanotis* | 9.1 |
| *Nycteris thebaica* | 9.2 |
| *Glauconycteris argentata* | 9.3 |
| *Rhinolophus landeri* | 9.4 |
| *Hipposideros caffer* | 9.5 |
| *Tadarida demonstrator* | 9.7 |
| *Dendromus vernayi* | 10 |
| *Desmodilliscus braueri* | 10 |
| *Mus baoulei* | 10 |
| *Mus callewaerti* | 10 |
| *Mus goundae* | 10 |
| *Mus haussa* | 10 |
| *Mus mattheyi* | 10 |
| *Mus neavei* | 10 |
| *Mus oubanguii* | 10 |
| *Mus sorella* | 10 |
| *Mus tenellus* | 10 |
| *Kerivoula argentata* | 10.1 |
| *Dendromus mesomelas* | 10.2 |
| *Rhinolophus blasii* | 10.3 |
| *Miniopterus natalensis* | 10.4 |
| *Dendromus kivu* | 10.5 |
| *Graphiurus kelleni* | 10.5 |
| *Pipistrellus flavescens* | 10.5 |
| *Hipposideros ruber* | 10.6 |
| *Coleura afra* | 10.7 |
| *Nycteris arge* | 10.8 |
| *Tadarida pumila* | 11 |
| *Crocidura glassi* | 11.2 |
| *Mus setulosus* | 11.2 |
| *Glauconycteris variegata* | 11.3 |
| *Crocidura somalica* | 11.5 |
| *Miniopterus schreibersii* | 11.5 |
| *Myosorex tenuis* | 11.7 |
| *Myosorex varius* | 11.7 |
| *Nycteris aurita* | 11.7 |
| *Nycteris intermedia* | 11.7 |
| *Nycteris major* | 11.7 |
| *Nycteris vinsoni* | 11.7 |
| *Sylvisorex isabellae* | 12 |
| *Tadarida bemmeleni* | 12.4 |
| *Gerbillus pusillus* | 12.6 |
| *Mus triton* | 12.6 |
| *Crocidura greenwoodi* | 13.1 |
| *Crocidura lamottei* | 13.1 |
| *Crocidura lucina* | 13.1 |
| *Crocidura macarthuri* | 13.1 |
| *Crocidura macmillani* | 13.1 |
| *Crocidura nana* | 13.1 |
| *Crocidura parvipes* | 13.1 |
| *Crocidura roosevelti* | 13.1 |
| *Crocidura smithii* | 13.1 |
| *Crocidura thalia* | 13.1 |
| *Crocidura voi* | 13.1 |
| *Crocidura xantippe* | 13.1 |
| *Rhinolophus fumigatus* | 13.1 |
| *Triaenops persicus* | 13.2 |
| *Myotis tricolor* | 13.7 |
| *Crocidura fischeri* | 14 |
| *Myotis morrisi* | 14.1 |
| *Sauromys petrophilus* | 14.3 |
| *Nycteris macrotis* | 14.5 |
| *Myomyscus angolensis* | 15 |
| *Myomyscus brockmani* | 15 |
| *Rhinolophus clivosus* | 15 |
| *Rhinolophus guineensis* | 15 |
| *Rhinolophus sakejiensis* | 15 |
| *Tadarida bivittata* | 15 |
| *Tadarida major* | 15.2 |
| *Crocidura hirta* | 15.8 |
| *Myotis welwitschii* | 15.9 |
| *Tadarida ansorgei* | 16 |
| *Tadarida brachyptera* | 16 |
| *Crocidura viaria* | 16.5 |
| *Megaloglossus woermanni* | 16.7 |
| *Graphiurus lorraineus* | 16.8 |
| *Malacothrix typica* | 17 |
| *Crocidura poensis* | 17.1 |
| *Crocidura theresae* | 17.6 |
| *Tadarida aegyptiaca* | 17.6 |
| *Rhinolophus alcyone* | 18.6 |
| *Rhinolophus eloquens* | 19.2 |
| *Crocidura longipes* | 19.3 |
| *Tadarida aloysiisabaudiae* | 19.5 |
| *Scotophilus viridis* | 19.8 |
| *Crocidura foxi* | 20 |
| *Graphiurus murinus* | 20.1 |
| *Acomys louisae* | 20.2 |
| *Scotophilus leucogaster* | 20.2 |
| *Tadarida niangarae* | 20.5 |
| *Steatomys krebsii* | 20.7 |
| *Tadarida trevori* | 21.2 |
| *Tadarida niveiventer* | 21.8 |
| *Nanonycteris veldkampii* | 21.9 |
| *Acomys kempi* | 22 |
| *Acomys percivali* | 22 |
| *Neamblysomus julianae* | 22 |
| *Tadarida nigeriae* | 22 |
| *Crocidura nigeriae* | 23 |
| *Crocidura wimmeri* | 23.5 |
| *Surdisorex norae* | 23.6 |
| *Lavia frons* | 23.8 |
| *Steatomys bocagei* | 24 |
| *Steatomys caurinus* | 24 |
| *Steatomys cuppedius* | 24 |
| *Calcochloris obtusirostris* | 24.1 |
| *Calcochloris tytonis* | 24.1 |
| *Taphozous perforatus* | 24.4 |
| *Scotophilus dinganii* | 25.1 |
| *Graphiurus johnstoni* | 25.4 |
| *Graphiurus monardi* | 25.4 |
| *Micropteropus intermedius* | 25.4 |
| *Micropteropus pusillus* | 25.4 |
| *Gerbillurus paeba* | 25.9 |
| *Rhinolophus hildebrandti* | 26 |
| *Acomys spinosissimus* | 26.5 |
| *Cardioderma cor* | 26.5 |
| *Lemniscomys macculus* | 26.5 |
| *Tadarida condylura* | 26.6 |
| *Idiurus macrotis* | 27.2 |
| *Scotophilus nigrita* | 27.3 |
| *Taphozous mauritianus* | 28 |
| *Gerbillus harwoodi* | 29 |
| *Gerbillus juliani* | 29 |
| *Gerbillus pulvinatus* | 29 |
| *Graphiurus angolensis* | 29.5 |
| *Graphiurus microtis* | 29.5 |
| *Acomys ignitus* | 29.7 |
| *Nycteris grandis* | 29.8 |
| *Taphozous hamiltoni* | 30 |
| *Tadarida lobata* | 30.4 |
| *Steatomys pratensis* | 30.6 |
| *Crocidura flavescens* | 31.2 |
| *Chlorotalpa duthieae* | 31.3 |
| *Praomys derooi* | 32 |
| *Taphozous nudiventris* | 32.5 |
| *Hipposideros cyclops* | 32.9 |
| *Tadarida fulminans* | 33.9 |
| *Crocidura olivieri* | 34.4 |
| *Acomys cineraceus* | 34.6 |
| *Hipposideros abae* | 34.8 |
| *Hipposideros lamottei* | 34.8 |
| *Hipposideros megalotis* | 34.8 |
| *Hipposideros vittatus* | 34.8 |
| *Otomops martiensseni* | 34.9 |
| *Praomys daltoni* | 35 |
| *Gerbillurus vallinus* | 35.2 |
| *Gerbillus rupicola* | 35.5 |
| *Uranomys ruddi* | 35.5 |
| *Rhinolophus maclaudi* | 35.8 |
| *Acomys johannis* | 36.6 |
| *Grammomys macmillani* | 37 |
| *Tadarida ventralis* | 37.4 |
| *Plerotes anchietae* | 38 |
| *Gerbillus dunni* | 39 |
| *Steatomys opimus* | 40 |
| *Rhabdomys pumilio* | 40.7 |
| *Lemniscomys mittendorfi* | 41.9 |
| *Mastomys natalensis* | 41.9 |
| *Grammomys aridulus* | 43.3 |
| *Lemniscomys bellieri* | 43.6 |
| *Lemniscomys hoogstraali* | 43.6 |
| *Lemniscomys linulus* | 43.6 |
| *Lemniscomys roseveari* | 43.6 |
| *Lemniscomys zebra* | 43.6 |
| *Lemniscomys striatus* | 43.7 |
| *Elephantulus brachyrhynchus* | 45.1 |
| *Tadarida midas* | 45.5 |
| *Graphiurus platyops* | 46.1 |
| *Mastomys shortridgei* | 46.5 |
| *Elephantulus fuscus* | 47 |
| *Elephantulus intufi* | 48.9 |
| *Epomophorus grandis* | 49.4 |
| *Lemniscomys rosalia* | 49.9 |
| *Taterillus gracilis* | 49.9 |
| *Ammodillus imbellis* | 50 |
| *Pelomys minor* | 50 |
| *Saccostomus campestris* | 50.3 |
| *Mastomys awashensis* | 51.6 |
| *Mastomys huberti* | 51.6 |
| *Mastomys kollmannspergeri* | 51.6 |
| *Mastomys pernanus* | 51.6 |
| *Taterillus emini* | 52 |
| *Elephantulus rufescens* | 52.8 |
| *Mastomys coucha* | 53.8 |
| *Zelotomys woosnami* | 54.1 |
| *Zelotomys hildegardeae* | 55.2 |
| *Lemniscomys griselda* | 55.9 |
| *Neamblysomus gunningi* | 56 |
| *Elephantulus fuscipes* | 57 |
| *Elephantulus myurus* | 59.5 |
| *Taterillus pygargus* | 60.2 |
| *Colomys goslingi* | 62.5 |
| *Amblysomus hottentotus* | 62.6 |
| *Lophuromys sikapusi* | 62.7 |
| *Mastomys erythroleucus* | 64.1 |
| *Saccostomus mearnsi* | 64.9 |
| *Cryptomys darlingi* | 65.6 |
| *Elephantulus rupestris* | 65.6 |
| *Lissonycteris angolensis* | 68.3 |
| *Epomophorus labiatus* | 69.7 |
| *Amblysomus marleyi* | 70 |
| *Paraxerus boehmi* | 70.2 |
| *Gerbilliscus leucogaster* | 73.8 |
| *Cryptomys hottentotus* | 75.1 |
| *Thallomys paedulcus* | 77.7 |
| *Dasymys nudipes* | 78 |
| *Taterillus congicus* | 80 |
| *Taterillus lacustris* | 80 |
| *Taterillus petteri* | 80 |
| *Taterillus tranieri* | 80 |
| *Aethomys chrysophilus* | 80.9 |
| *Cryptomys kafuensis* | 85 |
| *Pelomys campanae* | 85 |
| *Cryptomys anselli* | 85.6 |
| *Mystromys albicaudatus* | 86.9 |
| *Arvicanthis ansorgei* | 87 |
| *Aethomys namaquensis* | 88 |
| *Epomophorus angolensis* | 88.8 |
| *Epomophorus anselli* | 88.8 |
| *Epomophorus minimus* | 88.8 |
| *Arvicanthis neumanni* | 89 |
| *Gerbilliscus brantsii* | 89.4 |
| *Oenomys hypoxanthus* | 89.8 |
| *Aethomys kaiseri* | 90 |
| *Aethomys bocagei* | 92 |
| *Aethomys stannarius* | 92 |
| *Cryptomys bocagei* | 93.8 |
| *Epomophorus crypturus* | 95 |
| *Arvicanthis niloticus* | 95.8 |
| *Gerbilliscus robustus* | 96 |
| *Aethomys thomasi* | 100 |
| *Thallomys loringi* | 100 |
| *Thallomys shortridgei* | 100 |
| *Otomys angoniensis* | 100.6 |
| *Gerbilliscus kempi* | 100.8 |
| *Gerbilliscus guineae* | 102.5 |
| *Arvicanthis rufinus* | 103.3 |
| *Paraxerus ochraceus* | 104.3 |
| *Rousettus lanosus* | 104.8 |
| *Gerbilliscus boehmi* | 105.8 |
| *Gerbilliscus gambiana* | 105.8 |
| *Chrysospalax villosus* | 108.4 |
| *Mylomys dybowskii* | 111 |
| *Funisciurus congicus* | 112 |
| *Cryptomys damarensis* | 112.2 |
| *Myopterus daubentonii* | 114 |
| *Platymops setiger* | 114 |
| *Hipposideros gigas* | 115.4 |
| *Dasymys foxi* | 118 |
| *Gerbilliscus inclusus* | 118.5 |
| *Epomops franqueti* | 119 |
| *Pelomys fallax* | 120.5 |
| *Gerbilliscus validus* | 121 |
| *Epomops dobsonii* | 122.1 |
| *Gerbilliscus nigricaudus* | 123 |
| *Thallomys nigricauda* | 124.5 |
| *Parotomys littledalei* | 127 |
| *Gerbilliscus phillipsi* | 127.1 |
| *Dasymys incomtus* | 127.8 |
| *Aethomys ineptus* | 133.2 |
| *Epomophorus gambianus* | 134.6 |
| *Funisciurus bayonii* | 135 |
| *Aethomys hindei* | 140.7 |
| *Otomys laminatus* | 150 |
| *Heliophobius argenteocinereus* | 159.5 |
| *Paraxerus flavovittis* | 160 |
| *Nesokia indica* | 178.1 |
| *Arvicanthis nairobae* | 180 |
| *Otomys anchietae* | 180 |
| *Otomys saundersiae* | 180 |
| *Otomys tropicalis* | 180 |
| *Funisciurus substriatus* | 185.7 |
| *Galago moholi* | 192 |
| *Cryptomys ochraceocinereus* | 200 |
| *Galago senegalensis* | 215.2 |
| *Cryptomys zechi* | 217 |
| *Paraxerus cepapi* | 222.9 |
| *Tachyoryctes splendens* | 227.1 |
| *Funisciurus pyrropus* | 243.2 |
| *Heliosciurus gambianus* | 248.4 |
| *Galago gallarum* | 250 |
| *Atlantoxerus getulus* | 250.6 |
| *Eidolon helvum* | 254.6 |
| *Cryptomys mechowi* | 271.5 |
| *Helogale parvula* | 281.8 |
| *Atelerix albiventris* | 293.4 |
| *Poecilogale albinucha* | 308.2 |
| *Xerus rutilus* | 317 |
| *Atelerix frontalis* | 329.1 |
| *Heliosciurus rufobrachium* | 332.9 |
| *Hypsignathus monstrosus* | 337 |
| *Atelerix sclateri* | 360 |
| *Dologale dybowskii* | 361.9 |
| *Helogale hirtula* | 484.6 |
| *Xerus inauris* | 572.6 |
| *Xerus erythropus* | 602.2 |
| *Anomalurus derbianus* | 665.8 |
| *Cynictis penicillata* | 694.4 |
| *Suricata suricatta* | 730 |
| *Lophiomys imhausi* | 755 |
| *Ictonyx striatus* | 811 |
| *Otolemur crassicaudatus* | 1206.6 |
| *Mungos mungo* | 1260 |
| *Cricetomys gambianus* | 1267.5 |
| *Felis nigripes* | 1362.8 |
| *Crossarchus obscurus* | 1395.1 |
| *Genetta thierryi* | 1400 |
| *Genetta abyssinica* | 1405.5 |
| *Mungos gambianus* | 1645 |
| *Paracynictis selousi* | 1669.7 |
| *Genetta genetta* | 1756.2 |
| *Lepus microtis* | 1763.6 |
| *Genetta angolensis* | 1859.6 |
| *Genetta cristata* | 1863.2 |
| *Genetta maculata* | 1950 |
| *Lepus capensis* | 2005 |
| *Lepus habessinicus* | 2015.6 |
| *Genetta tigrina* | 2066.6 |
| *Lepus fagani* | 2227.7 |
| *Rhynchogale melleri* | 2240.3 |
| *Pronolagus rupestris* | 2250 |
| *Pronolagus saundersiae* | 2250 |
| *Pronolagus randensis* | 2376.5 |
| *Pronolagus crassicaudatus* | 2415.3 |
| *Heterohyrax brucei* | 2453.7 |
| *Madoqua piacentinii* | 2500 |
| *Poelagus marjorita* | 2509.7 |
| *Pedetes surdaster* | 2549 |
| *Pedetes capensis* | 2549.3 |
| *Lepus saxatilis* | 2593.6 |
| *Thryonomys gregorianus* | 2707.1 |
| *Lepus starcki* | 2760.7 |
| *Vulpes pallida* | 2800 |
| *Vulpes chama* | 2919.8 |
| *Procavia capensis* | 2952.5 |
| *Herpestes ichneumon* | 2980 |
| *Dendrohyrax arboreus* | 2981.1 |
| *Herpestes flavescens* | 3000 |
| *Herpestes ochraceus* | 3000 |
| *Herpestes pulverulentus* | 3000 |
| *Herpestes sanguineus* | 3000 |
| *Dendrohyrax dorsalis* | 3175 |
| *Madoqua saltiana* | 3424.5 |
| *Cercopithecus pogonias* | 3578.3 |
| *Ichneumia albicauda* | 3628.4 |
| *Thryonomys swinderianus* | 3750.9 |
| *Otocyon megalotis* | 4098.1 |
| *Felis silvestris* | 4573.1 |
| *Madoqua guentheri* | 4623.7 |
| *Madoqua kirkii* | 4833.4 |
| *Chlorocebus sabaeus* | 5000 |
| *Nesotragus moschatus* | 5400 |
| *Chlorocebus cynosuros* | 6000 |
| *Chlorocebus djamdjamensis* | 6000 |
| *Chlorocebus pygerythrus* | 6000 |
| *Chlorocebus aethiops* | 6500 |
| *Felis chaus* | 7158 |
| *Chlorocebus tantalus* | 7250 |
| *Erythrocebus patas* | 7966.3 |
| *Proteles cristata* | 8139.4 |
| *Canis mesomelas* | 8247.3 |
| *Mellivora capensis* | 9000 |
| *Raphicerus sharpei* | 9396.9 |
| *Canis aureus* | 9658.7 |
| *Canis adustus* | 10392.5 |
| *Raphicerus melanotis* | 10503.1 |
| *Dorcatragus megalotis* | 10918.1 |
| *Raphicerus campestris* | 11661.5 |
| *Caracal caracal* | 11964.4 |
| *Leptailurus serval* | 12000 |
| *Civettictis civetta* | 12075.6 |
| *Cephalophus rufilatus* | 12114.6 |
| *Hystrix cristata* | 13406.3 |
| *Oreotragus oreotragus* | 13486.6 |
| *Papio hamadryas* | 14007.1 |
| *Canis simensis* | 14361.9 |
| *Hystrix africaeaustralis* | 14936 |
| *Gazella dorcas* | 15636 |
| *Sylvicapra grimmia* | 15639.2 |
| *Papio cynocephalus* | 15822.2 |
| *Theropithecus gelada* | 15964.1 |
| *Ourebia ourebi* | 17186.1 |
| *Papio anubis* | 17728.6 |
| *Papio ursinus* | 17729.4 |
| *Smutsia temminckii* | 18000 |
| *Papio papio* | 18026.1 |
| *Aonyx capensis* | 19322.2 |
| *Gazella spekei* | 20000 |
| *Lycaon pictus* | 22000 |
| *Pelea capreolus* | 22731.3 |
| *Eudorcas thomsonii* | 22907.4 |
| *Eudorcas albonotata* | 24953.6 |
| *Eudorcas rufifrons* | 26999.8 |
| *Ammodorcas clarkei* | 28049.8 |
| *Redunca fulvorufula* | 29352.7 |
| *Antidorcas marsupialis* | 33571.2 |
| *Smutsia gigantea* | 35000 |
| *Hyaena hyaena* | 35070.5 |
| *Litocranius walleri* | 38804.4 |
| *Nanger soemmerringii* | 41582.9 |
| *Hyaena brunnea* | 42977.9 |
| *Tragelaphus scriptus* | 43250.4 |
| *Redunca redunca* | 43289 |
| *Pan troglodytes* | 45000 |
| *Capra nubiana* | 47763.7 |
| *Acinonyx jubatus* | 50577.9 |
| *Panthera pardus* | 52400 |
| *Aepyceros melampus* | 52591.7 |
| *Nanger granti* | 55464.5 |
| *Orycteropus afer* | 56175.2 |
| *Redunca arundinum* | 58059.2 |
| *Cephalophus silvicultor* | 62006.6 |
| *Crocuta crocuta* | 63370 |
| *Potamochoerus larvatus* | 69063.8 |
| *Potamochoerus porcus* | 70000.3 |
| *Nanger dama* | 71424.8 |
| *Kobus vardonii* | 71462.7 |
| *Tragelaphus spekii* | 75554.3 |
| *Phacochoerus aethiopicus* | 75607.5 |
| *Damaliscus pygargus* | 77784.6 |
| *Beatragus hunteri* | 79132.2 |
| *Kobus kob* | 80035.2 |
| *Phacochoerus africanus* | 82500 |
| *Kobus megaceros* | 85805.9 |
| *Tragelaphus angasii* | 87616.8 |
| *Kobus leche* | 88645 |
| *Ammotragus lervia* | 94202.2 |
| *Tragelaphus imberbis* | 94320.4 |
| *Capra walie* | 100144 |
| *Damaliscus lunatus* | 136000.3 |
| *Connochaetes gnou* | 156547.5 |
| *Panthera leo* | 158623.9 |
| *Alcelaphus buselaphus* | 160937.9 |
| *Oryx gazella* | 188404.5 |
| *Hylochoerus meinertzhageni* | 198130.5 |
| *Connochaetes taurinus* | 198619.7 |
| *Oryx beisa* | 200576.5 |
| *Kobus ellipsiprymnus* | 204393.5 |
| *Tragelaphus strepsiceros* | 206056.4 |
| *Hippotragus niger* | 236405.9 |
| *Hippotragus equinus* | 264174 |
| *Equus africanus* | 275000 |
| *Equus zebra* | 282462.1 |
| *Equus quagga* | 400000 |
| *Equus grevyi* | 408000.4 |
| *Syncerus caffer* | 592666 |
| *Tragelaphus oryx* | 600000 |
| *Tragelaphus derbianus* | 907000 |
| *Giraffa camelopardalis* | 964654.7 |
| *Diceros bicornis* | 995940.5 |
| *Hippopotamus amphibius* | 1536310 |
| *Ceratotherium simum* | 2285939 |
| *Loxodonta africana* | 3824540 |
