# Supplementary material for: Pyrodiversity interacts with rainfall to increase bird and mammal richness in African savannas
Source: Ecol Lett. 2018 Feb 14;21(4):557–67. doi: 10.1111/ele.12921 (PMC5888149; doi:10.1111/ele.12921)
Supplement: Supplementary file 5 [file ELE-21-557-s005.docx]

SUPPORTING INFORMATION APPENDIX 3: Savannah bird species included in the analysis: to be included, each species must be regularly found within savannah, though it may well primarily occur in other biomes.

| Scientific Name | English Name | Family |
| --- | --- | --- |
| Struthio camelus | Common Ostrich | Struthionidae |
| Struthio molybdophanes | Somali Ostrich | Struthionidae |
| Numida meleagris | Helmeted Guineafowl | Numididae |
| Guttera pucherani | Crested Guineafowl | Numididae |
| Acryllium vulturinum | Vulturine Guineafowl | Numididae |
| Peliperdix coqui | Coqui Francolin | Phasianidae |
| Peliperdix albogularis | White-throated Francolin | Phasianidae |
| Peliperdix schlegelii | Schlegel's Francolin | Phasianidae |
| Scleroptila streptophora | Ring-necked Francolin | Phasianidae |
| Scleroptila levaillantii | Red-winged Francolin | Phasianidae |
| Scleroptila finschi | Finsch's Francolin | Phasianidae |
| Scleroptila shelleyi | Shelley's Francolin | Phasianidae |
| Scleroptila levaillantoides | Orange River Francolin | Phasianidae |
| Pternistis hildebrandti | Hildebrandt's Francolin | Phasianidae |
| Pternistis bicalcaratus | Double-spurred Francolin | Phasianidae |
| Pternistis icterorhynchus | Heuglin's Francolin | Phasianidae |
| Pternistis clappertoni | Clapperton's Francolin | Phasianidae |
| Pternistis hartlaubi | Hartlaub's Spurfowl | Phasianidae |
| Pternistis adspersus | Red-billed Spurfowl | Phasianidae |
| Pternistis natalensis | Natal Spurfowl | Phasianidae |
| Pternistis leucoscepus | Yellow-necked Spurfowl | Phasianidae |
| Pternistis rufopictus | Grey-breasted Spurfowl | Phasianidae |
| Pternistis swainsonii | Swainson's Spurfowl | Phasianidae |
| Coturnix coturnix | Common Quail | Phasianidae |
| Coturnix delegorguei | Harlequin Quail | Phasianidae |
| Excalfactoria adansonii | Blue Quail | Phasianidae |
| Plectropterus gambensis | Spur-winged Goose | Anatidae |
| Alopochen aegyptiaca | Egyptian Goose | Anatidae |
| Ciconia nigra | Black Stork | Ciconiidae |
| Ciconia abdimii | Abdim's Stork | Ciconiidae |
| Leptoptilos crumeniferus | Marabou Stork | Ciconiidae |
| Threskiornis aethiopicus | African Sacred Ibis | Threskiornithidae |
| Geronticus calvus | Southern Bald Ibis | Threskiornithidae |
| Bostrychia hagedash | Hadada Ibis | Threskiornithidae |
| Bubulcus ibis | Western Cattle Egret | Ardeidae |
| Ardea cinerea | Grey Heron | Ardeidae |
| Ardea melanocephala | Black-headed Heron | Ardeidae |
| Sagittarius serpentarius | Secretarybird | Sagittariidae |
| Elanus caeruleus | Black-winged Kite | Accipitridae |
| Chelictinia riocourii | Scissor-tailed Kite | Accipitridae |
| Milvus aegyptius | Yellow-billed Kite | Accipitridae |
| Haliaeetus vocifer | African Fish Eagle | Accipitridae |
| Gypohierax angolensis | Palm-nut Vulture | Accipitridae |
| Gypaetus barbatus | Bearded Vulture | Accipitridae |
| Neophron percnopterus | Egyptian Vulture | Accipitridae |
| Necrosyrtes monachus | Hooded Vulture | Accipitridae |
| Gyps africanus | White-backed Vulture | Accipitridae |
| Gyps rueppellii | Rüppell's Vulture | Accipitridae |
| Gyps fulvus | Griffon Vulture | Accipitridae |
| Gyps coprotheres | Cape Vulture | Accipitridae |
| Trigonoceps occipitalis | White-headed Vulture | Accipitridae |
| Torgos tracheliotus | Lappet-faced Vulture | Accipitridae |
| Circaetus beaudouini | Beaudouin's Snake Eagle | Accipitridae |
| Circaetus pectoralis | Black-chested Snake Eagle | Accipitridae |
| Circaetus cinereus | Brown Snake Eagle | Accipitridae |
| Circaetus fasciolatus | Southern Banded Snake Eagle | Accipitridae |
| Circaetus cinerascens | Western Banded Snake Eagle | Accipitridae |
| Terathopius ecaudatus | Bateleur | Accipitridae |
| Polyboroides typus | African Harrier-Hawk | Accipitridae |
| Melierax metabates | Dark Chanting Goshawk | Accipitridae |
| Melierax poliopterus | Eastern Chanting Goshawk | Accipitridae |
| Melierax canorus | Pale Chanting Goshawk | Accipitridae |
| Micronisus gabar | Gabar Goshawk | Accipitridae |
| Accipiter minullus | Little Sparrowhawk | Accipitridae |
| Accipiter ovampensis | Ovambo Sparrowhawk | Accipitridae |
| Kaupifalco monogrammicus | Lizard Buzzard | Accipitridae |
| Butastur rufipennis | Grasshopper Buzzard | Accipitridae |
| Buteo auguralis | Red-necked Buzzard | Accipitridae |
| Buteo augur | Augur Buzzard | Accipitridae |
| Buteo rufofuscus | Jackal Buzzard | Accipitridae |
| Aquila rapax | Tawny Eagle | Accipitridae |
| Aquila verreauxii | Verreaux's Eagle | Accipitridae |
| Aquila fasciata | Bonelli's Eagle | Accipitridae |
| Aquila spilogaster | African Hawk-Eagle | Accipitridae |
| Hieraaetus wahlbergi | Wahlberg's Eagle | Accipitridae |
| Hieraaetus pennatus | Booted Eagle | Accipitridae |
| Polemaetus bellicosus | Martial Eagle | Accipitridae |
| Lophaetus occipitalis | Long-crested Eagle | Accipitridae |
| Polihierax semitorquatus | Pygmy Falcon | Falconidae |
| Falco tinnunculus | Common Kestrel | Falconidae |
| Falco rupicolus | Rock Kestrel | Falconidae |
| Falco rupicoloides | Greater Kestrel | Falconidae |
| Falco alopex | Fox Kestrel | Falconidae |
| Falco ardosiaceus | Grey Kestrel | Falconidae |
| Falco dickinsoni | Dickinson's Kestrel | Falconidae |
| Falco chicquera | Red-necked Falcon | Falconidae |
| Falco cuvierii | African Hobby | Falconidae |
| Falco biarmicus | Lanner Falcon | Falconidae |
| Falco peregrinus | Peregrine Falcon | Falconidae |
| Falco pelegrinoides | Barbary Falcon | Falconidae |
| Falco fasciinucha | Taita Falcon | Falconidae |
| Ardeotis arabs | Arabian Bustard | Otididae |
| Ardeotis kori | Kori Bustard | Otididae |
| Neotis ludwigii | Ludwig's Bustard | Otididae |
| Neotis denhami | Denham's Bustard | Otididae |
| Neotis heuglinii | Heuglin's Bustard | Otididae |
| Neotis nuba | Nubian Bustard | Otididae |
| Eupodotis senegalensis | White-bellied Bustard | Otididae |
| Eupodotis barrowii | Barrow's Korhaan | Otididae |
| Eupodotis humilis | Little Brown Bustard | Otididae |
| Lophotis savilei | Savile's Bustard | Otididae |
| Lophotis gindiana | Buff-crested Bustard | Otididae |
| Eupodotis afraoides | Northern Black Korhaan | Otididae |
| Lophotis ruficrista | Red-crested Korhaan | Otididae |
| Lissotis melanogaster | Black-bellied Bustard | Otididae |
| Lissotis hartlaubii | Hartlaub's Bustard | Otididae |
| Crex egregia | African Crake | Rallidae |
| Balearica regulorum | Grey Crowned Crane | Gruidae |
| Balearica pavonina | Black Crowned Crane | Gruidae |
| Turnix sylvaticus | Kurrichane Buttonquail | Turnicidae |
| Turnix nanus | Black-rumped Buttonquail | Turnicidae |
| Ortyxelos meiffrenii | Quail-plover | Turnicidae |
| Burhinus senegalensis | Senegal Thick-knee | Burhinidae |
| Burhinus vermiculatus | Water Thick-knee | Burhinidae |
| Burhinus capensis | Spotted Thick-knee | Burhinidae |
| Vanellus spinosus | Spur-winged Lapwing | Charadriidae |
| Vanellus tectus | Black-headed Lapwing | Charadriidae |
| Vanellus albiceps | White-crowned Lapwing | Charadriidae |
| Vanellus lugubris | Senegal Lapwing | Charadriidae |
| Vanellus melanopterus | Black-winged Lapwing | Charadriidae |
| Vanellus coronatus | Crowned Lapwing | Charadriidae |
| Vanellus senegallus | African Wattled Lapwing | Charadriidae |
| Vanellus superciliosus | Brown-chested Lapwing | Charadriidae |
| Cursorius temminckii | Temminck's Courser | Glareolidae |
| Rhinoptilus africanus | Double-banded Courser | Glareolidae |
| Rhinoptilus cinctus | Three-banded Courser | Glareolidae |
| Rhinoptilus chalcopterus | Bronze-winged Courser | Glareolidae |
| Pterocles exustus | Chestnut-bellied Sandgrouse | Pteroclididae |
| Pterocles gutturalis | Yellow-throated Sandgrouse | Pteroclididae |
| Pterocles decoratus | Black-faced Sandgrouse | Pteroclididae |
| Pterocles burchelli | Burchell's Sandgrouse | Pteroclididae |
| Columba guinea | Speckled Pigeon | Columbidae |
| Streptopelia lugens | Dusky Turtle Dove | Columbidae |
| Streptopelia roseogrisea | African Collared Dove | Columbidae |
| Streptopelia decipiens | Mourning Collared Dove | Columbidae |
| Streptopelia semitorquata | Red-eyed Dove | Columbidae |
| Streptopelia capicola | Ring-necked Dove | Columbidae |
| Spilopelia senegalensis | Laughing Dove | Columbidae |
| Turtur chalcospilos | Emerald-spotted Wood Dove | Columbidae |
| Turtur abyssinicus | Black-billed Wood Dove | Columbidae |
| Oena capensis | Namaqua Dove | Columbidae |
| Treron waalia | Bruce's Green Pigeon | Columbidae |
| Treron calvus | African Green Pigeon | Columbidae |
| Psittacula krameri | Rose-ringed Parakeet | Psittacidae |
| Agapornis pullarius | Red-headed Lovebird | Psittacidae |
| Agapornis taranta | Black-winged Lovebird | Psittacidae |
| Agapornis roseicollis | Rosy-faced Lovebird | Psittacidae |
| Agapornis fischeri | Fischer's Lovebird | Psittacidae |
| Agapornis personatus | Yellow-collared Lovebird | Psittacidae |
| Agapornis lilianae | Lilian's Lovebird | Psittacidae |
| Agapornis nigrigenis | Black-cheeked Lovebird | Psittacidae |
| Poicephalus fuscicollis | Brown-necked Parrot | Psittacidae |
| Poicephalus robustus | Cape Parrot | Psittacidae |
| Poicephalus suahelicus | Brown-necked Parrot | Psittacidae |
| Poicephalus meyeri | Meyer's Parrot | Psittacidae |
| Poicephalus rueppellii | Rüppell's Parrot | Psittacidae |
| Poicephalus cryptoxanthus | Brown-headed Parrot | Psittacidae |
| Poicephalus crassus | Niam-niam Parrot | Psittacidae |
| Poicephalus rufiventris | Red-bellied Parrot | Psittacidae |
| Poicephalus senegalus | Senegal Parrot | Psittacidae |
| Poicephalus flavifrons | Yellow-fronted Parrot | Psittacidae |
| Tauraco schalowi | Schalow's Turaco | Musophagidae |
| Tauraco leucolophus | White-crested Turaco | Musophagidae |
| Tauraco porphyreolophus | Purple-crested Turaco | Musophagidae |
| Musophaga violacea | Violet Turaco | Musophagidae |
| Musophaga rossae | Ross's Turaco | Musophagidae |
| Corythaixoides concolor | Grey Go-away-bird | Musophagidae |
| Corythaixoides personatus | Bare-faced Go-away-bird | Musophagidae |
| Corythaixoides leucogaster | White-bellied Go-away-bird | Musophagidae |
| Centropus senegalensis | Senegal Coucal | Cuculidae |
| Centropus superciliosus | White-browed Coucal | Cuculidae |
| Centropus burchellii | Burchell's Coucal | Cuculidae |
| Centropus grillii | Black Coucal | Cuculidae |
| Clamator glandarius | Great Spotted Cuckoo | Cuculidae |
| Clamator levaillantii | Levaillant's Cuckoo | Cuculidae |
| Clamator jacobinus | Jacobin Cuckoo | Cuculidae |
| Chrysococcyx caprius | Dideric Cuckoo | Cuculidae |
| Chrysococcyx klaas | Klaas's Cuckoo | Cuculidae |
| Cuculus clamosus | Black Cuckoo | Cuculidae |
| Cuculus gularis | African Cuckoo | Cuculidae |
| Tyto alba | Western Barn Owl | Tytonidae |
| Tyto capensis | African Grass Owl | Tytonidae |
| Otus senegalensis | African Scops Owl | Strigidae |
| Ptilopsis leucotis | Northern White-faced Owl | Strigidae |
| Ptilopsis granti | Southern White-faced Owl | Strigidae |
| Bubo ascalaphus | Pharaoh Eagle-Owl | Strigidae |
| Bubo capensis | Cape Eagle-Owl | Strigidae |
| Bubo africanus | Spotted Eagle-Owl | Strigidae |
| Bubo cinerascens | Greyish Eagle-Owl | Strigidae |
| Bubo lacteus | Verreaux's Eagle-Owl | Strigidae |
| Glaucidium perlatum | Pearl-spotted Owlet | Strigidae |
| Glaucidium capense | African Barred Owlet | Strigidae |
| Glaucidium scheffleri | Scheffler's Owlet | Strigidae |
| Asio capensis | Marsh Owl | Strigidae |
| Caprimulgus fraenatus | Sombre Nightjar | Caprimulgidae |
| Caprimulgus rufigena | Rufous-cheeked Nightjar | Caprimulgidae |
| Caprimulgus donaldsoni | Donaldson-Smith's Nightjar | Caprimulgidae |
| Caprimulgus nigriscapularis | Black-shouldered Nightjar | Caprimulgidae |
| Caprimulgus pectoralis | Fiery-necked Nightjar | Caprimulgidae |
| Caprimulgus natalensis | Swamp Nightjar | Caprimulgidae |
| Caprimulgus solala | Nechisar Nightjar | Caprimulgidae |
| Caprimulgus inornatus | Plain Nightjar | Caprimulgidae |
| Caprimulgus stellatus | Star-spotted Nightjar | Caprimulgidae |
| Caprimulgus tristigma | Freckled Nightjar | Caprimulgidae |
| Caprimulgus climacurus | Long-tailed Nightjar | Caprimulgidae |
| Caprimulgus clarus | Slender-tailed Nightjar | Caprimulgidae |
| Caprimulgus fossii | Square-tailed Nightjar | Caprimulgidae |
| Macrodipteryx longipennis | Standard-winged Nightjar | Caprimulgidae |
| Macrodipteryx vexillarius | Pennant-winged Nightjar | Caprimulgidae |
| Telacanthura ussheri | Mottled Spinetail | Apodidae |
| Neafrapus boehmi | Böhm's Spinetail | Apodidae |
| Cypsiurus parvus | African Palm Swift | Apodidae |
| Tachymarptis melba | Alpine Swift | Apodidae |
| Tachymarptis aequatorialis | Mottled Swift | Apodidae |
| Apus niansae | Nyanza Swift | Apodidae |
| Apus pallidus | Pallid Swift | Apodidae |
| Apus barbatus | African Black Swift | Apodidae |
| Apus affinis | Little Swift | Apodidae |
| Apus horus | Horus Swift | Apodidae |
| Apus toulsoni | Loanda Swift | Apodidae |
| Apus caffer | White-rumped Swift | Apodidae |
| Colius striatus | Speckled Mousebird | Coliidae |
| Colius leucocephalus | White-headed Mousebird | Coliidae |
| Colius castanotus | Red-backed Mousebird | Coliidae |
| Colius colius | White-backed Mousebird | Coliidae |
| Urocolius macrourus | Blue-naped Mousebird | Coliidae |
| Urocolius indicus | Red-faced Mousebird | Coliidae |
| Coracias naevius | Purple Roller | Coraciidae |
| Coracias spatulatus | Racket-tailed Roller | Coraciidae |
| Coracias caudatus | Lilac-breasted Roller | Coraciidae |
| Coracias abyssinicus | Abyssinian Roller | Coraciidae |
| Coracias cyanogaster | Blue-bellied Roller | Coraciidae |
| Halcyon leucocephala | Grey-headed Kingfisher | Alcedinidae |
| Halcyon albiventris | Brown-hooded Kingfisher | Alcedinidae |
| Halcyon chelicuti | Striped Kingfisher | Alcedinidae |
| Halcyon senegalensis | Woodland Kingfisher | Alcedinidae |
| Merops hirundineus | Swallow-tailed Bee-eater | Meropidae |
| Merops pusillus | Little Bee-eater | Meropidae |
| Merops bulocki | Red-throated Bee-eater | Meropidae |
| Merops bullockoides | White-fronted Bee-eater | Meropidae |
| Merops revoilii | Somali Bee-eater | Meropidae |
| Merops albicollis | White-throated Bee-eater | Meropidae |
| Merops boehmi | Böhm's Bee-eater | Meropidae |
| Merops malimbicus | Rosy Bee-eater | Meropidae |
| Merops nubicus | Northern Carmine Bee-eater | Meropidae |
| Merops nubicoides | Southern Carmine Bee-eater | Meropidae |
| Upupa africana | African Hoopoe | Upupidae |
| Phoeniculus purpureus | Green Wood Hoopoe | Phoeniculidae |
| Phoeniculus somaliensis | Black-billed Wood Hoopoe | Phoeniculidae |
| Phoeniculus damarensis | Violet Wood Hoopoe | Phoeniculidae |
| Phoeniculus granti | Grant's Wood Hoopoe | Phoeniculidae |
| Rhinopomastus aterrimus | Black Scimitarbill | Phoeniculidae |
| Rhinopomastus cyanomelas | Common Scimitarbill | Phoeniculidae |
| Rhinopomastus minor | Abyssinian Scimitarbill | Phoeniculidae |
| Tockus alboterminatus | Crowned Hornbill | Bucerotidae |
| Tockus bradfieldi | Bradfield's Hornbill | Bucerotidae |
| Tockus hemprichii | Hemprich's Hornbill | Bucerotidae |
| Tockus pallidirostris | Pale-billed Hornbill | Bucerotidae |
| Tockus nasutus | African Grey Hornbill | Bucerotidae |
| Tockus monteiri | Monteiro's Hornbill | Bucerotidae |
| Tockus erythrorhynchus | Northern Red-billed Hornbill | Bucerotidae |
| Tockus damarensis | Damara Red-billed Hornbill | Bucerotidae |
| Tockus rufirostris | Southern Red-billed Hornbill | Bucerotidae |
| Tockus ruahae | Tanzanian Red-billed Hornbill | Bucerotidae |
| Tockus kempi | Western Red-billed Hornbill | Bucerotidae |
| Tockus leucomelas | Southern Yellow-billed Hornbill | Bucerotidae |
| Tockus flavirostris | Northern Yellow-billed Hornbill | Bucerotidae |
| Tockus deckeni | Von der Decken's Hornbill | Bucerotidae |
| Tockus jacksoni | Jackson's Hornbill | Bucerotidae |
| Bucorvus abyssinicus | Abyssinian Ground Hornbill | Bucorvidae |
| Bucorvus leadbeateri | Southern Ground Hornbill | Bucorvidae |
| Stactolaema anchietae | Anchieta's Barbet | Lybiidae |
| Pogoniulus bilineatus | Yellow-rumped Tinkerbird | Lybiidae |
| Pogoniulus pusillus | Red-fronted Tinkerbird | Lybiidae |
| Pogoniulus chrysoconus | Yellow-fronted Tinkerbird | Lybiidae |
| Tricholaema diademata | Red-fronted Barbet | Lybiidae |
| Tricholaema frontata | Miombo Pied Barbet | Lybiidae |
| Tricholaema leucomelas | Acacia Pied Barbet | Lybiidae |
| Tricholaema lacrymosa | Spot-flanked Barbet | Lybiidae |
| Tricholaema melanocephala | Black-throated Barbet | Lybiidae |
| Lybius undatus | Banded Barbet | Lybiidae |
| Lybius vieilloti | Vieillot's Barbet | Lybiidae |
| Lybius leucocephalus | White-headed Barbet | Lybiidae |
| Lybius chaplini | Chaplin's Barbet | Lybiidae |
| Lybius rubrifacies | Red-faced Barbet | Lybiidae |
| Lybius guifsobalito | Black-billed Barbet | Lybiidae |
| Lybius melanopterus | Brown-breasted Barbet | Lybiidae |
| Lybius minor | Black-backed Barbet | Lybiidae |
| Lybius bidentatus | Double-toothed Barbet | Lybiidae |
| Lybius rolleti | Black-breasted Barbet | Lybiidae |
| Trachyphonus vaillantii | Crested Barbet | Lybiidae |
| Trachyphonus erythrocephalus | Red-and-yellow Barbet | Lybiidae |
| Trachyphonus margaritatus | Yellow-breasted Barbet | Lybiidae |
| Trachyphonus darnaudii | D'Arnaud's Barbet | Lybiidae |
| Trachyphonus usambiro | **Usambiro** Barbet | Lybiidae |
| Prodotiscus zambesiae | Green-backed Honeybird | Indicatoridae |
| Indicator minor | Lesser Honeyguide | Indicatoridae |
| Indicator indicator | Greater Honeyguide | Indicatoridae |
| Jynx ruficollis | Red-throated Wryneck | Picidae |
| Campethera punctuligera | Fine-spotted Woodpecker | Picidae |
| Campethera bennettii | Bennett's Woodpecker | Picidae |
| Campethera scriptoricauda | Speckle-throated Woodpecker | Picidae |
| Campethera nubica | Nubian Woodpecker | Picidae |
| Campethera cailliautii | Green-backed Woodpecker | Picidae |
| Dendropicos elachus | Little Grey Woodpecker | Picidae |
| Dendropicos poecilolaemus | Speckle-breasted Woodpecker | Picidae |
| Dendropicos obsoletus | Brown-backed Woodpecker | Picidae |
| Dendropicos fuscescens | Cardinal Woodpecker | Picidae |
| Dendropicos stierlingi | Stierling's Woodpecker | Picidae |
| Dendropicos namaquus | Bearded Woodpecker | Picidae |
| Dendropicos goertae | African Grey Woodpecker | Picidae |
| Dendropicos spodocephalus | Eastern Grey Woodpecker | Picidae |
| Batis molitor | Chinspot Batis | Platysteiridae |
| Batis senegalensis | Senegal Batis | Platysteiridae |
| Batis orientalis | Grey-headed Batis | Platysteiridae |
| Batis soror | Pale Batis | Platysteiridae |
| Batis pririt | Pririt Batis | Platysteiridae |
| Batis minor | Eastern Black-headed Batis | Platysteiridae |
| Batis erlangeri | Western Black-headed Batis | Platysteiridae |
| Batis perkeo | Pygmy Batis | Platysteiridae |
| Batis minulla | Angola Batis | Platysteiridae |
| Lanioturdus torquatus | White-tailed Shrike | Platysteiridae |
| Platysteira cyanea | Brown-throated Wattle-eye | Platysteiridae |
| Platysteira peltata | Black-throated Wattle-eye | Platysteiridae |
| Prionops plumatus | White-crested Helmetshrike | Prionopidae |
| Prionops poliolophus | Grey-crested Helmetshrike | Prionopidae |
| Malaconotus blanchoti | Grey-headed Bushshrike | Malaconotidae |
| Chlorophoneus sulfureopectus | Orange-breasted Bushshrike | Malaconotidae |
| Telophorus cruentus | Rosy-patched Shrike | Malaconotidae |
| Telophorus zeylonus | Bokmakierie | Malaconotidae |
| Rhodophoneus cruentus | Rosy-patched Bushshrike | Malaconotidae |
| Tchagra anchietae | Anchieta's Tchagra | Malaconotidae |
| Tchagra australis | Brown-crowned Tchagra | Malaconotidae |
| Tchagra jamesi | Three-streaked Tchagra | Malaconotidae |
| Tchagra tchagra | Southern Tchagra | Malaconotidae |
| Tchagra senegalus | Black-crowned Tchagra | Malaconotidae |
| Dryoscopus cubla | Black-backed Puffback | Malaconotidae |
| Dryoscopus gambensis | Northern Puffback | Malaconotidae |
| Dryoscopus pringlii | Pringle's Puffback | Malaconotidae |
| Laniarius leucorhynchus | Lowland Sooty Boubou | Malaconotidae |
| Laniarius funebris | Slate-colored Boubou | Malaconotidae |
| Laniarius ruficeps | Red-naped Bushshrike | Malaconotidae |
| Laniarius erlangeri | Somali Boubou | Malaconotidae |
| Laniarius sublacteus | East Coast Boubou | Malaconotidae |
| Laniarius ferrugineus | Southern Boubou | Malaconotidae |
| Laniarius turatii | Turati's Boubou | Malaconotidae |
| Laniarius atrococcineus | Crimson-breasted Shrike | Malaconotidae |
| Nilaus afer | Brubru | Malaconotidae |
| Campephaga flava | Black Cuckooshrike | Campephagidae |
| Corvinella corvina | Yellow-billed Shrike | Laniidae |
| Urolestes melanoleucus | Magpie Shrike | Laniidae |
| Eurocephalus anguitimens | Southern White-crowned Shrike | Laniidae |
| Eurocephalus ruppelli | Northern White-crowned Shrike | Laniidae |
| Lanius souzae | Souza's Shrike | Laniidae |
| Lanius gubernator | Emin's Shrike | Laniidae |
| Lanius mackinnoni | Mackinnon's Shrike | Laniidae |
| Lanius meridionalis | Southern Grey Shrike | Laniidae |
| Lanius excubitoroides | Grey-backed Fiscal | Laniidae |
| Lanius cabanisi | Long-tailed Fiscal | Laniidae |
| Lanius dorsalis | Taita Fiscal | Laniidae |
| Lanius somalicus | Somali Fiscal | Laniidae |
| Lanius collaris | Common Fiscal | Laniidae |
| Lanius humeralis | Northern Fiscal | Laniidae |
| Lanius marwitzi | Uhehe Fiscal | Laniidae |
| Oriolus auratus | African Golden Oriole | Oriolidae |
| Oriolus brachyrynchus | Western Black-headed Oriole | Oriolidae |
| Dicrurus adsimilis | Fork-tailed Drongo | Dicruridae |
| Terpsiphone viridis | African Paradise Flycatcher | Monarchidae |
| Ptilostomus afer | Piapiac | Corvidae |
| Corvus capensis | Cape Crow | Corvidae |
| Corvus albus | Pied Crow | Corvidae |
| Corvus albicollis | White-necked Raven | Corvidae |
| Parus guineensis | White-shouldered Black Tit | Paridae |
| Parus leucomelas | White-winged Black Tit | Paridae |
| Parus carpi | Carp's Black Tit | Paridae |
| Parus niger | Southern Black Tit | Paridae |
| Parus albiventris | White-bellied Tit | Paridae |
| Parus rufiventris | Rufous-bellied Tit | Paridae |
| Parus pallidiventris | Cinnamon-breasted Tit | Paridae |
| Parus fringillinus | Red-throated Tit | Paridae |
| Parus thruppi | Acacia Tit | Paridae |
| Parus griseiventris | Miombo Tit | Paridae |
| Parus cinerascens | Ashy Tit | Paridae |
| Parus afer | Grey Tit | Paridae |
| Anthoscopus punctifrons | Sennar Penduline Tit | Remizidae |
| Anthoscopus parvulus | Yellow Penduline Tit | Remizidae |
| Anthoscopus musculus | Mouse-colored Penduline Tit | Remizidae |
| Anthoscopus caroli | Grey Penduline Tit | Remizidae |
| Anthoscopus sylviella | Buff-bellied Penduline-tit | Remizidae |
| Anthoscopus minutus | Cape Penduline Tit | Remizidae |
| Mirafra cantillans | Singing Bush Lark | Alaudidae |
| Mirafra passerina | Monotonous Lark | Alaudidae |
| Mirafra albicauda | White-tailed Lark | Alaudidae |
| Mirafra cheniana | Melodious Lark | Alaudidae |
| Mirafra williamsi | Williams's Lark | Alaudidae |
| Mirafra pulpa | Friedmann's Lark | Alaudidae |
| Mirafra africana | Rufous-naped Lark | Alaudidae |
| Mirafra sharpii | Somali Lark | Alaudidae |
| Mirafra hypermetra | Red-winged Lark | Alaudidae |
| Mirafra somalica | Somali Lark | Alaudidae |
| Mirafra rufocinnamomea | Flappet Lark | Alaudidae |
| Mirafra fasciolata | Eastern Clapper Lark | Alaudidae |
| Mirafra collaris | Collared Lark | Alaudidae |
| Mirafra rufa | Rusty Bush Lark | Alaudidae |
| Mirafra gilletti | Gillett's Lark | Alaudidae |
| Heteromirafra ruddi | Rudd's Lark | Alaudidae |
| Heteromirafra archeri | Archer's Lark | Alaudidae |
| Heteromirafra sidamoensis | Sidamo Lark | Alaudidae |
| Calendulauda africanoides | Fawn-colored Lark | Alaudidae |
| Calendulauda alopex | Foxy Lark | Alaudidae |
| Calendulauda poecilosterna | Pink-breasted Lark | Alaudidae |
| Calendulauda sabota | Sabota Lark | Alaudidae |
| Mirafra naevia | Bradfield's Lark | Alaudidae |
| Certhilauda semitorquata | Eastern Long-billed Lark | Alaudidae |
| Certhilauda chuana | Short-clawed Lark | Alaudidae |
| Pinarocorys nigricans | Dusky Lark | Alaudidae |
| Pinarocorys erythropygia | Rufous-rumped Lark | Alaudidae |
| Chersomanes albofasciata | Spike-heeled Lark | Alaudidae |
| Chersomanes beesleyi | Beesley's Lark | Alaudidae |
| Calandrella cinerea | Red-capped Lark | Alaudidae |
| Calandrella somalica | Somali Short-toed Lark | Alaudidae |
| Calandrella athensis | Athi Short-toed Lark | Alaudidae |
| Spizocorys conirostris | Pink-billed Lark | Alaudidae |
| Spizocorys starki | Stark's Lark | Alaudidae |
| Pseudalaemon fremantlii | Short-tailed Lark | Alaudidae |
| Galerida modesta | Sun Lark | Alaudidae |
| Eremopterix leucotis | Chestnut-backed Sparrow-Lark | Alaudidae |
| Eremopterix verticalis | Grey-backed Sparrow-Lark | Alaudidae |
| Eremopterix leucopareia | Fischer's Sparrow-Lark | Alaudidae |
| Pycnonotus nigricans | African Red-eyed Bulbul | Pycnonotidae |
| Pycnonotus capensis | Cape Bulbul | Pycnonotidae |
| Pycnonotus barbatus | Common Bulbul | Pycnonotidae |
| Pycnonotus somaliensis | Somali Bulbul | Pycnonotidae |
| Pycnonotus dodsoni | Dodson's Bulbul | Pycnonotidae |
| Pycnonotus tricolor | Dark-capped Bulbul | Pycnonotidae |
| Chlorocichla flaviventris | Yellow-bellied Greenbul | Pycnonotidae |
| Phyllastrephus terrestris | Terrestrial Brownbul | Pycnonotidae |
| Phyllastrephus strepitans | Northern Brownbul | Pycnonotidae |
| Psalidoprocne albiceps | White-headed Saw-wing | Hirundinidae |
| Psalidoprocne chalybea | Shari Sawwing | Hirundinidae |
| Psalidoprocne mangbettorum | Mangbettu Sawwing | Hirundinidae |
| Psalidoprocne oleaginea | Ethiopian saw-wing | Hirundinidae |
| Pseudhirundo griseopyga | Grey-rumped Swallow | Hirundinidae |
| Riparia paludicola | Brown-throated Martin | Hirundinidae |
| Riparia cincta | Banded Martin | Hirundinidae |
| Hirundo lucida | Red-chested Swallow | Hirundinidae |
| Hirundo angolensis | Angola Swallow | Hirundinidae |
| Hirundo albigularis | White-throated Swallow | Hirundinidae |
| Hirundo aethiopica | Ethiopian Swallow | Hirundinidae |
| Hirundo smithii | Wire-tailed Swallow | Hirundinidae |
| Hirundo leucosoma | Pied-winged Swallow | Hirundinidae |
| Hirundo nigrorufa | Black-and-rufous Swallow | Hirundinidae |
| Hirundo dimidiata | Pearl-breasted Swallow | Hirundinidae |
| Ptyonoprogne fuligula | Rock Martin | Hirundinidae |
| Cecropis cucullata | Greater Striped Swallow | Hirundinidae |
| Cecropis abyssinica | Lesser Striped Swallow | Hirundinidae |
| Cecropis semirufa | Red-breasted Swallow | Hirundinidae |
| Cecropis senegalensis | Mosque Swallow | Hirundinidae |
| Cecropis daurica | Red-rumped Swallow | Hirundinidae |
| Cecropis domicella | West African Swallow | Hirundinidae |
| Petrochelidon rufigula | Red-throated Cliff Swallow | Hirundinidae |
| Petrochelidon preussi | Preuss's Cliff Swallow | Hirundinidae |
| Petrochelidon spilodera | South African Cliff Swallow | Hirundinidae |
| Cisticola lateralis | Whistling Cisticola | Cisticolidae |
| Cisticola woosnami | Trilling Cisticola | Cisticolidae |
| Cisticola anonymus | Chattering Cisticola | Cisticolidae |
| Cisticola bulliens | Bubbling Cisticola | Cisticolidae |
| Cisticola aberrans | Lazy Cisticola | Cisticolidae |
| Cisticola emini | Rock-loving Cisticola | Cisticolidae |
| Cisticola chiniana | Rattling Cisticola | Cisticolidae |
| Cisticola cinereolus | Ashy Cisticola | Cisticolidae |
| Cisticola rufilatus | Tinkling Cisticola | Cisticolidae |
| Cisticola subruficapilla | Grey-backed Cisticola | Cisticolidae |
| Cisticola lais | Wailing Cisticola | Cisticolidae |
| Cisticola marginatus | Winding Cisticola | Cisticolidae |
| Cisticola haematocephalus | Coastal Cisticola | Cisticolidae |
| Cisticola tinniens | Levaillant's Cisticola | Cisticolidae |
| Cisticola robustus | Stout Cisticola | Cisticolidae |
| Cisticola angolensis | Angola C**isticola** | Cisticolidae |
| Cisticola natalensis | Croaking Cisticola | Cisticolidae |
| Cisticola ruficeps | Red-pate Cisticola | Cisticolidae |
| Cisticola dorsti | Dorst's Cisticola | Cisticolidae |
| Cisticola nana | Tiny Cisticola | Cisticolidae |
| Cisticola brachypterus | Short-winged Cisticola | Cisticolidae |
| Cisticola rufus | Rufous Cisticola | Cisticolidae |
| Cisticola troglodytes | Foxy Cisticola | Cisticolidae |
| Cisticola fulvicapilla | Neddicky | Cisticolidae |
| Cisticola angusticauda | Long-tailed Cisticola | Cisticolidae |
| Cisticola melanurus | Black-tailed Cisticola | Cisticolidae |
| Cisticola juncidis | Zitting Cisticola | Cisticolidae |
| Cisticola aridulus | Desert Cisticola | Cisticolidae |
| Cisticola eximius | Black-backed Cisticola | Cisticolidae |
| Cisticola dambo | Dambo Cisticola | Cisticolidae |
| Cisticola brunnescens | Pectoral-patch Cisticola | Cisticolidae |
| Malcorus pectoralis | Rufous-eared Warbler | Cisticolidae |
| Prinia subflava | Tawny-flanked Prinia | Cisticolidae |
| Prinia somalica | Pale Prinia | Cisticolidae |
| Prinia flavicans | Black-chested Prinia | Cisticolidae |
| Prinia bairdii | Banded Prinia | Cisticolidae |
| Prinia melanops | Black-faced Prinia | Cisticolidae |
| Spiloptila clamans | Cricket Longtail | Cisticolidae |
| Apalis flavida | Yellow-breasted Apalis | Cisticolidae |
| Apalis karamojae | Karamoja Apalis | Cisticolidae |
| Urorhipis rufifrons | Red-fronted Apalis | Cisticolidae |
| Camaroptera brachyura | Green-backed Camaroptera | Cisticolidae |
| Camaroptera brevicaudata | Grey-backed Camaroptera | Cisticolidae |
| Calamonastes simplex | Grey Wren-Warbler | Cisticolidae |
| Calamonastes undosus | Miombo Wren-Warbler | Cisticolidae |
| Calamonastes stierlingi | Stierling's Wren-Warbler | Cisticolidae |
| Calamonastes fasciolatus | Barred Wren-Warbler | Cisticolidae |
| Euryptila subcinnamomea | Cinnamon-breasted Warbler | Cisticolidae |
| Eremomela icteropygialis | Yellow-bellied Eremomela | Cisticolidae |
| Eremomela salvadorii | Salvadori's Eremomela | Cisticolidae |
| Eremomela flavicrissalis | Yellow-vented Eremomela | Cisticolidae |
| Eremomela pusilla | Senegal Eremomela | Cisticolidae |
| Eremomela canescens | Green-backed Eremomela | Cisticolidae |
| Eremomela scotops | Green-capped Eremomela | Cisticolidae |
| Eremomela usticollis | Burnt-necked Eremomela | Cisticolidae |
| Eremomela atricollis | Black-necked Eremomela | Cisticolidae |
| Sylvietta brachyura | Northern Crombec | Family Incertae Sedis |
| Sylvietta whytii | Red-faced Crombec | Family Incertae Sedis |
| Sylvietta philippae | Philippa's Crombec | Family Incertae Sedis |
| Sylvietta rufescens | Long-billed Crombec | Family Incertae Sedis |
| Sylvietta isabellina | Somali Crombec | Family Incertae Sedis |
| Sylvietta ruficapilla | Red-capped Crombec | Family Incertae Sedis |
| Sylvietta leucophrys | White-browed Crombec | Family Incertae Sedis |
| Turdoides fulva | Fulvous Babbler | Timaliidae |
| Turdoides aylmeri | Scaly Chatterer | Timaliidae |
| Turdoides rubiginosa | Rufous Chatterer | Timaliidae |
| Turdoides melanops | Black-faced Babbler | Timaliidae |
| Turdoides sharpei | Black-lored Babbler | Timaliidae |
| Turdoides reinwardtii | Blackcap Babbler | Timaliidae |
| Turdoides plebejus | Brown Babbler | Timaliidae |
| Turdoides leucocephala | White-headed Babbler | Timaliidae |
| Turdoides jardineii | Arrow-marked Babbler | Timaliidae |
| Turdoides squamulata | Scaly Babbler | Timaliidae |
| Turdoides hartlaubii | Hartlaub's Babbler | Timaliidae |
| Turdoides hypoleuca | Northern Pied Babbler | Timaliidae |
| Turdoides bicolor | Southern Pied Babbler | Timaliidae |
| Turdoides gymnogenys | Bare-cheeked Babbler | Timaliidae |
| Sylvia subcaerulea | Chestnut-vented Warbler | Sylviidae |
| Sylvia boehmi | Banded Warbler | Sylviidae |
| Zosterops abyssinicus | Abyssinian White-eye | Zosteropidae |
| Zosterops capensis | Cape White-eye | Zosteropidae |
| Zosterops senegalensis | African Yellow White-eye | Zosteropidae |
| Hyliota flavigaster | Yellow-bellied Hyliota | Hyliotidae |
| Hyliota australis | Southern Hyliota | Hyliotidae |
| Salpornis spilonotus | Spotted Creeper | Certhiidae |
| Creatophora cinerea | Wattled Starling | Sturnidae |
| Lamprotornis nitens | Cape Starling | Sturnidae |
| Lamprotornis chalybaeus | Greater Blue-eared Starling | Sturnidae |
| Lamprotornis chloropterus | Lesser Blue-eared Starling | Sturnidae |
| Lamprotornis elisabeth | Miombo Blue-eared Starling | Sturnidae |
| Lamprotornis chalcurus | Bronze-tailed Starling | Sturnidae |
| Lamprotornis iris | Emerald Starling | Sturnidae |
| Lamprotornis purpureus | Purple Starling | Sturnidae |
| Lamprotornis purpuroptera | Rüppell's Starling | Sturnidae |
| Lamprotornis caudatus | Long-tailed Glossy Starling | Sturnidae |
| Lamprotornis regius | Golden-breasted Starling | Sturnidae |
| Lamprotornis mevesii | Meves's Starling | Sturnidae |
| Lamprotornis australis | Burchell's Starling | Sturnidae |
| Lamprotornis superbus | Superb Starling | Sturnidae |
| Lamprotornis hildebrandti | Hildebrandt's Starling | Sturnidae |
| Lamprotornis shelleyi | Shelley's Starling | Sturnidae |
| Lamprotornis pulcher | Chestnut-bellied Starling | Sturnidae |
| Lamprotornis unicolor | Ashy Starling | Sturnidae |
| Lamprotornis fischeri | Fischer's Starling | Sturnidae |
| Lamprotornis bicolor | Pied Starling | Sturnidae |
| Lamprotornis albicapillus | White-crowned Starling | Sturnidae |
| Cinnyricinclus leucogaster | Violet-backed Starling | Sturnidae |
| Onychognathus morio | Red-winged Starling | Sturnidae |
| Onychognathus neumanni | Neumann's Starling | Sturnidae |
| Grafisia torquata | White-collared Starling | Sturnidae |
| Speculipastor bicolor | Magpie Starling | Sturnidae |
| Neocichla gutturalis | Babbling Starling | Sturnidae |
| Buphagus africanus | Yellow-billed Oxpecker | Buphagidae |
| Buphagus erythrorhynchus | Red-billed Oxpecker | Buphagidae |
| Psophocichla litsitsirupa | Groundscraper Thrush | Turdidae |
| Turdus tephronotus | Bare-eyed Thrush | Turdidae |
| Turdus smithi | Karoo Thrush | Turdidae |
| Cossypha caffra | Cape Robin-Chat | Muscicapidae |
| Cossypha heuglini | White-browed Robin-Chat | Muscicapidae |
| Cossypha natalensis | Red-capped Robin-Chat | Muscicapidae |
| Cichladusa arquata | Collared Palm Thrush | Muscicapidae |
| Cichladusa ruficauda | Rufous-tailed Palm Thrush | Muscicapidae |
| Cichladusa guttata | Spotted Palm Thrush | Muscicapidae |
| Erythropygia barbata | Miombo Scrub Robin | Muscicapidae |
| Erythropygia hartlaubi | Brown-backed Scrub Robin | Muscicapidae |
| Erythropygia leucophrys | White-browed Scrub Robin | Muscicapidae |
| Erythropygia paena | Kalahari Scrub Robin | Muscicapidae |
| Namibornis herero | Herero Chat | Muscicapidae |
| Oenanthe pileata | Capped Wheatear | Muscicapidae |
| Oenanthe heuglini | Heuglin's Wheatear | Muscicapidae |
| Oenanthe phillipsi | Somali Wheatear | Muscicapidae |
| Oenanthe schalowi | Schalow's Wheatear | Muscicapidae |
| Oenanthe familiaris | Familiar Chat | Muscicapidae |
| Myrmecocichla tholloni | Congo Moor Chat | Muscicapidae |
| Myrmecocichla aethiops | Anteater Chat | Muscicapidae |
| Myrmecocichla formicivora | Ant-eating Chat | Muscicapidae |
| Myrmecocichla nigra | Sooty Chat | Muscicapidae |
| Myrmecocichla melaena | Rüppell's Black Chat | Muscicapidae |
| Pentholaea albifrons | White-fronted Black Chat | Muscicapidae |
| Pentholaea arnotti | Arnot's Chat | Muscicapidae |
| Pentholaea collaris | Ruaha Chat | Muscicapidae |
| Thamnolaea cinnamomeiventris | Mocking Cliff Chat | Muscicapidae |
| Thamnolaea coronata | White-crowned Cliff Chat | Muscicapidae |
| Pinarornis plumosus | Boulder Chat | Muscicapidae |
| Monticola semirufus | White-winged Cliff-Chat | Muscicapidae |
| Monticola rupestris | Cape Rock Thrush | Muscicapidae |
| Monticola explorator | Sentinel Rock Thrush | Muscicapidae |
| Monticola brevipes | Short-toed Rock Thrush | Muscicapidae |
| Monticola angolensis | Miombo Rock Thrush | Muscicapidae |
| Monticola pretoriae | Pretoria Rock Thrush | Muscicapidae |
| Bradornis pallidus | Pale Flycatcher | Muscicapidae |
| Bradornis microrhynchus | African Grey Flycatcher | Muscicapidae |
| Bradornis pumilus | Ethiopian Grey Flycatcher | Muscicapidae |
| Bradornis mariquensis | Marico Flycatcher | Muscicapidae |
| Sigelus silens | Fiscal Flycatcher | Muscicapidae |
| Empidornis semipartitus | Silverbird | Muscicapidae |
| Muscicapa gambagae | Gambaga Flycatcher | Muscicapidae |
| Muscicapa caerulescens | Ashy Flycatcher | Muscicapidae |
| Muscicapa aquatica | Swamp Flycatcher | Muscicapidae |
| Muscicapa boehmi | Böhm's Flycatcher | Muscicapidae |
| Anthreptes anchietae | Anchieta's Sunbird | Nectariniidae |
| Anthreptes longuemarei | Western Violet-backed Sunbird | Nectariniidae |
| Anthreptes orientalis | Eastern Violet-backed Sunbird | Nectariniidae |
| Anthreptes aurantis | Violet-tailed Sunbird | Nectariniidae |
| Chalcomitra amethystina | Amethyst Sunbird | Nectariniidae |
| Chalcomitra senegalensis | Scarlet-chested Sunbird | Nectariniidae |
| Chalcomitra hunteri | Hunter's Sunbird | Nectariniidae |
| Nectarinia bocagii | Bocage's Sunbird | Nectariniidae |
| Cinnyris manoensis | Miombo Double-collared Sunbird | Nectariniidae |
| Cinnyris neergaardi | Neergaard's Sunbird | Nectariniidae |
| Cinnyris afer | Greater Double-collared Sunbird | Nectariniidae |
| Cinnyris pulchellus | Beautiful Sunbird | Nectariniidae |
| Cinnyris mariquensis | Marico Sunbird | Nectariniidae |
| Cinnyris hofmanni | Hofmann's Sunbird | Nectariniidae |
| Cinnyris erythrocercus | Red-chested Sunbird | Nectariniidae |
| Cinnyris nectarinioides | Black-bellied Sunbird | Nectariniidae |
| Cinnyris bifasciatus | Purple-banded Sunbird | Nectariniidae |
| Cinnyris tsavoensis | Tsavo Sunbird | Nectariniidae |
| Cinnyris habessinicus | Shining Sunbird | Nectariniidae |
| Cinnyris johannae | Johanna's Sunbird | Nectariniidae |
| Cinnyris oustaleti | Oustalet's Sunbird | Nectariniidae |
| Cinnyris talatala | White-bellied Sunbird | Nectariniidae |
| Cinnyris venustus | Variable Sunbird | Nectariniidae |
| Cinnyris cupreus | Copper Sunbird | Nectariniidae |
| Plocepasser mahali | White-browed Sparrow-Weaver | Passeridae |
| Plocepasser superciliosus | Chestnut-crowned Sparrow-Weaver | Passeridae |
| Plocepasser donaldsoni | Donaldson-Smith's Sparrow-Weaver | Passeridae |
| Plocepasser rufoscapulatus | Chestnut-backed Sparrow-Weaver | Passeridae |
| Histurgops ruficauda | Rufous-tailed Weaver | Passeridae |
| Pseudonigrita arnaudi | Grey-capped Social Weaver | Passeridae |
| Pseudonigrita cabanisi | Black-capped Social Weaver | Passeridae |
| Philetairus socius | Sociable Weaver | Passeridae |
| Passer domesticus | House Sparrow | Passeridae |
| Passer castanopterus | Somali Sparrow | Passeridae |
| Passer motitensis | Great Sparrow | Passeridae |
| Passer rufocinctus | Kenya Sparrow | Passeridae |
| Passer shelleyi | Shelley's Sparrow | Passeridae |
| Passer melanurus | Cape Sparrow | Passeridae |
| Passer griseus | Northern Grey-headed Sparrow | Passeridae |
| Passer swainsonii | Swainson's Sparrow | Passeridae |
| Passer gongonensis | Parrot-billed Sparrow | Passeridae |
| Passer suahelicus | Swahili Sparrow | Passeridae |
| Passer diffusus | Southern Grey-headed Sparrow | Passeridae |
| Passer simplex | Desert Sparrow | Passeridae |
| Passer eminibey | Chestnut Sparrow | Passeridae |
| Gymnoris superciliaris | Yellow-throated Petronia | Passeridae |
| Gymnoris dentata | Bush Petronia | Passeridae |
| Gymnoris pyrgita | Yellow-spotted Petronia | Passeridae |
| Bubalornis albirostris | White-billed Buffalo Weaver | Ploceidae |
| Bubalornis niger | Red-billed Buffalo Weaver | Ploceidae |
| Dinemellia dinemelli | White-headed Buffalo Weaver | Ploceidae |
| Sporopipes squamifrons | Scaly-feathered Weaver | Ploceidae |
| Sporopipes frontalis | Speckle-fronted Weaver | Ploceidae |
| Ploceus nigrimentus | Black-chinned Weaver | Ploceidae |
| Ploceus pelzelni | Slender-billed Weaver | Ploceidae |
| Ploceus subpersonatus | Loango Weaver | Ploceidae |
| Ploceus luteolus | Little Weaver | Ploceidae |
| Ploceus nigricollis | Black-necked Weaver | Ploceidae |
| Ploceus capensis | Cape Weaver | Ploceidae |
| Ploceus xanthops | Holub's Golden Weaver | Ploceidae |
| Ploceus aurantius | Orange Weaver | Ploceidae |
| Ploceus heuglini | Heuglin's Masked Weaver | Ploceidae |
| Ploceus bojeri | Golden Palm Weaver | Ploceidae |
| Ploceus castaneiceps | Taveta Weaver | Ploceidae |
| Ploceus castanops | Northern Brown-throated Weaver | Ploceidae |
| Ploceus xanthopterus | Southern Brown-throated Weaver | Ploceidae |
| Ploceus galbula | Rüppell's Weaver | Ploceidae |
| Ploceus taeniopterus | Northern Masked Weaver | Ploceidae |
| Ploceus intermedius | Lesser Masked Weaver | Ploceidae |
| Ploceus velatus | Southern Masked Weaver | Ploceidae |
| Ploceus reichardi | Tanzanian Masked Weaver | Ploceidae |
| Ploceus vitellinus | Vitelline Masked Weaver | Ploceidae |
| Ploceus spekei | Speke's Weaver | Ploceidae |
| Ploceus cucullatus | Village Weaver | Ploceidae |
| Ploceus melanocephalus | Black-headed Weaver | Ploceidae |
| Ploceus dichrocephalus | Juba Weaver | Ploceidae |
| Ploceus jacksoni | Golden-backed Weaver | Ploceidae |
| Ploceus badius | Cinnamon Weaver | Ploceidae |
| Ploceus rubiginosus | Chestnut Weaver | Ploceidae |
| Ploceus aureonucha | Golden-naped Weaver | Ploceidae |
| Ploceus superciliosus | Compact Weaver | Ploceidae |
| Ploceus olivaceiceps | Olive-headed Weaver | Ploceidae |
| Ploceus angolensis | Bar-winged Weaver | Ploceidae |
| Anaplectes rubriceps | Red-headed Weaver | Ploceidae |
| Quelea cardinalis | Cardinal Quelea | Ploceidae |
| Quelea erythrops | Red-headed Quelea | Ploceidae |
| Quelea quelea | Red-billed Quelea | Ploceidae |
| Euplectes gierowii | Black Bishop | Ploceidae |
| Euplectes nigroventris | Zanzibar Red Bishop | Ploceidae |
| Euplectes hordeaceus | Black-winged Red Bishop | Ploceidae |
| Euplectes orix | Southern Red Bishop | Ploceidae |
| Euplectes franciscanus | Northern Red Bishop | Ploceidae |
| Euplectes aureus | Golden-backed Bishop | Ploceidae |
| Euplectes capensis | Yellow Bishop | Ploceidae |
| Euplectes axillaris | Fan-tailed Widowbird | Ploceidae |
| Euplectes albonotatus | White-winged Widowbird | Ploceidae |
| Euplectes macroura | Yellow-mantled Widowbird | Ploceidae |
| Pytilia lineata | Red-billed Pytilia | Estrildidae |
| Pytilia phoenicoptera | Red-winged Pytilia | Estrildidae |
| Pytilia afra | Orange-winged Pytilia | Estrildidae |
| Pytilia melba | Green-winged Pytilia | Estrildidae |
| Amadina erythrocephala | Red-headed Finch | Estrildidae |
| Amadina fasciata | Cut-throat Finch | Estrildidae |
| Lagonosticta rara | Black-bellied Firefinch | Estrildidae |
| Lagonosticta rufopicta | Bar-breasted Firefinch | Estrildidae |
| Lagonosticta senegala | Red-billed Firefinch | Estrildidae |
| Lagonosticta sanguinodorsalis | Rock Firefinch | Estrildidae |
| Lagonosticta umbrinodorsalis | Chad Firefinch | Estrildidae |
| Lagonosticta virata | Mali Firefinch | Estrildidae |
| Lagonosticta rubricata | African Firefinch | Estrildidae |
| Lagonosticta landanae | Landana Firefinch | Estrildidae |
| Lagonosticta rhodopareia | Jameson's Firefinch | Estrildidae |
| Lagonosticta larvata | Black-faced Firefinch | Estrildidae |
| Lagonosticta vinacea | Grey Black-faced Firefinch | Estrildidae |
| Uraeginthus angolensis | Blue Waxbill | Estrildidae |
| Uraeginthus bengalus | Red-cheeked Cordon-bleu | Estrildidae |
| Uraeginthus cyanocephalus | Blue-capped Cordon-bleu | Estrildidae |
| Uraeginthus granatinus | Violet-eared Waxbill | Estrildidae |
| Uraeginthus ianthinogaster | Purple Grenadier | Estrildidae |
| Estrilda caerulescens | Lavender Waxbill | Estrildidae |
| Estrilda thomensis | Cinderella Waxbill | Estrildidae |
| Estrilda paludicola | Fawn-breasted Waxbill | Estrildidae |
| Estrilda melpoda | Orange-cheeked Waxbill | Estrildidae |
| Estrilda rhodopyga | Crimson-rumped Waxbill | Estrildidae |
| Estrilda troglodytes | Black-rumped Waxbill | Estrildidae |
| Estrilda astrild | Common Waxbill | Estrildidae |
| Estrilda erythronotos | Black-faced Waxbill | Estrildidae |
| Estrilda charmosyna | Black-cheeked Waxbill | Estrildidae |
| Amandava subflava | Orange-breasted Waxbill | Estrildidae |
| Ortygospiza atricollis | Black-faced Quail-Finch | Estrildidae |
| Ortygospiza fuscocrissa | African Quail-Finch | Estrildidae |
| Ortygospiza gabonensis | Black-chinned Quail-Finch | Estrildidae |
| Paludipasser locustella | Locust Finch | Estrildidae |
| Euodice cantans | African Silverbill | Estrildidae |
| Odontospiza caniceps | Grey-headed Silverbill | Estrildidae |
| Vidua chalybeata | Village Indigobird | Viduidae |
| Vidua purpurascens | Purple Indigobird | Viduidae |
| Vidua larvaticola | Barka Indigobird | Viduidae |
| Vidua funerea | Dusky Indigobird | Viduidae |
| Vidua codringtoni | Zambezi Indigobird | Viduidae |
| Vidua wilsoni | Wilson's Indigobird | Viduidae |
| Vidua nigeriae | Quailfinch Indigobird | Viduidae |
| Vidua maryae | Jos Plateau Indigobird | Viduidae |
| Vidua macroura | Pin-tailed Whydah | Viduidae |
| Vidua hypocherina | Steel-blue Whydah | Viduidae |
| Vidua fischeri | Straw-tailed Whydah | Viduidae |
| Vidua regia | Shaft-tailed Whydah | Viduidae |
| Vidua paradisaea | Long-tailed Paradise Whydah | Viduidae |
| Vidua orientalis | Sahel Paradise Whydah | Viduidae |
| Vidua interjecta | Exclamatory Paradise Whydah | Viduidae |
| Vidua togoensis | Togo Paradise Whydah | Viduidae |
| Vidua obtusa | Broad-tailed Paradise Whydah | Viduidae |
| Anomalospiza imberbis | Cuckoo Finch | Viduidae |
| Motacilla aguimp | African Pied Wagtail | Motacillidae |
| Tmetothylacus tenellus | Golden Pipit | Motacillidae |
| Macronyx capensis | Cape Longclaw | Motacillidae |
| Macronyx croceus | Yellow-throated Longclaw | Motacillidae |
| Macronyx aurantiigula | Pangani Longclaw | Motacillidae |
| Macronyx ameliae | Rosy-throated Longclaw | Motacillidae |
| Macronyx grimwoodi | Grimwood's Longclaw | Motacillidae |
| Anthus cinnamomeus | African Pipit | Motacillidae |
| Anthus latistriatus | Jackson's Pipit | Motacillidae |
| Anthus campestris | Tawny Pipit | Motacillidae |
| Anthus similis | Long-billed Pipit | Motacillidae |
| Anthus nyassae | Wood Pipit | Motacillidae |
| Anthus vaalensis | Buffy Pipit | Motacillidae |
| Anthus longicaudatus | Long-tailed Pipit | Motacillidae |
| Anthus leucophrys | Plain-backed Pipit | Motacillidae |
| Anthus lineiventris | Striped Pipit | Motacillidae |
| Anthus crenatus | African Rock Pipit | Motacillidae |
| Anthus brachyurus | Short-tailed Pipit | Motacillidae |
| Anthus caffer | Bushveld Pipit | Motacillidae |
| Anthus pseudosimilis | Kimberley Pipit | Motacillidae |
| Serinus flavivertex | Yellow-crowned Canary | Fringillidae |
| Crithagra capistrata | Black-faced Canary | Fringillidae |
| Crithagra leucopygia | White-rumped Seedeater | Fringillidae |
| Crithagra atrogularis | Black-throated Canary | Fringillidae |
| Crithagra citrinipectus | Lemon-breasted Canary | Fringillidae |
| Crithagra xanthopygia | Yellow-rumped Seedeater | Fringillidae |
| Crithagra reichenowi | Reichenow's Seedeater | Fringillidae |
| Crithagra flavigula | Yellow-throated Seedeater | Fringillidae |
| Crithagra xantholaema | Salvadori's Seedeater | Fringillidae |
| Crithagra mozambica | Yellow-fronted Canary | Fringillidae |
| Crithagra donaldsoni | Northern Grosbeak-Canary | Fringillidae |
| Crithagra buchanani | Southern Grosbeak-Canary | Fringillidae |
| Crithagra dorsostriata | White-bellied Canary | Fringillidae |
| Crithagra reichardi | Reichard's Seedeater | Fringillidae |
| Crithagra gularis | Streaky-headed Seedeater | Fringillidae |
| Crithagra canicapilla | West African Seedeater | Fringillidae |
| Crithagra mennelli | Black-eared Seedeater | Fringillidae |
| Emberiza impetuani | Lark-like Bunting | Emberizidae |
| Emberiza tahapisi | Cinnamon-breasted Bunting | Emberizidae |
| Emberiza vincenti | Vincent's Bunting | Emberizidae |
| Emberiza poliopleura | Somali Bunting | Emberizidae |
| Emberiza flaviventris | Golden-breasted Bunting | Emberizidae |
| Emberiza affinis | Brown-rumped Bunting | Emberizidae |
