# Supplementary material for: Pyrodiversity interacts with rainfall to increase bird and mammal richness in African savannas
Source: Ecol Lett. 2018 Feb 14;21(4):557–67. doi: 10.1111/ele.12921 (PMC5888149; doi:10.1111/ele.12921)
Supplement: Supplementary file 6 [file ELE-21-557-s006.docx]

Supplementary Material S4

The following tables present INLA model fixed effects summaries for each biodiversity category model run as means and cv. Abbreviations: cv, coefficient of variation; FRI, fire return interval; FRP, fire radiative power; mm/yr, GAM, generalized additive model; millimeters per year; NPP, net primary productivity; sd, standard deviation (1σ). As detailed explicitly in Beale et al 2010, Beale et al 2013, and generally in standard texts on spatial regression methods, when fitted properly this type of spatially explicit model usually models almost all the variance in the data through the combination of fixed and spatially explicit random effects. In our models >90% of the total variance is explained in each case, because species richness varies very smoothly across Africa. A calculation of maximum the percent variance explained by the fixed effects can be used to estimate the relative importance of fixed (explained) vs random (unexplained, but spatially structured) effects. In all models, the spatial random effect was strong (>70% of explained variance), but pseudo-r^2^ were also strong (mean 0.509, range 0.27, 0.66). The latter value is quoted above each model. wAIC is a measure of model support equivalent to the well-known AIC score but appropriate to Bayesian models that can be used to compare relative support for different models of the same data: this is presented alongside pseudo-r^2^. Joint estimation of the spatial error term and fixed effects enables accurate computation of fixed effects but the relatively strong spatial effects modelled mean comparison of the raw data with the confidence intervals of the parameters may be misleading: to the naïve eye, confidence intervals may be more precisely estimated than raw data seems to imply possible.

| **ID** | **Model name** | **Dependent variable** | **Independent variables** |
| --- | --- | --- | --- |
| 1 | mamm.all_mean_raw_mod | Mammals | Pyrodiversity, ruggedness, NPP(GAM), and means of FRI, FRP, seasonality, area |
| 2 | mamm.all_cv_raw_mod |  | Pyrodiversity, ruggedness, NPP(GAM), and coefficient of variation in FRI, FRP, seasonality, area |
| 3 | mamm.nobats_mean_raw_mod | Mammals: no bats | Pyrodiversity, ruggedness, NPP(GAM), and means of FRI, FRP, seasonality, area |
| 4 | mamm.nobats_cv_raw_mod |  | Pyrodiversity, ruggedness, NPP(GAM), and coefficient of variation in FRI, FRP, seasonality, area |
| 5 | mamm.bats_mean_raw_mod | Mammals: bats | Pyrodiversity, ruggedness, NPP(GAM), and means of FRI, FRP, seasonality, area |
| 6 | mamm.bats_cv_raw_mod |  | Pyrodiversity, ruggedness, NPP(GAM), and coefficient of variation in FRI, FRP, seasonality, area |
| 7 | mamm.comm_mean_raw_mod | Common mammals | Pyrodiversity, ruggedness, NPP(GAM), and means of FRI, FRP, seasonality, area |
| 8 | mamm.comm_cv_raw_mod |  | Pyrodiversity, ruggedness, NPP(GAM), and coefficient of variation in FRI, FRP, seasonality, area |
| 9 | mamm.heavy_mean_raw_mod | Large Mammals | Pyrodiversity, ruggedness, NPP(GAM), and means of FRI, FRP, seasonality, area |
| 10 | mamm.heavy_cv_raw_mod |  | Pyrodiversity, ruggedness, NPP(GAM), and coefficient of variation in FRI, FRP, seasonality, area |
| 11 | mamm.light_mean_raw_mod | Small mammals | Pyrodiversity, ruggedness, NPP(GAM), and means of FRI, FRP, seasonality, area |
| 12 | mamm.light_cv_raw_mod |  | Pyrodiversity, ruggedness, NPP(GAM), and coefficient of variation in FRI, FRP, seasonality, area |
| 13 | birds.comm_mean_raw_mod | Birds | Pyrodiversity, ruggedness, NPP(GAM), and means of FRI, FRP, seasonality, area |
| 14 | birds.div_cv_raw_mod |  | Pyrodiversity, ruggedness, NPP(GAM), and coefficient of variation in FRI, FRP, seasonality, area |
| 15 | birds.comm_mean_raw_mod | Common birds | Pyrodiversity, ruggedness, NPP(GAM), and means of FRI, FRP, seasonality, area |
| 16 | birds.comm_cv_raw_mod |  | Pyrodiversity, ruggedness, NPP(GAM), and coefficient of variation in FRI, FRP, seasonality, area |
| 17 | mamm.all_mean_raw_mod_poly | Mammals | Pyrodiversity, ruggedness, NPP(GAM), and quadratics of means of FRI, FRP, seasonality, area |
| 18 | mamm.all_cv_raw_mod_poly |  | Pyrodiversity, ruggedness, NPP(GAM), and quadratics of coefficient of variation in FRI, FRP, seasonality, area |
| 19 | mamm.nobats_mean_raw_mod_poly | Mammals: no bats | Pyrodiversity, ruggedness, NPP(GAM), and quadratics of means of FRI, FRP, seasonality, area |
| 20 | mamm.nobats_cv_raw_mod_poly |  | Pyrodiversity, ruggedness, NPP(GAM), and quadratics of coefficient of variation in FRI, FRP, seasonality, area |
| 21 | mamm.bats_mean_raw_mod_poly | Mammals: bats | Pyrodiversity, ruggedness, NPP(GAM), and quadratics of means of FRI, FRP, seasonality, area |
| 22 | mamm.bats_cv_raw_mod_poly |  | Pyrodiversity, ruggedness, NPP(GAM), and quadratics of coefficient of variation in FRI, FRP, seasonality, area |
| 23 | mamm.comm_mean_raw_mod_poly | Common mammals | Pyrodiversity, ruggedness, NPP(GAM), and quadratics of means of FRI, FRP, seasonality, area |
| 24 | mamm.comm_cv_raw_mod_poly |  | Pyrodiversity, ruggedness, NPP(GAM), and quadratics of coefficient of variation in FRI, FRP, seasonality, area |
| 25 | mamm.heavy_mean_raw_mod_poly | Large Mammals | Pyrodiversity, ruggedness, NPP(GAM), and quadratics of means of FRI, FRP, seasonality, area |
| 26 | mamm.heavy_cv_raw_mod_poly |  | Pyrodiversity, ruggedness, NPP(GAM), and quadratics of coefficient of variation in FRI, FRP, seasonality, area |
| 27 | mamm.light_mean_raw_mod_poly | Small mammals | Pyrodiversity, ruggedness, NPP(GAM), and quadratics of means of FRI, FRP, seasonality, area |
| 28 | mamm.light_cv_raw_mod_poly |  | Pyrodiversity, ruggedness, NPP(GAM), and quadratics of coefficient of variation in FRI, FRP, seasonality, area |
| 29 | birds.comm_mean_raw_mod_poly | Birds | Pyrodiversity, ruggedness, NPP(GAM), and quadratics of means of FRI, FRP, seasonality, area |
| 30 | birds.div_cv_raw_mod_poly |  | Pyrodiversity, ruggedness, NPP(GAM), and quadratics of coefficient of variation in FRI, FRP, seasonality, area |
| 31 | birds.comm_mean_raw_mod_poly | Common birds | Pyrodiversity, ruggedness, NPP(GAM), and quadratics of means of FRI, FRP, seasonality, area |
| 32 | birds.comm_cv_raw_mod_poly |  | Pyrodiversity, ruggedness, NPP(GAM), and quadratics of coefficient of variation in FRI, FRP, seasonality, area |

1 mamm.all_mean_raw_mod: pseudo R-square (fixed) = 0.546, wAIC = 3460

|  | mean | sd | 0.025 quantile | 0.5 quantile | 0.975 quantile |
| --- | --- | --- | --- | --- | --- |
| intercept | 4.544547 | 0.009409 | 4.526021 | 4.544564 | 4.56296 |
| pyrodiversity | 0.091337 | 0.01094 | 0.069991 | 0.091292 | 0.112917 |
| FRI | -0.05436 | 0.011047 | -0.07614 | -0.05433 | -0.03277 |
| FRP | -0.02626 | 0.012606 | -0.0512 | -0.02619 | -0.0017 |
| seasonality | 0.000934 | 0.009681 | -0.01806 | 0.00093 | 0.019936 |
| area | -0.07599 | 0.010189 | -0.09604 | -0.07598 | -0.05604 |
| precipitation <650 mm/yr | -0.08627 | 0.022059 | -0.12963 | -0.08625 | -0.04303 |
| ruggedness | 0.0641 | 0.00772 | 0.048947 | 0.064097 | 0.07925 |
| NPP spline 1 | -0.18972 | 0.010282 | -0.20994 | -0.18971 | -0.16958 |
| NPP spline 2 | 0.076408 | 0.00864 | 0.059367 | 0.076434 | 0.09329 |
| pyrodiversity:precipitation <650 mm/yr | -0.06217 | 0.016773 | -0.09534 | -0.06209 | -0.02947 |
| FRI:precipitation <650 mm/yr | -0.05311 | 0.017894 | -0.08832 | -0.05308 | -0.01807 |
| FRP:precipitation <650 mm/yr | -0.00791 | 0.017751 | -0.04256 | -0.00799 | 0.027102 |
| seasonality:precipitation <650 mm/yr | 0.087148 | 0.015966 | 0.055881 | 0.08712 | 0.118546 |
| area:precipitation <650 mm/yr | 0.080932 | 0.014909 | 0.051803 | 0.08088 | 0.110318 |

2 mamm.all_cv_raw_mod: pseudo R-square (fixed) = 0.538, wAIC = 3544

|  | mean | sd | 0.025 quantile | 0.5 quantile | 0.975 quantile |
| --- | --- | --- | --- | --- | --- |
| intercept | 4.558504 | 0.009437 | 4.539961 | 4.558509 | 4.577004 |
| pyrodiversity | 0.029161 | 0.013972 | 0.001743 | 0.029154 | 0.05659 |
| FRI | -0.00091 | 0.014386 | -0.02915 | -0.00091 | 0.02732 |
| FRP | 0.001799 | 0.01252 | -0.02262 | 0.00174 | 0.026516 |
| seasonality | 0.064378 | 0.01101 | 0.042808 | 0.06436 | 0.086027 |
| area | -0.00527 | 0.014725 | -0.03416 | -0.00528 | 0.023653 |
| precipitation <650 mm/yr | -0.11294 | 0.018937 | -0.15021 | -0.11291 | -0.07586 |
| ruggedness | 0.060227 | 0.007446 | 0.045596 | 0.06023 | 0.074828 |
| NPP spline 1 | -0.19382 | 0.009744 | -0.21301 | -0.1938 | -0.17475 |
| NPP spline 2 | 0.079664 | 0.00856 | 0.062793 | 0.079686 | 0.096399 |
| pyrodiversity:precipitation <650 mm/yr | 0.025418 | 0.019207 | -0.01235 | 0.025437 | 0.063052 |
| FRI:precipitation <650 mm/yr | -0.03218 | 0.018385 | -0.06833 | -0.03217 | 0.003847 |
| FRP:precipitation <650 mm/yr | 0.010477 | 0.016703 | -0.02244 | 0.010517 | 0.043141 |
| seasonality:precipitation <650 mm/yr | -0.11846 | 0.017085 | -0.1522 | -0.11839 | -0.08512 |
| area:precipitation <650 mm/yr | -0.04015 | 0.019004 | -0.07745 | -0.04015 | -0.00285 |

3 mamm.nobats_mean_raw_mod : pseudo R-square (fixed) = 0.520, wAIC = 2805

|  | mean | sd | 0.025 quantile | 0.5 quantile | 0.975 quantile |
| --- | --- | --- | --- | --- | --- |
| intercept | 4.228311 | 0.010634 | 4.207374 | 4.22833 | 4.249121 |
| pyrodiversity | 0.098838 | 0.01213 | 0.07507 | 0.098821 | 0.122677 |
| FRI | -0.01077 | 0.012436 | -0.03524 | -0.01075 | 0.013579 |
| FRP | 0.001481 | 0.013828 | -0.0258 | 0.001525 | 0.028496 |
| seasonality | -0.01012 | 0.011003 | -0.03174 | -0.01011 | 0.011453 |
| area | -0.04441 | 0.011521 | -0.06702 | -0.04441 | -0.0218 |
| precipitation <650 mm/yr | 0.000767 | 0.023666 | -0.04573 | 0.000776 | 0.047173 |
| ruggedness | 0.046361 | 0.008799 | 0.029047 | 0.046373 | 0.063592 |
| NPP spline 1 | -0.15091 | 0.011421 | -0.17338 | -0.1509 | -0.12855 |
| NPP spline 2 | 0.052648 | 0.009792 | 0.033334 | 0.052677 | 0.071782 |
| pyrodiversity:precipitation <650 mm/yr | -0.07882 | 0.017854 | -0.11399 | -0.07878 | -0.0439 |
| FRI:precipitation <650 mm/yr | -0.07043 | 0.01934 | -0.10845 | -0.07041 | -0.03253 |
| FRP:precipitation <650 mm/yr | -0.01283 | 0.018933 | -0.0499 | -0.01287 | 0.02441 |
| seasonality:precipitation <650 mm/yr | 0.082834 | 0.017332 | 0.048864 | 0.082813 | 0.116891 |
| area:precipitation <650 mm/yr | 0.051353 | 0.015862 | 0.020241 | 0.051341 | 0.082506 |

4 mamm.nobats_cv_raw_mod: pseudo R-square (fixed) = 0.525, wAIC = 2805

|  | mean | sd | 0.025 quantile | 0.5 quantile | 0.975 quantile |
| --- | --- | --- | --- | --- | --- |
| intercept | 4.236662 | 0.010792 | 4.215426 | 4.236678 | 4.257793 |
| pyrodiversity | 0.044043 | 0.015852 | 0.012931 | 0.044038 | 0.075155 |
| FRI | 0.026061 | 0.016409 | -0.00612 | 0.026049 | 0.058284 |
| FRP | -0.00424 | 0.014025 | -0.03163 | -0.00429 | 0.023416 |
| seasonality | 0.061561 | 0.012403 | 0.03719 | 0.061566 | 0.085884 |
| area | 0.024415 | 0.016737 | -0.00832 | 0.024369 | 0.057372 |
| precipitation <650 mm/yr | -0.01308 | 0.020775 | -0.05394 | -0.01306 | 0.027612 |
| ruggedness | 0.040714 | 0.008625 | 0.023726 | 0.040731 | 0.05759 |
| NPP spline 1 | -0.1621 | 0.010869 | -0.18351 | -0.16208 | -0.14084 |
| NPP spline 2 | 0.054576 | 0.009881 | 0.035083 | 0.054607 | 0.07388 |
| pyrodiversity:precipitation <650 mm/yr | -0.01305 | 0.021108 | -0.05454 | -0.01304 | 0.028325 |
| FRI:precipitation <650 mm/yr | -0.04833 | 0.020477 | -0.08858 | -0.04832 | -0.00819 |
| FRP:precipitation <650 mm/yr | 0.008033 | 0.018243 | -0.0279 | 0.00807 | 0.043727 |
| seasonality:precipitation <650 mm/yr | -0.09789 | 0.018322 | -0.134 | -0.09785 | -0.06206 |
| area:precipitation <650 mm/yr | -0.0379 | 0.021077 | -0.07936 | -0.03787 | 0.00339 |

5 mamm.bats_mean_raw_mod: pseudo R-square (fixed) = 0.545, wAIC = 2934

|  | mean | sd | 0.025 quantile | 0.5 quantile | 0.975 quantile |
| --- | --- | --- | --- | --- | --- |
| intercept | 3.213435 | 0.01869 | 3.176552 | 3.213497 | 3.24994 |
| pyrodiversity | 0.061808 | 0.019721 | 0.023241 | 0.061753 | 0.100639 |
| FRI | -0.15542 | 0.0207 | -0.19628 | -0.15535 | -0.11501 |
| FRP | -0.08698 | 0.02394 | -0.13446 | -0.08682 | -0.04044 |
| seasonality | 0.017677 | 0.017897 | -0.01746 | 0.017674 | 0.052798 |
| area | -0.14525 | 0.018994 | -0.18264 | -0.14522 | -0.10807 |
| precipitation <650 mm/yr | -0.4239 | 0.047216 | -0.51716 | -0.42371 | -0.33174 |
| ruggedness | 0.103692 | 0.014249 | 0.075704 | 0.103694 | 0.131646 |
| NPP spline 1 | -0.33055 | 0.02232 | -0.37458 | -0.33048 | -0.28694 |
| NPP spline 2 | 0.163038 | 0.0167 | 0.130155 | 0.163069 | 0.195718 |
| pyrodiversity:precipitation <650 mm/yr | 0.030861 | 0.034901 | -0.03798 | 0.030966 | 0.09906 |
| FRI:precipitation <650 mm/yr | -0.03975 | 0.038864 | -0.11624 | -0.03969 | 0.036349 |
| FRP:precipitation <650 mm/yr | -0.07981 | 0.037194 | -0.15263 | -0.07989 | -0.00664 |
| seasonality:precipitation <650 mm/yr | 0.17519 | 0.036374 | 0.10433 | 0.174996 | 0.247061 |
| area:precipitation <650 mm/yr | 0.134007 | 0.031493 | 0.072413 | 0.133921 | 0.196021 |

6 mamm.bats_cv_raw_mod: pseudo R-square (fixed) = 0.523, wAIC = 2934

|  | mean | sd | 0.025 quantile | 0.5 quantile | 0.975 quantile |
| --- | --- | --- | --- | --- | --- |
| intercept | 3.246539 | 0.018372 | 3.210337 | 3.246582 | 3.282471 |
| pyrodiversity | -0.00644 | 0.026043 | -0.05757 | -0.00645 | 0.044674 |
| FRI | -0.06914 | 0.026738 | -0.12169 | -0.06913 | -0.01671 |
| FRP | 0.019179 | 0.023949 | -0.02726 | 0.018977 | 0.066732 |
| seasonality | 0.067615 | 0.020658 | 0.027095 | 0.067595 | 0.108204 |
| area | -0.07662 | 0.027242 | -0.1301 | -0.07663 | -0.02313 |
| precipitation <650 mm/yr | -0.47688 | 0.041505 | -0.55882 | -0.47674 | -0.39584 |
| ruggedness | 0.103948 | 0.013778 | 0.07686 | 0.103958 | 0.130956 |
| NPP spline 1 | -0.30911 | 0.02133 | -0.35127 | -0.30902 | -0.26751 |
| NPP spline 2 | 0.165059 | 0.016499 | 0.132612 | 0.165077 | 0.19738 |
| pyrodiversity:precipitation <650 mm/yr | 0.204809 | 0.041094 | 0.124174 | 0.204788 | 0.285489 |
| FRI:precipitation <650 mm/yr | -0.03174 | 0.037978 | -0.1064 | -0.03172 | 0.042704 |
| FRP:precipitation <650 mm/yr | 0.04371 | 0.036084 | -0.02737 | 0.043785 | 0.114313 |
| seasonality:precipitation <650 mm/yr | -0.27761 | 0.041602 | -0.36026 | -0.27729 | -0.19685 |
| area:precipitation <650 mm/yr | -0.12755 | 0.038401 | -0.20297 | -0.12755 | -0.0522 |

7 mamm.comm_mean_raw_mod : pseudo R-square (fixed) = 0.662, wAIC = 3960

|  | mean | sd | 0.025 quantile | 0.5 quantile | 0.975 quantile |
| --- | --- | --- | --- | --- | --- |
| intercept | 4.221752 | 0.01092 | 4.200254 | 4.221772 | 4.243126 |
| pyrodiversity | 0.065939 | 0.012219 | 0.042015 | 0.065916 | 0.089974 |
| FRI | -0.06494 | 0.012483 | -0.08952 | -0.06492 | -0.04052 |
| FRP | -0.03123 | 0.014213 | -0.05928 | -0.03118 | -0.00347 |
| seasonality | -0.01877 | 0.011082 | -0.04055 | -0.01876 | 0.002957 |
| area | -0.0445 | 0.011624 | -0.06734 | -0.0445 | -0.02171 |
| precipitation <650 mm/yr | -0.13455 | 0.025369 | -0.18443 | -0.13453 | -0.08483 |
| ruggedness | 0.032543 | 0.008962 | 0.014916 | 0.032553 | 0.050099 |
| NPP spline 1 | -0.21614 | 0.012085 | -0.23993 | -0.21612 | -0.19248 |
| NPP spline 2 | 0.088322 | 0.010068 | 0.068469 | 0.08835 | 0.107997 |
| pyrodiversity:precipitation <650 mm/yr | -0.04462 | 0.019517 | -0.08311 | -0.04457 | -0.00648 |
| FRI:precipitation <650 mm/yr | -0.03574 | 0.020937 | -0.07689 | -0.03573 | 0.005295 |
| FRP:precipitation <650 mm/yr | -0.04938 | 0.020486 | -0.08952 | -0.04941 | -0.00911 |
| seasonality:precipitation <650 mm/yr | 0.053151 | 0.018583 | 0.016731 | 0.053127 | 0.089668 |
| area:precipitation <650 mm/yr | 0.073311 | 0.017042 | 0.039944 | 0.073277 | 0.106833 |

8 mamm.comm_cv_raw_mod: pseudo R-square (fixed) = 0.634, wAIC = 2966

|  | mean | sd | 0.025 quantile | 0.5 quantile | 0.975 quantile |
| --- | --- | --- | --- | --- | --- |
| intercept | 4.254307 | 0.010922 | 4.23284 | 4.254314 | 4.275716 |
| pyrodiversity | 0.014845 | 0.016002 | -0.01656 | 0.014837 | 0.046259 |
| FRI | -0.01936 | 0.016331 | -0.05139 | -0.01937 | 0.012714 |
| FRP | 0.009022 | 0.014567 | -0.01941 | 0.008961 | 0.037762 |
| seasonality | 0.047157 | 0.012681 | 0.022251 | 0.047158 | 0.072034 |
| area | 0.028303 | 0.017054 | -0.005 | 0.02824 | 0.061927 |
| precipitation <650 mm/yr | -0.22239 | 0.022403 | -0.2665 | -0.22235 | -0.17855 |
| ruggedness | 0.033266 | 0.008733 | 0.016074 | 0.033281 | 0.050362 |
| NPP spline 1 | -0.19981 | 0.011567 | -0.22261 | -0.19978 | -0.17719 |
| NPP spline 2 | 0.088315 | 0.010081 | 0.068442 | 0.088342 | 0.108023 |
| pyrodiversity:precipitation <650 mm/yr | 0.031635 | 0.022548 | -0.01269 | 0.031652 | 0.075825 |
| FRI:precipitation <650 mm/yr | 0.003525 | 0.021261 | -0.03825 | 0.003534 | 0.045211 |
| FRP:precipitation <650 mm/yr | -0.00875 | 0.019733 | -0.04761 | -0.00871 | 0.029866 |
| seasonality:precipitation <650 mm/yr | -0.08983 | 0.019904 | -0.12908 | -0.08978 | -0.05093 |
| area:precipitation <650 mm/yr | -0.11216 | 0.022247 | -0.156 | -0.1121 | -0.06865 |

9 mamm.heavy_mean_raw_mod : pseudo R-square (fixed) = 0.603, wAIC = 3126

|  | mean | sd | 0.025 quantile | 0.5 quantile | 0.975 quantile |
| --- | --- | --- | --- | --- | --- |
| intercept | 4.222504 | 0.011003 | 4.200844 | 4.222523 | 4.244042 |
| pyrodiversity | 0.086349 | 0.012459 | 0.062001 | 0.08631 | 0.110892 |
| FRI | -0.05264 | 0.012608 | -0.07747 | -0.05261 | -0.02798 |
| FRP | -0.03732 | 0.014489 | -0.06597 | -0.03725 | -0.00907 |
| seasonality | -0.00059 | 0.011141 | -0.02247 | -0.00059 | 0.021267 |
| area | -0.07413 | 0.011759 | -0.09727 | -0.07412 | -0.0511 |
| precipitation <650 mm/yr | -0.14783 | 0.025899 | -0.19883 | -0.14778 | -0.09714 |
| ruggedness | 0.055637 | 0.008922 | 0.038107 | 0.055641 | 0.073131 |
| NPP spline 1 | -0.23148 | 0.012376 | -0.25583 | -0.23146 | -0.20725 |
| NPP spline 2 | 0.086721 | 0.010293 | 0.066411 | 0.086755 | 0.106826 |
| pyrodiversity:precipitation <650 mm/yr | -0.04274 | 0.019408 | -0.08104 | -0.04267 | -0.00484 |
| FRI:precipitation <650 mm/yr | -0.06183 | 0.021029 | -0.10321 | -0.06181 | -0.02065 |
| FRP:precipitation <650 mm/yr | -0.02068 | 0.02065 | -0.06106 | -0.02074 | 0.019982 |
| seasonality:precipitation <650 mm/yr | 0.118161 | 0.019167 | 0.080665 | 0.118113 | 0.155889 |
| area:precipitation <650 mm/yr | 0.083393 | 0.017418 | 0.049345 | 0.083339 | 0.117707 |

10 mamm.heavy_cv_raw_mod : pseudo R-square (fixed) = 0.584, wAIC = 3219

|  | mean | sd | 0.025 quantile | 0.5 quantile | 0.975 quantile |
| --- | --- | --- | --- | --- | --- |
| intercept | 4.242089 | 0.011041 | 4.220383 | 4.242097 | 4.263727 |
| pyrodiversity | 0.029587 | 0.01613 | -0.00207 | 0.029579 | 0.061252 |
| FRI | 0.000922 | 0.016553 | -0.03154 | 0.000906 | 0.03344 |
| FRP | 0.007501 | 0.014502 | -0.02078 | 0.007434 | 0.036127 |
| seasonality | 0.054795 | 0.012694 | 0.02989 | 0.054787 | 0.079724 |
| area | 0.001971 | 0.016936 | -0.03118 | 0.001933 | 0.035301 |
| precipitation <650 mm/yr | -0.17138 | 0.022397 | -0.21549 | -0.17133 | -0.12756 |
| ruggedness | 0.0539 | 0.008619 | 0.036943 | 0.053911 | 0.070783 |
| NPP spline 1 | -0.23454 | 0.011837 | -0.25787 | -0.23451 | -0.21139 |
| NPP spline 2 | 0.091189 | 0.010255 | 0.070966 | 0.091218 | 0.111232 |
| pyrodiversity:precipitation <650 mm/yr | 0.047824 | 0.022498 | 0.003611 | 0.047836 | 0.091932 |
| FRI:precipitation <650 mm/yr | -0.04116 | 0.021511 | -0.08347 | -0.04114 | 0.000983 |
| FRP:precipitation <650 mm/yr | 0.005733 | 0.019676 | -0.03303 | 0.005775 | 0.044225 |
| seasonality:precipitation <650 mm/yr | -0.13804 | 0.020634 | -0.17882 | -0.13796 | -0.09779 |
| area:precipitation <650 mm/yr | -0.06107 | 0.022163 | -0.10466 | -0.06105 | -0.01764 |

11 mamm.light_mean_raw_mod: pseudo R-square (fixed) = 0.272, wAIC = 2363

|  | mean | sd | 0.025 quantile | 0.5 quantile | 0.975 quantile |
| --- | --- | --- | --- | --- | --- |
| intercept | 3.243524 | 0.01728 | 3.209426 | 3.24358 | 3.277275 |
| pyrodiversity | 0.091571 | 0.019365 | 0.053544 | 0.091572 | 0.129559 |
| FRI | -0.04818 | 0.020387 | -0.08833 | -0.04814 | -0.00829 |
| FRP | 0.013534 | 0.022099 | -0.03016 | 0.013635 | 0.05663 |
| seasonality | -0.00039 | 0.017793 | -0.03535 | -0.00038 | 0.034495 |
| area | -0.07415 | 0.018609 | -0.11062 | -0.07417 | -0.03758 |
| precipitation <650 mm/yr | 0.042313 | 0.037802 | -0.03202 | 0.042347 | 0.116389 |
| ruggedness | 0.080879 | 0.014001 | 0.053279 | 0.080915 | 0.108253 |
| NPP spline 1 | -0.09679 | 0.017902 | -0.13202 | -0.09676 | -0.06174 |
| NPP spline 2 | 0.057047 | 0.014794 | 0.027878 | 0.057088 | 0.085965 |
| pyrodiversity:precipitation <650 mm/yr | -0.08716 | 0.028371 | -0.14298 | -0.08712 | -0.0316 |
| FRI:precipitation <650 mm/yr | -0.0279 | 0.030981 | -0.08875 | -0.02789 | 0.032858 |
| FRP:precipitation <650 mm/yr | -0.00261 | 0.030057 | -0.06144 | -0.00268 | 0.056526 |
| seasonality:precipitation <650 mm/yr | 0.035868 | 0.027279 | -0.0176 | 0.035835 | 0.08947 |
| area:precipitation <650 mm/yr | 0.065735 | 0.025195 | 0.016231 | 0.065745 | 0.115136 |

12 mamm.light_cv_raw_mod: pseudo R-square (fixed) = 0.295, wAIC = 2362

|  | mean | sd | 0.025 quantile | 0.5 quantile | 0.975 quantile |
| --- | --- | --- | --- | --- | --- |
| intercept | 3.240645 | 0.017533 | 3.206071 | 3.240694 | 3.274913 |
| pyrodiversity | 0.02625 | 0.02532 | -0.02345 | 0.026243 | 0.075939 |
| FRI | -0.00098 | 0.026529 | -0.05306 | -0.00098 | 0.051071 |
| FRP | -0.01769 | 0.022196 | -0.0609 | -0.01781 | 0.026192 |
| seasonality | 0.083952 | 0.019698 | 0.045146 | 0.083994 | 0.122494 |
| area | -0.01837 | 0.026545 | -0.07025 | -0.01846 | 0.03395 |
| precipitation <650 mm/yr | 0.030731 | 0.033448 | -0.03505 | 0.030765 | 0.096261 |
| ruggedness | 0.07122 | 0.013767 | 0.044047 | 0.071267 | 0.098108 |
| NPP spline 1 | -0.10333 | 0.016821 | -0.13652 | -0.10328 | -0.07047 |
| NPP spline 2 | 0.056132 | 0.014769 | 0.027006 | 0.056175 | 0.084995 |
| pyrodiversity:precipitation <650 mm/yr | -0.01225 | 0.033712 | -0.07849 | -0.01223 | 0.053844 |
| FRI:precipitation <650 mm/yr | -0.0194 | 0.032778 | -0.08376 | -0.01941 | 0.044901 |
| FRP:precipitation <650 mm/yr | 0.03092 | 0.028808 | -0.02588 | 0.030999 | 0.087233 |
| seasonality:precipitation <650 mm/yr | -0.09036 | 0.028389 | -0.14626 | -0.0903 | -0.0348 |
| area:precipitation <650 mm/yr | 8.07E-05 | 0.033304 | -0.06542 | 0.000116 | 0.065332 |

13 birds.div_mean_raw_mod: pseudo R-square (fixed) = 0.423, wAIC = 5582

|  | mean | sd | 0.025 quantile | 0.5 quantile | 0.975 quantile |
| --- | --- | --- | --- | --- | --- |
| intercept | 5.360429 | 0.007429 | 5.34582 | 5.360436 | 5.374984 |
| pyrodiversity | 0.206964 | 0.009388 | 0.18858 | 0.206947 | 0.225422 |
| FRI | -0.01698 | 0.008565 | -0.03381 | -0.01697 | -0.00019 |
| FRP | -0.08499 | 0.011342 | -0.10733 | -0.08497 | -0.06281 |
| seasonality | 0.015809 | 0.008103 | -0.00008 | 0.015802 | 0.031723 |
| area | -0.04224 | 0.008282 | -0.05850 | -0.04224 | -0.026 |
| precipitation <650 mm/yr | -0.04732 | 0.020819 | -0.08842 | -0.04724 | -0.00668 |
| ruggedness | 0.076774 | 0.006216 | 0.06455 | 0.07678 | 0.08895 |
| NPP spline 1 | -0.14508 | 0.007394 | -0.15960 | -0.14508 | -0.13058 |
| NPP spline 2 | 0.017796 | 0.00682 | 0.00437 | 0.017808 | 0.031144 |
| pyrodiversity:precipitation <650 mm/yr | -0.08129 | 0.017411 | -0.11514 | -0.08141 | -0.04682 |
| FRI:precipitation <650 mm/yr | -0.16157 | 0.01752 | -0.19629 | -0.16146 | -0.1275 |
| FRP:precipitation <650 mm/yr | 0.046367 | 0.017102 | 0.01300 | 0.046295 | 0.080097 |
| seasonality:precipitation <650 mm/yr | 0.039256 | 0.012359 | 0.01503 | 0.039243 | 0.063536 |
| area:precipitation <650 mm/yr | 0.121224 | 0.015171 | 0.09167 | 0.121145 | 0.151187 |

14 birds.div_cv_raw_mod: pseudo R-square (fixed) = 0.473, wAIC = 5953

|  | mean | sd | 0.025 quantile | 0.5 quantile | 0.975 quantile |
| --- | --- | --- | --- | --- | --- |
| intercept | 5.38521 | 0.007094 | 5.371289 | 5.385207 | 5.399136 |
| pyrodiversity | 0.092351 | 0.012044 | 0.068757 | 0.092331 | 0.116034 |
| FRI | 0.085942 | 0.012483 | 0.061541 | 0.085903 | 0.110534 |
| FRP | 0.040802 | 0.011284 | 0.018891 | 0.040719 | 0.063166 |
| seasonality | 0.062236 | 0.008877 | 0.044806 | 0.062235 | 0.079655 |
| area | -0.01835 | 0.012705 | -0.04339 | -0.01832 | 0.006497 |
| precipitation <650 mm/yr | -0.10983 | 0.014819 | -0.13904 | -0.10979 | -0.08086 |
| ruggedness | 0.083821 | 0.005653 | 0.072733 | 0.083817 | 0.09492 |
| NPP spline 1 | -0.14946 | 0.006661 | -0.16256 | -0.14945 | -0.13641 |
| NPP spline 2 | 0.015998 | 0.006459 | 0.003266 | 0.016015 | 0.028625 |
| pyrodiversity:precipitation <650 mm/yr | -0.01327 | 0.016416 | -0.04549 | -0.01328 | 0.018957 |
| FRI:precipitation <650 mm/yr | -0.11292 | 0.015605 | -0.14366 | -0.11289 | -0.08238 |
| FRP:precipitation <650 mm/yr | -0.03559 | 0.014884 | -0.06514 | -0.03548 | -0.00668 |
| seasonality:precipitation <650 mm/yr | -0.06187 | 0.013141 | -0.08779 | -0.06183 | -0.03619 |
| area:precipitation <650 mm/yr | -0.0381 | 0.016269 | -0.06993 | -0.03814 | -0.00608 |

15 birds.comm_mean_raw_mod : pseudo R-square (fixed) = 0.528, wAIC = 3960

|  | mean | sd | 0.025 quantile | 0.5 quantile | 0.975 quantile |
| --- | --- | --- | --- | --- | --- |
| intercept | 4.920369 | 0.008153 | 4.904325 | 4.920381 | 4.936333 |
| pyrodiversity | 0.089551 | 0.009751 | 0.070528 | 0.089509 | 0.108788 |
| FRI | 0.009923 | 0.009495 | -0.00876 | 0.009937 | 0.028514 |
| FRP | -0.06665 | 0.011568 | -0.08955 | -0.06658 | -0.04412 |
| seasonality | -0.03296 | 0.008655 | -0.04995 | -0.03297 | -0.01597 |
| area | 0.027135 | 0.009076 | 0.0093 | 0.02714 | 0.044928 |
| precipitation <650 mm/yr | -0.07239 | 0.019704 | -0.1112 | -0.07235 | -0.03382 |
| ruggedness | 0.048722 | 0.006799 | 0.035383 | 0.048718 | 0.062072 |
| NPP spline 1 | -0.13162 | 0.008356 | -0.14805 | -0.13162 | -0.11525 |
| NPP spline 2 | 0.039859 | 0.007496 | 0.025064 | 0.039884 | 0.054499 |
| pyrodiversity:precipitation <650 mm/yr | -0.02877 | 0.015117 | -0.05844 | -0.02878 | 0.000914 |
| FRI:precipitation <650 mm/yr | -0.07869 | 0.01609 | -0.11045 | -0.07863 | -0.04726 |
| FRP:precipitation <650 mm/yr | -0.02753 | 0.016125 | -0.05904 | -0.02758 | 0.004251 |
| seasonality:precipitation <650 mm/yr | 0.03647 | 0.013343 | 0.010328 | 0.03645 | 0.0627 |
| area:precipitation <650 mm/yr | 0.043591 | 0.014236 | 0.016107 | 0.043433 | 0.071976 |

16 birds.comm_cv_raw_mod: pseudo R-square (fixed) = 0.474, wAIC = 4184

|  | mean | sd | 0.025 quantile | 0.5 quantile | 0.975 quantile |
| --- | --- | --- | --- | --- | --- |
| intercept | 4.96616 | 0.007953 | 4.950564 | 4.966154 | 4.98178 |
| pyrodiversity | 0.035753 | 0.012128 | 0.011991 | 0.035734 | 0.059598 |
| FRI | 0.05968 | 0.012633 | 0.035022 | 0.059629 | 0.084599 |
| FRP | 0.015384 | 0.010911 | -0.00588 | 0.01533 | 0.036931 |
| seasonality | 0.022954 | 0.009481 | 0.004275 | 0.022975 | 0.0415 |
| area | 0.045999 | 0.013151 | 0.020314 | 0.04595 | 0.071926 |
| precipitation <650 mm/yr | -0.18325 | 0.016123 | -0.21503 | -0.18321 | -0.15173 |
| ruggedness | 0.052519 | 0.006373 | 0.040011 | 0.052517 | 0.065026 |
| NPP spline 1 | -0.10939 | 0.007728 | -0.12459 | -0.10938 | -0.09425 |
| NPP spline 2 | 0.030454 | 0.00721 | 0.016232 | 0.030477 | 0.044539 |
| pyrodiversity:precipitation <650 mm/yr | 0.027422 | 0.016481 | -0.00495 | 0.027424 | 0.059753 |
| FRI:precipitation <650 mm/yr | -0.05619 | 0.015757 | -0.08723 | -0.05616 | -0.02536 |
| FRP:precipitation <650 mm/yr | -0.01059 | 0.014395 | -0.03901 | -0.01054 | 0.017514 |
| seasonality:precipitation <650 mm/yr | -0.02746 | 0.013747 | -0.05448 | -0.02745 | -0.0005 |
| area:precipitation <650 mm/yr | -0.12812 | 0.016682 | -0.1611 | -0.12804 | -0.09559 |

17 mamm.all_mean_raw_mod_poly: wAIC = 3155

|  | 0.025 quantile | 0.5 quantile | 0.975 quantile |
| --- | --- | --- | --- |
| (Intercept) | 5.098 | 8.322 | 11.55 |
| pyro | -11.31 | -7.991 | -4.674 |
| logFRI | -8.82 | -5.081 | -1.353 |
| logFRP | -2.597 | 0.309 | 3.213 |
| fireday | -6.357 | -2.53 | 1.289 |
| logArea | -4.322 | -1.734 | 0.848 |
| I(pyro^2) | 0.3008 | 3.832 | 7.367 |
| I(logFRI^2) | 1.198 | 3.472 | 5.739 |
| I(logFRP^2) | -0.7147 | 2.151 | 5.019 |
| I(fireday^2) | 0.6555 | 3.63 | 6.608 |
| I(logArea^2) | -1.414 | 7.138 | 15.69 |
| precipitation <650 mm/yr | 2.222 | 4.515 | 6.808 |
| rugg | -12.55 | -6.268 | 0.002856 |
| pyro:precipitation <650 mm/yr | -6.791 | -0.6253 | 5.53 |
| logFRI:precipitation <650 mm/yr | 2.019 | 7.971 | 13.94 |
| logFRP:precipitation <650 mm/yr | 2.019 | 7.971 | 13.94 |
| fireday:precipitation <650 mm/yr | -0.4618 | 3.805 | 8.07 |
| logArea:precipitation <650 mm/yr | -2.347 | 2.273 | 6.893 |
| I(pyro^2):precipitation <650 mm/yr | -2.342 | 1.15 | 4.64 |
| I(logFRI^2):precipitation <650 mm/yr | -7.698 | -3.464 | 0.7617 |
| I(logFRP^2):precipitation <650 mm/yr | -8.626 | -5.695 | -2.767 |
| I(fireday^2):precipitation <650 mm/yr | -5.182 | -1.38 | 2.415 |
| I(logArea^2):precipitation <650 mm/yr | -6.921 | -3.545 | -0.179 |

18 mamm.all_cv_raw_mod_poly: wAIC = 3520

|  | 0.025 quantile | 0.5 quantile | 0.975 quantile |
| --- | --- | --- | --- |
| (Intercept) | 4.539 | 4.562 | 4.584 |
| pyro | -0.004214 | 0.02489 | 0.05392 |
| logFRIcv | -0.01052 | 0.02243 | 0.05535 |
| logFRPcv | -0.0008636 | 0.03104 | 0.06329 |
| firedaycv | 0.03802 | 0.07146 | 0.105 |
| logAreacv | -0.01316 | 0.02649 | 0.06629 |
| I(pyro^2) | -0.006804 | 0.01247 | 0.03161 |
| I(logFRIcv^2) | -0.009318 | 0.008063 | 0.02521 |
| I(logFRPcv^2) | 0.004635 | 0.01277 | 0.02092 |
| I(firedaycv^2) | -0.03025 | -0.01482 | 0.0005782 |
| I(logAreacv^2) | 0.0009979 | 0.01311 | 0.02518 |
| precipitation <650 mm/yr | -0.172 | -0.1285 | -0.08545 |
| rugg | 0.04375 | 0.05857 | 0.07335 |
| pyro:precipitation <650 mm/yr | -0.2153 | -0.1943 | -0.1734 |
| logFRIcv:precipitation <650 mm/yr | 0.06661 | 0.08469 | 0.1026 |
| logFRPcv:precipitation <650 mm/yr | -0.009874 | 0.04171 | 0.09362 |
| firedaycv:precipitation <650 mm/yr | -0.1041 | -0.06122 | -0.0186 |
| logAreacv:precipitation <650 mm/yr | -0.04512 | -0.00458 | 0.03568 |
| I(pyro^2):precipitation <650 mm/yr | -0.1998 | -0.1494 | -0.09945 |
| I(logFRIcv^2):precipitation <650 mm/yr | -0.1009 | -0.05253 | -0.0042 |
| I(logFRPcv^2):precipitation <650 mm/yr | -0.04141 | -0.0151 | 0.01109 |
| I(firedaycv^2):precipitation <650 mm/yr | -0.009892 | 0.00984 | 0.0298 |
| I(logAreacv^2):precipitation <650 mm/yr | -0.02566 | -0.01441 | -0.003341 |

19 mamm.nobats_mean_raw_mod_poly : wAIC = 2805

|  | 0.025 quantile | 0.5 quantile | 0.975 quantile |
| --- | --- | --- | --- |
| (Intercept) | 4.128 | 4.172 | 4.215 |
| pyro | 0.07978 | 0.105 | 0.1302 |
| logFRI | -0.05594 | -0.02921 | -0.0027 |
| logFRP | -0.05189 | -0.02257 | 0.006549 |
| fireday | -0.03533 | -0.01184 | 0.01155 |
| logArea | -0.04761 | -0.01699 | 0.01347 |
| I(pyro^2) | -0.02591 | -0.00531 | 0.01498 |
| I(logFRI^2) | -0.01785 | 0.01048 | 0.03853 |
| I(logFRP^2) | 0.006982 | 0.02423 | 0.04122 |
| I(fireday^2) | -0.01198 | 0.01128 | 0.03448 |
| I(logArea^2) | 0.003925 | 0.02714 | 0.05033 |
| precipitation <650 mm/yr | 0.01021 | 0.08226 | 0.1543 |
| rugg | 0.0218 | 0.03989 | 0.05786 |
| z.npp1 | -0.1843 | -0.1599 | -0.1356 |
| z.npp2 | 0.04081 | 0.06165 | 0.08228 |
| pyro:precipitation <650 mm/yr | -0.1392 | -0.08678 | -0.03417 |
| logFRI:precipitation <650 mm/yr | -0.1074 | -0.05643 | -0.00544 |
| logFRP:precipitation <650 mm/yr | 0.0007197 | 0.04922 | 0.09876 |
| fireday:precipitation <650 mm/yr | 0.04345 | 0.07941 | 0.1155 |
| logArea:precipitation <650 mm/yr | -0.02025 | 0.01754 | 0.05544 |
| I(pyro^2):precipitation <650 mm/yr | -0.02652 | 0.001694 | 0.02991 |
| I(logFRI^2):precipitation <650 mm/yr | -0.04462 | -0.01029 | 0.02419 |
| I(logFRP^2):precipitation <650 mm/yr | -0.06731 | -0.04417 | -0.02118 |
| I(fireday^2):precipitation <650 mm/yr | -0.04352 | -0.01239 | 0.01865 |
| I(logArea^2):precipitation <650 mm/yr | -0.05598 | -0.02864 | -0.00144 |

20 mamm.nobats_cv_raw_mod: wAIC = 2828

|  | 0.025 quantile | 0.5 quantile | 0.975 quantile |
| --- | --- | --- | --- |
| (Intercept) | 4.217 | 4.243 | 4.269 |
| pyro | 0.005211 | 0.03792 | 0.0705 |
| logFRIcv | 0.002771 | 0.03993 | 0.07699 |
| logFRPcv | -0.01887 | 0.01676 | 0.05232 |
| firedaycv | 0.03645 | 0.07385 | 0.1113 |
| logAreacv | 0.01023 | 0.055 | 0.09981 |
| I(pyro^2) | -0.008454 | 0.01329 | 0.03482 |
| I(logFRIcv^2) | -0.01976 | 0.0002856 | 0.02003 |
| I(logFRPcv^2) | 0.0006428 | 0.00979 | 0.01879 |
| I(firedaycv^2) | -0.03356 | -0.01632 | 0.0007689 |
| I(logAreacv^2) | 0.0003821 | 0.01405 | 0.02749 |
| precipitation <650 mm/yr | -0.08 | -0.03277 | 0.01416 |
| rugg | 0.02229 | 0.03951 | 0.05659 |
| z.npp1 | -0.1876 | -0.1643 | -0.1413 |
| z.npp2 | 0.04049 | 0.06117 | 0.08163 |
| pyro:precipitation <650 mm/yr | -0.06235 | -0.007084 | 0.04853 |
| logFRIcv:precipitation <650 mm/yr | -0.1099 | -0.06284 | -0.01591 |
| logFRPcv:precipitation <650 mm/yr | -0.05018 | -0.00626 | 0.03765 |
| firedaycv:precipitation <650 mm/yr | -0.1825 | -0.1283 | -0.07437 |
| logAreacv:precipitation <650 mm/yr | -0.1082 | -0.05474 | -0.00124 |
| I(pyro^2):precipitation <650 mm/yr | -0.04004 | -0.0113 | 0.01738 |
| I(logFRIcv^2):precipitation <650 mm/yr | -0.008322 | 0.01395 | 0.03647 |
| I(logFRPcv^2):precipitation <650 mm/yr | -0.02301 | -0.01097 | 0.001004 |
| I(firedaycv^2):precipitation <650 mm/yr | -0.002092 | 0.01735 | 0.03684 |
| I(logAreacv^2):precipitation <650 mm/yr | -0.04001 | -0.02242 | -0.004849 |

21 mamm.bats_mean_raw_mod_poly: wAIC = 2851

|  | 0.025 quantile | 0.5 quantile | 0.975 quantile |
| --- | --- | --- | --- |
| (Intercept) | 2.969 | 3.05 | 3.129 |
| pyro | 0.03832 | 0.0821 | 0.1264 |
| logFRI | -0.2752 | -0.2252 | -0.1768 |
| logFRP | -0.2272 | -0.1699 | -0.1153 |
| fireday | -0.01726 | 0.02375 | 0.06474 |
| logArea | -0.1427 | -0.08891 | -0.0358 |
| I(pyro^2) | -0.08405 | -0.04611 | -0.009618 |
| I(logFRI^2) | 0.01974 | 0.07151 | 0.1236 |
| I(logFRP^2) | 0.02882 | 0.05984 | 0.09093 |
| I(fireday^2) | -0.02178 | 0.02061 | 0.06254 |
| I(logArea^2) | 0.03727 | 0.08022 | 0.1249 |
| precipitation <650 mm/yr | -0.1514 | 0.002284 | 0.1579 |
| rugg | 0.05987 | 0.09039 | 0.1209 |
| z.npp1 | -0.3951 | -0.3466 | -0.2987 |
| z.npp2 | 0.1556 | 0.1932 | 0.231 |
| pyro:precipitation <650 mm/yr | -0.08033 | 0.04448 | 0.1716 |
| logFRI:precipitation <650 mm/yr | -0.09765 | 0.0149 | 0.1283 |
| logFRP:precipitation <650 mm/yr | 0.06222 | 0.166 | 0.2763 |
| fireday:precipitation <650 mm/yr | 0.1 | 0.1789 | 0.2602 |
| logArea:precipitation <650 mm/yr | -0.07963 | -0.000576 | 0.07835 |
| I(pyro^2):precipitation <650 mm/yr | -0.06411 | -0.002861 | 0.05759 |
| I(logFRI^2):precipitation <650 mm/yr | -0.1695 | -0.09753 | -0.02646 |
| I(logFRP^2):precipitation <650 mm/yr | -0.213 | -0.1626 | -0.1143 |
| I(fireday^2):precipitation <650 mm/yr | -0.1237 | -0.0568 | 0.009932 |
| I(logArea^2):precipitation <650 mm/yr | -0.2033 | -0.1382 | -0.07668 |

22 mamm.bats_cv_raw_mod_poly: wAIC = 2975

|  | 0.025 quantile | 0.5 quantile | 0.975 quantile |
| --- | --- | --- | --- |
| (Intercept) | 3.209 | 3.252 | 3.294 |
| pyro | -0.0639 | -0.0089 | 0.04571 |
| logFRIcv | -0.08826 | -0.02625 | 0.03538 |
| logFRPcv | 0.004959 | 0.06522 | 0.1266 |
| firedaycv | 0.01491 | 0.07861 | 0.1426 |
| logAreacv | -0.1321 | -0.05765 | 0.01626 |
| I(pyro^2) | -0.02978 | 0.006317 | 0.04193 |
| I(logFRIcv^2) | -0.00242 | 0.02933 | 0.06054 |
| I(logFRPcv^2) | 0.004066 | 0.01924 | 0.03444 |
| I(firedaycv^2) | -0.04777 | -0.01849 | 0.01048 |
| I(logAreacv^2) | -0.01607 | 0.00642 | 0.02845 |
| precipitation <650 mm/yr | -0.5415 | -0.4427 | -0.3449 |
| rugg | 0.07598 | 0.1034 | 0.1307 |
| z.npp1 | -0.3494 | -0.303 | -0.2573 |
| z.npp2 | 0.1275 | 0.1626 | 0.1977 |
| pyro:precipitation <650 mm/yr | 0.1543 | 0.2753 | 0.3993 |
| logFRIcv:precipitation <650 mm/yr | -0.2181 | -0.1252 | -0.03342 |
| logFRPcv:precipitation <650 mm/yr | -0.02605 | 0.06131 | 0.149 |
| firedaycv:precipitation <650 mm/yr | -0.391 | -0.2763 | -0.1621 |
| logAreacv:precipitation <650 mm/yr | -0.2037 | -0.1042 | -0.00356 |
| I(pyro^2):precipitation <650 mm/yr | -0.0874 | -0.03001 | 0.02648 |
| I(logFRIcv^2):precipitation <650 mm/yr | -0.04228 | -0.00305 | 0.03657 |
| I(logFRPcv^2):precipitation <650 mm/yr | -0.05422 | -0.02696 | -0.00126 |
| I(firedaycv^2):precipitation <650 mm/yr | -0.02407 | 0.0228 | 0.06501 |
| I(logAreacv^2):precipitation <650 mm/yr | -0.109 | -0.07204 | -0.03629 |

23 mamm.comm_mean_raw_mod_poly : wAIC = 2862

|  | 0.025 quantile | 0.5 quantile | 0.975 quantile |
| --- | --- | --- | --- |
| (Intercept) | 4.139 | 4.183 | 4.227 |
| pyro | 0.04369 | 0.06934 | 0.09512 |
| logFRI | -0.1065 | -0.07951 | -0.05285 |
| logFRP | -0.07745 | -0.04704 | -0.017 |
| fireday | -0.03983 | -0.01592 | 0.007911 |
| logArea | -0.06225 | -0.03108 | -0.000152 |
| I(pyro^2) | -0.03725 | -0.01615 | 0.004592 |
| I(logFRI^2) | -0.00458 | 0.02449 | 0.05337 |
| I(logFRP^2) | -0.00293 | 0.01526 | 0.03315 |
| I(fireday^2) | -0.01762 | 0.00628 | 0.03003 |
| I(logArea^2) | -0.00911 | 0.0146 | 0.03834 |
| precipitation <650 mm/yr | -0.1014 | -0.02335 | 0.05471 |
| rugg | 0.01098 | 0.02942 | 0.04777 |
| z.npp1 | -0.2463 | -0.2204 | -0.1947 |
| z.npp2 | 0.07509 | 0.09668 | 0.1181 |
| pyro:precipitation <650 mm/yr | -0.1092 | -0.049 | 0.01137 |
| logFRI:precipitation <650 mm/yr | -0.08263 | -0.02671 | 0.02934 |
| logFRP:precipitation <650 mm/yr | -0.02331 | 0.02894 | 0.08246 |
| fireday:precipitation <650 mm/yr | 0.004223 | 0.0429 | 0.08173 |
| logArea:precipitation <650 mm/yr | 0.002957 | 0.04324 | 0.08374 |
| I(pyro^2):precipitation <650 mm/yr | -0.02464 | 0.006543 | 0.03764 |
| I(logFRI^2):precipitation <650 mm/yr | -0.06701 | -0.02999 | 0.007014 |
| I(logFRP^2):precipitation <650 mm/yr | -0.07441 | -0.04908 | -0.024 |
| I(fireday^2):precipitation <650 mm/yr | -0.04236 | -0.00878 | 0.0248 |
| I(logArea^2):precipitation <650 mm/yr | -0.05874 | -0.02948 | -0.000482 |

24 mamm.comm_cv_raw_mod_poly: wAIC = 2966

|  | 0.025 quantile | 0.5 quantile | 0.975 quantile |
| --- | --- | --- | --- |
| (Intercept) | 4.239 | 4.265 | 4.291 |
| pyro | -0.02505 | 0.00843 | 0.04179 |
| logFRIcv | -0.04846 | -0.01061 | 0.02714 |
| logFRPcv | -0.01556 | 0.02081 | 0.05725 |
| firedaycv | 0.02455 | 0.06255 | 0.1006 |
| logAreacv | 0.0002033 | 0.0452 | 0.09037 |
| I(pyro^2) | -0.01689 | 0.00511 | 0.0269 |
| I(logFRIcv^2) | -0.01886 | 0.001083 | 0.02077 |
| I(logFRPcv^2) | -0.002514 | 0.007164 | 0.01669 |
| I(firedaycv^2) | -0.03219 | -0.01456 | 0.002925 |
| I(logAreacv^2) | -0.005729 | 0.008333 | 0.02215 |
| precipitation <650 mm/yr | -0.2597 | -0.2087 | -0.1581 |
| rugg | 0.0148 | 0.03218 | 0.04944 |
| z.npp1 | -0.2212 | -0.1964 | -0.1719 |
| z.npp2 | 0.06885 | 0.09004 | 0.1111 |
| pyro:precipitation <650 mm/yr | 0.01892 | 0.08062 | 0.1429 |
| logFRIcv:precipitation <650 mm/yr | -0.08398 | -0.03378 | 0.01625 |
| logFRPcv:precipitation <650 mm/yr | -0.04477 | 0.002175 | 0.04903 |
| firedaycv:precipitation <650 mm/yr | -0.1763 | -0.1182 | -0.06046 |
| logAreacv:precipitation <650 mm/yr | -0.1725 | -0.1167 | -0.0611 |
| I(pyro^2):precipitation <650 mm/yr | -0.05223 | -0.02099 | 0.01005 |
| I(logFRIcv^2):precipitation <650 mm/yr | -0.01889 | 0.0039 | 0.02686 |
| I(logFRPcv^2):precipitation <650 mm/yr | -0.02097 | -0.00767 | 0.005486 |
| I(firedaycv^2):precipitation <650 mm/yr | 0.001712 | 0.02226 | 0.04282 |
| I(logAreacv^2):precipitation <650 mm/yr | -0.05174 | -0.03253 | -0.01344 |

25 mamm.heavy_mean_raw_mod_poly : wAIC = 3099

|  | 0.025 quantile | 0.5 quantile | 0.975 quantile |
| --- | --- | --- | --- |
| (Intercept) | 4.109 | 4.155 | 4.2 |
| pyro | 0.06396 | 0.09024 | 0.1168 |
| logFRI | -0.1053 | -0.07734 | -0.04985 |
| logFRP | -0.09804 | -0.06611 | -0.03496 |
| fireday | -0.02422 | 0.00008071 | 0.02436 |
| logArea | -0.08019 | -0.0482 | -0.01653 |
| I(pyro^2) | -0.04437 | -0.02243 | -0.00096 |
| I(logFRI^2) | -0.00570 | 0.02417 | 0.05393 |
| I(logFRP^2) | 0.006607 | 0.02502 | 0.04326 |
| I(fireday^2) | -0.00436 | 0.0202 | 0.04471 |
| I(logArea^2) | 0.007247 | 0.03172 | 0.05648 |
| precipitation <650 mm/yr | -0.06661 | 0.0132 | 0.09334 |
| rugg | 0.02967 | 0.04819 | 0.06665 |
| z.npp1 | -0.2639 | -0.2373 | -0.2108 |
| z.npp2 | 0.07602 | 0.09826 | 0.1203 |
| pyro:precipitation <650 mm/yr | -0.1023 | -0.04196 | 0.01846 |
| logFRI:precipitation <650 mm/yr | -0.09894 | -0.04169 | 0.0156 |
| logFRP:precipitation <650 mm/yr | 0.01904 | 0.07328 | 0.1296 |
| fireday:precipitation <650 mm/yr | 0.07042 | 0.1105 | 0.1509 |
| logArea:precipitation <650 mm/yr | -0.00059 | 0.04091 | 0.08272 |
| I(pyro^2):precipitation <650 mm/yr | -0.02123 | 0.01009 | 0.04136 |
| I(logFRI^2):precipitation <650 mm/yr | -0.06701 | -0.02932 | 0.008326 |
| I(logFRP^2):precipitation <650 mm/yr | -0.08444 | -0.05865 | -0.03329 |
| I(fireday^2):precipitation <650 mm/yr | -0.0731 | -0.03831 | -0.00362 |
| I(logArea^2):precipitation <650 mm/yr | -0.08024 | -0.0492 | -0.0188 |

26 mamm.heavy_cv_raw_mod_poly : wAIC = 3202

|  | 0.025 quantile | 0.5 quantile | 0.975 quantile |
| --- | --- | --- | --- |
| (Intercept) | 4.221 | 4.248 | 4.274 |
| pyro | -0.01011 | 0.02359 | 0.05719 |
| logFRIcv | -0.01224 | 0.02575 | 0.06376 |
| logFRPcv | -0.008079 | 0.02875 | 0.06584 |
| firedaycv | 0.02322 | 0.06188 | 0.1006 |
| logAreacv | -0.00569 | 0.03981 | 0.08544 |
| I(pyro^2) | -0.01381 | 0.008483 | 0.03059 |
| I(logFRIcv^2) | -0.0103 | 0.009742 | 0.02953 |
| I(logFRPcv^2) | 0.0005974 | 0.01012 | 0.01957 |
| I(firedaycv^2) | -0.0322 | -0.01443 | 0.003228 |
| I(logAreacv^2) | 0.002278 | 0.01611 | 0.0298 |
| precipitation <650 mm/yr | -0.2385 | -0.1869 | -0.1358 |
| rugg | 0.03492 | 0.05212 | 0.06921 |
| z.npp1 | -0.2583 | -0.2329 | -0.2076 |
| z.npp2 | 0.07414 | 0.09575 | 0.1171 |
| pyro:precipitation <650 mm/yr | 0.01202 | 0.07338 | 0.1353 |
| logFRIcv:precipitation <650 mm/yr | -0.1275 | -0.07703 | -0.02691 |
| logFRPcv:precipitation <650 mm/yr | -0.04213 | 0.005408 | 0.05281 |
| firedaycv:precipitation <650 mm/yr | -0.2247 | -0.1648 | -0.1054 |
| logAreacv:precipitation <650 mm/yr | -0.1282 | -0.07156 | -0.01488 |
| I(pyro^2):precipitation <650 mm/yr | -0.04474 | -0.01376 | 0.01704 |
| I(logFRIcv^2):precipitation <650 mm/yr | -0.01032 | 0.01269 | 0.03593 |
| I(logFRPcv^2):precipitation <650 mm/yr | -0.02638 | -0.01289 | 0.000391 |
| I(firedaycv^2):precipitation <650 mm/yr | -0.004671 | 0.01658 | 0.03775 |
| I(logAreacv^2):precipitation <650 mm/yr | -0.05888 | -0.03965 | -0.02062 |

27 mamm.light_mean_raw_mod_poly: wAIC = 2339

|  | 0.025 quantile | 0.5 quantile | 0.975 quantile |
| --- | --- | --- | --- |
| (Intercept) | 3.054 | 3.125 | 3.196 |
| pyro | 0.07534 | 0.1157 | 0.1559 |
| logFRI | -0.1328 | -0.0886 | -0.04488 |
| logFRP | -0.08941 | -0.04335 | 0.002495 |
| fireday | -0.04152 | -0.00331 | 0.03479 |
| logArea | -0.05992 | -0.01074 | 0.03832 |
| I(pyro^2) | -0.02358 | 0.008794 | 0.04059 |
| I(logFRI^2) | -0.01963 | 0.02581 | 0.07068 |
| I(logFRP^2) | 0.02473 | 0.05127 | 0.0773 |
| I(fireday^2) | -0.04107 | -0.00346 | 0.03385 |
| I(logArea^2) | 0.02168 | 0.05899 | 0.09619 |
| precipitation <650 mm/yr | 0.02606 | 0.1417 | 0.2571 |
| rugg | 0.04253 | 0.07132 | 0.09982 |
| z.npp1 | -0.1588 | -0.1205 | -0.08253 |
| z.npp2 | 0.04368 | 0.07534 | 0.1067 |
| pyro:precipitation <650 mm/yr | -0.1994 | -0.1166 | -0.03303 |
| logFRI:precipitation <650 mm/yr | -0.07287 | 0.007851 | 0.08894 |
| logFRP:precipitation <650 mm/yr | 0.03078 | 0.1089 | 0.1894 |
| fireday:precipitation <650 mm/yr | -0.02167 | 0.03511 | 0.09209 |
| logArea:precipitation <650 mm/yr | -0.0672 | -0.00718 | 0.0528 |
| I(pyro^2):precipitation <650 mm/yr | -0.05719 | -0.01255 | 0.03199 |
| I(logFRI^2):precipitation <650 mm/yr | -0.08085 | -0.02615 | 0.02884 |
| I(logFRP^2):precipitation <650 mm/yr | -0.118 | -0.08115 | -0.04482 |
| I(fireday^2):precipitation <650 mm/yr | -0.02302 | 0.02623 | 0.07551 |
| I(logArea^2):precipitation <650 mm/yr | -0.09082 | -0.04778 | -0.00490 |

28 mamm.light_cv_raw_mod_poly: wAIC = 2363

|  | 0.025 quantile | 0.5 quantile | 0.975 quantile |
| --- | --- | --- | --- |
| (Intercept) | 3.196 | 3.238 | 3.279 |
| pyro | -0.0246 | 0.02721 | 0.07871 |
| logFRIcv | -0.04819 | 0.01173 | 0.07114 |
| logFRPcv | -0.02655 | 0.02981 | 0.08572 |
| firedaycv | 0.04313 | 0.1027 | 0.1622 |
| logAreacv | -0.09338 | -0.02025 | 0.0524 |
| I(pyro^2) | -0.01062 | 0.02379 | 0.05767 |
| I(logFRIcv^2) | -0.0251 | 0.007015 | 0.0384 |
| I(logFRPcv^2) | 0.003747 | 0.01769 | 0.03129 |
| I(firedaycv^2) | -0.04796 | -0.02032 | 0.006857 |
| I(logAreacv^2) | -0.02228 | 0.0001212 | 0.0217 |
| precipitation <650 mm/yr | -0.05235 | 0.02281 | 0.0975 |
| rugg | 0.04355 | 0.07111 | 0.09831 |
| z.npp1 | -0.148 | -0.1118 | -0.07607 |
| z.npp2 | 0.03101 | 0.06207 | 0.09285 |
| pyro:precipitation <650 mm/yr | -0.1102 | -0.02303 | 0.06509 |
| logFRIcv:precipitation <650 mm/yr | -0.1025 | -0.02765 | 0.04725 |
| logFRPcv:precipitation <650 mm/yr | -0.08512 | -0.01631 | 0.05275 |
| firedaycv:precipitation <650 mm/yr | -0.2075 | -0.1229 | -0.03848 |
| logAreacv:precipitation <650 mm/yr | -0.07639 | 0.008772 | 0.09447 |
| I(pyro^2):precipitation <650 mm/yr | -0.064 | -0.01849 | 0.02691 |
| I(logFRIcv^2):precipitation <650 mm/yr | -0.03501 | 0.0001898 | 0.03597 |
| I(logFRPcv^2):precipitation <650 mm/yr | -0.03588 | -0.0175 | 0.0007174 |
| I(firedaycv^2):precipitation <650 mm/yr | -0.00837 | 0.0221 | 0.05279 |
| I(logAreacv^2):precipitation <650 mm/yr | -0.03012 | -0.002367 | 0.02557 |

29 birds.div_mean_raw_mod_poly: wAIC = 4956

|  | 0.025 quantile | 0.5 quantile | 0.975 quantile |
| --- | --- | --- | --- |
| (Intercept) | 5.233 | 5.266 | 5.299 |
| pyro | 0.1856 | 0.2048 | 0.2241 |
| logFRI | -0.09974 | -0.07976 | -0.05998 |
| logFRP | -0.1696 | -0.1434 | -0.1178 |
| fireday | -0.03096 | -0.0126 | 0.005723 |
| logArea | -0.04943 | -0.02559 | -0.00193 |
| I(pyro^2) | -0.07553 | -0.05666 | -0.03809 |
| I(logFRI^2) | -0.03313 | -0.00852 | 0.01573 |
| I(logFRP^2) | 0.03159 | 0.04948 | 0.06679 |
| I(fireday^2) | -0.01488 | 0.005596 | 0.02608 |
| I(logArea^2) | 0.07061 | 0.09214 | 0.1141 |
| precipitation <650 mm/yr | 0.002991 | 0.06971 | 0.1355 |
| rugg | 0.0496 | 0.06262 | 0.07558 |
| z.npp1 | -0.1896 | -0.1728 | -0.1561 |
| z.npp2 | 0.03155 | 0.04715 | 0.06266 |
| pyro:precipitation <650 mm/yr | -0.1195 | -0.06694 | -0.0134 |
| logFRI:precipitation <650 mm/yr | -0.1048 | -0.05671 | -0.00879 |
| logFRP:precipitation <650 mm/yr | 0.1654 | 0.2104 | 0.2563 |
| fireday:precipitation <650 mm/yr | 0.04462 | 0.073 | 0.1017 |
| logArea:precipitation <650 mm/yr | 0.009884 | 0.04452 | 0.07974 |
| I(pyro^2):precipitation <650 mm/yr | -0.00735 | 0.02001 | 0.04763 |
| I(logFRI^2):precipitation <650 mm/yr | -0.04598 | -0.01558 | 0.01495 |
| I(logFRP^2):precipitation <650 mm/yr | -0.136 | -0.1151 | -0.09413 |
| I(fireday^2):precipitation <650 mm/yr | 0.007864 | 0.03853 | 0.0694 |
| I(logArea^2):precipitation <650 mm/yr | -0.1144 | -0.08734 | -0.06083 |
|  |  |  |  |

30 birds.div_cv_raw_mod_poly: wAIC = 5847

|  | 0.025 quantile | 0.5 quantile | 0.975 quantile |
| --- | --- | --- | --- |
| (Intercept) | 5.389 | 5.407 | 5.425 |
| pyro | 0.04217 | 0.06898 | 0.09595 |
| logFRIcv | 0.02226 | 0.05245 | 0.08246 |
| logFRPcv | 0.03363 | 0.06351 | 0.0944 |
| firedaycv | 0.0756 | 0.1053 | 0.1345 |
| logAreacv | -0.06406 | -0.02903 | 0.005864 |
| I(pyro^2) | -0.03178 | -0.01367 | 0.00439 |
| I(logFRIcv^2) | -0.04651 | -0.02994 | -0.01397 |
| I(logFRPcv^2) | 0.005962 | 0.01433 | 0.02309 |
| I(firedaycv^2) | -0.02803 | -0.01315 | 0.002287 |
| I(logAreacv^2) | -0.01589 | -0.00384 | 0.008408 |
| precipitation <650 mm/yr | -0.1672 | -0.1315 | -0.09592 |
| rugg | 0.07148 | 0.08283 | 0.09417 |
| z.npp1 | -0.1723 | -0.1571 | -0.1418 |
| z.npp2 | 0.01819 | 0.03274 | 0.04715 |
| pyro:precipitation <650 mm/yr | 0.04737 | 0.09461 | 0.1432 |
| logFRIcv:precipitation <650 mm/yr | -0.1279 | -0.08978 | -0.05174 |
| logFRPcv:precipitation <650 mm/yr | -0.08996 | -0.04997 | -0.01166 |
| firedaycv:precipitation <650 mm/yr | -0.2164 | -0.1724 | -0.1295 |
| logAreacv:precipitation <650 mm/yr | -0.06603 | -0.02399 | 0.01798 |
| I(pyro^2):precipitation <650 mm/yr | -0.05414 | -0.02932 | -0.00511 |
| I(logFRIcv^2):precipitation <650 mm/yr | 0.01772 | 0.03572 | 0.0542 |
| I(logFRPcv^2):precipitation <650 mm/yr | -0.02438 | -0.01348 | -0.00287 |
| I(firedaycv^2):precipitation <650 mm/yr | 0.01487 | 0.03096 | 0.04682 |
| I(logAreacv^2):precipitation <650 mm/yr | -0.01205 | 0.003101 | 0.01824 |

31 birds.comm_mean_raw_mod_poly : wAIC = 3758

|  | 0.025 quantile | 0.5 quantile | 0.975 quantile |
| --- | --- | --- | --- |
| (Intercept) | 4.862 | 4.897 | 4.931 |
| pyro | 0.07736 | 0.09778 | 0.1184 |
| logFRI | -0.03799 | -0.01685 | 0.003983 |
| logFRP | -0.1173 | -0.09192 | -0.06724 |
| fireday | -0.05752 | -0.03835 | -0.01921 |
| logArea | -0.00086 | 0.024 | 0.0486 |
| I(pyro^2) | -0.05084 | -0.03261 | -0.01483 |
| I(logFRI^2) | -0.02099 | 0.002791 | 0.02641 |
| I(logFRP^2) | 0.01555 | 0.03071 | 0.0457 |
| I(fireday^2) | -0.025 | -0.00497 | 0.01512 |
| I(logArea^2) | 0.00234 | 0.02226 | 0.04255 |
| precipitation <650 mm/yr | -0.05248 | 0.009335 | 0.07129 |
| rugg | 0.02723 | 0.04138 | 0.0555 |
| z.npp1 | -0.1609 | -0.1425 | -0.1242 |
| z.npp2 | 0.03437 | 0.05094 | 0.06733 |
| pyro:precipitation <650 mm/yr | -0.04927 | -0.00132 | 0.04729 |
| logFRI:precipitation <650 mm/yr | -0.07654 | -0.03233 | 0.01207 |
| logFRP:precipitation <650 mm/yr | 0.05224 | 0.096 | 0.1412 |
| fireday:precipitation <650 mm/yr | 0.01689 | 0.04573 | 0.07491 |
| logArea:precipitation <650 mm/yr | -0.02739 | 0.004852 | 0.0377 |
| I(pyro^2):precipitation <650 mm/yr | -0.03157 | -0.00597 | 0.01959 |
| I(logFRI^2):precipitation <650 mm/yr | -0.06346 | -0.03359 | -0.00399 |
| I(logFRP^2):precipitation <650 mm/yr | -0.1057 | -0.08469 | -0.06397 |
| I(fireday^2):precipitation <650 mm/yr | 0.007057 | 0.03454 | 0.06226 |
| I(logArea^2):precipitation <650 mm/yr | -0.0554 | -0.03102 | -0.00724 |

32 birds.comm_cv_raw_mod_poly: wAIC = 4076

|  | 0.025 quantile | 0.5 quantile | 0.975 quantile |
| --- | --- | --- | --- |
| (Intercept) | 4.975 | 4.994 | 5.013 |
| pyro | -0.00804 | 0.01761 | 0.04322 |
| logFRIcv | -0.00492 | 0.02381 | 0.05268 |
| logFRPcv | -0.01874 | 0.008734 | 0.03642 |
| firedaycv | 0.04665 | 0.07537 | 0.104 |
| logAreacv | -0.02972 | 0.004807 | 0.0394 |
| I(pyro^2) | -0.037 | -0.01978 | -0.00269 |
| I(logFRIcv^2) | -0.03231 | -0.01685 | -0.00161 |
| I(logFRPcv^2) | -0.00748 | 0.000003373 | 0.00745 |
| I(firedaycv^2) | -0.03066 | -0.01702 | -0.00337 |
| I(logAreacv^2) | -0.0293 | -0.0176 | -0.00613 |
| precipitation <650 mm/yr | -0.2141 | -0.1775 | -0.1412 |
| rugg | 0.04075 | 0.05338 | 0.06601 |
| z.npp1 | -0.1314 | -0.1148 | -0.09819 |
| z.npp2 | 0.02161 | 0.03697 | 0.05214 |
| pyro:precipitation <650 mm/yr | 0.1064 | 0.1524 | 0.1992 |
| logFRIcv:precipitation <650 mm/yr | -0.08487 | -0.04789 | -0.01119 |
| logFRPcv:precipitation <650 mm/yr | -0.02043 | 0.01448 | 0.04903 |
| firedaycv:precipitation <650 mm/yr | -0.1567 | -0.1143 | -0.07282 |
| logAreacv:precipitation <650 mm/yr | -0.1269 | -0.08545 | -0.04443 |
| I(pyro^2):precipitation <650 mm/yr | -0.05689 | -0.03305 | -0.00951 |
| I(logFRIcv^2):precipitation <650 mm/yr | -0.00695 | 0.01027 | 0.02762 |
| I(logFRPcv^2):precipitation <650 mm/yr | -0.00753 | 0.002139 | 0.01177 |
| I(firedaycv^2):precipitation <650 mm/yr | 0.01849 | 0.03363 | 0.04893 |
| I(logAreacv^2):precipitation <650 mm/yr | -0.00844 | 0.006024 | 0.02059 |
